# Supplementary material for: Asymmetric biomimetic transamination of α-keto amides to peptides
Source: Nat Commun. 2021 Aug 30;12:5174. doi: 10.1038/s41467-021-25449-y (PMC8405696; doi:10.1038/s41467-021-25449-y)
Supplement: Supplementary file 1 — Supplementary Information [file 41467_2021_25449_MOESM1_ESM.pdf]

SUPPLEMENTARY INFORMATION

Asymmetric Biomimetic Transamination of α-Keto Amides to Peptides

Weiqi Cai[1,2], Xuelong Qiao[1,2], Hao Zhang[1], Bo Li[1], Jianhua Guo[1], Liangliang Zhang[1], Wen-Wen Chen[1] & Baoguo Zhao[1,*]

[1]The Education Ministry Key Lab of Resource Chemistry and Shanghai Key Laboratory of Rare Earth Functional Materials, Shanghai Normal University, Shanghai 200234, P. R. China

Email: zhaobg2006@shnu.edu.cn

[2]These authors contributed equally: Weiqi Cai, Xuelong Qiao

Table of Contents

I. Supplementary Methods S-2

1. General Information S-2
2. Preparation of Catalysts S-3
3. Preparation of α-Keto Amides 2 S-10
4. Procedure for Transamination of Glycinyl α-Keto Amides and α-Keto Phenylbutanamides S-29
5. Procedure for Transamination of Peptidyl α-Keto Amides S-44
6. Procedure for Divergent Extension of Ubenimex Methyl Ester S-48
7. Procedures for Successive Extension of Peptides S-51
8. Procedure for Comparison of Catalysts S-57
9. Determination of the Absolute Configurations of Transamination Products S-58
10. NMR Spectra S-66
11. Chromatograms for Determination of ee and dr Values S-137

II. Supplementary References S-182

I. Supplementary Methods

1. General Information

All commercially available reagents were used without further purification unless otherwise stated. dry THF were obtained by fresh distillation from sodium-benzophenone under argon atmosphere. CH_2Cl_2 was freshly distilled from CaH_2. Column chromatography was performed on silica gel (200-300 mesh). ^{1}H NMR spectra were recorded on a 400 or 600 MHz NMR spectrometer and ^{13}C NMR spectra were recorded on a 100 MHz or 150 MHz NMR spectrometer. ^{1}H shifts were referenced to $CDCl_3$ at 7.26 ppm and ^{13}C shifts were referenced to $CDCl_3$ at 77.16 ppm. Infrared spectra (IR) were recorded on a FT-IR Spectrometer. Melting points were uncorrected. Compound (*R*,*S*)-**5** and chiral pyridoxamine (*S*)-**1g** and were prepared by following the previously reported procedure.[1]

2. Preparation of Catalysts 1a-e and 1f Using (*R*)-1b as the Representative Example (Fig. 2 and 6b)

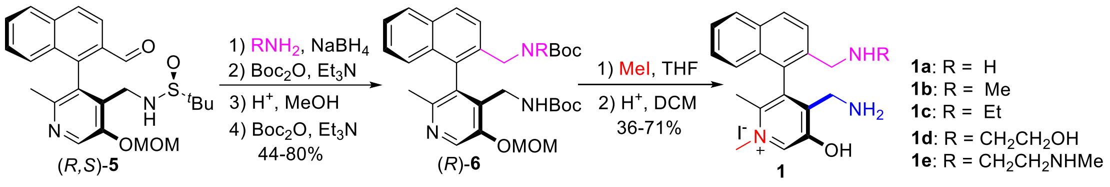

2.1 Synthesis of Compound (*R*)-6b (R = Me)

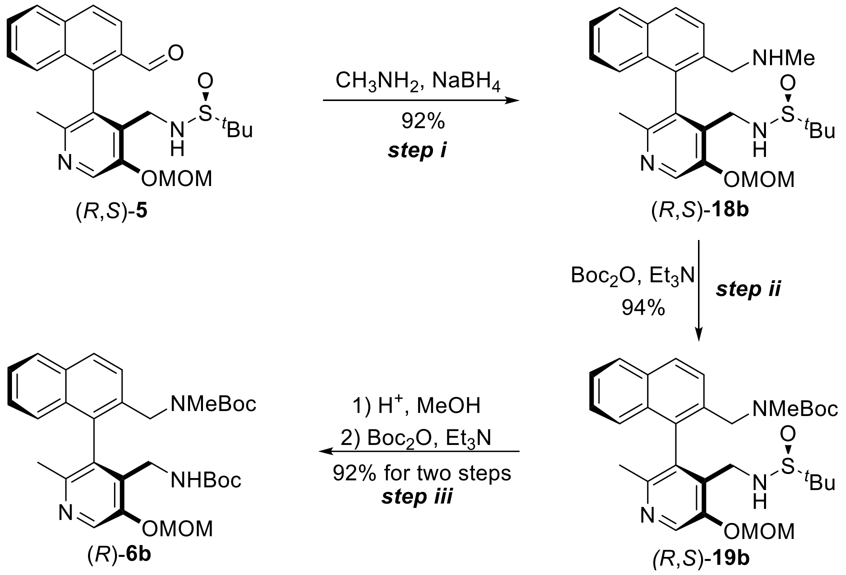

Step i. Synthesis of compound (*R*,*S*)-18b[1]

To a 25 mL round-bottom flask equipped with a magnetic stirrer bar were added aldehyde (*R*,*S*)-**5** (0.370 g, 0.841 mmol), methylamine aqueous solution (0.363 mL, 40 wt.% in H_2O), and MeOH (5 mL). After the mixture was stirred at room temperature for 5 h, $NaBH_4$ (0.0650 g, 4.22 mmol) was added in one portion at 0 °C. The resulting mixture was allowed to be warmed up to room temperature and stirred at the temperature overnight. The reaction was quenched with saturated aqueous NH_4Cl solution until no gas bubbled, the reaction mixture was concentrated via rotary evaporator to remove most of the solvent and extracted with DCM (20 mL × 3). The combined organic layers were dried over anhydrous Na_2SO_4, filtered, concentrated via rotary evaporator under reduced pressure, and purified by column chromatography on silica gel [DCM : MeOH : ammonia solution (2.9 M) in ethanol = 20:1:0.42] to give

(*R,S*)-**18b** (0.353 g, 92%) as a white solid. For pyridoxamine **1a**, the corresponding compound **18a** was prepared by following the previously reported procedure.[1]

(*R,S*)-**18b**: White solid; Mp: 28-30 °C; $[\alpha]_D^{25}$ = -18.5 (*c* = 0.10, $CHCl_3$); ^1H NMR (400 MHz, $CDCl_3$) δ 8.54 (s, 1H), 7.93 (d, *J* = 8.4 Hz, 1H), 7.90 (d, *J* = 8.0 Hz, 1H), 7.64 (d, *J* = 8.4 Hz, 1H), 7.47 (ddd, *J* = 8.4, 6.8, 1.2 Hz, 1H), 7.36 (ddd, *J* = 8.4, 6.8, 1.2 Hz, 1H), 7.09 (d, *J* = 8.4 Hz, 1H), 5.37 (d, *J* = 6.8 Hz, 1H), 5.34 (d, *J* = 6.8 Hz, 1H), 3.96 (d, *J* = 13.2 Hz, 1H), 3.59-3.53 (m, 5H), 3.44 (d, *J* = 13.2 Hz, 1H), 2.36 (s, 3H), 2.00 (s, 3H), 0.99 (s, 9H); ^{13}C NMR (100 MHz, $CDCl_3$) δ 150.95, 150.87, 136.2, 135.8, 135.5, 133.0, 132.8, 132.1, 128.9, 128.6, 127.04, 126.99, 126.2, 125.2, 95.7, 56.7, 55.9, 53.5, 41.8, 36.6, 22.7, 22.6; HRMS *m/z* Calcd. for $C_{25}H_{34}N_3O_3S$ (M + H^+): 456.2315; Found: 456.2318.

***Step ii*. Synthesis of compound (*R,S*)-19b**

To a stirred solution of (*R,S*)-**18b** (0.335 g, 0.736 mmol) in anhydrous DCM (5.0 mL) was added triethylamine (0.132 mL, 1.47 mmol) at 0 °C, followed by addition of a solution of di-*tert*-butyl dicarbonate (Boc_2O) (0.193 g, 0.885 mmol) in anhydrous DCM (1.0 mL) via a syringe [for pyridoxamine **1f**, acetyl anhydride (1.5 equiv) was added instead of the di-*tert*-butyl dicarbonate solution in DCM in the synthesis of the corresponding **19f**]. After being warm up to room temperature for 2 h, the mixture was evaporated in vacuo. The residue was purified by column chromatography on silica gel (ethyl acetate) to give compound (*R,S*)-**19b** (0.383 g, 94%) as a white solid.

(*R,S*)-**19b**: White solid; Mp: 39-41 °C; $[\alpha]_D^{25}$ = 2.3 (*c* = 0.10, MeOH); ^1H NMR (400 MHz, $CDCl_3$) δ 8.53 (s, 1H), 7.94 (d, *J* = 8.4 Hz, 1H), 7.91 (d, *J* = 8.0 Hz, 1H), 7.51-7.34 (m, 3H), 7.11 (d, *J* = 7.6 Hz, 1H), 5.32 (s, 2H), 4.22-4.13 (m, 2H), 3.91 (dd, *J* = 13.2, 9.2 Hz, 1H), 3.78-3.68 (m, 1H), 3.56 (s, 3H), 3.50-3.35 (m, 1H), 2.77 (s, 1.6H for major isomer), 2.73 (s, 1.4H for minor isomer), 2.03 (s, 3H), 1.46 (s, 4.2H for minor isomer), 1.41 (s, 4.8H for major isomer), 0.96 (s, 9H); ^{13}C NMR (100 MHz, $CDCl_3$) δ 156.2, 156.0, 151.0, 150.7, 135.9, 135.7, 135.5, 133.7, 133.1, 132.6, 132.1, 129.3, 128.8, 127.1, 126.2, 124.8, 124.5, 95.5, 80.3, 80.0, 56.7, 55.8, 51.1, 50.3, 42.2, 34.6, 28.5, 22.5, 22.3; HRMS *m/z* Calcd. for $C_{30}H_{42}N_3O_5S$ (M + H^+): 556.2840; Found: 556.2847.

***Step iii.* Synthesis of compound (*R*)-6b**

To a solution of compound (*R*,*S*)-**19b** (0.363 g, 0.654 mmol) in methanol (19.2 mL) was added HCl (4.0 M in dioxane, 0.493 mL) at 0 °C. After being warmed up to room temperature and stirred at the temperature for 2 h, the reaction mixture was evaporated in vacuo. Without further purification, the residue was dissolved in anhydrous DCM (5 mL) and then triethylamine (Et_3N) (0.410 mL, 2.96 mmol) was added at 0 °C, followed by addition of di-*tert*-butyl dicarbonate (Boc_2O) (0.323 g, 1.48 mmol) in anhydrous DCM (1.0 mL) via a syringe. After being warmed up to room temperature and stirred at the temperature for 2 h, The mixture was concentrated via rotary evaporation to remove most of the organic solvent and purified by column chromatography on silica gel (petroleum ether : ethyl acetate = 1:1) to give compound (*R*)-**6b** (0.330 g, 92%) as a white solid.

(*R*)-**6b**: White solid; Mp: 90-92 °C; $[\alpha]_D^{25}$ = -54.9 (*c* = 0.10, $CHCl_3$); ^{1}H NMR (400 MHz, $CDCl_3$) δ 8.50 (s, 0.75H for major isomer), 8.38 (s, 0.25H for minor isomer), 7.97-7.82 (m, 2H), 7.50-7.31 (m, 3H), 7.11-7.00 (m, 1H), 5.33 (s, 2H), 4.25-3.70 (m, 4H), 3.57 (s, 3H), 3.12 (s, 0.75H for minor isomer), 2.84 (s, 2.25H for major isomer), 2.04 (s, 2.25H for major isomer), 1.98 (s, 0.75H for minor isomer), 1.53-1.41 (m, 9H), 1.30-1.18 (m, 9H); ^{13}C NMR (100 MHz, $CDCl_3$) δ 156.1, 155.0, 150.1, 150.0, 135.6, 133.8, 133.0, 132.3, 129.1, 129.0, 128.5, 127.2, 127.0, 126.0, 124.7, 95.4, 80.1, 56.7, 37.4, 29.8, 28.5, 28.43, 28.37, 22.2; HRMS *m/z* Calcd. for $C_{31}H_{42}N_3O_6$ (M + H^+): 552.3068; Found: 552.3065.

2.2 Synthesis of Compound (*R*)-1b

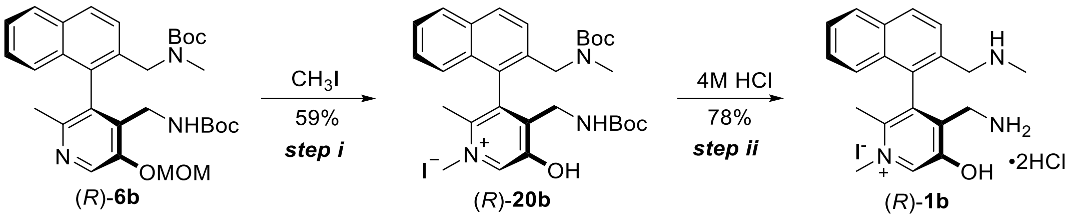

***Step i*. Synthesis of compound (*R*)-20b**[2]

To a stirred solution of (*R*)-**6b** (0.300 g, 0.544 mmol) in THF (5.0 mL) was added MeI (0.387 mL, 2.72 mmol) at room temperature. Then the reaction mixture was stirred at 50 °C for 4 h. Subsequently, another portion of MeI (0.194 mL, 1.36 mmol) was added to the mixture and stirred at 50 °C overnight. Upon the reaction completion, the mixture was concentrated via rotary evaporation under reduced pressure to remove most of the organic solvent and purified by column chromatography on silica gel (ethyl acetate : MeOH = 20:1) to give compound (*R*)-**20b** (0.205 g, 59%) as a light yellow solid.

(*R*)-**20b**: Light yellow solid; Mp: 28-30 °C; $[\alpha]_D^{25}$ = -25.9 (*c* = 0.10, MeOH); ^{1}H NMR (400 MHz, CD_3OD) δ 8.42 (s, 1H), 8.11 (d, *J* = 8.8 Hz, 1H), 8.01 (d, *J* = 8.0 Hz, 1H), 7.61-7.42 (m, 3H), 7.22 (d, *J* = 8.8 Hz, 1H), 4.45-4.10 (m, 5H), 4.05-3.75 (m, 2H), 2.91 (s, 3H), 2.19 (s, 3H), 1.44 (s, 9H), 1.27 (s, 9H); ^{13}C NMR (100 MHz, CD_3OD) δ 158.4, 157.5, 157.1, 145.8, 144.0, 139.5, 135.5, 134.9, 134.4, 132.9, 131.3, 130.9, 129.7, 128.9, 127.7, 126.6, 125.7, 81.7, 81.0, 47.9, 38.7, 28.7, 28.6, 17.4; HRMS *m/z* Calcd. for $C_{30}H_{40}N_3O_5$ (M – I^-): 522.2962; Found: 552.2968.

***Step ii*. Synthesis of pyridoxamine (*R*)-1b**

To a 25 mL round-bottom flask equipped with a magnetic stirrer bar were added (*R*)-**20b** (0.120 g, 0.185 mmol) and HCl (4.0 M in DCM, 3.0 mL) via syringe at room temperature. Upon stirring at 40 °C for 3 h, the mixture was concentrated via rotary evaporation under reduced pressure to remove most of the organic solvent. The residue was vacuum dried to give the *N*-quaternized chiral pyridoxamines (*R*)-**1b** (0.0750 g, 78%) as a pale yellow solid.

(*R*)-**1b**: Pale yellow solid; Mp: 58-60 °C; $[\alpha]_D^{25}$ = -2.1 (*c* = 0.10, MeOH); ^{1}H NMR (400 MHz, CD_3OD) δ 8.64 (s, 1H), 8.29 (d, *J* = 8.8 Hz, 1H), 8.10 (d, *J* = 8.4 Hz, 1H), 7.87 (d, *J* = 8.4 Hz, 1H), 7.68 (t, *J* = 7.6 Hz, 1H), 7.61 (t, *J* = 7.6 Hz, 1H), 7.34 (d, *J* = 7.6 Hz, 1H), 4.41 (s, 3H), 4.22 (d, *J* = 14.8 Hz, 1H), 4.16 (d, *J* = 14.8 Hz, 1H), 3.91 (d, *J* = 14.8 Hz, 1H), 3.68 (d, *J* = 14.8 Hz, 1H), 2.84 (s, 3H), 2.29 (s, 3H); ^{13}C NMR (100 MHz, CD_3OD) δ 155.5, 147.9, 138.8, 138.5, 135.1, 134.8, 132.7, 132.3, 130.5, 130.2,

130.0, 129.0, 126.3, 125.6, 51.1, 38.3, 34.7, 18.3; HRMS *m/z* Calcd. for $C_{20}H_{24}N_3O$ (M – I^- – 2HCl): 322.1914; Found: 322.1910.

Compounds (*S*)-**1a**, (*S*)-**1b**, (*S*)-**1c**, and (*R*)-**1d-f** were prepared by a similar procedure.

Compound (*S*)-1b (Fig. 2)

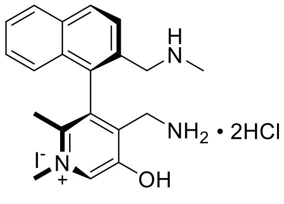

(*S*)-**1b**

Light red solid; Mp: 67-69 °C; $[\alpha]_D^{25}$ = 20.1 (*c* = 0.10, MeOH); ^{1}H NMR (400 MHz, CD_3OD) δ 8.64 (s, 1H), 8.30 (d, *J* = 8.8 Hz, 1H), 8.12 (dd, *J* = 8.4, 1.2 Hz, 1H), 7.89 (d, *J* = 8.8 Hz, 1H), 7.69 (ddd, *J* = 8.4, 6.8, 1.2 Hz, 1H), 7.63 (ddd, *J* = 8.4, 6.8, 1.6 Hz, 1H), 7.36 (dd, *J* = 8.4, 0.8 Hz, 1H), 4.42 (s, 3H), 4.24 (d, *J* = 14.8 Hz, 1H), 4.19 (d, *J* = 14.8 Hz, 1H), 3.93 (d, *J* = 14.8 Hz, 1H), 3.68 (d, *J* = 14.8 Hz, 1H), 2.86 (s, 3H), 2.30 (s, 3H); ^{13}C NMR (100 MHz, CD_3OD) δ 155.4, 148.0, 138.7, 138.6, 135.1, 134.8, 132.7, 132.3, 130.6, 130.5, 130.2, 130.0, 129.1, 126.3, 125.6, 50.9, 48.6, 38.2, 34.6, 18.2. HRMS *m/z* Calcd. for $C_{20}H_{24}N_3O$ (M – I^- – 2HCl): 322.1914; Found: 322.1916.

Compound (*S*)-1a (Fig. 2)

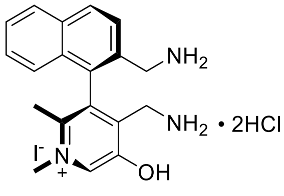

(S)-**1a**

Light red solid; Mp: 40-42 °C; $[\alpha]_D^{25}$ = 37.6 (*c* = 0.10, MeOH); ^{1}H NMR (400 MHz, CD_3OD) δ 8.64 (s, 1H), 8.30 (d, *J* = 8.8 Hz, 1H), 8.11 (dd, *J* = 8.8, 1.6 Hz, 1H), 7.84 (d, *J* = 8.8 Hz, 1H), 7.69 (ddd, *J* = 8.0, 6.8, 1.2 Hz, 1H), 7.62 (ddd, *J* = 8.4, 6.8, 1.2 Hz, 1H), 7.33 (d, *J* = 8.0 Hz, 1H), 4.41 (s, 3H), 4.15 (d, *J* = 14.8 Hz, 1H), 4.06 (d, *J* = 14.8 Hz, 1H), 3.90 (d, *J* = 14.8 Hz, 1H), 3.68 (d, *J* = 14.8 Hz, 1H), 2.29 (s, 3H); ^{13}C NMR

(100 MHz, CD_3OD) δ 155.4, 148.0, 138.8, 138.7, 134.9, 134.8, 132.7, 132.3, 131.8, 130.3, 130.1, 130.0, 129.0, 126.1, 125.8, 48.5, 41.5, 38.1, 18.0; HRMS *m/z* Calcd. for $C_{19}H_{22}N_3O$ (M – I^- – 2HCl): 308.1757; Found: 308.1757.

Compound (*S*)-1c (Fig. 2)

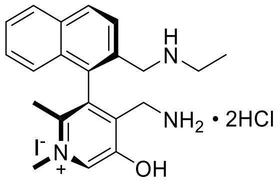

(*S*)-**1c**

Light red solid; Mp: 38-40 °C; $[\alpha]_D^{25}$ = -0.9 (*c* = 0.10, MeOH); ^1H NMR (400 MHz, CD_3OD) δ 8.63 (s, 1H), 8.30 (d, *J* = 8.7 Hz, 1H), 8.11 (d, *J* = 8.4 Hz, 1H), 7.93 (d, *J* = 8.8 Hz, 1H), 7.69 (ddd, *J* = 8.0, 6.8, 1.2 Hz, 1H), 7.62 (ddd, *J* = 8.4, 6.8, 1.4 Hz, 1H), 7.32 (d, *J* = 8.4 Hz, 1H), 4.41 (s, 3H), 4.22 (d, *J* = 14.8 Hz, 1H), 4.15 (d, *J* = 14.8 Hz, 1H), 3.90 (d, *J* = 14.8 Hz, 1H), 3.69 (d, *J* = 14.8 Hz, 1H), 3.26 (q, *J* = 7.2 Hz, 2H), 2.30 (s, 3H), 1.40 (t, *J* = 7.2 Hz, 3H); ^{13}C NMR (100 MHz, CD_3OD) δ 155.4, 148.0, 138.8, 138.6, 135.0, 134.7, 132.7, 132.3, 130.7, 130.4, 130.2, 130.0, 129.0, 126.2, 125.7, 49.5, 48.7, 45.4, 38.2, 18.2, 11.7; HRMS *m/z* Calcd. for $C_{21}H_{26}N_3O$ (M – I^- – 2HCl): 336.2070; Found: 336.2067.

Compound (*R*)-1d (Fig. 2)

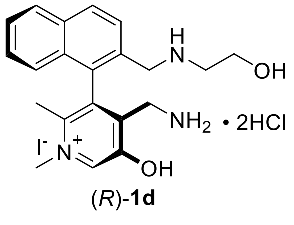

(*R*)-**1d**

Light red oil; $[\alpha]_D^{25}$ = -21.9 (*c* = 0.10, MeOH); ^1H NMR (400 MHz, CD_3OD) δ 8.65 (s, 1H), 8.31 (d, *J* = 8.8 Hz, 1H), 8.12 (d, *J* = 8.0 Hz, 1H), 7.94 (d, *J* = 8.8 Hz, 1H), 7.70 (t, *J* = 7.6 Hz, 1H), 7.63 (t, *J* = 7.6 Hz, 1H), 7.35 (d, *J* = 8.4 Hz, 1H), 4.42 (s, 3H), 4.26 (d, *J* = 14.4 Hz, 1H), 4.20 (d, *J* = 14.4 Hz, 1H), 3.93 (d, *J* = 14.8 Hz, 1H), 3.89 (t, *J* = 4.8 Hz, 2H), 3.69 (d, *J* = 14.8 Hz, 1H), 3.30 (t, *J* = 4.8 Hz, 2H), 2.31 (s, 3H); ^{13}C NMR (100 MHz, CD_3OD) δ 155.4, 148.0, 138.8, 138.6, 134.9, 134.8, 132.7, 132.3, 130.6,

130.5, 130.2, 130.0, 129.1, 126.1, 125.7, 57.8, 51.0, 49.3, 48.5, 38.2, 18.1; HRMS *m/z* Calcd. for $C_{21}H_{26}N_3O_2$ (M – I^- – 2HCl): 352.2020; Found: 352.2012.

Compound (*R*)-1e (Fig. 2)

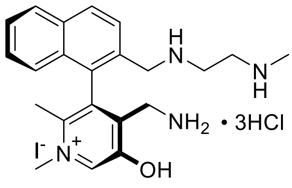

(*R*)-**1e**

Light red solid; Mp: 227-229 °C; $[\alpha]_D^{25}$ = -41.9 (*c* = 0.10, MeOH); ^{1}H NMR (400 MHz, CD_3OD) δ 8.62 (s, 1H), 8.29 (d, *J* = 8.4 Hz, 1H), 8.12-8.07 (m, 2H), 7.71-7.67 (m, 1H), 7.64-7.60 (m, 1H), 7.30 (d, *J* = 8.4 Hz, 1H), 4.41 (s, 3H), 4.34 (d, *J* = 14.8 Hz, 1H), 4.30 (d, *J* = 14.8 Hz, 1H), 3.88 (d, *J* = 14.8 Hz, 1H), 3.78 (d, *J* = 14.8 Hz, 1H), 3.75-3.53 (m, 4H), 2.79 (s, 3H), 2.31 (s, 3H); ^{13}C NMR (100 MHz, CD_3OD) δ 155.4, 148.0, 138.8, 138.4, 134.9, 134.7, 132.6, 132.2, 130.5, 130.2, 130.1, 130.0, 129.0, 126.2, 126.0, 50.1, 48.6, 46.1, 45.6, 38.3, 34.0, 18.3; HRMS *m/z* Calcd. for $C_{22}H_{29}N_4O$ (M – I^- – 3HCl): 365.2336; Found: 365.2337.

Compound (*R*)-1f (Fig. 6b)

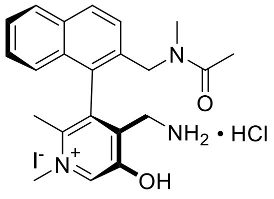

(*R*)-**1f**

Light red solid; Mp: 62-64 °C; $[\alpha]_D^{25}$ = -90.3 (*c* = 0.10, MeOH); ^{1}H NMR (400 MHz, CD_3OD) δ 8.62 (s, 1H), 8.12 (d, *J* = 8.4 Hz, 1H), 8.01 (d, *J* = 8.0 Hz, 1H), 7.63-7.47 (m, 3H), 7.25 (d, *J* = 8.0 Hz, 1H), 4.45 (d, *J* = 14.8 Hz, 1H), 4.38 (s, 3H), 4.15 (d, *J* = 15.6 Hz, 1H), 3.82 (d, *J* = 14.4 Hz, 1H), 3.62 (d, *J* = 14.0 Hz, 1H), 3.27 (s, 3H), 2.27 (s, 3H), 2.11 (s, 3H); ^{13}C NMR (100 MHz, CD_3OD) δ 173.6, 155.4, 148.1, 140.2, 138.5, 136.5, 134.4, 134.3, 132.5, 132.0, 129.9, 129.6, 128.8, 128.0, 125.8, 125.5, 51.6, 48.4, 39.0, 37.9, 21.8, 17.7; HRMS *m/z* Calcd. for $C_{22}H_{26}N_3O_2$ (M – I^- – 2HCl): 364.2025; Found: 364.2025.

3. Preparation of α-Keto Amides 2

3.1 Synthesis of Compounds 2a-l and 2y Using 2e as the Representative Example (Fig. 4)[3]

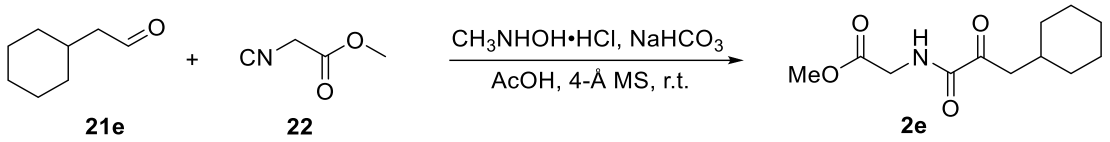

To a 25 mL round-bottom flask equipped with a magnetic stirrer bar were added aldehyde **21e** (1.57 g, 12.5 mmol), *N*-methylhydroxylamine hydrochloride ($CH_3NHOH\cdot HCl$) (1.14 g, 13.7 mmol), $NaHCO_3$ (2.10 g, 25.0 mmol), and 4 Å-MS (2.0 g), successively. The sealed flask was evacuated and refilled with N_2 for three times, followed by addition of anhydrous MeOH (12.5 mL) via a syringe. After the mixture was stirred at room temperature for 0.5 h, methyl isocyanoacetate **22** (1.35 g, 13.7 mmol) and AcOH (6.5 mL, 112.5 mmol) were then added via a syringe. Upon stirring at room temperature for 24 h, the reaction mixture was filtered, and quenched by addition of saturated aqueous $NaHCO_3$ solution till pH 7~8. The mixture was concentrated via rotary evaporation under reduced pressure to remove most of the organic solvent. The residue was diluted with H_2O (20 mL) and extracted with ethyl acetate (20 mL × 3). The combined organic layers were washed with brine, dried over Na_2SO_4, filtered, evaporated in vacuo and purified by column chromatography on silica gel (petroleum ether : ethyl acetate = 5:1) to give compound **2e** (1.59 g, 53%) as a white solid.

2e: White solid; Mp: 61-63 °C; ^{1}H NMR (400 MHz, $CDCl_3$) δ 7.39 (s, 1H), 4.07 (d, J = 5.6 Hz, 2H), 3.78 (s, 3H), 2.78 (d, J = 6.8 Hz, 2H), 1.92-1.83 (m, 1H), 1.72-1.61 (m, 5H), 1.33-1.09 (m, 3H), 1.03-0.93 (m, 2H); ^{13}C NMR (100 MHz, $CDCl_3$) δ 197.9, 169.5, 160.5, 52.7, 44.1, 41.0, 33.7, 33.2, 26.2, 26.1; HRMS *m/z*: Calcd. for $C_{12}H_{20}NO_4$ (M + H^+): 242.1387; Found: 242.1386.

Compounds **2a-d**, **2f-l**, and **2y** were prepared, respectively, in 67%, 60%, 54%, 51%, 52%, 16%, 67%, 62%, 48%, 61%, 58% and 29% yields by a similar procedure.

Compound 2a (Fig. 4)

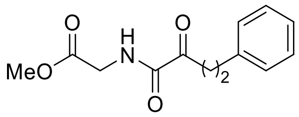

2a

Yellow solid; Mp: 82-84 °C; ^{1}H NMR (400 MHz, $CDCl_3$) δ 7.42 (s, 1H), 7.31-7.28 (m, 2H), 7.22-7.18 (m, 3H), 4.08 (d, *J* = 5.6 Hz, 2H), 3.77 (s, 3H), 3.27 (t, *J* = 7.6 Hz, 2H), 2.95 (t, *J* = 7.6 Hz, 2H); ^{13}C NMR (100 MHz, $CDCl_3$) δ 197.2, 169.3, 160.2, 140.4, 128.6, 128.5, 126.4, 52.6, 41.0, 38.5, 29.2; HRMS *m/z*: Calcd. for $C_{13}H_{16}NO_4$ (M + H^+): 250.1074; Found: 250.1072.

Compound 2b (Fig. 4)

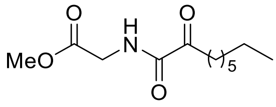

2b

White solid; Mp: 73-75°C; ^{1}H NMR (400 MHz, $CDCl_3$) δ 7.39 (s, 1H), 4.08 (d, *J* =6.0 Hz, 2H), 3.78 (s, 3H), 2.91 (t, *J* = 7.2 Hz, 2H), 1.65-1.57 (m, 2H), 1.33-1.25 (m, 8H), 0.89-0.86 (m, 3H), ^{13}C NMR (100 MHz, $CDCl_3$) δ 198.3, 169.4, 160.4, 52.7, 41.0, 36.9 31.7, 29.1, 23.2, 22.7, 14.2; HRMS *m/z*: Calcd. for $C_{12}H_{22}NO_4$ (M + H^+): 244.1543; Found: 244.1541.

Compound 2c (Fig. 4)

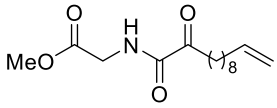

2c

White solid; Mp: 71-73 °C; ^{1}H NMR (400 MHz, $CDCl_3$) δ 7.40 (t, *J* = 6.0 Hz, 1H), 5.85-5.75 (m, 1H), 5.01-4.90 (m, 2H), 4.08 (d, *J* = 5.6 Hz, 2H), 3.77 (s, 3H), 2.90 (t, *J* = 7.2 Hz, 2H), 2.05-1.99 (m, 2H), 1.65-1.55 (m, 2H), 1.37-1.24 (m, 10H); ^{13}C NMR (100 MHz, $CDCl_3$) δ 198.3, 169.4, 160.3, 139.3, 114.3, 52.7, 41.0, 36.9, 33.9, 29.4, 29.14, 29.10, 29.0, 23.2; HRMS *m/z*: Calcd. for $C_{15}H_{26}NO_4$ (M + H^+): 284.1856; Found: 284.1858.

Compound 2d (Fig. 4)

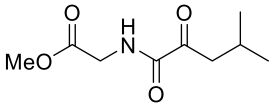

2d

Yellow oil; ^{1}H NMR (400 MHz, $CDCl_3$) δ 7.39 (s, 1H), 4.08 (d, *J* = 5.6 Hz, 2H), 3.78 (s, 3H), 2.80 (d, *J* = 6.8 Hz, 2H), 2.25-2.15 (m, 1H), 0.96 (d, *J* = 6.8 Hz, 6H); ^{13}C NMR (100 MHz, $CDCl_3$) δ 197.9, 169.4, 160.4, 52.6, 45.3, 41.0, 24.4, 22.6; HRMS *m/z*: Calcd. for $C_9H_{16}NO_4$ (M + H^+): 202.1074; Found: 202.1072.

Compound 2f (Fig. 4)

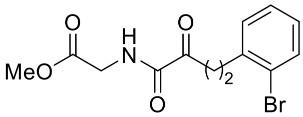

2f

Yellow solid; Mp: 76-78 °C; ^{1}H NMR (600 MHz, $CDCl_3$) δ 7.53 (dd, *J* = 8.4, 1.2 Hz, 1H), 7.45 (t, *J* = 6.0 Hz, 1H), 7.28-7.20 (m, 2H), 7.07 (td, *J* = 7.6, 1.2 Hz, 1H), 4.08 (d, *J* = 6.0 Hz, 2H), 3.77 (s, 3H), 3.29 (t, *J* = 7.8 Hz, 2H), 3.05 (t, *J* = 7.8 Hz, 2H); ^{13}C NMR (100 MHz, $CDCl_3$) δ 196.9, 169.4, 160.1, 139.6, 133.0, 130.6, 128.2, 127.7, 124.4, 52.7, 41.0, 37.0, 29.7; HRMS *m/z*: Calcd. for $C_{13}H_{15}BrNO_4$ (M + H^+): 328.0179; Found: 328.0175.

Compound 2g (Fig. 4)

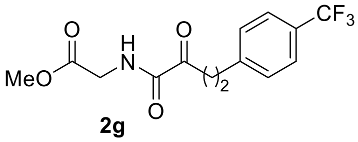

2g

Yellow solid; Mp: 77-79 °C; ^{1}H NMR (400 MHz, $CDCl_3$) δ 7.53 (d, *J* = 8.0 Hz, 2H), 7.41 (brs, 1H), 7.32 (d, *J* = 8.0 Hz, 2H), 4.08 (d, *J* = 5.6 Hz, 2H), 3.78 (s, 3H), 3.29 (t, *J* = 7.4 Hz, 2H), 3.00 (t, *J* = 7.4 Hz, 2H); ^{13}C NMR (100 MHz, $CDCl_3$) δ 196.8, 169.4, 160.0, 144.5, 128.9, 128.7 (q, *J* = 32.3 Hz) 125.5 (q, *J* = 3.6 Hz), 124.3 (q, *J* = 270.3 Hz), 52.7, 41.0, 38.0, 28.8; HRMS *m/z*: Calcd. for $C_{14}H_{15}F_3NO_4$ (M + H^+): 318.0948; Found: 318.0948.

Compound 2h (Fig. 4)

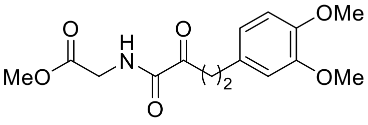

2h

Yellow solid; Mp: 116-118 °C; ^{1}H NMR (400 MHz, CDCl$_3$) δ 7.39 (s, 1H), 6.79-6.73 (m, 3H), 4.08 (d, *J* = 5.6 Hz, 2H), 3.86 (s, 3H), 3.85 (s, 3H), 3.78 (s, 3H), 3.25 (t, *J* = 7.6 Hz, 2H), 2.90 (t, *J* = 7.6 Hz, 2H); ^{13}C NMR (150 MHz, CDCl$_3$) δ 197.3, 169.3, 160.2, 149.0, 147.6, 133.0, 120.3, 111.8, 111.4, 56.03, 55.95, 52.7, 41.0, 38.7, 28.9; HRMS *m/z*: Calcd. for $C_{15}H_{20}NO_6$ (M + H$^+$): 310.1285; Found: 310.1279.

Compound 2i (Fig. 4)

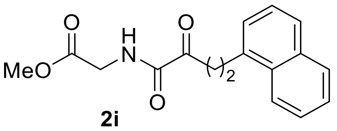

2i

Yellow solid; Mp: 135-137 °C; ^{1}H NMR (400 MHz, CDCl$_3$) δ 8.04 (d, *J* = 8.4 Hz, 1H), 7.86 (d, *J* = 8.4 Hz, 1H), 7.74 (d, *J* = 8.0 Hz, 1H), 7.56-7.47 (m, 2H), 7.43-7.35 (m, 3H), 4.10 (d, *J* = 5.6 Hz, 2H), 3.79 (s, 3H), 3.45-3.36 (m, 4H); ^{13}C NMR (100 MHz, CDCl$_3$) δ 197.3, 169.4, 160.2, 136.4, 134.0, 131.7, 129.0, 127.3, 126.3, 126.2, 125.8, 125.7, 123.5, 52.7, 41.1, 38.0, 26.5; HRMS *m/z*: Calcd. for $C_{17}H_{18}NO_4$ (M + H$^+$): 300.1230; Found: 300.1229.

Compound 2j (Fig. 4)

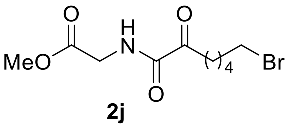

2j

Colorless oil; ^{1}H NMR (400 MHz, CDCl$_3$) δ 7.42 (s, 1H), 4.06 (d, *J* = 5.6 Hz, 2H), 3.76 (s, 3H), 3.39 (t, *J* = 6.6 Hz, 2H), 2.92 (t, *J* = 7.2 Hz, 2H), 1.91-1.82 (m, 2H), 1.68-1.58 (m, 2H), 1.50-1.43 (m, 2H); ^{13}C NMR (100 MHz, CDCl$_3$) δ 197.9, 169.4, 160.3, 52.7, 41.0, 36.6, 33.5, 32.5, 27.6, 22.4; HRMS *m/z*: Calcd. for $C_{10}H_{17}BrNO_4$ (M + H$^+$): 294.0335; Found: 294.0334.

Compound 2k (Fig. 4)

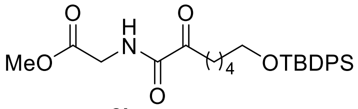

2k

Yellow oil; ^1H NMR (600 MHz, $CDCl_3$) δ 7.66 (dt, *J* = 7.2, 1.2 Hz, 4H), 7.43-7.36 (m, 7H), 4.08 (d, *J* = 5.4 Hz, 2H), 3.78 (s, 3H), 3.66 (t, *J* = 6.0 Hz, 2H), 2.90 (t, *J* = 7.2 Hz, 2H), 1.63-1.55 (m, 4H), 1.44-1.38 (m, 2H), 1.04 (s, 9H); ^{13}C NMR (100 MHz, $CDCl_3$) δ 198.1, 169.4, 160.3, 135.7, 134.1, 129.6, 127.7, 63.7, 52.7, 41.0, 36.9, 32.3, 27.0, 25.4, 23.0, 19.3; HRMS *m/z*: Calcd. for $C_{26}H_{36}NO_5Si$ (M + H^+): 470.2357; Found: 470.2358.

Compound 2l (Fig. 4)

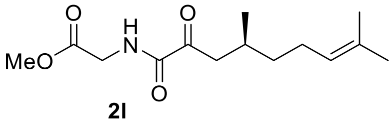

2l

Colorless oil; ^1H NMR (400 MHz, $CDCl_3$) δ 7.40 (s, 1H), 5.10-5.05 (m, 1H), 4.08 (d, *J* = 5.6 Hz, 2H), 3.78 (s, 3H), 2.89 (dd, *J* = 17.2, 5.6 Hz, 1H), 2.75 (dd, *J* = 17.2, 8.0 Hz, 1H), 2.10-1.92 (m, 3H), 1.67 (s, 3H), 1.59 (s, 3H), 1.39-1.20 (m, 2H), 0.92 (d, *J* = 6.8 Hz, 3H); ^{13}C NMR (100 MHz, $CDCl_3$) δ 198.0, 169.4, 160.4, 131.7, 124.2, 52.6, 43.7, 41.0, 37.0, 28.7, 25.8, 25.5, 19.8, 17.7; HRMS *m/z*: Calcd. for $C_{14}H_{24}NO_4$ (M + H^+): 270.1700; Found: 270.1702.

Compound 2y (Fig. 4)

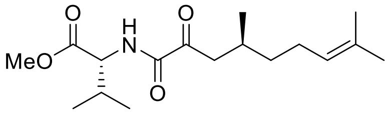

2y

Light yellow oil; $[\alpha]_D^{25}$ = -25.5 (*c* = 0.10, $CHCl_3$); ^1H NMR (400 MHz, $CDCl_3$) δ 7.37 (d, *J* = 9.2 Hz, 1H), 5.06 (t, *J* = 7.2 Hz, 1H), 4.46 (dd, *J* = 9.2, 4.8 Hz, 1H), 3.74 (s, 3H), 2.89 (dd, *J* = 17.2, 5.6 Hz, 1H), 2.73 (dd, *J* = 17.2, 8.0 Hz, 1H), 2.27-2.15 (m, 1H), 2.10-1.91 (m, 3H), 1.66 (s, 3H), 1.57 (s, 3H), 1.38-1.16 (m, 2H), 0.96-0.85 (m, 9H); ^{13}C NMR (100 MHz, $CDCl_3$) δ 198.4, 171.5, 160.2, 131.7, 124.3, 57.3, 52.4, 43.8, 37.1,

31.5, 28.8, 25.8, 25.5, 19.8, 19.1, 17.81, 17.76; HRMS *m/z*: Calcd. for $C_{17}H_{30}NO_4$ (M + H^+): 312.2169; Found: 312.2169.

3.2 Synthesis of Compounds 2m-r, 2v-2x and 2z-aa with Compound 2m as the Representative Example (Fig. 4)[4]

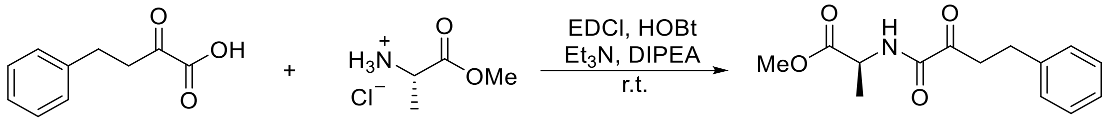

9a **23m** **2m**

To a 50 mL round-bottom flask were successively added compound **9a** (0.600 g, 3.37 mmol), (*S*)-alanine methyl ester hydrochloride **23m** (0.517 g, 3.71 mmol), 1-(3-dimethylaminopropyl)-3-ethylcarbodiimide hydrochloride (EDCl) (1.42 g, 7.41 mmol), 1-hydroxybenzotriazole (HOBt) (1.00 g, 7.41 mmol), dry DMF (20.0 mL), Et_3N (2.30 mL, 11.1 mmol), and *N*,*N*-diisopropylethylamine (DIPEA) (1.46 mL, 7.41 mmol). The sealed flask was evacuated and refilled with N_2 three time. After stirring at room temperature overnight, the reaction was quenched by addition of H_2O (60 mL), and extracted with ethyl acetate (20 mL × 3). The combined organic layers were washed with brine, dried over Na_2SO_4, filtered, concentrated and purified by column chromatography on silica gel (petroleum ether : ethyl acetate = 5:1) to give compound **2m** (0.450 g, 52%) as a yellow oil.

2m: Yellow oil; $[\alpha]_D^{25}$ = -22.8 (*c* = 0.10, $CHCl_3$); ^1H NMR (600 MHz, $CDCl_3$) δ 7.42 (d, *J* = 8.4 Hz, 1H), 7.30-7.26 (m, 2H), 7.22-7.18 (m, 3H), 4.58-4.53 (m, 1H), 3.76 (s, 2.3H for major isomer), 3.75 (s, 0.7H for minor isomer), 3.31-3.22 (m, 2H), 2.94 (t, *J* = 7.2 Hz, 2H), 1.45 (d, *J* = 7.2 Hz, 2.3H for major isomer), 1.41 (d, *J* = 7.2 Hz, 0.7H for minor isomer); ^{13}C NMR (150 MHz, $CDCl_3$) δ 197.5, 172.4, 159.5, 140.4, 128.6, 128.5, 126.4, 52.8, 48.1, 38.4, 29.2, 18.2; HRMS *m/z*: Calcd. for $C_{14}H_{18}NO_4$ (M + H^+): 264.1230; Found: 264.1230.

Compounds **2n-r, 2v-2x** and **2z-aa** were prepared, respectively, in 59%, 59%, 70%, 49%, 51%, 26%, 47%, 21%, 57%, and 44% yields by a similar procedure.

Compound 2n (Fig. 4)

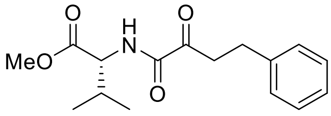

2n

White solid; Mp: 61-63 °C; $[\alpha]_D^{25}$ = -24.3 (*c* = 0.10, $CHCl_3$); ^1H NMR (400 MHz, $CDCl_3$) δ 7.37 (d, *J* = 8.8 Hz, 1H), 7.30-7.26 (m, 2H), 7.22-7.18 (m, 3H), 4.48 (dd, *J* = 9.2, 5.2 Hz, 1H), 3.75 (s, 3H), 3.34-3.20 (m, 2H), 2.95 (t, *J* = 7.6 Hz, 2H), 2.28-2.17 (m, 1H), 0.94 (d, *J* = 6.8 Hz, 3H), 0.93 (d, *J* = 6.8 Hz, 3H); ^{13}C NMR (150 MHz, $CDCl_3$) δ 197.6, 171.4, 159.9, 140.4, 128.6, 128.5, 126.4, 57.4, 52.4, 38.5, 31.4, 29.2, 19.1, 17.8; HRMS *m/z*: Calcd. for $C_{16}H_{22}NO_4$ (M + H^+): 292.1543; Found: 292.1546.

Compound 2o (Fig. 4)

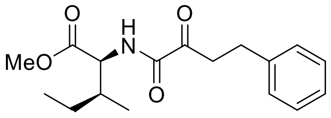

2o

Yellow oil; $[\alpha]_D^{25}$ = -9.6 (*c* = 0.10, $CHCl_3$); ^1H NMR (400 MHz, $CDCl_3$) δ 7.31 (d, *J* = 8.8 Hz, 1H), 7.23-7.19 (m, 2H), 7.14-7.10 (m, 3H), 4.44 (dd, *J* = 9.2, 5.2 Hz, 1H), 3.67 (s, 3H), 3.26-3.12 (m, 2H), 2.87 (t, *J* = 7.2 Hz, 2H), 1.92-1.82 (m, 1H), 1.42-1.32 (m, 1H), 1.18-1.07 (m, 1H), 0.87-0.83 (m, 6H); ^{13}C NMR (100 MHz, $CDCl_3$) δ 197.6, 171.4, 159.7, 140.4, 128.6, 128.5, 126.4, 56.7, 52.4, 38.5, 38.0, 29.2, 25.2, 15.6, 11.6； HRMS *m/z*: Calcd. for $C_{17}H_{24}NO_4$ (M + H^+): 306.1700; Found: 306.1700.

Compound 2p (Fig. 4)

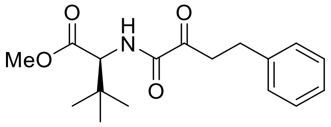

2p

Yellow oil; $[\alpha]_D^{25}$ = -34.3 (*c* = 0.10, $CHCl_3$); ^1H NMR (400 MHz, $CDCl_3$) δ 7.36 (d, *J* = 10.0 Hz, 1H), 7.24-7.19 (m, 2H), 7.15-7.11 (m, 3H), 4.31 (d, *J* = 10.0 Hz, 1H), 3.67 (s, 3H), 3.27-3.13 (m, 2H), 2.87 (t, *J* = 7.6 Hz, 2H), 0.92 (s, 9H); ^{13}C NMR (100 MHz,

$CDCl_3$) δ 197.6, 171.0, 159.6, 140.4, 128.6, 128.5, 126.4, 60.3, 52.1, 38.6, 35.1, 29.2, 26.6; HRMS *m/z*: Calcd. for $C_{17}H_{24}NO_4$ (M + H^+): 306.1700; Found: 306.1700.

Compound 2q (Fig. 4)

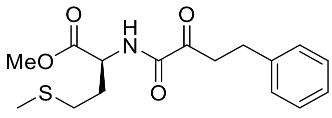

2q

Yellow solid; Mp: 41-43 °C; $[\alpha]_D^{25}$ = 5.9 (*c* = 0.10, $CHCl_3$); ^1H NMR (400 MHz, $CDCl_3$) δ 7.52 (d, *J* = 8.4 Hz, 1H), 7.30-7.26 (m, 2H), 7.21-7.17 (m, 3H), 4.69 (ddd, *J* = 8.4, 7.2, 4.8 Hz, 1H), 3.77 (s, 3H), 3.34-3.20 (m, 2H), 2.95 (t, *J* = 7.2 Hz, 2H), 2.50 (t, *J* = 7.2 Hz, 2H), 2.26-2.15 (m, 1H), 2.12-2.00 (m, 4H); ^{13}C NMR (150 MHz, $CDCl_3$) δ 197.4, 171.4, 159.8, 140.3, 128.6, 128.5, 126.4, 52.8, 51.5, 38.4, 31.5, 30.0, 29.2, 15.5; HRMS *m/z*: Calcd. for $C_{16}H_{22}NO_4S$ (M + H^+): 324.1264; Found: 324.1264.

Compound 2r (Fig. 4)

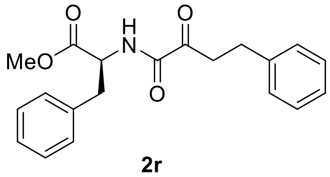

2r

Yellow oil; $[\alpha]_D^{25}$ = 3.1 (*c* = 0.10, $CHCl_3$); ^1H NMR (400 MHz, $CDCl_3$) δ 7.34 (d, *J* = 8.4 Hz, 1H), 7.31-7.23 (m, 5H), 7.22-7.17 (m, 3H), 7.11-7.06 (m, 2H), 4.81 (ddd, *J* = 8.4, 6.4, 5.6 Hz, 1H), 3.72 (s, 3H), 3.27-3.17 (m, 2H), 3.16-3.08 (m, 2H), 2.91 (t, *J* = 7.6 Hz, 2H); ^{13}C NMR (100 MHz, $CDCl_3$) δ 197.3, 171.0, 159.5, 140.3, 135.4, 129.2, 128.8, 128.6, 128.4, 127.4, 126.4, 53.3, 52.6, 38.4, 38.0, 29.1; HRMS *m/z*: Calcd. for $C_{20}H_{22}NO_4$ (M + H^+): 340.1543; Found: 340.1544.

Compound 2v (Fig. 4)

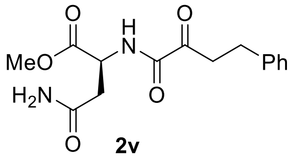

2v

Yellow solid; Mp: 167-169 °C; $[\alpha]_D^{25}$ = 8.3 (c = 0.10, $CHCl_3$); ^1H NMR (400 MHz, $CDCl_3$) δ 8.03 (d, J = 9.2 Hz, 1H), 7.33-7.26 (m, 2H), 7.22-7.15 (m, 3H), 5.66 (s, 2H), 4.82-4.70 (m, 1H), 3.76 (s, 3H), 3.25 (t, J = 7.6 Hz, 2H), 3.01 (dd, J = 16.4, 4.8 Hz, 1H), 2.94 (t, J = 7.4 Hz, 2H), 2.74 (dd, J = 16.4, 4.4 Hz, 1H); ^{13}C NMR (100 MHz, $CDCl_3$) δ 197.1, 171.8, 170.7, 159.9, 140.5, 128.6, 128.5, 126.4, 53.1, 48.8, 38.6, 36.6, 29.2; HRMS m/z: Calcd. for $C_{15}H_{19}N_2O_5$ (M + H^+): 307.1288; Found: 307.1287.

Compound 2w (Fig. 4)

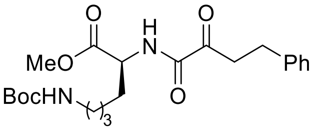

2w

Yellow oil; $[\alpha]_D^{25}$ = -8.6 (c = 0.10, $CHCl_3$); ^1H NMR (400 MHz, $CDCl_3$) δ 7.39 (d, J = 8.8 Hz, 1H), 7.32-7.24 (m, 2H), 7.23-7.16 (m, 3H), 4.65-4.45 (m, 2H), 3.75 (s, 3H), 3.26 (t, J = 7.6 Hz, 2H), 3.15-3.0 (m, 2H), 2.94 (t, J = 7.6 Hz, 2H), 1.96-1.82 (m, 1H), 1.81-1.66 (m, 1H), 1.55-1.45 (m, 2H), 1.42 (s, 9H), 1.37-1.27 (m, 2H); ^{13}C NMR (100 MHz, $CDCl_3$) δ 197.4, 171.8, 159.7, 156.1, 140.4, 128.6, 128.5, 126.4, 79.3, 52.7, 52.1, 40.2, 38.5, 32.1, 29.6, 29.2, 28.5, 22.6; HRMS m/z: Calcd. for $C_{22}H_{32}N_2O_6Na^+$ (M + Na $^+$): 443.2153; Found: 443.2154.

Compound 2x (Fig. 4)

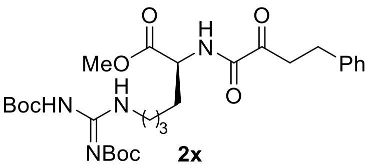

2x

Yellow oil; $[\alpha]_D^{25}$ = -14.4 (c = 0.10, $CHCl_3$); ^1H NMR (400 MHz, $CDCl_3$) δ 11.49 (s, 1H), 8.30 (t, J = 5.2 Hz, 1H), 7.37 (d, J = 8.4 Hz, 1H), 7.32-7.26 (m, 2H), 7.26-7.15 (m, 3H), 4.58-4.48 (m, 1H), 3.76 (s, 3H), 3.43-3.35 (m, 2H), 3.26 (t, J = 7.6 Hz, 2H), 2.94 (t, J = 7.6 Hz, 2H), 1.96-1.84 (m, 1H), 1.82-1.70 (m, 1H), 1.63-1.52 (m, 2H), 1.49 (s, 9H), 1.48 (s, 9H), 1.42-1.30 (m, 2H); ^{13}C NMR (100 MHz, $CDCl_3$) δ 197.4, 171.8, 163.7, 159.7, 156.3, 153. 4, 140.4, 128.6, 128.5, 126.4, 83.3, 79.4, 52.8, 52.1, 40.5, 38.5,

32.0, 29.2, 28.6, 28.4, 28.2, 22.7; HRMS *m/z*: Calcd. for $C_{28}H_{43}N_4O_8$ (M + H^+): 563.3075; Found: 563.3077.

Compound 2z (Fig. 4)

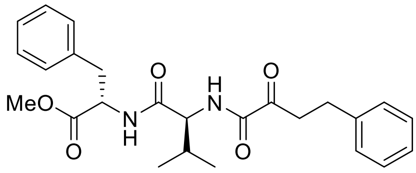

2z

Yellow solid; Mp: 123-125 °C; $[\alpha]_D^{25}$ = 30.0 (*c* = 0.10, $CHCl_3$); ^{1}H NMR (600 MHz, $CDCl_3$) δ 7.41 (d, *J* = 9.0 Hz, 1H), 7.30-7.17 (m, 8H), 7.10-7.09 (m, 0.5H for minor isomer), 7.06-7.04 (m, 1.5H for major isomer), 6.39 (d, *J* = 8.4 Hz, 1H), 4.91-4.86 (m, 1H), 4.22-4.18 (m, 1H), 3.70 (s, 3H), 3.30-3.02 (m, 4H), 2.95-2.91 (m, 2H), 2.16-2.04 (m, 1H), 0.92 (d, *J* = 6.6 Hz, 2.25H for major isomer), 0.89 (d, *J* = 6.6 Hz, 2.25H for major isomer), 0.83 (d, *J* = 7.2 Hz, 0.75H for minor isomer), 0.80 (d, *J* = 6.6 Hz, 0.75H for minor isomer); ^{13}C NMR (100 MHz, $CDCl_3$) δ 197.3, 171.7, 169.9, 159.9, 140.5, 135.6, 129.3, 128.7, 128.6, 128.5, 127.3, 126.4, 58.4, 53.1, 52.5, 38.6, 37.9, 31.3, 29.2, 19.2, 18.1; HRMS *m/z*: Calcd. for $C_{25}H_{31}N_2O_5$ (M + H^+): 439.2227; Found: 439.2226.

Compound 2aa (Fig. 4)

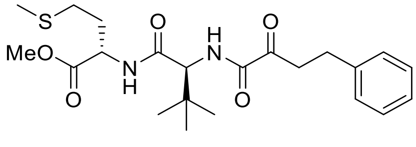

2aa

Yellow oil; $[\alpha]_D^{25}$ = -11.5 (*c* = 0.10, $CHCl_3$); ^{1}H NMR (400 MHz, $CDCl_3$) δ 7.52 (d, *J* = 9.6 Hz, 1H), 7.23-7.10 (m, 5H), 6.76 (d, *J* = 7.6 Hz, 0.15H for minor isomer), 6.63 (d, *J* = 7.6 Hz, 0.85H for major isomer), 4.67-4.57 (m, 1H), 4.21 (d, *J* = 9.6 Hz, 0.15H for minor isomer), 4.14 (d, *J* = 9.6 Hz, 0.85H for major isomer), 3.68 (s, 3H), 3.27-3.11 (m, 2H), 2.87 (t, *J* = 7.2 Hz, 2H), 2.42 (t, *J* = 7.2 Hz, 2H), 2.10-1.87 (m, 5H), 0.95 (s, 9H); ^{13}C NMR (150 MHz, $CDCl_3$) δ 197.4, 172.1, 169.4, 159.9, 140.5, 128.6, 128.5,

126.4, 61.0, 52.7, 51.7, 38.7, 35.2, 31.3, 30.1, 29.2, 26.7, 15.6; HRMS *m/z*: Calcd. for $C_{22}H_{33}N_2O_5S$ (M + H^+): 437.2105; Found: 437.2108.

3.3 Synthesis of Compounds 2s-u and 2ac with Compound 2s as the Representative Example (Fig. 4)[5]

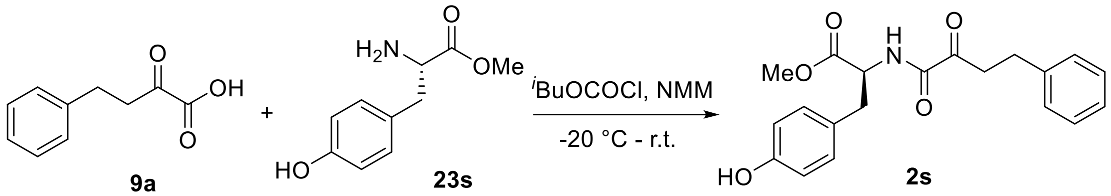

To a 50 mL round-bottom flask were added compound **9a** (0.700 g, 3.93 mmol) and anhydrous THF (5 mL). To the reaction mixture were added isobutyl chloroformate (^{i}BuOCOCl) (0.233 g, 3.93 mmol) and *N*-methylmorpholine (NMM) (0.172 g, 3.93 mmol) via a syringe at -20 °C under N_2 atmosphere. Upon stirring at -20 °C for 20 min, a solution of methyl (*S*)-tyrosinate **23s** (0.766 g, 3.93 mmol) in anhydrous DMF (10 mL) was added via a syringe at the same temperature. The resulting mixture was allowed to be warmed up to room temperature and stirred at the temperature for 3 h. Then the reaction mixture was evaporated in vacuo, and quenched by addition of H_2O (50 mL). The resulting mixture was extracted with ethyl acetate (30 mL × 3). The combined organic layers were washed with brine, dried over Na_2SO_4, filtered, concentrated and purified by column chromatography on silica gel (petroleum ether : ethyl acetate = 3:1) to give compound **2s** (0.650 g, 47%) as a yellow oil.

2s: Yellow oil; $[\alpha]_D^{25}$ = 32.5 (*c* = 0.10, $CHCl_3$); ^{1}H NMR (600 MHz, $CDCl_3$) δ 7.39 (d, *J* = 8.4 Hz, 1H), 7.29-7.25 (m, 2H), 7.21-7.16 (m, 3H), 6.97-6.93 (m, 2H), 6.74-6.71 (m, 2H), 4.79-4.75 (m, 1H), 3.72 (s, 3H), 3.27-3.16 (m, 2H), 3.11-3.01 (m, 2H), 2.91 (t, *J* = 7.8 Hz, 2H); ^{13}C NMR (150 MHz, $CDCl_3$) δ 197.3, 171.3, 159.6, 155.5, 140.3, 130.3, 128.6, 128.4, 126.8, 126.4, 115.8, 53.5, 52.7, 38.4, 37.2, 29.1; HRMS *m/z*: Calcd. for $C_{20}H_{22}NO_5$ (M + H^+): 356.1492; Found: 356.1494.

The compounds **2t-u** and **2ac** were prepared, respectively, in 46%, 89% and 72%

yields by a similar procedure.

Compound 2t (Fig. 4)

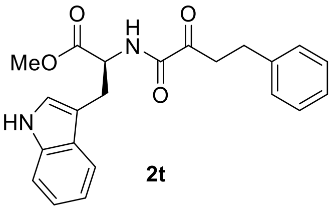

2t

Light yellow solid; Mp: 41-43 °C; $[\alpha]_D^{25}$ = 17.5 (*c* = 0.10, $CHCl_3$); ^{1}H NMR (600 MHz, $CDCl_3$) δ 8.27 (s, 1H), 7.53 (d, *J* = 7.8 Hz, 1H), 7.46 (d, *J* = 8.4 Hz, 1H), 7.34-7.26 (m, 3H), 7.23-7.18 (m, 4H), 7.15-7.11 (m, 1H), 6.96 (d, *J* = 2.4 Hz, 1H), 4.91-4.86 (m, 1H), 3.70 (s, 3H), 3.39-3.31 (m, 2H), 3.27-3.17 (m, 2H), 2.90 (t, *J* = 7.8 Hz, 2H); ^{13}C NMR (150 MHz, $CDCl_3$) δ 197.4, 171.5, 159.7, 140.4, 136.2, 128.6, 128.4, 127.3, 126.3, 123.0, 122.4, 119.8, 118.5, 111.5, 109.5, 52.9, 52.6, 38.4, 29.1, 27.8; HRMS *m/z*: Calcd. for $C_{22}H_{23}N_2O_4$ (M + H^+): 379.1652; Found: 379.1659.

Compound 2u (Fig. 4)

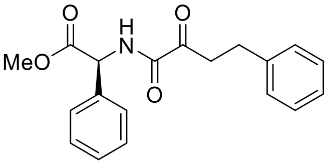

2u

Yellow solid; Mp: 67-69 °C; $[\alpha]_D^{25}$ = 64.1 (*c* = 0.10, $CHCl_3$); ^{1}H NMR (600 MHz, $CDCl_3$) δ 7.85 (d, *J* = 7.8 Hz, 1H), 7.38-7.30 (m, 5H), 7.28-7.24 (m, 2H), 7.20-7.16 (m, 3H), 5.50 (d, *J* = 7.2 Hz, 1H), 3.73 (s, 3H), 3.30-3.19 (m, 2H), 2.97-2.88 (m, 2H); ^{13}C NMR (150 MHz, $CDCl_3$) δ 197.3, 170.4, 159.3, 140.4, 135.6, 129.2, 129.0, 128.6, 128.5, 127.4, 126.4, 56.5, 53.1, 38.5, 29.2; HRMS *m/z*: Calcd. for $C_{19}H_{20}NO_4$ (M + H^+): 326.1387; Found: 326.1387.

Compound 2ac (Fig. 4)

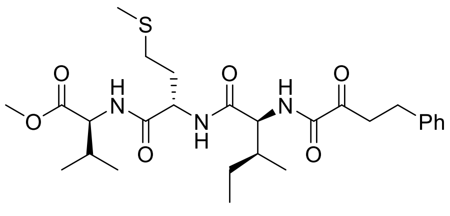

2ac

Yellow solid; Mp: 155-157 °C; $[\alpha]_D^{25}$ = -33.9 (c = 0.10, $CHCl_3$); ^1H NMR (400 MHz, $CDCl_3$) δ 7.62-7.54 (m, 1H), 7.24-6.90 (m, 7H), 4.73-4.63 (m, 1H), 4.47 (dd, J = 8.8, 4.8 Hz, 1H), 4.25 (t, J = 8.4 Hz, 1H), 3.66 (s, 3H), 3.28-3.12 (m, 2H), 2.85 (t, J = 7.6 Hz, 2H), 2.54-2.42 (m, 2H), 2.16-1.81 (m, 7H), 1.47-1.36 (m, 1H), 1.09-0.96 (m, 1H), 0.90-0.75 (m, 12H); ^{13}C NMR (100 MHz, $CDCl_3$) δ 197.4, 172.1, 170.9, 170.4, 160.1, 140.5, 128.6, 128.5, 126.3, 57.9, 57.4, 52.3, 52.1, 38.7, 37.4, 31.3, 31.0, 29.9, 29.1, 25.0, 19.0, 17.8, 15.5, 15.0, 11.3; HRMS m/z: Calcd. for $C_{27}H_{42}N_3O_6S$ (M + H^+): 536.2789; Found: 536.2798.

3.4 Synthesis of Compounds 2ab (Fig. 4)

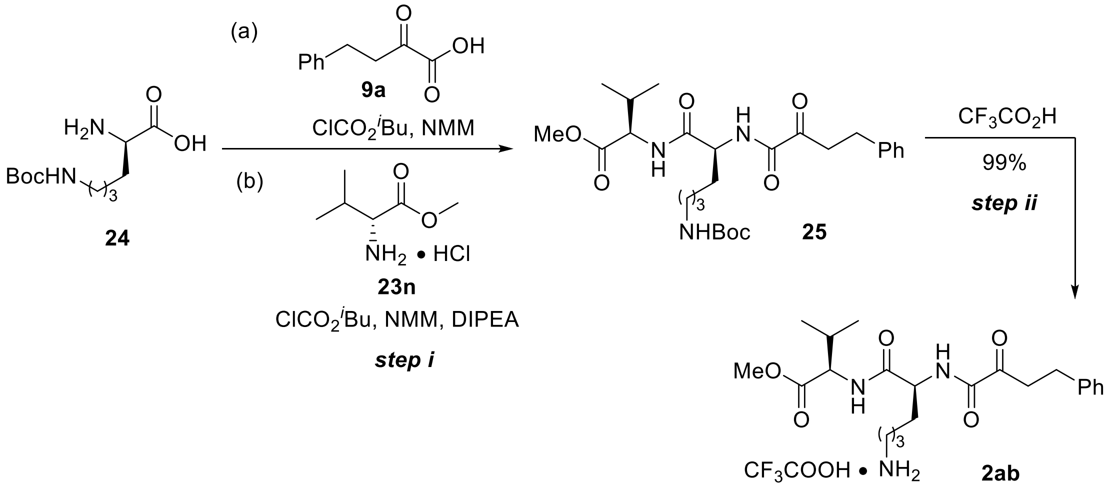

Step i. Synthesis of compound 25[5]

To a 100 mL round-bottom flask were added compound **9a** (2.00 g, 11.2 mmol), anhydrous THF (50 mL). To the mixture were added isobutyl chloroformate (^{i}BuOCOCl) (1.69 g, 12.3 mmol) and N-methylmorpholine (NMM) (1.25 g, 12.3 mmol) via a syringe at -20 °C under N_2 atmosphere. Upon stirring at -20 °C for 20 min, a solution of compound **24** (3.32 g, 13.4 mmol) in anhydrous DMF (10 mL) was added via a syringe at the temperature. The resulting mixture was allowed to warm up to room

temperature and stirred at the temperature overnight. The reaction mixture was filtered to remove unreacted reactant **24**. To the filtrate was added H_2O (50 mL). The resulting mixture was extracted with ethyl acetate (50 mL × 3). The combined organic layers were washed with brine, dried over Na_2SO_4, filtered, concentrated and purified by column chromatography on silica gel (ethyl acetate : acetic acid = 100:1) to give condensation product (0.900 g) as a yellow oil. The reaction was repeated for two times.

The above obtained product (1.80 g) was dissolved in anhydrous THF (25 mL). To the solution were added isobutyl chloroformate (^{i}BuOCOCl) (0.667 g, 4.87 mmol) and *N*-methylmorpholine (NMM) (0.493 g, 4.87 mmol) via a syringe at -20 °C. Upon stirring at -20 °C for 20 min, a mixture of compound **23n** (0.748 g, 4.40 mmol) and *N*,*N*-diisopropylethylamine (DIPEA) in anhydrous DCM (8.0 mL) was added via a syringe at -20 °C. The resulting mixture was allowed to warm up to room temperature and stirred at the temperature overnight. To the reaction mixture was added H_2O (30 mL). The resulting mixture was extracted with DCM (30 mL × 3). The combined organic layers were washed with brine, dried over Na_2SO_4, filtered, concentrated and purified by column chromatography on silica gel (petroleum ether : ethyl acetate = 1:1) to give product **25** (1.00 g, 9% for two steps) as a yellow oil.

25: Light yellow oil; $[\alpha]_D^{25}$ = -20.1 (*c* = 0.10, $CHCl_3$); ^{1}H NMR (400 MHz, $CDCl_3$) δ 7.51 (d, *J* = 8.4 Hz, 1H), 7.30-7.22 (m, 2H), 7.22-7.15 (m, 3H), 6.73 (d, *J* = 8.8 Hz, 1H), 4.62 (t, *J* = 6.0 Hz, 1H), 4.55-4.40 (m, 2H), 3.70 (s, 3H), 3.25 (t, *J* = 7.6 Hz, 2H), 3.17-3.00 (m, 2H), 2.93 (t, *J* = 7.6 Hz, 2H), 2.22-2.10 (m, 1H), 2.00-1.82 (m, 1H), 1.77-1.63 (m, 1H), 1.55-1.43 (m, 2H), 1.42 (s, 9H), 1.40-1.30 (m, 2H), 0.93 (d, *J* = 6.8, 3H), 0.89 (d, *J* = 6.8, 3H); ^{13}C NMR (100 MHz, $CDCl_3$) δ 197.3, 172.2, 170.7, 160.1, 156.2, 140.4, 128.6, 128.5, 126.4, 79.3, 57.3, 53.1, 52.4, 40.0, 38.6, 32.3, 31.2, 29.6, 29.1, 28.5, 22.7, 19.2, 17.9; HRMS *m/z*: Calcd. for $C_{27}H_{42}N_3O_7$ (M + H^+): 520.3017; Found: 520.3019.

***Step ii*. Synthesis of compound 2ab**

To a 50 mL round-bottom flask were added compound **25** (1.00 g, 1.90 mmol), trifluoroacetic acid (CF_3CO_2H) (10.0 mL) and DCM (5.0 mL). The reaction mixture

was stirred at room temperature overnight and then evaporated in vacuo to give product **2ab** (1.03 g, 99%) as a yellow oil.

2ab: Yellow oil; $[\alpha]_D^{25}$ = -8.9 (c = 0.10, $CHCl_3$); ^{1}H NMR (400 MHz, $CDCl_3$) δ 7.82 (d, J = 8.4 Hz, 1H), 7.63 (s, 2H), 7.51 (d, J = 8.8 Hz, 1H), 7.43-7.22 (m, 2H), 7.22-7.13 (m, 3H), 4.55-4.39 (m, 2H), 3.67 (s, 3H), 3.18 (t, J = 7.6 Hz, 2H), 3.10-2.90 (m, 2H), 2.88 (t, J = 7.6 Hz, 2H), 2.20-2.06 (m, 1H), 1.92-1.80 (m, 1H), 1.80-1.60 (m, 3H), 1.52-1.34 (m, 2H), 0.89 (d, J = 6.8, 3H), 0.86 (d, J = 6.4, 3H); ^{13}C NMR (100 MHz, $CDCl_3$) δ 196.7, 172.8, 171.8, 160.7, 140.4, 128.6, 128.5, 126.4, 57.9, 53.0, 52.6, 39.9, 38.6, 31.6, 30.9, 28.9, 26.6, 22.2, 19.0, 17.7; HRMS *m/z*: Calcd. for $C_{22}H_{34}N_3O_5$ (M + H^+): 420.2493; Found: 420.2494.

3.5 Synthesis of Compounds 2ae-ai with 2ae as the Representative Example (Fig. 5a)

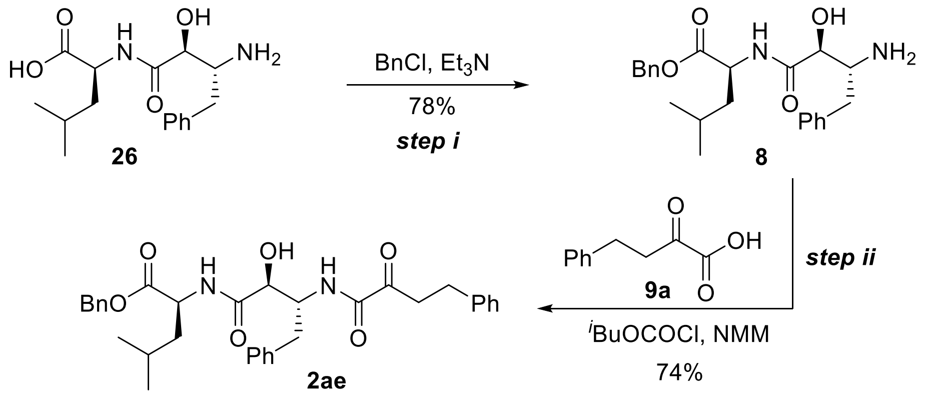

***Step i.* Synthesis of compound 8**

To a 10 mL round-bottom flask were successively added compound **26** (0.100 g, 0.324 mmol), acetone (1.0 mL), and Et_3N (0.0320 g, 0.324 mmol) via a syringe at 0 °C After stirring for 30 min at 0 °C, a solution of BnCl (0.0554 g, 0.324 mmol) in acetone (1.0 mL) was added via a syringe and the reaction mixture was stirred for 4 h at the same temperature. Then the mixture was filtered, and quenched by addition of saturated aqueous $NaHCO_3$ solution (30 mL). The resulting mixture was extracted with ethyl acetate (10 mL × 2). The combined organic layers were washed with brine, dried over Na_2SO_4, filtered, concentrated and purified by column chromatography on silica gel (ethyl acetate) to give compound **8** (0.100 g, 78%) as a white solid.

8: White solid; Mp: 129-131 °C; $[\alpha]_D^{25}$ = -16.4 (*c* = 0.10, $CHCl_3$); ^1H NMR (400 MHz, $CDCl_3$) δ 7.86 (d, *J* = 8.8 Hz, 1H), 7.40-7.27 (m, 7H), 7.26-7.17 (m, 3H), 5.18 (s, 2H), 4.71-4.62 (m, 1H), 3.97 (d, *J* = 2.8 Hz, 1H), 3.62-3.56 (m, 1H), 2.96 (dd, *J* = 13.6, 4.4 Hz, 1H), 2.56 (dd, *J* = 13.6, 10.0 Hz, 1H), 1.75-1.56 (m, 3H), 0.92 (d, *J* = 6.4 Hz, 3H), 0.91 (d, *J* = 6.0 Hz, 3H); ^{13}C NMR (100 MHz, $CDCl_3$) δ 173.3, 172.7, 138.5, 135.5, 129.4, 128.8, 128.7, 128.5, 128.4, 126.7, 72.6, 67.2, 54.3, 50.9, 41.3, 38.3, 25.1, 23.0, 21.9; HRMS m/z: Calcd. for $C_{23}H_{31}N_2O_4$ ($M + H^+$): 399.2278; Found: 399.2281.

Step ii. Synthesis of compound **2ae**[5]

To a 25 mL round-bottom flask was added compound **9a** (0.586 g, 3.29 mmol). The sealed flask was evacuated and refilled with N_2 for three times, followed by addition of isobutyl chloroformate (^{i}BuOCOCl) (0.450 g, 3.29 mmol), *N*-methylmorpholine (NMM) (0.333 g, 3.29 mmol) and anhydrous THF (5 mL) via a syringe at -20 °C. After stirring for 20 min, a solution of compound **8** (0.900 g, 2.74 mmol) in anhydrous THF (3 mL) was added via a syringe at -20 °C. The resulting mixture was allowed to be warmed up to room temperature and stirred at the temperature for 3 h. Then the reaction mixture was concentrated via rotary evaporator under reduced pressure, and quenched by addition of H_2O (40 mL). The resulting mixture was extracted with ethyl acetate (30 mL × 3). The combined organic layers were washed with brine, dried over Na_2SO_4, filtered, concentrated and purified by column chromatography on silica gel (petroleum ether : ethyl acetate = 2:1) to give compound **2ae** (1.10 g, 74%) as a white solid.

2ae: White solid; Mp: 155-157 °C; $[\alpha]_D^{25}$ = 4.8 (*c* = 0.10, $CHCl_3$); ^1H NMR (400 MHz, $CDCl_3$) δ 7.67 (d, *J* = 9.2 Hz, 1H), 7.42-7.16 (m, 16H), 5.20-5.10 (m, 3H), 4.73-4.65 (m, 1H), 4.59-4.51 (m, 1H), 4.25-4.20 (m, 1H), 3.18 (t, *J* = 7.6 Hz, 2H), 3.17-3.04 (m, 1H), 2.94-2.84 (m, 3H), 1.70-1.50 (m, 3H), 0.89 (d, *J* = 6.4 Hz, 3H), 0.86 (d, *J* = 6.4 Hz, 3H); ^{13}C NMR (100 MHz, $CDCl_3$) δ 197.3, 172.9, 172.2, 160.1, 140.4, 137.2, 135.3, 129.3, 128.67, 128.64, 128.56, 128.48, 128.4, 128.2, 126.8, 126.3, 71.8, 67.2, 54.2, 50.6, 40.8, 38.5, 36.7, 29.1, 24.9, 22.9, 21.5; HRMS *m/z*: Calcd. for $C_{33}H_{39}N_2O_6$ ($M + H^+$): 559.2803; Found: 559.2815.

Compounds **2af-ai** were prepared, respectively, in 92%, 97%, 80% and 43% yields by following a similar procedure.

Compound 2af (Fig. 5a)

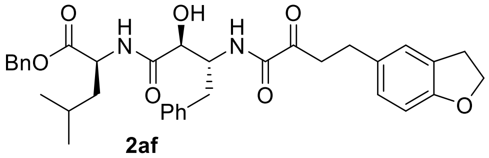

2af

Yellow oil; $[\alpha]_D^{25}$ = 6.5 (*c* = 0.10, $CHCl_3$); ^1H NMR (400 MHz, $CDCl_3$) δ 7.46 (d, *J* = 8.8 Hz, 1H), 7.30-7.06 (m, 11H), 6.93 (s, 1H), 6.81 (d, *J* = 8.4 Hz, 1H), 6.61 (d, *J* = 8.0 Hz, 1H), 5.06 (d, *J* = 12.4 Hz, 1H), 5.03 (d, *J* = 12.4 Hz, 1H), 4.80 (d, *J* = 6.4 Hz, 1H), 4.58-4.51 (m, 1H), 4.45 (t, *J* = 8.4 Hz, 2H), 4.42-4.34 (m, 1H), 4.09 (dd, *J* = 6.0, 2.4 Hz, 1H), 3.07 (t, *J* = 8.4 Hz, 2H), 3.03 (t, *J* = 7.6 Hz, 2H), 2.99 (dd, *J* = 13.6, 7.2 Hz, 1H), 2.82 (dd, *J* = 13.6, 8.4 Hz, 1H), 2.72 (t, *J* = 7.2 Hz, 2H), 1.58-1.36 (m, 3H), 0.77 (d, *J* = 6.4 Hz, 3H), 0.74 (t, *J* = 6.4 Hz, 3H); ^{13}C NMR (100 MHz, $CDCl_3$) δ 197.3, 172.7, 172.0, 160.5, 158.7, 137.2, 135.4, 132.4, 129.3, 128.8, 128.7, 128.5, 128.3, 127.9, 127.3, 127.0, 125.0, 109.2, 72.2, 71.3, 67.3, 54.5, 50.7, 41.0, 39.0, 36.6, 29.9, 28.7, 24.9, 22.9, 21.5; HRMS *m/z*: Calcd. for $C_{35}H_{41}N_2O_7$ (M + H^+): 601.2908; Found: 601.2913.

Compound 2ag (Fig. 5a)

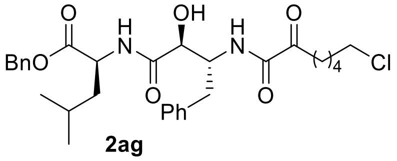

2ag

Yellow oil; $[\alpha]_D^{25}$ = 5.1 (*c* = 0.10, $CHCl_3$);^1H NMR (400 MHz, $CDCl_3$) δ 7.51 (d, *J* = 8.8 Hz, 1H), 7.41-7.21 (m, 10H), 7.11 (d, *J* = 8.8 Hz, 1H), 5.15 (s, 2H), 4.67-4.60 (m, 1H), 4.50-4.42 (m, 1H), 4.19 (d, *J* = 2.8 Hz, 1H), 3.54 (t, *J* = 6.8 Hz, 2H), 3.10 (dd, *J* = 13.6, 7.2 Hz, 1H), 2.95 (dd, *J* = 13.6, 8.2 Hz, 1H), 2.84 (t, *J* = 7.2 Hz, 2H), 1.89-1.75 (m, 2H), 1.67-1.40 (m, 7H), 0.86 (dd, *J* = 12.8, 6.4 Hz, 6H); ^{13}C NMR (150 MHz, $CDCl_3$) δ 197.8, 172.6, 171.9, 160.7, 137.2, 135.4, 129.3, 128.9, 128.8, 128.6, 128.3,

127.1, 72.4, 67.3, 54.6, 50.8, 44.8, 41.1, 36.6, 36.5, 32.4, 26.4, 25.0, 23.0, 22.5, 21.6; HRMS *m/z*: Calcd. for $C_{30}H_{40}ClN_2O_6$ (M + H^+): 559.2569; Found: 559.2573.

Compound 2ah (Fig. 5a)

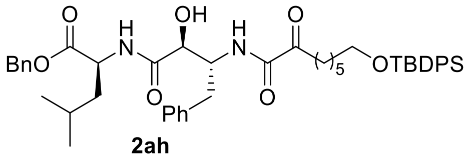

2ah

Colorless oil; $[\alpha]_D^{25}$ = 4.9 (*c* = 0.10, $CHCl_3$); ^1H NMR (400 MHz, $CDCl_3$) δ 7.70-7.62 (m, 4H), 7.47 (d, *J* = 8.8 Hz, 1H), 7.45-7.24 (m, 13H), 7.24-7.17 (m, 3H), 7.09 (d, *J* = 8.8 Hz, 1H), 5.13 (s, 2H), 4.61 (td, *J* = 9.2, 4.8 Hz, 1H), 4.42 (qd, *J* = 8.0, 2.4 Hz, 1H), 4.18 (d, *J* = 2.4 Hz, 1H), 3.64 (t, *J* = 6.4 Hz, 2H), 3.08 (dd, *J* = 13.6, 7.2 Hz, 1H), 2.95 (dd, *J* = 13.6, 8.0 Hz, 1H), 2.78 (t, *J* = 7.2 Hz, 2H), 1.65-1.43 (m, 7H), 1.39-1.31 (m, 2H), 1.31-1.22 (m, 2H), 1.04 (s, 9H), 0.85 (d, *J* = 6.4 Hz, 3H), 0.82 (d, *J* = 6.4 Hz, 3H); ^{13}C NMR (100 MHz, $CDCl_3$) δ 198.1, 172.7, 172.0, 160.8, 137.2, 135.7, 135.4, 134.2, 129.6, 129.3, 128.8, 128.7, 128.6, 128.3, 127.7, 127.1, 72.5, 67.3, 63.9, 54.8, 50.7, 41.0, 36.8, 36.4, 32.5, 29.0, 27.0, 25.7, 24.9, 23.2, 23.0, 21.5, 19.3; HRMS *m/z*: Calcd. for $C_{47}H_{60}N_2NaO_7Si$ (M + Na^+): 815.4062; Found: 815.4068.

Compound 2ai (Fig. 5a)

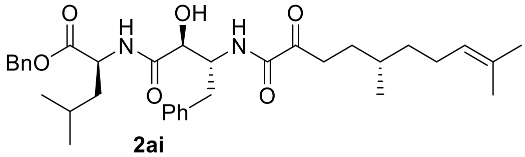

2ai

Colorless oil; $[\alpha]_D^{25}$ = 0.4 (*c* = 0.10, $CHCl_3$); ^1H NMR (400 MHz, $CDCl_3$) δ 7.46 (d, *J* = 8.8 Hz, 1H), 7.38-7.27 (m, 7H), 7.24-7.18 (m, 3H), 7.07 (d, *J* = 8.8 Hz, 1H), 5.13 (s, 2H), 5.10-5.04 (m, 1H), 4.82 (d, *J* = 6.4 Hz, 1H), 4.65-4.58 (m, 1H), 4.46-4.38 (m, 1H), 4.17 (dd, *J* = 6.4, 2.8 Hz, 1H), 3.08 (dd, *J* = 13.6, 7.2 Hz, 1H), 2.96 (dd, *J* = 13.6, 8.4 Hz, 1H), 2.81 (t, *J* = 7.6 Hz, 2H), 2.04-1.89 (m, 2H), 1.67 (s, 3H), 1.57-1.24 (m, 10H), 1.20-1.10 (m, 1H), 0.87 (d, *J* = 6.4 Hz, 3H), 0.85 (d, *J* = 6.8 Hz, 3H), 0.82 (d, *J* = 6.4

Hz, 3H); ^{13}C NMR (100 MHz, $CDCl_3$) δ 198.5, 172.8, 172.1, 160.6, 137.3, 135.4, 131.4, 129.3, 128.75, 128.72, 128.5, 128.3, 127.0, 124.7, 72.3, 67.3, 54.5, 50.7, 41.0, 36.9, 36.6, 34.6, 32.1, 30.1, 25.8, 25.6, 24.9, 23.0, 21.5, 19.4, 17.8; HRMS *m/z*: Calcd. for $C_{35}H_{49}N_2O_6$ (M + H^+): 593.3585; Found: 593.3597.

4. Procedure for Synthesis of Peptides 3a-y by Asymmetric Transamination Using 3a as the Representative Example (Fig. 4)

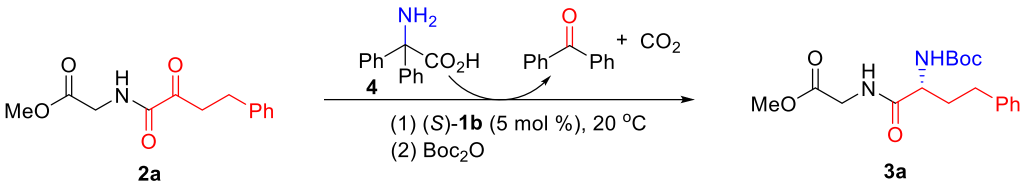

To a 2.5 mL vial equipped with a magnetic stirring bar were successively added α-keto amide **2a** (0.0249 g, 0.10 mmol), *N*-quaternized chiral pyridoxamine (*S*)-**1b** (0.0026 g, 0.0050 mmol), 2,2-diphenylglycine **4** (0.0250 g, 0.11 mmol), CH_3COOH (0.024 g, 0.40 mmol), Na_2HPO_4 (0.0284 g, 0.20 mmol), CF_3CH_2OH (0.48 mL), and H_2O (0.12 mL). After the suspension mixture was stirred at 20 °C for 72 h, MeOH (1.0 mL) was added to dissolve the solid completely followed by the addition of di-*tert*-butyl dicarbonate (Boc_2O) (0.0655 g, 0.30 mmol) at room temperature. Upon stirring for 3 h, the reaction mixture was evaporated to remove most of the solvent. The residue was submitted to flash column chromatography on silica gel (petroleum ether : ethyl acetate = 3:1) to give product **3a** (0.0308 g, 88%) as a white solid. Note: Before loading sample, the silica gel was rinsed with a mixed solvent [a mixture of petroleum ether, DCM, and 2.9 M ammonia solution in ethanol (1:1:0.02)]

Compounds **3d-e**, **3j**, **3l** and **3g** were prepared by a similar procedure with MeOH/H_2O (4/1) as the solvent at 25 °C. Compounds **3k**, **3m-t** and **3w-y** were prepared by a similar procedure with MeOH/H_2O (4/1) as the solvent at 20 °C. Compounds **3a-c**, **3f** and **3h-i** were prepared by a similar procedure with TFEA/H_2O (4/1) as the solvent at 20 °C. Compounds **3u** and **3v** were prepared by a similar procedure with MeOH/H_2O/DCM (4/1/1) as the solvent at 20 °C. Compounds **3d-e**, **3j**, and **3l-y** were carried out in a double scale.

The enantiomeric excesses (ee's) of products **3a-c**, **3f-i**, **3k** and the dr values of products **3m-x** were determined by chiral HPLC analysis without transformation. The

ee values for **3d**, **3e**, and **3j** were determined by chiral HPLC analysis after the products were converted to the corresponding *N*-benzoyl derivatives by removal of the Boc-group with TFA (50.0 equiv.) followed by treatment with benzoyl chloride (2.0 equiv.) and Et_3N (pH 8-9) in THF at room temperature. The dr values of **3l** and **3y** were determined by chiral HPLC analysis after the products were converted to the corresponding *N*-benzoyl derivatives by removal of the Boc-group with concentrated H_2SO_4 (20.0 equiv.) in THF at 50 °C, followed by treatment with benzoyl chloride (2.0 equiv.) and Et_3N (pH 8-9) at room temperature for 2 h.

3a: White solid; Mp: 46-48 °C; $[\alpha]_D^{25}$ = 10.6 (*c* = 0.10, $CHCl_3$); ^1H NMR (400 MHz, $CDCl_3$) δ 7.20 (t, *J* = 7.6 Hz, 2H), 7.13-7.08 (m, 3H), 6.71 (s, 1H), 5.11 (d, *J* = 8.0 Hz, 1H), 4.11-3.89 (m, 3H), 3.67 (s, 3H), 2.62 (t, *J* = 8.0 Hz, 2H), 2.15-2.05 (m, 1H), 1.91-1.81 (m, 1H), 1.37 (s, 9H); ^{13}C NMR (100 MHz, $CDCl_3$) δ 172.6, 170.2, 155.8, 141.0, 128.49, 128.46, 126.1, 80.1, 54.0, 52.3, 41.1, 34.3, 31.8, 28.4; HRMS *m/z* Calcd. for $C_{18}H_{27}N_2O_5$ (M + H^+): 351.1914; Found: 351.1919.

Compound 3b (Fig. 4)

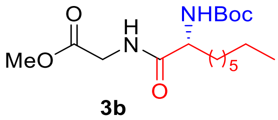

3b

Light yellow oil; $[\alpha]_D^{25}$ = 13.3 (*c* = 0.10, $CHCl_3$); ^1H NMR (600 MHz, $CDCl_3$) δ 6.78 (s, 1H), 5.09 (d, *J* = 6.4 Hz, 1H), 4.17-4.08 (m, 1H), 4.07-3.98 (m, 2H), 3.73 (s, 3H), 1.86-1.78 (m, 1H), 1.61-1.52 (m, 1H), 1.43 (s, 9H), 1.35-1.20 (m, 10H), 0.85 (t, *J* = 7.2 Hz, 3H); ^{13}C NMR (150 MHz, $CDCl_3$) δ 172.8, 170.2, 155.8, 80.2, 54.6, 52.4, 41.2, 32.7, 31.9, 29.4, 29.2, 28.4, 25.7, 22.7, 14.2; HRMS *m/z* Calcd. for $C_{17}H_{33}N_2O_5$ (M + H^+): 345.2384; Found: 345.2386.

Compound 3c (Fig. 4)

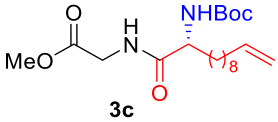

3c

White solid; Mp: 61-63 °C; $[\alpha]_D^{25}$ = 17.9 (c = 0.10, $CHCl_3$); ^{1}H NMR (600 MHz, $CDCl_3$) δ 6.66 (t, J = 6.0 Hz, 1H), 5.83-5.76 (m, 1H), 5.02-4.95 (m, 2H), 4.93-4.90 (m, 1H), 4.16-4.07 (m, 1H), 4.04 (d, J = 5.4 Hz, 2H), 3.74 (s, 3H), 2.05-1.99 (m, 2H), 1.87-1.80 (m, 1H), 1.62-1.54 (m, 1H), 1.44 (s, 9H), 1.37-1.23 (m, 12H); ^{13}C NMR (150 MHz, $CDCl_3$) δ 172.6, 170.2, 155.8, 139.3, 114.3, 80.2, 54.6, 52.5, 41.3, 33.9, 32.6, 29.50, 29.49, 29.41, 29.2, 29.0, 28.4, 25.7; HRMS m/z Calcd. for $C_{20}H_{37}N_2O_5$ (M + H^+): 385.2697; Found: 385.2700.

Compound 3d (Fig. 4)

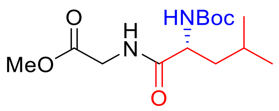

3d

White solid; Mp: 195-197 °C; $[\alpha]_D^{25}$ = 24.4 (c = 0.10, $CHCl_3$); ^{1}H NMR (600 MHz, $CDCl_3$) δ 6.69 (s, 1H), 4.90 (d, J = 8.4 Hz, 1H), 4.19-4.03 (m, 1H), 4.04 (d, J = 5.4 Hz, 2H), 3.75 (s, 3H), 1.72-1.66 (m, 2H), 1.51-1.41 (m, 10H), 0.94 (t, J = 6.3 Hz, 6H); ^{13}C NMR (150 MHz, $CDCl_3$) δ 173.1, 170.3, 155.9, 80.2, 53.0, 52.4, 41.3, 41.2, 28.4, 24.8, 23.1, 22.0; HRMS m/z Calcd. for $C_{14}H_{26}N_2NaO_5$ (M + Na^+): 325.1734; Found: 325.1737.

Compound 3e (Fig. 4)

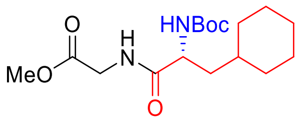

3e

White solid; Mp: 75-77 °C; $[\alpha]_D^{25}$ = 20.5 (c = 0.10, $CHCl_3$); ^{1}H NMR (600 MHz, $CDCl_3$) δ 6.91 (s, 1H), 5.07 (d, J = 8.4 Hz, 1H), 4.24-4.18 (m, 1H), 4.05-3.95 (m, 2H), 3.71 (s, 3H), 1.78-1.74 (m, 1H), 1.70-1.59 (m, 5H), 1.47-1.42 (m, 1H), 1.41 (s, 9H), 1.36-1.07 (m, 4H), 0.97-0.79 (m, 2H); ^{13}C NMR (150 MHz, $CDCl_3$) δ 173.3, 170.3, 155.9, 80.1, 52.3, 41.2, 40.0, 34.1, 33.7, 32.6, 28.4, 26.5, 26.3, 26.1; HRMS m/z Calcd. for $C_{17}H_{31}N_2O_5$ (M + H^+): 343.2227; Found: 343.2231.

Compound 3f (Fig. 4)

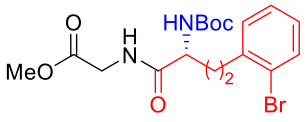

3f

White solid; Mp: 126-128 °C; $[\alpha]_D^{25}$ = -6.9 (c = 0.10, $CHCl_3$); ^1H NMR (400 MHz, $CDCl_3$) δ 7.51 (d, J = 7.2 Hz, 1H), 7.24-7.21 (m, 2H), 7.08-7.03 (m, 1H), 6.74 (s, 1H), 5.15 (d, J = 8.0 Hz, 1H), 4.25-4.15 (m, 1H), 4.05 (d, J = 5.2 Hz, 2H), 3.75 (s, 3H), 2.88-2.75 (m, 2H), 2.23-2.11 (m, 1H), 1.96-1.86 (m, 1H), 1.46 (s, 9H); ^{13}C NMR (100 MHz, $CDCl_3$) δ 172.2, 170.2, 156.8, 140.4, 133.0, 130.6, 128.1, 127.8, 124.5, 80.5, 54.2, 52.5, 41.3, 32.6, 32.3, 28.5; HRMS *m/z* Calcd. for $C_{18}H_{26}BrN_2O_5$ (M + H^+): 429.1020; Found: 429.1022.

Compound 3g (Fig. 4)

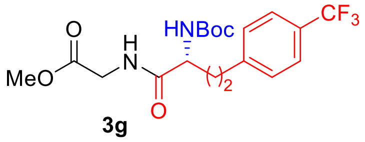

3g

Light yellow oil; $[\alpha]_D^{25}$ = -13.3 (c = 0.10, $CHCl_3$); ^1H NMR (400 MHz, $CDCl_3$) δ 7.53 (d, J = 8.0 Hz, 2H), 7.30 (d, J = 8.0 Hz, 2H), 6.69 (t, J = 5.6 Hz, 1H), 5.12 (d, J = 8.4 Hz, 1H), 4.22-4.12 (m, 1H), 4.09-3.97 (m, 2H), 3.75 (s, 3H), 2.76 (t, J = 8.0 Hz, 2H), 2.24-2.15 (m, 1H), 1.99-1.88 (m, 1H), 1.44 (s, 9H); ^{13}C NMR (100 MHz, $CDCl_3$) δ 172.3, 170.2, 155.8, 145.2, 128.9, 128.6 (q, J = 32.2 Hz), 125.5 (q, J = 3.6 Hz), 124.4 (q, J = 270.2 Hz), 80.4, 53.9, 52.5, 41.2, 33.9, 31.7, 28.4; HRMS *m/z* Calcd. for $C_{19}H_{26}F_3N_2O_5$ (M + H^+): 419.1788; Found: 419.1793.

Compound 3h (Fig. 4)

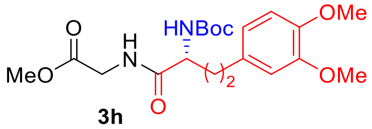

3h

Light yellow oil; $[\alpha]_D^{25}$ = -8.4 (c = 0.10, $CHCl_3$); ^1H NMR (600 MHz, $CDCl_3$) δ 6.77 (d, J = 8.4 Hz, 1H), 6.72-6.71 (m, 2H), 6.67 (t, J = 5.4 Hz, 1H), 5.09 (d, J = 7.8 Hz, 1H), 4.20-4.10 (m, 1H), 4.07-3.98 (m, 2H), 3.85 (s, 3H), 3.84 (s, 3H), 3.74 (s, 3H), 2.64

(t, *J* = 7.8 Hz, 2H), 2.18-2.11 (m, 1H), 1.95-1.88 (m, 1H), 1.44 (s, 9H); ^{13}C NMR (150 MHz, $CDCl_3$) δ 172.4, 170.2, 155.8, 149.1, 147.5, 133.5, 120.4, 111.9, 111.5, 80.4, 56.0, 55.9, 54.0, 52.5, 41.2, 34.3, 31.5, 28.4; HRMS *m/z* Calcd. for $C_{20}H_{30}N_2NaO_7$ (M + Na$^+$): 433.1945; Found: 433.1948.

Compound 3i (Fig. 4)

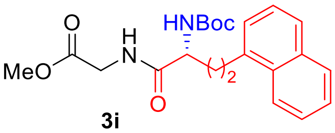

3i

White solid; Mp: 118-120 °C; $[\alpha]_D^{25}$ = -5.6 (*c* = 0.10, $CHCl_3$); ^{1}H NMR (400 MHz, $CDCl_3$) δ 8.02 (d, *J* = 8.0 Hz, 1H), 7.84 (dd, *J* = 7.6, 2.0 Hz, 1H), 7.71 (d, *J* = 8.0 Hz, 1H), 7.52-7.44 (m, 2H), 7.40-7.31 (m, 2H), 6.76 (s, 1H), 5.20 (d, *J* = 8.0 Hz, 1H), 4.34-4.24 (m, 1H), 4.05 (d, *J* = 5.2 Hz, 2H), 3.73 (s, 3H), 3.16 (t, *J* = 8.0 Hz, 2H), 2.38-2.28 (m, 1H), 2.11-2.01 (m, 1H), 1.46 (s, 9H); ^{13}C NMR (100 MHz, $CDCl_3$) δ 172.4, 170.1, 155.9, 137.2, 134.0, 131.8, 128.9, 127.1, 126.2, 126.1, 125.7, 125.6, 123.7, 80.4, 54.4, 52.4, 41.3, 33.5, 29.0, 28.4; HRMS *m/z* Calcd. for $C_{22}H_{28}N_2NaO_5$ (M + Na$^+$): 423.1890; Found: 423.1896.

Compound 3j (Fig. 4)

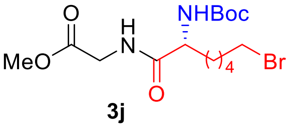

3j

Colorless oil; $[\alpha]_D^{25}$ = -4.9 (*c* = 0.10, $CHCl_3$); ^{1}H NMR (600 MHz, $CDCl_3$) δ 6.75 (s, 1H), 5.09 (d, *J* = 8.4 Hz, 1H), 4.19-4.10 (m, 1H), 4.07-3.99 (m, 2H), 3.74 (s, 3H), 3.38 (t, *J* = 6.6 Hz, 2H), 1.88-1.80 (m, 4H), 1.64-1.57 (m, 1H), 1.47-1.36 (m, 12H); ^{13}C NMR (150 MHz, $CDCl_3$) δ 172.5, 170.2, 155.8, 80.3, 54.4, 52.5, 41.2, 33.7, 32.6, 32.4, 28.4, 27.9, 24.8; HRMS *m/z* Calcd. for $C_{15}H_{27}BrN_2NaO_5$ (M + Na$^+$): 417.0996; Found: 417.1000.

Compound 3k (Fig. 4)

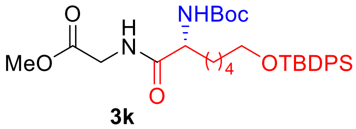

3k

Light yellow oil; $[\alpha]_D^{25}$ = -8.9 (c = 0.10, $CHCl_3$); ^1H NMR (600 MHz, $CDCl_3$) δ 7.68-7.65 (m, 4H), 7.43-7.35 (m, 6H), 6.69 (t, J = 5.4 Hz, 1H), 5.03 (d, J = 7.8 Hz, 1H), 4.14-4.08 (m, 1H), 4.03 (d, J = 4.8 Hz, 2H), 3.74 (s, 3H), 3.64 (t, J = 6.6 Hz, 2H), 1.86-1.80 (m, 1H), 1.61-1.53 (m, 3H), 1.43 (s, 9H), 1.40-1.31 (m, 4H), 1.04 (s, 9H); ^{13}C NMR (150 MHz, $CDCl_3$) δ 172.6, 170.2, 155.8, 135.7, 134.2, 129.6, 127.7, 80.2, 63.9, 54.6, 52.4, 41.2, 32.6, 32.5, 28.4, 27.0, 25.7, 25.5, 19.3; HRMS *m/z* Calcd. for $C_{31}H_{46}N_2NaO_6Si$ (M + Na^+): 593.3017; Found: 593.3032.

Compound 3l (Fig. 4)

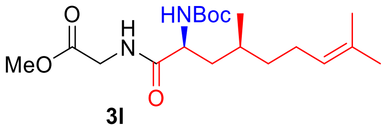

3l

Light yellow oil; $[\alpha]_D^{25}$ = -29.1 (c = 0.10, $CHCl_3$); ^1H NMR (600 MHz, $CDCl_3$) δ 6.78 (s, 1H), 5.08-4.97 (m, 2H), 4.21-4.17 (m, 1H), 4.05-3.97 (m, 2H), 3.73 (s, 3H), 2.02-1.87 (m, 2H), 1.83-1.77 (m, 1H), 1.65 (d, J = 1.2 Hz, 3H), 1.57 (d, J = 1.2 Hz, 3H), 1.56-1.51 (m, 1H), 1.42 (s, 9H), 1.42-1.37 (s, 2H), 1.15-1.08 (m, 1H), 0.92 (d, J = 6.6 Hz, 3H); ^{13}C NMR (150 MHz, $CDCl_3$) δ 173.0, 170.2, 155.8, 131.4, 124.6, 80.2, 52.8, 52.4, 41.2, 39.8, 36.4, 29.3, 28.4, 25.8, 25.4, 19.9, 17.8; HRMS *m/z* Calcd. for $C_{19}H_{35}N_2O_5$ (M + H^+): 371.2540; Found: 371.2544.

Compound (*S*,*R*)-3m (Fig. 4)

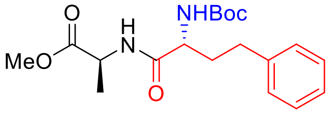

(*S*,*R*)-**3m**

Light yellow oil; $[\alpha]_D^{25}$ = -6.6 (c = 0.10, $CHCl_3$); ^1H NMR (400 MHz, $CDCl_3$) δ 7.31-7.23 (m, 2H), 7.22-7.14 (m, 3H), 6.75 (s, 1H), 5.12 (d, J = 8.0 Hz, 1H), 4.62-4.50 (m, 1H), 4.24-4.10 (m, 1H), 3.73 (s, 3H), 2.68 (t, J = 7.8 Hz, 2H), 2.23-2.11 (m, 1H), 1.98-

1.86 (m, 1H), 1.45 (s, 9H), 1.40 (d, *J* = 7.2 Hz, 3H); ^{13}C NMR (100 MHz, $CDCl_3$) δ 173.3, 171.6, 155.8, 141.0, 128.6, 128.5, 126.3, 80.3, 54.1, 52.6, 48.1, 34.1, 31.9, 28.4, 18.4; HRMS *m/z* Calcd. for $C_{19}H_{29}N_2O_5$ (M + H$^+$): 365.2071; Found: 365.2076.

Compound (*S*,*S*)-3m (Fig. 4)

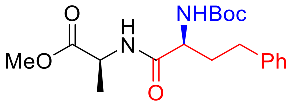

(*S*,*S*)-**3m**

Light yellow oil; $[\alpha]_D^{25}$ = -20.8 (*c* = 0.10, $CHCl_3$); ^{1}H NMR (400 MHz, $CDCl_3$) δ 7.30-7.23 (m, 2H), 7.21-7.14 (m, 3H), 6.69 (d, *J* = 7.6 Hz, 1H), 5.21 (d, *J* = 8.4 Hz, 1H), 4.60-4.50 (m, 1H), 4.19-4.10 (m, 1H), 3.73 (s, 3H), 2.69 (t, *J* = 8.0 Hz, 2H), 2.20-2.08 (m, 1H), 1.98-1.85 (m, 1H), 1.44 (s, 9H), 1.38 (d, *J* = 7.2 Hz, 3H); ^{13}C NMR (100 MHz, $CDCl_3$) δ 173.2, 171.7, 155.7, 141.0, 128.6, 128.5, 126.2, 80.2, 54.1, 52.6, 48.1, 34.3, 31.8, 28.4, 18.2; HRMS *m/z*: Calcd. for $C_{19}H_{29}N_2O_5$ (M + H$^+$): 365.2071; Found: 365.2072.

Compound (*R*,*R*)-3n (Fig. 4)

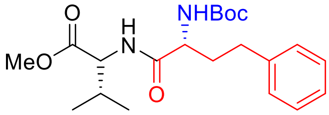

(*R*,*R*)-**3n**

Light yellow solid; Mp: 131-133 °C; $[\alpha]_D^{25}$ = -19.0 (*c* = 0.10, $CHCl_3$); ^{1}H NMR (400 MHz, $CDCl_3$) δ 7.31-7.27 (m, 2H), 7.22-7.15 (m, 3H), 6.57 (d, *J* = 8.8 Hz, 1H), 5.07 (d, *J* = 8.0 Hz, 1H), 4.53 (dd, *J* = 8.8, 5.2 Hz, 1H), 4.13-4.06 (m, 1H), 3.73 (s, 3H), 2.68 (t, *J* = 8.0 Hz, 2H), 2.22-2.10 (m, 2H), 1.97-1.86 (m, 1H), 1.44 (s, 9H), 0.92 (d, *J* = 7.2 Hz, 3H), 0.89 (d, *J* = 6.8 Hz, 3H); ^{13}C NMR (100 MHz, $CDCl_3$) δ 172.3, 172.0, 155.8, 141.0, 128.62, 128.56, 126.2, 80.2, 57.2, 54.2, 52.3, 33.7, 31.9, 31.3, 28.4, 19.1, 17.7; HRMS *m/z* Calcd. for $C_{21}H_{33}N_2O_5$ (M + H$^+$): 393.2384; Found: 393.2383.

Compound (*R*,*S*)-3n (Fig. 4)

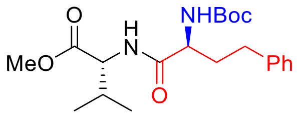

(*R*,*S*)-**3n**

Light yellow solid; Mp: 140-142 °C; $[\alpha]_D^{25}$ = -45.9 (*c* = 0.10, $CHCl_3$); ^{1}H NMR (400 MHz, $CDCl_3$) δ 7.32-7.24 (m, 2H), 7.23-7.15 (m, 3H), 6.69 (d, *J* = 8.8 Hz, 1H), 5.03 (d, *J* = 7.6 Hz, 1H), 4.53 (dd, *J* = 8.8, 5.2 Hz, 1H), 4.24-4.08 (m, 1H), 3.72 (s, 3H), 2.69 (t, *J* = 8.0 Hz, 2H), 2.26-2.12 (m, 2H), 1.98-1.85 (m, 1H), 1.45 (s, 9H), 0.94 (d, *J* = 6.8 Hz, 3H), 0.89 (d, *J* = 6.8 Hz, 3H); ^{13}C NMR (100 MHz, $CDCl_3$) δ 172.3, 172.1, 155.8, 141.0, 128.7, 128.5, 126.3, 80.4, 57.1, 54.4, 52.3, 34.0, 32.0, 31.3, 28.4, 19.1, 17.8; HRMS *m/z*: Calcd. for $C_{21}H_{33}N_2O_5$ (M + H^+): 393.2384; Found: 393.2386.

Compound 3o (Fig. 4)

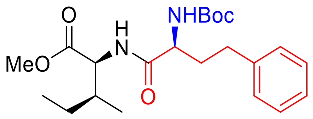

3o

Yellow solid; Mp: 116-118 °C; $[\alpha]_D^{25}$ = -32.6 (*c* = 0.10, $CHCl_3$); ^{1}H NMR (400 MHz, $CDCl_3$) δ 7.31-7.24 (m, 2H), 7.22-7.12 (m, 3H), 6.58 (d, *J* = 8.4 Hz, 1H), 5.10 (d, *J* = 8.4 Hz, 1H), 4.57 (dd, *J* = 8.8, 5.2 Hz, 1H), 4.14-4.06 (m, 1H), 3.72 (s, 3H), 2.68 (t, *J* = 8.0 Hz, 2H), 2.20-2.10 (m, 1H), 1.97-1.86 (m, 2H), 1.44 (s, 9H), 1.42-1.35 (m, 1H), 1.22-1.10 (m, 1H), 0.93-0.86 (m, 6H); ^{13}C NMR (100 MHz, $CDCl_3$) δ 172.2, 171.8, 155.8, 141.0, 128.62, 128.56, 126.2, 80.2, 56.6, 54.1, 52.2, 37.9, 33.7, 31.9, 28.4, 25.1, 15.5, 11.6; HRMS *m/z* Calcd. for $C_{22}H_{35}N_2O_5$ (M + H^+): 407.2540; Found: 407.2544.

Compound 3p (Fig. 4)

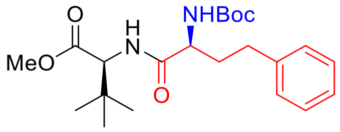

3p

Yellow oil; $[\alpha]_D^{25}$ = -46.6 (*c* = 0.10, $CHCl_3$); ^1H NMR (400 MHz, $CDCl_3$) δ 7.30-7.24 (m, 2H), 7.22-7.14 (m, 3H), 6.71 (d, *J* = 9.2 Hz, 1H), 5.11 (d, *J* = 8.0 Hz, 1H), 4.43 (d, *J* = 9.2 Hz, 1H), 4.12-4.04 (m, 1H), 3.71 (s, 3H), 2.66 (t, *J* = 7.8 Hz, 2H), 2.23-2.09 (m, 1H), 2.00-1.86 (m, 1H), 1.44 (s, 9H), 0.96 (s, 9H); ^{13}C NMR (100 MHz, $CDCl_3$) δ 171.8, 171.7, 155.8, 141.0, 128.6, 128.5, 126.2, 80.3, 60.2, 54.2, 51.9, 34.9, 33.4, 32.0, 28.4, 26.6; HRMS *m/z* Calcd. for $C_{22}H_{35}N_2O_5$ (M + H^+): 407.2540; Found: 407.2543.

Compound 3q (Fig. 4)

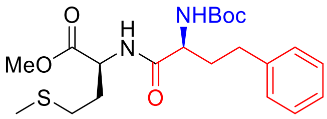

3q

Yellow oil; $[\alpha]_D^{25}$ = -18.8 (*c* = 0.10, $CHCl_3$); ^1H NMR (400 MHz, $CDCl_3$) δ 7.31-7.24 (m, 2H), 7.22-7.14 (m, 3H), 6.74 (d, *J* = 8.0 Hz, 1H), 5.11 (d, *J* = 8.0 Hz, 1H), 4.73-4.65 (m, 1H), 4.15-4.06 (m, 1H), 3.74 (s, 3H), 2.68 (t, *J* = 7.6 Hz, 2H), 2.49 (t, *J* = 7.6 Hz, 2H), 2.21-2.10 (m, 2H), 2.07 (s, 3H), 2.02-1.87 (m, 2H), 1.44 (s, 9H); ^{13}C NMR (100 MHz, $CDCl_3$) δ 172.2, 171.9, 155.7, 140.9, 128.64, 128.56, 126.3, 80.3, 54.2, 52.7, 51.6, 33.9, 31.8, 31.6, 29.9, 28.4, 15.5; HRMS *m/z* Calcd. for $C_{21}H_{33}N_2O_5S$ (M + H^+): 425.2105; Found: 425.2106.

Compound (*S*,*R*)-3r (Fig. 4)

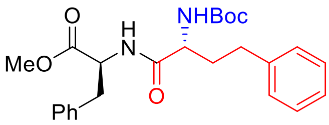

(*S*,*R*)-**3r**

White solid; Mp: 158-160 °C; $[\alpha]_D^{25}$ = 9.1 (*c* = 0.10, $CHCl_3$); ^1H NMR (400 MHz, $CDCl_3$) δ 7.30-7.16 (m, 6H), 7.13-7.08 (m, 4H), 6.63 (d, *J* = 8.0 Hz, 1H), 5.02 (d, *J* = 8.4 Hz, 1H), 4.87 (dt, *J* = 8.0, 6.0 Hz, 1H), 4.18-4.08 (m, 1H), 3.71 (s, 3H), 3.17-3.03 (m, 2H), 2.58 (t, *J* = 8.0 Hz, 2H), 2.14-2.04 (m, 1H), 1.87-1.79 (m, 1H), 1.43 (s, 9H); ^{13}C NMR (100 MHz, $CDCl_3$) δ 171.9, 171.7, 155.7, 141.0, 135.9, 129.3, 128.7, 128.6, 128.5, 127.3, 126.2, 80.2, 54.1, 53.2, 52.5, 38.0, 34.1, 31.7, 28.4; HRMS *m/z* Calcd. for $C_{25}H_{33}N_2O_5$ (M + H^+): 441.2384; Found: 441.2384.

Compound (*S*,*S*)-3r (Fig. 4)

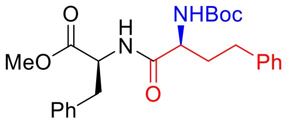

(*S*,*S*)-**3r**

Light yellow solid; Mp: 152-154 °C; $[\alpha]_D^{25}$ = 10.4 (*c* = 0.10, $CHCl_3$); ^{1}H NMR (400 MHz, $CDCl_3$) δ 7.31-7.22 (m, 5H), 7.21-7.06 (m, 5H), 6.44 (d, *J* = 7.6 Hz, 1H), 5.00 (d, *J* = 8.4 Hz, 1H), 4.89-4.81 (m, 1H), 4.12-4.04 (m, 1H), 3.71 (s, 3H), 3.15 (dd, *J* = 13.6, 6.0 Hz, 1H), 3.08 (dd, *J* = 13.6, 5.6 Hz, 1H), 2.64 (t, *J* = 7.6 Hz, 2H), 2.18-2.03 (m, 1H), 1.94-1.77 (m, 1H), 1.44 (s, 9H); ^{13}C NMR (100 MHz, $CDCl_3$) δ 171.7, 171.6, 155.6, 140.9, 135.8, 129.4, 128.7, 128.6, 128.5, 127.3, 126.3, 80.2, 54.2, 53.3, 52.5, 38.0, 34.2, 31.8, 28.4; HRMS *m/z* Calcd. for $C_{25}H_{33}N_2O_5$ (M + H^+): 441.2384; Found: 441.2388.

Compound 3s (Fig. 4)

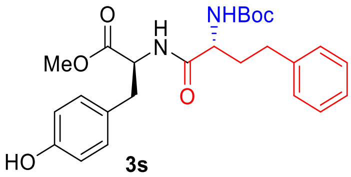

3s

White solid; Mp: 72-74 °C; $[\alpha]_D^{25}$ = 3.4 (*c* = 0.10, $CHCl_3$); ^{1}H NMR (400 MHz, $CDCl_3$) δ 7.29-7.23 (m, 2H), 7.17 (t, *J* = 7.6 Hz, 1H), 7.11 (d, *J* = 7.6 Hz, 2H), 6.92 (d, *J* = 7.6 Hz, 2H), 6.78 (d, *J* = 8.0 Hz, 1H), 6.71-6.60 (m, 3H), 5.14 (d, *J* = 8.4 Hz, 1H), 4.82 (dt, *J* = 8.4, 6.0 Hz, 1H), 4.20-4.10 (m, 1H), 3.70 (s, 3H), 3.09-2.92 (m, 2H), 2.58 (t, *J* = 8.0 Hz, 2H), 2.15-2.05 (m, 1H), 1.89-1.76 (m, 1H), 1.44 (s, 9H); ^{13}C NMR (100 MHz, $CDCl_3$) δ 172.3, 172.0, 155.9, 155.5, 140.9, 130.4, 128.6, 128.5, 127.1, 126.2, 115.8, 80.5, 54.2, 53.4, 52.6, 37.2, 34.0, 31.8, 28.4; HRMS *m/z* Calcd. for $C_{25}H_{33}N_2O_6$ (M + H^+): 457.2333; Found: 457.2331

Compound 3t (Fig. 4)

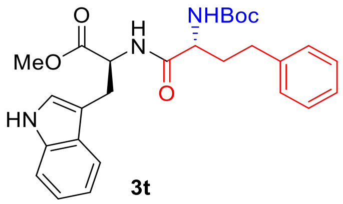

3t

White solid; Mp: 82-84 °C; $[\alpha]_D^{25}$ = 3.4 (*c* = 0.10, $CHCl_3$); ^{1}H NMR (400 MHz, $CDCl_3$) δ 8.20 (s, 1H), 7.52 (d, *J* = 8.0 Hz, 1H), 7.31 (d, *J* = 8.4 Hz, 1H), 7.27-7.23 (m, 2H), 7.21-7.13 (m, 2H), 7.11-7.02 (m, 3H), 6.97 (d, *J* = 2.4 Hz, 1H), 6.74 (d, *J* = 8.0 Hz, 1H), 5.08 (d, *J* = 8.0 Hz, 1H), 4.94-4.87 (m, 1H), 4.16-4.08 (m, 1H), 3.65 (s, 3H), 3.29 (d, *J* = 6.0 Hz, 2H), 2.54 (t, *J* = 8.0 Hz, 2H), 2.12-1.97 (m, 1H), 1.84-1.74 (m, 1H), 1.42 (s, 9H); ^{13}C NMR (100 MHz, $CDCl_3$) δ 172.4, 171.8, 155.8, 141.0, 136.2, 128.6, 128.5, 127.5, 126.2, 123.1, 122.3, 119.7, 118.5, 111.4, 109.8, 80.2, 54.2, 52.9, 52.5, 34.1, 31.7, 28.4, 27.7; HRMS *m/z* Calcd. for $C_{27}H_{34}N_3O_5$ (M + H^+): 480.2493; Found: 480.2499.

Compound 3u (Fig. 4)

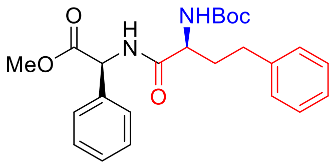

3u

White solid; Mp: 197-199 °C; $[\alpha]_D^{25}$ = 55.5 (*c* = 0.10, $CHCl_3$); ^{1}H NMR (400 MHz, $CDCl_3$) δ 7.37-7.27 (m, 7H), 7.22-7.16 (m, 3H), 7.14 (d, *J* = 7.6 Hz, 1H) 5.53 (d, *J* = 7.2 Hz, 1H), 5.07 (d, *J* = 8.4 Hz, 1H), 4.21-4.12 (m, 1H), 3.72 (s, 3H), 2.70 (t, *J* = 8.0 Hz, 2H), 2.21-2.11 (m, 1H), 1.97-1.87 (m, 1H), 1.42 (s, 9H); ^{13}C NMR (100 MHz, $CDCl_3$) δ 171.3, 171.1, 155.7, 141.0, 136.2, 129.1, 128.7, 128.63, 128.57, 127.3, 126.3, 80.3, 56.6, 54.0, 52.9, 33.9, 31.8, 28.4; HRMS *m/z* Calcd. for $C_{24}H_{31}N_2O_5$ (M + H^+): 427.2227; Found: 427.2231.

Compound (*S*,*R*)-3v (Fig. 4)

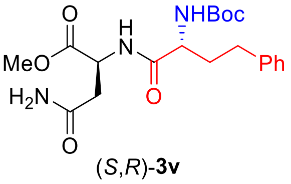

(*S*,*R*)-**3v**

White solid; Mp: 205-207 °C; $[\alpha]_D^{25}$ = -3.3 (*c* = 0.10, $CHCl_3$); ^1H NMR (400 MHz, $CDCl_3$) δ 7.75 (d, *J* = 8.0 Hz, 1H), 7.30-7.20 (m, 2H), 7.20-7.10 (m, 3H), 6.08 (s, 1H), 5.95 (s, 1H), 5.51 (d, *J* = 7.2 Hz, 1H), 4.85-4.71 (m, 1H), 4.3-4.15 (m, 1H), 3.67 (s, 3H), 2.95 (dd, *J* = 16.0, 5.2 Hz, 1H), 2.75 (dd, *J* = 16.0, 4.4 Hz, 1H), 2.67 (t, *J* = 8.0 Hz, 2H), 2.22-2.06 (m, 1H), 2.03-1.85 (m, 1H), 1.42 (s, 9H); ^{13}C NMR (100 MHz, $CDCl_3$) δ 172.6, 172.2, 171.6, 155.7, 141.2, 128.6, 128.5, 126.2, 80.2, 54.5, 52.8, 49.1, 36.9, 34.4, 31.7, 28.4; HRMS *m/z*: Calcd. for $C_{20}H_{30}N_3O_6$ (M + H^+): 408.2129; Found: 408.2127.

Compound (*S*,*S*)-3v (Fig. 4)

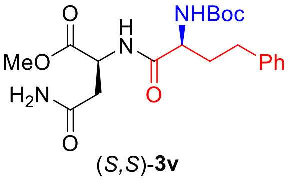

(*S*,*S*)-**3v**

White solid; Mp: 165-167 °C; $[\alpha]_D^{25}$ = -5.5 (*c* = 0.10, $CHCl_3$); ^1H NMR (400 MHz, $CDCl_3$) δ 7.73 (d, *J* = 8.0 Hz, 1H), 7.26-7.20 (m, 2H), 7.20-7.10 (m, 3H), 6.26 (s, 1H), 6.21 (s, 1H), 5.50 (d, *J* = 8.0 Hz, 1H), 4.86-4.75 (m, 1H), 4.35-4.15 (m, 1H), 3.69 (s, 3H), 2.92 (dd, *J* = 16.0, 4.4 Hz, 1H), 2.83-2.57 (m, 3H), 2.17-2.05 (m, 1H), 1.98-1.82 (m, 1H), 1.42 (s, 9H); ^{13}C NMR (100 MHz, $CDCl_3$) δ 172.7, 172.5, 171.6, 155.8, 141.2, 128.6, 128.5, 126.1, 80.1, 54.3, 52.8, 49.0, 37.1, 34.7, 31.8, 28.4; HRMS *m/z*: Calcd. for $C_{20}H_{30}N_3O_6$ (M + H^+): 408.2129; Found: 408.2129.

Compound (*S*,*R*)-3w (Fig. 4)

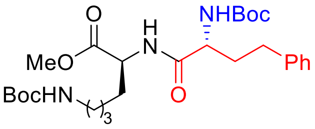

(*S*,*R*)-**3w**

Yellow solid; Mp: 48-50 °C; $[\alpha]_D^{25}$ = -16.5 (c = 0.10, $CHCl_3$); ^1H NMR (400 MHz, $CDCl_3$) δ 7.32-7.26 (m, 2H), 7.22-7.13 (m, 3H), 6.68 (d, J = 8.0 Hz, 1H), 4.98 (d, J = 7.2 Hz, 1H), 4.67-4.50 (m, 2H), 4.20-4.05 (m, 1H), 3.73 (s, 3H), 3.15-2.95 (m, 2H), 2.69 (t, J = 8.0 Hz, 2H), 2.25-2.10 (m, 1H), 1.99-1.80 (m, 2H), 1.75-1.62 (m, 2H), 1.55-1.48 (m, 1H), 1.45 (s, 9H), 1.43 (s, 9H), 1.37-1.25 (m, 2H); ^{13}C NMR (100 MHz, $CDCl_3$) δ 172.7, 171.8, 156.2, 155.8, 140.9, 128.7, 128.5, 126.3, 80.5, 79.2, 54.3, 52.6, 52.0, 40.3, 33.9, 32.1, 32.0, 29.6, 28.6, 28.4, 22.5; HRMS *m/z*: Calcd. for $C_{27}H_{44}N_3O_7$ (M + H^+): 522.3174; Found: 522.3173.

Compound (*S*,*S*)-3w (Fig. 4)

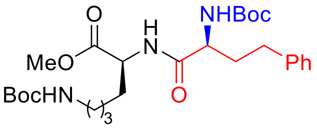

(*S*,*S*)-**3w**

Yellow solid; Mp: 35-37 °C; $[\alpha]_D^{25}$ = -36.6 (c = 0.10, $CHCl_3$); ^1H NMR (400 MHz, $CDCl_3$) δ 7.32-7.26 (m, 2H), 7.22-7.15 (m, 3H), 6.60 (d, J = 8.0 Hz, 1H), 5.17 (d, J = 8.4 Hz, 1H), 4.72 (s, 1H), 4.65-4.56 (m, 1H), 4.17-4.03 (m, 1H), 3.73 (s, 3H), 3.12-3.04 (m, 2H), 2.69 (t, J = 7.6 Hz, 2H), 2.25-2.08 (m, 1H), 1.98-1.78 (m, 2H), 1.72-1.57 (m, 1H), 1.55-1.47 (m, 2H), 1.44 (s, 9H), 1.43 (s, 9H), 1.37-1.26 (m, 2H); ^{13}C NMR (100 MHz, $CDCl_3$) δ 172.6, 171.9, 156.2, 155.8, 141.0, 128.6, 128.6, 126.3, 80.3, 79.3, 54.1, 52.6, 51.9, 40.2, 33.9, 32.0, 31.9, 29.3, 28.6, 28.4, 22.4; HRMS *m/z*: Calcd. for $C_{27}H_{44}N_3O_7$ (M + H^+): 522.3174; Found: 522.3180.

Compound (*S*,*R*)-3x (Fig. 4)

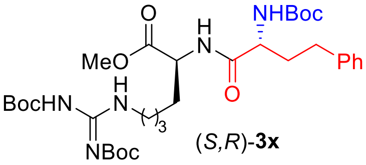

(*S*,*R*)-**3x**

Light yellow solid; Mp: 120-122 °C; $[\alpha]_D^{25}$ = -19.9 (c = 0.10, $CHCl_3$); ^1H NMR (400 MHz, $CDCl_3$) δ 11.48 (s, 1H), 8.29 (t, J = 5.6 Hz, 1H), 7.31-7.26 (m, 2H), 7.23-7.13 (m, 3H), 6.70 (s, 1H), 5.03 (s, 1H), 4.59-4.51 (m, 1H), 4.20-4.05 (m, 1H), 3.72 (s, 3H),

3.45-3.32 (m, 2H), 2.67 (t, J = 8.0 Hz, 2H), 2.22-2.10 (m, 1H), 1.97-1.80 (m, 2H), 1.76-1.65 (m, 1H), 1.63-1.53 (m, 2H), 1.49 (s, 9H), 1.47 (s, 9H), 1.44 (s, 9H), 1.41-1.32 (m, 2H); ^{13}C NMR (100 MHz, $CDCl_3$) δ 172.6, 171.8, 163.7, 156.2, 155.8, 153.4, 140.9, 128.7, 128.5, 126.3, 83.2, 80.4, 79.4, 54.1, 52.5, 52.0, 40.6, 33.9, 32.1, 32.0, 28.7, 28.4, 28.2, 22.7; HRMS *m/z*: Calcd. for $C_{33}H_{54}N_5O_9$ (M + H^+): 664.3916; Found: 664.3918.

Compound (*S*,*S*)-3x (Fig. 4)

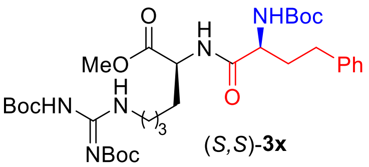

(*S*,*S*)-**3x**

Light yellow solid; Mp: 110-112 °C; $[\alpha]_D^{25}$ = -30.6 (c = 0.10, $CHCl_3$); ^{1}H NMR (400 MHz, $CDCl_3$) δ 11.49 (s, 1H), 8.28 (t, J = 5.6 Hz, 1H), 7.32-7.26 (m, 2H), 7.24-7.15 (m, 3H), 6.57 (d, J = 8.0 Hz, 1H), 5.12 (d, J = 8.4 Hz, 1H), 4.61-4.53 (m, 1H), 4.17-4.03 (m, 1H), 3.74 (s, 3H), 3.42-3.35 (m, 2H), 2.68 (t, J = 8.0 Hz, 2H), 2.23-2.10 (m, 1H), 1.97-1.78 (m, 3H), 1.75-1.63 (m, 1H), 1.62-1.52 (m, 2H), 1.49 (s, 9H), 1.48 (s, 9H), 1.44 (s, 9H) 1.41-1.34 (m, 1H); ^{13}C NMR (100 MHz, $CDCl_3$) δ 172.6, 171.8, 163.6, 156.2, 155.8, 153.4, 141.0, 128.65, 128.57, 126.3, 83.3, 80.3, 79.5, 54.1, 52.6, 52.0, 40.7, 33.8, 32.2, 31.9, 28.6, 28.4, 28.2, 22.7; HRMS *m/z*: Calcd. for $C_{33}H_{54}N_5O_9$ (M + H^+): 664.3916; Found: 664.3920.

Compound (*R*,*R*,*S*)-3y (Fig. 4)

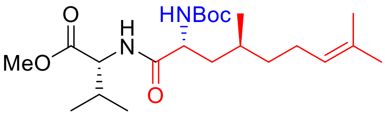

(*R*,*R*,*S*)-**3y**

Light yellow oil; $[\alpha]_D^{25}$ = -8.3 (c = 0.10, $CHCl_3$); ^{1}H NMR (400 MHz, $CDCl_3$) δ 6.70 (d, J = 9.2 Hz, 0.32H for minor amide *trans*/*cis* isomer), 6.60 (d, J = 8.8 Hz, 0.68H for major amide *trans*/*cis* isomer), 5.12-5.01 (m, 1H), 4.92-4.72 (m, 1H), 4.53 (dd, J = 8.8, 4.8 Hz, 1H), 4.22-4.00 (m, 1H), 3.72 (s, 3H), 2.25-2.10 (m, 1H), 2.05-1.90 (m, 2H), 1.67 (s, 3H), 1.63-1.48 (m, 6H), 1.44 (s, 9H), 1.34-1.16 (m, 2H), 1.00-0.85 (m, 9H);

^{13}C NMR (100 MHz, $CDCl_3$) For major amide *trans*/*cis* isomer: δ 172.67, 172.3 155.9, 131.57, 124.60, 80.3, 57.10, 53.0, 52.3, 38.8, 37.4, 31.5, 29.1, 28.4, 25.8, 25.5, 19.12, 19.05, 17.82, 17.75; For minor amide *trans*/*cis* isomer: δ 172.72, 172.4, 155.9, 131.63, 124.55, 80.3, 57.06, 53.0, 52.3, 39.1, 37.4, 31.4, 29.2, 28.4, 25.8, 25.5, 19.3, 19.1, 17.82, 17.75; HRMS *m/z*: Calcd. for $C_{22}H_{41}N_2O_5$ ($M + H^+$): 413.3010; Found: 413.3011.

Compound (*R*,*S*,*S*)-3y (Fig. 4)

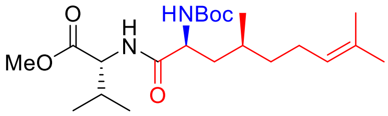

(*R*,*S*,*S*)-**3y**

Light yellow oil; $[\alpha]_D^{25}$ = -55.0 (*c* = 0.10, $CHCl_3$); ^1H NMR (400 MHz, $CDCl_3$) δ 6.68 (d, *J* = 9.2 Hz, 0.54H for major amide *trans*/*cis* isomer), 6.59 (d, *J* = 9.2 Hz, 0.46H for minor amide *trans*/*cis* isomer), 5.12-5.02 (m, 1H), 4.98-4.80 (m, 1H), 4.57-4.45 (m, 1H), 4.20-4.05 (m, 1H), 3.72 (s, 3H), 2.22-2.10 (m, 1H), 2.02-1.87 (m, 2H), 1.87-1.85 (m, 1H), 1.66 (s, 3H), 1.58 (s, 3H), 1.57-1.47 (m, 1H), 1.44 (d, *J* = 4.0 Hz, 9H), 1.42-1.35 (m, 2H), 1.18-1.07 (m, 1H), 0.96-0.85 (m, 9H); ^{13}C NMR (100 MHz, $CDCl_3$) For major amide *trans*/*cis* isomer: δ 172.5, 172.34, 155.7, 131.55, 124.55, 80.27, 57.11, 53.0, 52.3, 39.5, 36.4, 31.3, 29.33, 28.4, 25.8, 25.3, 19.8, 19.1, 17.8; For minor amide *trans*/*cis* isomer: δ 172.34, 172.26, 155.7, 131.49 , 124.61, 80.19, 57.06, 53.0, 52.2, 39.2, 36.7, 31.4, 29.26, 28.4, 25.8, 25.4, 19.8, 19.0, 17.8; HRMS *m/z*: Calcd. for $C_{22}H_{41}N_2O_5$ ($M + H^+$): 413.3010; Found: 413.3011.

5. Procedure for Asymmetric Transamination of Peptidyl α-Keto Amides Using 3z as the Representative Example (Fig. 4, 3z)

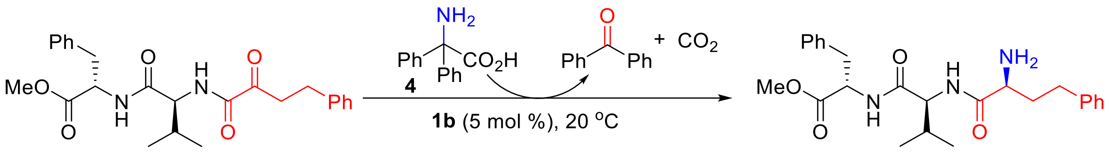

To a 2.5 mL vial equipped with a magnetic stirrer bar were added **2z** (0.0438 g, 0.10 mmol), *N*-quaternized chiral pyridoxamine (*R*)-**1b** (0.0026 g, 0.0050 mmol), 2,2-diphenylglycine **4** (0.0250 g, 0.11 mmol), CH_3COOH (0.024 g, 0.40 mmol), Na_2HPO_4 (0.0282 g, 0.20 mmol), MeOH (0.48 mL), and H_2O (0.12 mL). After the mixture was stirred at 20 °C for 72 h, the reaction mixture was concentrated via rotary evaporator to remove most of the solvent. The residue was purified by flash column chromatography on silica gel (DCM : MeOH = 30:1) to give product **3z** (0.0339 g, 77%) as a pale yellow oil.

The products **3aa-ac** were prepared by a similar procedure unless otherwise stated. For compound **3ab**, the reactions were carried out in a double scale; For compound **3ac**, a mixture of CF_3CH_2OH (0.40 mL), THF (0.2 mL), and H_2O (0.10 mL) was used as the solvent.

The dr value of product **3z** were determined by HPLC analysis after being converted to the corresponding *N*-Boc protected derivative by treatment with *di*-tert-butyl dicarbonate (3.0 equiv.) at room temperature for 3 h. The dr values of **3aa** and **3ac** were determined by HPLC analysis after the products were converted to the corresponding *N*-benzoyl derivatives by treatment with benzoyl chloride (1.5 equiv.) and $NaHCO_3$ (2.0 equiv.) in THF at room temperature for 0.5 h. The dr values of **3ab** were determined by HPLC analysis after the products were converted to the corresponding derivatives by treatment with 3,5-di-*tert*-butylbenzoic acid (2.5 equiv.),

EDCl (2.0 equiv.), HOBt (2.0 equiv.) and Et_3N (3.0 equiv.) in DCM at room temperature for 2 h. For the synthetic procedure of compound **3ad**, see section 7.1 (Page S52).

3z: White solid; Mp: 157-159 °C; $[\alpha]_D^{25}$ = 18.4 (*c* = 0.10, $CHCl_3$); ^1H NMR (400 MHz, $CDCl_3$) δ 7.73 (d, *J* = 8.8 Hz, 1H), 7.38-7.15 (m, 8H), 7.09 (d, *J* = 6.8 Hz, 2H), 6.63 (d, *J* = 8.0 Hz, 1H), 4.88-4.81 (m, 1H), 4.22 (dd, *J* = 8.8, 6.4 Hz, 1H), 3.68 (s, 3H), 3.44 (dd, *J* = 8.0, 4.8 Hz, 1H), 3.20-3.04 (m, 4H), 2.78-2.61 (m, 2H), 2.21-2.07 (m, 2H), 1.82-1.71 (m, 1H), 0.92 (d, *J* = 6.8 Hz, 3H), 0.90 (d, *J* = 6.8 Hz, 3H); ^{13}C NMR (100 MHz, $CDCl_3$) δ 174.9, 171.9, 171.1, 141.1, 135.8, 129.3, 128.7, 128.6, 128.5, 127.2, 126.3, 58.6, 54.6, 53.3, 52.5, 37.9, 36.6, 32.2, 30.7, 19.4, 18.2; HRMS *m/z* Calcd. for $C_{25}H_{34}N_3O_4$ (M + H^+): 440.2544; Found: 440.2544.

Compound (*S*,*S*,*S*)-3aa (Fig. 4)

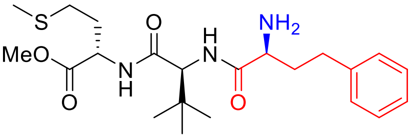

(*S*,*S*,*S*)-**3aa**

Yellow oil; $[\alpha]_D^{25}$ = -32.0 (*c* = 0.10, $CHCl_3$); ^1H NMR (400 MHz, $CDCl_3$) δ 8.01 (d, *J* = 9.6 Hz, 1H), 7.31-7.26 (m, 2H), 7.23-7.15 (m, 3H), 6.60 (d, *J* = 7.6 Hz, 1H), 4.71-4.65 (m, 1H), 4.23 (d, *J* = 9.6 Hz, 1H), 3.75 (s, 3H), 3.42 (dd, *J* = 8.0, 4.4 Hz, 1H), 2.80-2.65 (m, 2H), 2.49 (t, *J* = 7.2 Hz, 2H), 2.25-1.92 (m, 6H), 1.89-1.67 (m, 3H), 1.03 (s, 9H); ^{13}C NMR (100 MHz, $CDCl_3$) δ 175.0, 172.2, 170.6, 141.3, 128.7, 128.6, 126.3, 60.6, 55.1, 52.6, 51.7, 37.1, 34.7, 32.4, 31.5, 30.1, 26.8, 15.6; HRMS *m/z* Calcd. for $C_{22}H_{35}N_3NaO_4S$ (M + Na^+): 460.2240; Found: 460.2245.

Compound (*S*,*S*,*R*)-3aa (Fig. 4)

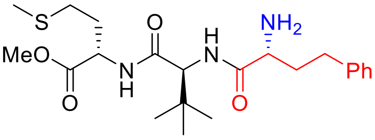

(*S*,*S*,*R*)-**3aa**

Light yellow oil; $[\alpha]_D^{25}$ = -21.1 (c = 0.10, $CHCl_3$); ^{1}H NMR (400 MHz, $CDCl_3$) δ 7.86 (d, J = 9.2 Hz, 1H), 7.30-7.23 (m, 2H), 7.22-7.14 (m, 3H), 6.78 (d, J = 8.0 Hz, 1H), 4.72-4.50 (m, 1H), 4.24 (d, J = 9.2 Hz, 1H), 3.73 (s, 3H), 3.41 (dd, J = 8.4, 4.4 Hz, 1H), 2.80-2.62 (m, 2H), 2.47 (t, J = 7.4 Hz, 2H), 2.25-2.07 (m, 2H), 2.06-1.91 (m, 1H), 2.02 (s, 3H), 1.86-1.67 (m, 3H), 1.02 (s, 9H); ^{13}C NMR (100 MHz, $CDCl_3$) δ 175.0, 172.3, 170.6, 141.2, 128.61, 128.55, 126.2, 60.7, 54.8, 52.6, 51.6, 36.7, 34.6, 32.2, 31.5, 30.0, 26.8, 15.5； HRMS m/z: Calcd. for $C_{22}H_{35}N_3NaO_4S$ (M + Na^+): 460.2240; Found: 460.2237.

Compound (*R*,*S*,*R*)-3ab (Fig. 4)

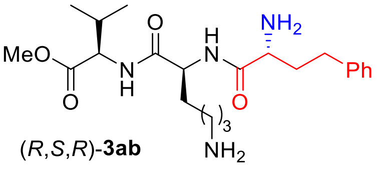

Light yellow oil; $[\alpha]_D^{25}$ = -38.0 (c = 0.10, $CHCl_3$); ^{1}H NMR (400 MHz, $CDCl_3$) δ 7.82 (d, J = 8.4 Hz, 1H), 7.33-7.22 (m, 2H), 7.22-7.14 (m, 4H), 4.54-4.39 (m, 2H), 3.65 (s, 3H), 3.36 (dd, J = 8.4, 4.4 Hz, 1H), 2.90-2.55 (m, 8H), 2.25-2.07 (m, 2H), 1.98-1.85 (m, 1H), 1.85-1.72 (m, 1H), 1.72-1.60 (m, 1H), 1.58-1.46 (m, 2H), 1.45-1.32 (m, 2H), 0.92 (d, J = 6.8 Hz, 3H), 0.89 (d, J = 6.8 Hz, 3H); ^{13}C NMR (100 MHz, $CDCl_3$) δ 175.7, 172.4, 172.0, 141.2, 128.6, 128.5, 126.2, 57.4, 54.8, 52.6, 52.2, 41.4, 36.7, 32.2, 32.0, 31.6, 31.1, 22.9, 19.2, 17.9; HRMS m/z: Calcd. for $C_{22}H_{37}N_4O_4$ (M + H^+): 421.2809; Found: 421.2810.

Compound (*R*,*S*,*S*)-3ab (Fig. 4)

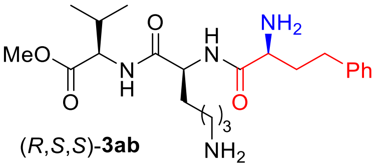

Light yellow oil; $[\alpha]_D^{25}$ = -27.0 (c = 0.10, $CHCl_3$); ^{1}H NMR (400 MHz, $CDCl_3$) δ 7.89 (d, J = 8.0 Hz, 1H), 7.49 (d, J = 8.4 Hz, 1H), 7.28-7.20 (m, 2H), 7.20-7.11 (m, 3H), 4.57-4.47 (m, 1H), 4.47-4.35 (m, 1H), 4.12-3.72 (m, 4H, $2NH_2$), 3.65 (s, 3H), 3.39 (t,

J = 6.4 Hz, 1H), 2.76 (t, J = 7.4, 2H), 2.72-2.57 (m, 2H), 2.22-2.02 (m, 2H), 1.90-1.71 (m, 2H), 1.70-1.47 (m, 3H), 1.45-1.30 (m, 2H), 0.91 (d, J = 6.4 Hz, 3H), 0.88 (d, J = 8.0 Hz, 3H); ^{13}C NMR (100 MHz, $CDCl_3$) δ 175.8, 172.7, 172.2, 141.2, 128.6, 128.5, 126.2, 57.5, 54.8, 52.6, 52.2, 40.5, 36.8, 32.2, 31.9, 30.9, 29.9, 22.6, 19.2, 18.0; HRMS *m/z*: Calcd. for $C_{22}H_{37}N_4O_4$ (M + H^+): 421.2809; Found: 421.2812.

Compound 3ac (Fig. 4)

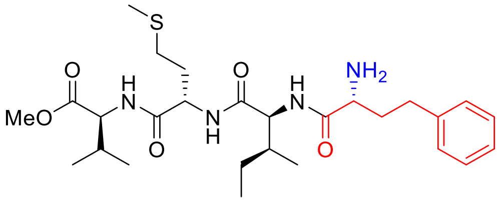

3ac

White solid; Mp: 230-232 °C; $[\alpha]_D^{25}$ = -19.8 (c = 0.10, $CHCl_3$); ^{1}H NMR (400 MHz, CD_3OD) δ 7.30-7.24 (m, 2H), 7.22-7.14 (m, 3H), 4.58 (dd, J = 8.4, 6.0 Hz, 1H), 4.32 (d, J = 6.0 Hz, 1H), 4.27 (d, J = 7.6 Hz, 1H), 3.72 (s, 3H), 3.59 (t, J = 6.4 Hz, 1H), 2.66 (t, J = 8.4 Hz, 2H), 2.62-2.46 (m, 2H), 2.21-1.80 (m, 9H), 1.61-1.50 (m, 1H), 1.27-1.15 (m, 1H), 0.98-0.86 (m, 12H); ^{13}C NMR (100 MHz, CD_3OD) δ 175.5, 173.71, 173.69, 173.4, 142.5, 129.6, 129.3, 127.2, 59.2, 59.1, 55.3, 53.6, 52.5, 38.0, 37.3, 32.9, 32.6, 31.7, 30.9, 26.0, 19.5, 18.5, 16.0, 15.2, 11.3; HRMS *m/z* Calcd. for $C_{27}H_{44}N_4NaO_5S$ (M + Na^+): 559.2925; Found: 559.2931.

6. Representative Procedure for Divergent Extension of Ubenimex Methyl Ester (Fig. 5a, 3ae).

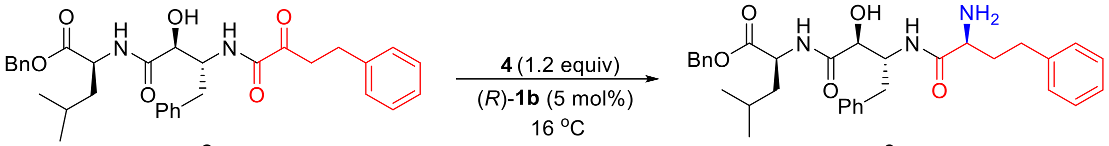

2ae **3ae**

To a 2.5 mL vial equipped with a magnetic stirrer bar were added **2ae** (0.0279 g, 0.050 mmol), *N*-quaternized chiral pyridoxamine (*R*)-**1b** (0.0013 g, 0.00250 mmol), 2,2-diphenylglycine (**4**) (0.0136 g, 0.060 mmol), CH_3COOH (0.012 g, 0.20 mmol), KOAc (0.00980 g, 0.10 mmol), MeOH (0.20 mL), DCM (0.10 mL) and H_2O (0.050 mL). After being stirred at 16 °C for 60 h, the reaction mixture was concentrated via rotary evaporator to remove most of the solvent. The residue was purified by flash column chromatography on silica gel (DCM : MeOH = 10:1) to give product **3ae** (0.0218 g, 78%) as a white solid.

The products **3af-ai** were prepared by the same procedure unless otherwise stated. For **3ag**, a mixture of CF_3CH_2OH (0.20 mL), THF (0.10 mL), and H_2O (0.05 mL) was used as the solvent. For **3ah**, a mixture of CF_3CH_2OH (0.40 mL), THF (0.20 mL), and H_2O (0.10 mL) was used as the solvent. For **3ai**, the reaction was carried out in a double scale in MeOH (0.40 mL), THF (0.10 mL), DCM (0.10 mL), and H_2O (0.10 mL). The dr values of products for **3ae-ai** were determined by HPLC analysis after the products were converted to the corresponding *N*-benzoyl derivatives by treatment with benzoyl chloride (1.5 equiv.) and $NaHCO_3$ (2.0 equiv.) in THF at room temperature for 0.5 h.

3ae: White solid; Mp: 198-200 °C; $[\alpha]_D^{25}$ = 5.5 (*c* = 0.10, $CHCl_3$); ^1H NMR (600 MHz, CD_3OD) δ 7.36-7.28 (m, 5H), 7.27-7.20 (m, 6H), 7.16-7.08 (m, 4H), 5.17 (d, *J* = 12.0 Hz, 1H), 5.12 (d, *J* = 12.0 Hz, 1H), 4.55-4.47 (m, 2H), 4.10 (d, *J* = 3.0 Hz, 1H), 3.20 (dd, *J* = 7.2, 5.4 Hz, 1H), 3.00 (dd, *J* = 13.8, 6.6 Hz, 1H), 2.76 (dd, *J* = 13.8, 9.0 Hz, 1H), 2.50-2.42 (m, 1H), 2.40-2.32 (m, 1H), 1.82-1.72 (m, 1H), 1.67-1.55 (m, 4H), 0.87 (d, *J* = 6.0 Hz, 3H), 0.85 (d, *J* = 6.6 Hz, 3H); ^{13}C NMR (150 MHz, $CDCl_3$) δ 175.4, 173.0, 172.9, 140.7, 138.0, 135.4, 129.3, 128.7, 128.6, 128.52, 128.47, 128.3, 126.8,

126.3, 73.3, 67.3, 55.2, 54.2, 50.9, 40.8, 35.7, 35.5, 31.8, 24.9, 23.0, 21.6; HRMS *m/z* Calcd. for $C_{33}H_{41}N_3NaO_5$ (M + Na$^+$): 582.2938; Found: 582.2940.

Compound 3af (Fig. 5a)

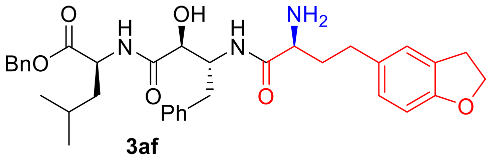

3af

Light yellow solid; Mp: 139-141 °C; $[\alpha]_D^{25}$ = 3.8 (*c* = 0.10, $CHCl_3$); ^1H NMR (600 MHz, $CDCl_3$) δ 7.83 (d, *J* = 8.4 Hz, 1H), 7.51 (d, *J* = 8.4 Hz, 1H), 7.37-7.30 (m, 5H), 7.26-7.15 (m, 5H), 6.94 (s, 1H), 6.81 (d, *J* = 9.0 Hz, 1H), 6.67 (d, *J* = 7.8 Hz, 1H), 5.16-5.07 (m, 2H), 4.65-4.57 (m, 1H), 4.52 (t, *J* = 8.4 Hz, 2H), 4.34-4.28 (m, 1H), 4.23 (d, *J* = 3.6 Hz, 0.85H for major isomer), 4.18 (d, *J* = 3.6 Hz, 0.15H for minor isomer), 3.28-3.22 (m, 1H), 3.17-3.08 (m, 3H), 2.99 (t, *J* = 12.3 Hz, 1H), 2.58-2.38 (m, 2H), 2.00 (s, 2H), 1.65-1.45 (m, 3H), 0.88-0.76 (m, 6H); ^{13}C NMR (100 MHz, $CDCl_3$) δ 172.82, 172.77, 172.4, 158.6, 138.0, 135.4, 132.6, 129.4, 128.74, 128.66, 128.5, 128.3, 127.9, 127.4, 126.8, 125.0, 109.2, 73.8, 71.3, 67.3, 55.7, 54.2, 50.8, 40.9, 36.3, 35.6, 31.4, 29.9, 24.9, 23.0, 21.6； HRMS *m/z* Calcd. for $C_{35}H_{44}N_3O_6$ (M + H$^+$): 602.3225; Found: 602.3233.

Compound 3ag (Fig. 5a)

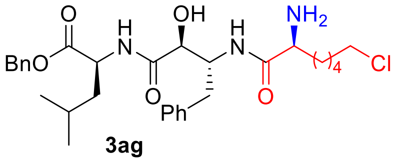

3ag

White solid; Mp: 125-127 °C; $[\alpha]_D^{25}$ = 2.5 (*c* = 0.10, $CHCl_3$); ^1H NMR (400 MHz, CD_3OD) δ 7.38-7.13 (m, 10H), 5.18 (d, *J* = 12.0 Hz, 1H), 5.15 (d, *J* = 12.0 Hz, 1H), 4.61-4.42 (m, 2H), 4.11 (d, *J* = 3.2 Hz, 1H), 3.51 (t, *J* = 6.8 Hz, 2H), 3.24 (t, *J* = 6.8 Hz, 1H), 3.01 (dd, *J* = 13.6, 6.0 Hz, 1H), 2.71 (dd, *J* = 13.6, 9.6 Hz, 1H), 1.80-1.56 (m, 5H), 1.55-1.41 (m, 1H), 1.40-1.23 (m, 3H), 1.17-0.98 (m, 2H), 0.94 (d, *J* = 6.0 Hz, 3H), 0.91 (d, *J* = 6.0 Hz, 3H); ^{13}C NMR (100 MHz, CD_3OD) δ 176.0, 174.9, 173.8, 139.5, 137.2,

130.4, 129.6, 129.5, 129.4, 129.3, 127.6, 72.9, 68.0, 55.7, 54.8, 52.1, 45.6, 41.4, 37.8, 35.3, 33.5, 27.8, 26.0, 25.5, 23.2, 22.0; HRMS *m/z* Calcd. for $C_{30}H_{42}ClN_3NaO_5$ (M + Na^+): 582.2705; Found: 582.2708.

Compound 3ah (Fig. 5a)

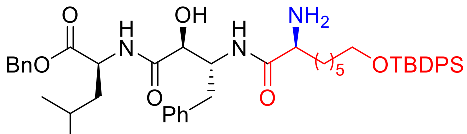

3ah

Colorless oil; $[\alpha]_D^{25}$ = -6.3 (*c* = 0.10, $CHCl_3$); ^{1}H NMR (400 MHz, $CDCl_3$) δ 7.87 (d, *J* = 7.6 Hz, 1H), 7.70-7.62 (m, 4H), 7.46-7.29 (m, 12H), 7.27-7.17 (m, 5H), 5.15 (s, 2H), 4.62 (td, *J* = 11.6, 5.2 Hz, 1H), 4.30-4.14 (m, 2H), 3.65 (t, *J* = 6.8 Hz, 2H), 3.20-2.95 (m, 3H), 1.77-1.44 (m, 7H), 1.42-1.17 (m, 6H), 1.05 (s, 9H), 0.90 (d, *J* = 6.4 Hz, 3H), 0.89 (d, *J* = 6.4 Hz, 3H); ^{13}C NMR (100 MHz, $CDCl_3$) δ 177.9, 172.9, 172.4, 138.0, 135.7, 135.5, 134.2, 129.6, 129.4, 128.7, 128.6, 128.5, 128.3, 127.7, 126.8, 74.2, 67.1, 64.0, 56.0, 55.0, 50.6, 41.1, 35.7, 34.8, 32.6, 29.2, 27.0, 26.1, 25.8, 24.9, 23.1, 21.7, 19.3; HRMS *m/z* Calcd. for $C_{47}H_{64}N_3O_6Si$ (M + H^+): 794.4559; Found: 794.4552.

Compound 3ai (Fig. 5a)

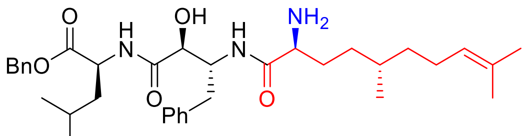

3ai

Yellow solid; Mp: 125-127 °C; $[\alpha]_D^{25}$ = -3.1 (*c* = 0.10, $CHCl_3$); ^{1}H NMR (400 MHz, $CDCl_3$) δ 7.81 (d, *J* = 7.2 Hz, 1H), 7.35-7.10 (m, 11H), 5.07 (s, 2H), 5.01 (t, *J* = 7.2 Hz, 1H), 4.57-4.50 (m, 1H), 4.20-4.10 (m, 2H), 3.10-3.00 (m, 3H), 1.96-1.68 (m, 3H), 1.61 (s, 3H), 1.58-1.40 (m, 6H), 1.36-0.98 (m, 8H), 0.85-0.75 (m, 9H); ^{13}C NMR (150 MHz, $CDCl_3$) δ 175.6, 173.0, 172.9, 138.1, 135.5, 131.3, 129.3, 128.7, 128.6, 128.5, 128.3, 126.7, 124.8, 73.4, 67.3, 55.3, 55.1, 50.9, 40.8, 36.8, 35.4, 32.9, 32.5, 31.7, 25.8, 25.6, 24.9, 23.0, 21.6, 19.4, 17.8; HRMS *m/z* Calcd. for $C_{35}H_{51}N_3NaO_5$ (M + Na^+): 616.3721; Found: 616.3726.

7. Procedures for Synthesis of Peptides via Successive Transamination (Fig. 5b)

7.1 Synthesis of Compound 3ad

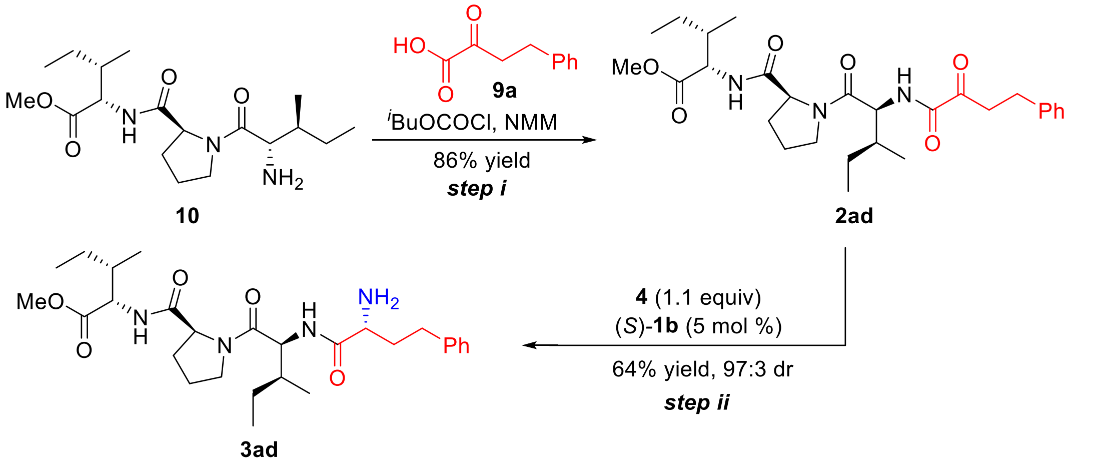

***Step i*. Synthesis of compound 2ad**[5]

To a 50 mL round-bottom flask were added compound **9a** (0.602 g, 3.38 mmol). The sealed flask was evacuated and refilled with N_2 for three times, followed by addition of isobutyl chloroformate (^{i}BuOCOCl) (0.462 g, 3.38 mmol), *N*-methylmorpholine (NMM) (0.342 g, 3.38 mmol) and anhydrous THF (10 mL) via a syringe at -10 °C. After stirring for 10 min, a solution of compound **10** (1.00 g, 2.81 mmol) in anhydrous THF (5 mL) was added via a syringe at -20 °C. Then the reaction mixture was allowed to warm to room temperature and stirred for 1 h. The reaction mixture was concentrated via rotary evaporator to remove most of the solvent, and the residue was quenched by addition of H_2O (40 mL). The resulting mixture was extracted with ethyl acetate (30 mL × 3). The combined organic layers were washed with brine, dried over Na_2SO_4, filtered, concentrated and purified by column chromatography on silica gel (petroleum ether : ethyl acetate = 2:1) to give compound **2ad** (1.24 g, 86%) as a yellow oil.

2ad: yellow oil; $[\alpha]_D^{25}$ = -69.0 (c = 0.10, $CHCl_3$); ^{1}H NMR (600 MHz, $CDCl_3$) δ 7.46 (d, J = 9.6 Hz, 1H), 7.23-7.08 (m, 6H), 4.51-4.41 (m, 3H), 3.72-3.67 (m, 1H), 3.66

(s, 3H), 3.59-3.55 (m, 1H), 3.23-3.10 (m, 2H), 2.86 (t, J = 7.8 Hz, 2H), 2.28-2.23 (m, 1H), 2.12-2.04 (m, 1H), 1.95-1.75 (m, 4H), 1.50-1.43 (m, 1H), 1.40-1.32 (m, 1H), 1.15-1.01 (m, 2H), 0.90 (d, J = 6.6 Hz, 3H), 0.86-0.78 (m, 9H); ^{13}C NMR (150 MHz, $CDCl_3$) δ 197.3, 172.2, 171.2, 170.7, 159.9, 140.4, 128.6, 128.5, 126.4, 60.2, 56.9, 55.1, 52.2, 48.0, 38.6, 37.9, 37.8, 29.2, 27.5, 25.2, 24.5, 15.54, 15.47, 11.7, 11.1; HRMS *m/z*: Calcd. for $C_{28}H_{42}N_3O_6$ (M + H^+): 516.3068; Found: 516.3077.

***Step ii*. Synthesis of compound 3ad**

To a 50 mL round-bottom flask were added keto amide **2ad** (1.2 g, 2.33 mmol), *N*-quaternized chiral pyridoxamine (*S*)-**1b** (0.061 g, 0.117 mmol), 2,2-diphenylglycine (**4**) (0.635 g, 2.80 mmol) and KOAc (0.458 g, 4.66 mmol). The sealed flask was evacuated and refilled with N_2 three times. Then CH_3COOH (0.560 g, 9.32 mmol), MeOH (5.0 mL), DCM (2.5 mL) and H_2O (1.25 mL) were added and the reaction mixture was stirred at 16 °C for 120 h. The solvent was removed by rotary evaporation and the residue was purified by flash column chromatography on silica gel (DCM : MeOH : 2.9 M ammonia solution in ethanol = 20:1:0.42) to give compound **3ad** with 0.770 g (64% yield) of the major diastereomer as a yellow oil and 25 mg of the minor diastereomer as a yellow oil. The dr value were determined as 97:3 by the ratio of the weights of the two diastereomers.

3ad: Yellow oil; $[\alpha]_D^{25}$ = -34.9 (c = 0.10, $CHCl_3$); ^{1}H NMR (600 MHz, $CDCl_3$) δ 7.64 (d, J = 9.0 Hz, 1H), 7.30-7.24 (m, 3H), 7.22-7.16 (m, 3H), 4.64-4.55 (m, 2H), 4.49 (dd, J = 8.4, 4.8 Hz, 1H), 3.88-3.79 (m, 1H), 3.71 (s, 3H), 3.67-3.57 (m, 1H), 3.36 (dd, J = 8.4, 4.2 Hz, 1H), 2.76-2.64 (m, 2H), 2.36-2.30 (m, 1H), 2.22-2.06 (m, 2H), 2.00-1.74 (m, 7H), 1.61-1.52 (m, 1H), 1.46-1.36 (m, 1H), 1.22-1.09 (m, 2H), 0.96 (d, J = 6.6 Hz, 3H), 0.91-0.86 (m, 9H); ^{13}C NMR (150 MHz, $CDCl_3$) δ 175.0, 172.5, 172.2, 170.9, 141.2, 128.6, 128.5, 126.2, 60.1, 56.9, 54.9, 54.6, 52.1, 48.0, 37.8, 37.6, 36.9, 32.2, 27.3, 25.3, 25.2, 24.6, 15.53, 15.52, 11.7, 11.1; HRMS *m/z* Calcd. for $C_{28}H_{45}N_4O_5$ (M + H^+): 517.3384; Found: 517.3388.

7.2 Synthesis of Compound 3aj

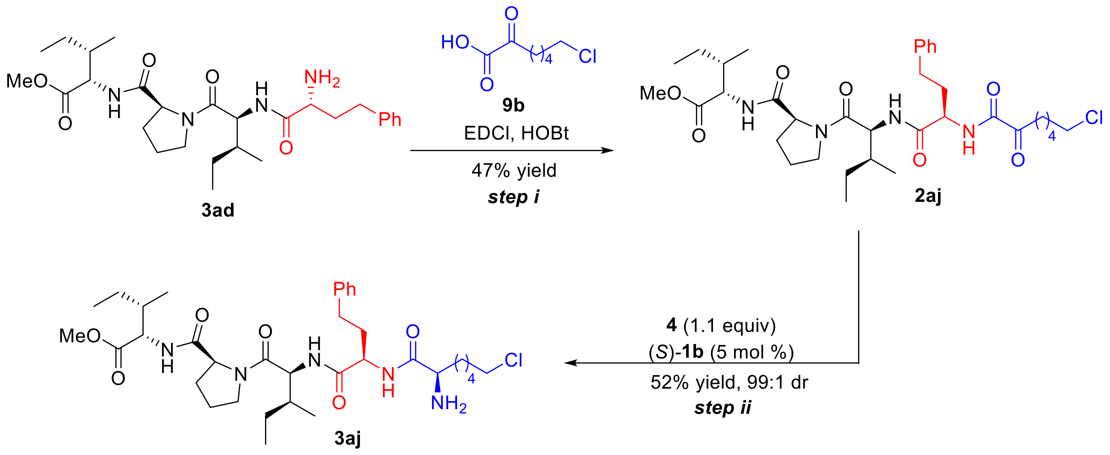

Step i. Synthesis of compound 2aj[4]

To a 50 mL round-bottom flask were added compound **9b** (0.569 g, 3.20 mmol), **3ad** (1.1 g, 2.13 mmol), 1-(3-dimethylaminopropyl)-3-ethylcarbodiimide hydrocholoride (EDCl) (0.817 g, 4.26 mmol), 1-hydroxybenzotriazole (HOBt) (0.575 g, 4.26 mmol), Et_3N (0.647 g, 6.39 mmol), and anhydrous THF (15 mL). The reaction mixture was stirred at room temperature under N_2 atmosphere overnight and then the reaction was quenched by addition of H_2O (40 mL). The resulting mixture was extracted with ethyl acetate (30 mL × 3). The combined organic layers were washed with brine, dried over Na_2SO_4, filtered, concentrated in vacuo and purified by column chromatography on silica gel (petroleum ether : ethyl acetate = 2:1) to give compound **2aj** (0.670 g, 47%) as a yellow oil.

2aj: Yellow oil; $[\alpha]_D^{25}$ = -71.3 (*c* = 0.10, $CHCl_3$); ^1H NMR (400 MHz, $CDCl_3$) δ 7.52 (d, *J* = 8.4 Hz, 1H), 7.22-7.04 (m, 6H), 6.93 (d, *J* = 8.8 Hz, 1H), 4.60-4.39 (m, 4H), 3.78-3.71 (m, 1H), 3.64 (s, 3H), 3.61-3.55 (m, 1H), 3.46 (t, *J* = 6.4 Hz, 2H), 2.83 (t, *J* = 7.2 Hz, 2H), 2.63-2.50 (m, 2H), 2.23-2.03 (m, 3H), 1.99-1.68 (m, 7H), 1.62-1.25 (m, 6H), 1.14-1.01 (m, 2H), 0.90 (d, *J* = 6.8 Hz, 3H), 0.83-0.76 (m, 9H); ^{13}C NMR (150 MHz, $CDCl_3$) δ 198.1, 172.3, 171.7, 171.0, 170.5, 159.8, 140.7, 128.7, 128.4, 126.4, 60.0, 56.8, 55.0, 53.0, 52.1, 48.0, 44.8, 37.84, 37.79, 36.7, 34.7, 32.4, 31.6, 27.9, 26.4, 25.4, 25.0, 24.6, 22.5, 15.5, 15.4, 11.7, 11.0; HRMS *m/z*: Calcd. for $C_{35}H_{54}ClN_4O_7$ ($M + H^+$): 677.3676; Found: 677.3688.

***Step ii*. Synthesis of compound 3aj**

To a 50 mL round-bottom flask were added keto amide **2aj** (0.670 g, 0.991 mmol), *N*-quaternized chiral pyridoxamine (*S*)-**1b** (0.0259 g, 0.0496 mmol), 2,2-diphenylglycine **4** (0.269 g, 1.09 mmol) and KOAc (0.194 g, 1.98 mmol). The sealed flask was evacuated and refilled with N_2 three times. Then CH_3COOH (0.238 g, 3.96 mmol), MeOH (6.0 mL), DCM (1.5 mL), THF (1.5 mL) and H_2O (1.5 mL) were added and the reaction mixture was stirred at 18 °C for 120 h. Then the solvent was removed via rotary evaporator under reduced pressure, and the residue was submitted to flash column chromatography on silica gel (DCM : MeOH : 2.9 M ammonia solution in ethanol = 30:1:0.62) (Note: Before loading sample, the silica gel was rinsed with a mixed solvent (petroleum ether : DCM : 2.9 M ammonia solution in ethanol = 1:1:0.04) to give compound **3aj** (0.350 g, 52%) as a yellow solid. The dr value was determined by HPLC analysis after the product was converted to the corresponding N-protected derivative by treatment with 3,5-di-*tert*-butylbenzoic acid (1.5 equiv.), EDCl (2.0 equiv.), HOBt (2.0 equiv.) and Et_3N (3.0 equiv.) in THF at room temperature for 2 h.

3aj: Yellow solid; Mp: 115-117 °C; $[\alpha]_D^{25}$ = -48.0 (*c* = 0.10, $CHCl_3$); ^{1}H NMR (400 MHz, $CDCl_3$) δ 7.85 (d, *J* = 8.0 Hz, 1H), 7.36 (d, *J* = 8.0 Hz, 1H), 7.32-7.24 (m, 2H), 7.23-7.12 (m, 4H), 4.68-4.58 (m, 3H), 4.45 (dd, *J* = 8.4, 4.8 Hz 1H), 3.87-3.77 (m, 1H), 3.71 (s, 3H), 3.67-3.60 (m, 1H), 3.52 (t, *J* = 6.8 Hz 2H), 3.38 (dd, *J* = 8.0, 4.4 Hz, 1H), 2.72-2.59 (m, 2H), 2.35-1.69 (m, 13H), 1.66-1.33 (m, 7H), 1.23-1.09 (m, 2H), 0.98 (d, *J* = 6.8 Hz, 3H), 0.94-0.80 (m, 9H); ^{13}C NMR (100 MHz, $CDCl_3$) δ 175.3, 172.2, 171.9, 171.6, 171.1, 141.2, 128.6, 128.5, 126.2, 60.0, 56.8, 55.1, 55.0, 52.5, 52.1, 48.0, 45.0, 37.7, 34.9, 34.5, 32.4, 31.8, 27.7, 26.8, 26.3, 25.4, 25.2, 25.0, 24.5, 15.6, 15.4, 11.7, 11.1; HRMS *m/z*: Calcd. for $C_{35}H_{57}ClN_5O_6$ (M + H^+): 678.3992; Found: 678.4003.

7.3 Synthesis of Compound 3ak

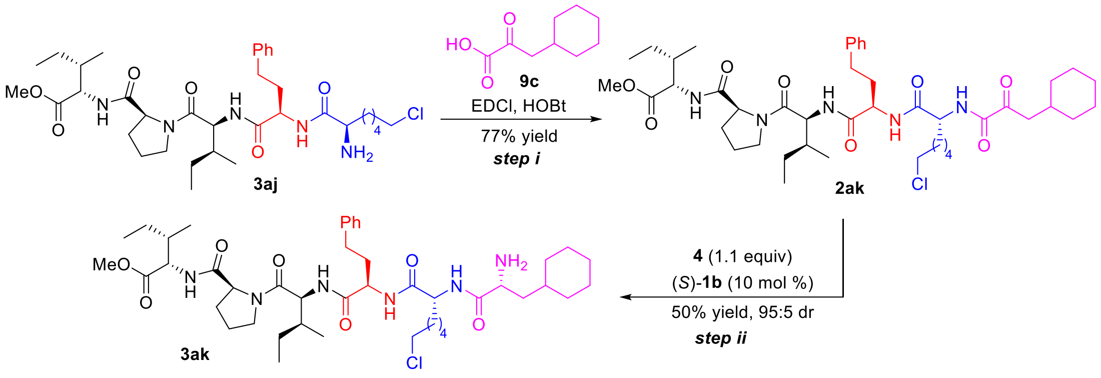

Step i. Synthesis of compound 2ak[4]

To a 50 mL round-bottom flask were added compound **9c** (0.128 g, 0.753 mmol), **3aj** (0.340 g, 0.502 mmol), 1-(3-dimethylaminopropyl)-3-ethylcarbodiimide hydrochloride (EDCl) (0.212 g, 1.10 mmol), 1-hydroxybenzotriazole (HOBt) (0.149 g, 1.10 mmol), Et_3N (0.150 mL, 1.51 mmol), and anhydrous THF (10 mL). The reaction mixture was stirred at room temperature under N_2 atmosphere overnight. The reaction was quenched by addition of H_2O (50 mL), and the resulting mixture was extracted with ethyl acetate (20 mL × 3). The combined organic layers were washed with brine, dried over Na_2SO_4, filtered, concentrated and purified by column chromatography on silica gel (petroleum ether : ethyl acetate = 1:1) to give compound **2ak** (0.320 g, 77%) as a yellow solid.

2ak: yellow solid; Mp: 81-83 °C; $[\alpha]_D^{25}$ = -45.8 (*c* = 0.10, $CHCl_3$); ^1H NMR (400 MHz, $CDCl_3$) δ 7.83 (d, *J* = 8.4 Hz, 1H), 7.47 (d, *J* = 8.0 Hz,, 1H), 7.29-7.10 (m, 6H), 7.03 (d, *J* = 8.0 Hz, 1H), 4.74-4.67 (m, 2H), 4.58 (t, *J* = 8.4 Hz, 1H), 4.50 (dd, *J* = 8.4, 4.8 Hz, 1H), 4.46-4.39 (m, 1H), 3.85-3.79 (m, 1H), 3.68 (s, 3H), 3.67-3.62 (m, 1H), 3.48 (t, *J* = 6.6 Hz, 2H), 2.84 (dd, *J* = 17.2, 6.8 Hz, 1H), 2.73 (dd, *J* = 16.8, 6.8 Hz, 1H), 2.66-2.56 (m, 2H), 2.31-2.11 (m, 3H), 2.02-1.54 (m, 17H), 1.48-1.07 (m, 9H), 1.00-0.95 (m, 5H), 0.89-0.83 (m, 9H); ^{13}C NMR (100 MHz, $CDCl_3$) δ 198.5, 172.1, 171.9, 171.3, 171.0, 170.5, 160.8, 141.2, 128.6, 128.4, 126.2, 59.9, 56.6, 54.9, 52.9, 52.8, 52.1, 48.1, 44.9, 44.3, 37.8, 37.7, 35.2, 33.6, 33.29, 33.26, 32.3, 31.9, 31.6, 27.9, 26.7, 26.2,

26.1, 25.3, 25.1, 25.0, 24.6, 15.45, 15.39, 11.6, 10.9; HRMS *m/z*: Calcd. for $C_{44}H_{69}ClN_5O_8$ (M + H^+): 830.4829; Found: 830.4833.

***Step ii*. Synthesis of compound 3ak**

To a 2.5 mL vial equipped with a magnetic stirrer bar were added **2ak** (0.030 g, 0.0362 mmol), *N*-quaternized chiral pyridoxamine (*S*)-**1b** (0.00189 g, 0.00362 mmol), 2,2-diphenylglycine (**4**) (0.0098 g, 0.0398 mmol), CH_3COOH (0.0087 g, 0.145 mmol), KOAc (0.00709 g, 0.0723 mmol), MeOH (0.3 mL), THF (0.075 mL) and H_2O (0.075 mL). The mixture was stirred at 25 °C for 72 h. Then the solvent was removed via rotary evaporator under reduced pressure, and the residue was submitted to flash column chromatography on silica gel (DCM : MeOH : 2.9 M ammonia solution in ethanol = 10:1:0.22. Note: Before loading sample, the silica gel was rinsed with mixed solvents (petroleum ether : DCM : 2.9 M ammonia solution in ethanol = 1:1:0.04) to give compound **3ak** (0.015 g, 50%) as a yellow solid. The dr value was determined by HPLC analysis after the product was converted to the corresponding N-protected derivative by treatment with 3,5-dimethylbenzoic chloride (1.5 equiv.), $NaHCO_3$ (2.0 equiv.), in THF (0.2 mL) and DCM (0.2 mL) at room temperature for 0.5 h.

3ak: yellow solid; Mp: 89-91 °C; $[\alpha]_D^{25}$ = -35.1 (*c* = 0.10, $CHCl_3$); ^{1}H NMR (400 MHz, $CDCl_3$) δ 7.79 (d, *J* = 8.0 Hz, 1H), 7.31-7.21 (m, 3H), 7.20-7.10 (m, 3H), 7.02 (d, *J* = 7.6 Hz, 1H), 6.96 (d, *J* = 8.8 Hz, 1H), 4.63-4.51 (m, 3H), 4.47 (dd, *J* = 8.0, 4.8 Hz, 1H), 4.39-4.31 (m, 1H), 3.83-3.76 (m, 1H), 3.71 (s, 3H), 3.65-3.55 (m, 1H), 3.52-3.44 (m, 3H), 2.67-2.55 (m, 2H), 2.35-1.50 (m, 21H), 1.49-1.05 (m, 12H), 1.03-0.80 (m, 14H); ^{13}C NMR (100 MHz, $CDCl_3$) δ 176.5, 172.3, 172.0, 171.7, 171.1, 171.0, 141.1, 128.6, 128.5, 126.3, 60.1, 56.8, 55.1, 53.1, 53.0, 52.9, 52.1, 48.0, 45.0, 42.7, 37.8, 37.7, 34.44, 34.35, 34.2, 32.4, 32.2, 31.84, 31.77, 27.6, 26.6, 26.5, 26.4, 26.2, 25.4, 25.1, 24.6, 15.53, 15.46, 11.7, 11.1; HRMS *m/z*: Calcd. for $C_{44}H_{72}ClN_6O_7$ (M + H^+): 831.5146; Found: 831.5140.

8. Procedure for Comparison of Catalyts (Fig. 6b)

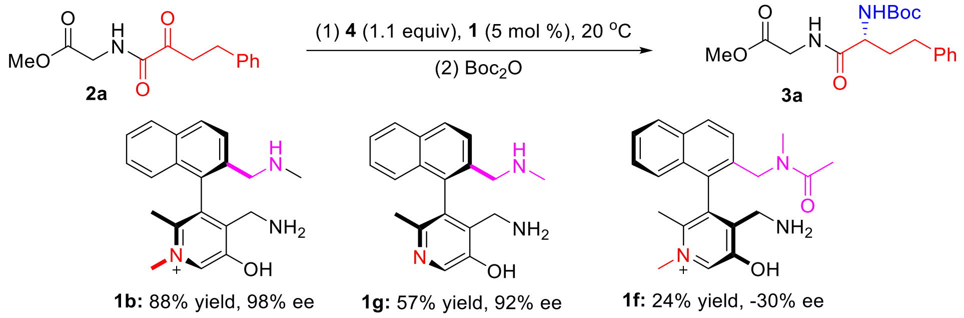

To a 2.5 mL vial equipped with a magnetic stirrer bar were added α-keto amide **2a** (0.0249 g, 0.10 mmol), chiral pyridoxamine (*S*)-**1b** or **1g** (0.0050 mmol), 2,2-diphenylglycine (**4**) (0.0250 g, 0.11 mmol), CH_3COOH (0.024 g, 0.40 mmol), Na_2HPO_4 (0.0284 g, 0.20 mmol), CF_3CH_2OH (0.48 mL), and H_2O (0.12 mL). After the suspension mixture was stirred at 20 °C for 72 h, MeOH (1.0 mL) was added to dissolve the solid completely, followed by addition of di-*tert*-butyl dicarbonate (Boc_2O) (0.0655 g, 0.30 mmol) at room temperature. Upon stirring for 3 h, the reaction mixture was concentrated via rotary evaporator to remove most of the solvent. The residue was submitted to flash column chromatography on silica gel (petroleum ether : ethyl acetate = 3:1) to give product **3a** as a white solid. For **1f**, the reaction was carried out in a double scale. The enantiomeric excesses of products were determined by HPLC analysis.

9. Determination of the Absolute Configurations of Transamination Products

9.1 Determination the Absolute Configuration of Transamination Product 3d

The single crystal of transamination product **3d** was obtained by recrystallization from a mixed solvent of *n*-hexane and dichloromethane.

The absolute configuration of compound **3d** obtained by (*S*)-**1b**-catalyzed transamination was determined as *R* by X-ray analysis (Supplementary Figure 1).

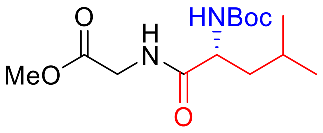

3d

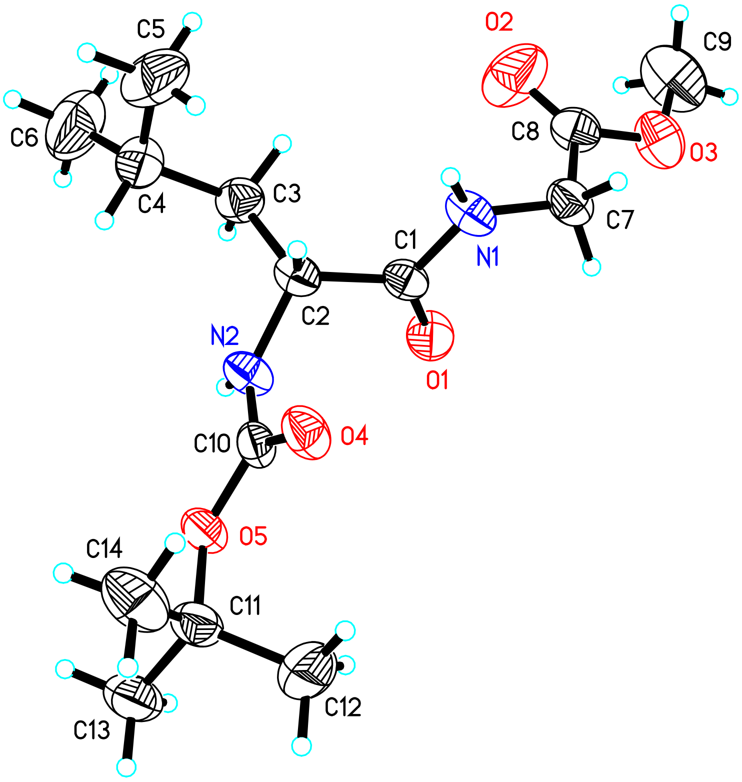

Supplementary Figure 1. X-ray structure of compound 3d

Supplementary Table 1. Crystal Data and Structure Refinement for (*R*)-3d (CCDC 2036531)

Identification code	cu_dd20064_0m	
Empirical formula	$C_{14}H_{26}N_2O_5$	
Formula weight	302.37	
Temperature	293(2) K	
Wavelength	1.54178 Å	
Crystal system	Triclinic	
Space group	P 1	
Unit cell dimensions	a = 11.0471(3) Å	α = 73.3290(10)°.
	b = 11.9018(3) Å	β = 78.478(2)°.
	c = 14.4845(4) Å	γ = 88.1220(10)°.
Volume	1787.01(8) $Å^3$	
Z	4	
Density (calculated)	1.124 Mg/m^3	
Absorption coefficient	0.703 mm^{-1}	
F(000)	656	
Crystal size	0.200 x 0.140 x 0.110 mm^3	
Theta range for data collection	3.250 to 67.491°.	
Index ranges	-13<=h<=13, -14<=k<=14, -17<=l<=17	
Reflections collected	69877	
Independent reflections	12613 [R(int) = 0.0530]	
Completeness to theta = 67.679°	98.4 %	
Absorption correction	Semi-empirical from equivalents	
Max. and min. transmission	0.7533 and 0.3793	
Refinement method	Full-matrix least-squares on F^2	
Data / restraints / parameters	12613 / 51 / 834	
Goodness-of-fit on F^2	1.025	
Final R indices [I>2sigma(I)]	R1 = 0.0480, wR2 = 0.1292	
R indices (all data)	R1 = 0.0530, wR2 = 0.1355	
Absolute structure parameter	0.07(5)	
Extinction coefficient	0.0137(18)	
Largest diff. peak and hole	0.561 and -0.204 $e.Å^{-3}$	

9.2 Determination the Absolute Configuration of Transamination Product 3m

The absolute configuration of the newly-generated chiral carbon for transamination product **3m** catalyzed by (*R*)-**1b** was determined to be *S* by X-ray analysis of its cyclized derivative (*S*,*S*)-**3m-cyclized** (Supplementary Figure 2). Compound (*S*,*S*)-**3m-cyclized** was prepared by the following procedure.

9.2.1 Synthesis of compound (*S*,*S*)-3m-cyclized

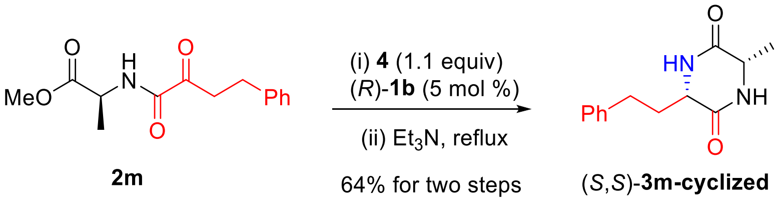

To a 5 mL vial equipped with a magnetic stirrer bar were added α-keto amide **2m** (0.0526 g, 0.20 mmol), *N*-quaternized chiral pyridoxamine (*R*)-**1b** (0.0052 g, 0.010 mmol), 2,2-diphenylglycine (**4**) (0.050 g, 0.22 mmol), CH_3COOH (0.048 g, 0.80 mmol), Na_2HPO_4 (0.0564 g, 0.40 mmol), MeOH (0.80 mL), and H_2O (0.20 mL). After the mixture was stirred at 20 °C for 72 h, the reaction mixture was concentrated via rotary evaporator to remove most of the solvent. The residue was submitted to flash column chromatography on silica gel [DCM : MeOH : 2.9 M ammonia solution in ethanol = 20:1:0.42. Note: Before loading sample, the silica gel was rinsed with a mixed solvent (petroleum ether : DCM : 2.9 M ammonia solution in ethanol = 1:1:0.04)] to give NH_2-free transamination product (0.080 g, 76%) as a light yellow oil. A small amount of the NH_2-free transamination product underwent cyclization to form compound (*S*,*S*)**-3m-cyclized** during the isolation.

The mixture of the NH_2-free transamination product (0.080 g, 0.303 mmol) and Et_3N (0.0610 g, 0.606 mmol) in MeOH (8.0 mL) was stirred at 80 °C for 24 h. The reaction mixture was filtered and the solid was washed with cold MeOH and petroleum ether. The solid was collected and dried in vacuo to give product (*S*,*S*)-**3m-cyclized** (0.059 g, 84%) as a white solid.

(S,S)-**3m-cyclized**: White solid; Mp: 330-332 °C; $[\alpha]_D^{25}$ = -59.6 (c = 0.10, DMSO); ^{1}H NMR (400 MHz, DMSO-d_6) δ 8.27 (d, J = 1.6 Hz, 1H), 8.21 (d, J = 2.0 Hz, 1H), 7.32-7.22 (m, 2H), 7.22-7.13 (m, 3H), 3.95-3.85 (m, 2H), 2.67-2.53 (m, 2H), 2.03-1.85 (m, 2H), 1.28 (d, J = 6.8 Hz, 3H); ^{13}C NMR (100 MHz, DMSO-d_6) δ 169.7, 168.3, 141.6, 128.8, 128.7, 126.4, 54.1, 50.2, 34.9, 30.6, 19.8; HRMS m/z Calcd. for $C_{13}H_{17}N_2O_2$ (M + H$^+$): 233.1285; Found: 233.1285.

9.2.2 X-ray Analysis of Compound (*S*,*S*)-3m-cyclized

The single crystal of compound (*S*,*S*)-**3m-cyclized** was obtained by recrystallization from a mixed solvent of *n*-hexane, methanol and dichloromethane.

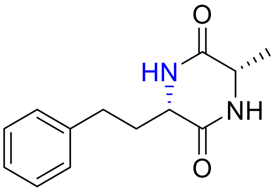

(*S*,*S*)-**3m-cyclized**

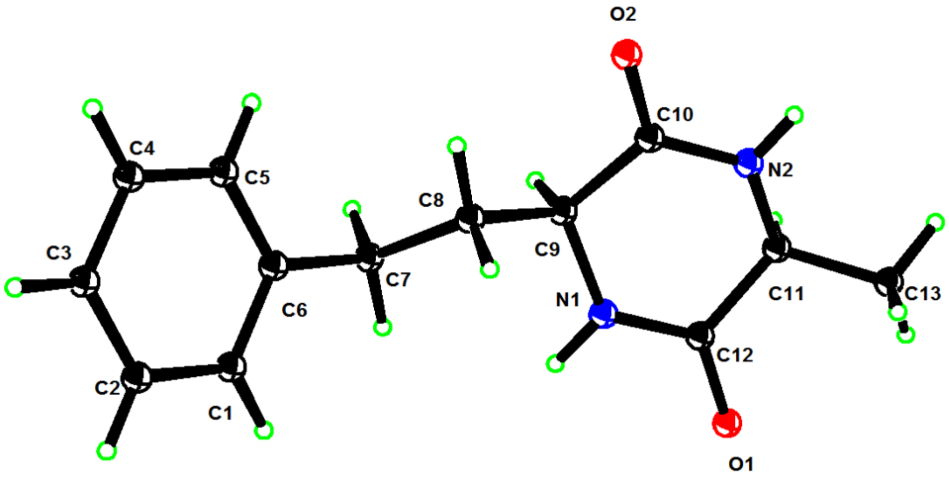

Supplementary Figure 2. X-ray structure of compound (*S*,*S*)-3m-cyclized

Supplementary Table 2. Crystal Data and Structure Refinement for (*S*,*S*)-3m-cyclized (CCDC 2036532)

Identification code	220191059_0m
Empirical formula	$C_{13}H_{16}N_2O_2$
Formula weight	232.28
Temperature/K	170.0
Crystal system	monoclinic
Space group	P21
a/Å	6.1618(3)
b/Å	25.4642(15)
c/Å	7.4864(4)
α/°	90
β/°	94.111(3)
γ/°	90
Volume/Å3	1171.63(11)
Z	4
ρcalcg/cm3	1.317
μ/mm-1	0.728
F(000)	496.0
Crystal size/mm3	0.11 × 0.08 × 0.05
Radiation	CuKα (λ = 1.54178)
2Θ range for data collection/°	6.942 to 148.644
Index ranges	-7 ≤ h ≤ 7, -31 ≤ k ≤ 31, -9 ≤ l ≤ 8
Reflections collected	16611
Independent reflections	4669 [Rint = 0.0478, Rsigma = 0.0442]
Data/restraints/parameters	4669/1/325
Goodness-of-fit on F2	1.056
Final R indexes [I>=2σ (I)]	R1 = 0.0388, wR2 = 0.1015
Final R indexes [all data]	R1 = 0.0408, wR2 = 0.1033
Largest diff. peak/hole / e Å-3	0.19/-0.19
Flack parameter	0.05(12)

9.3 Determination the Absolute Configuration of Transamination Compound (*S*,*R*)-3r

The single crystal of (*S*)-**1b**-catalyzed transamination product (*S*,*R*)-**3r** was obtained by recrystallization from a mixed solvent of *n*-hexane and ethyl acetate.

The absolute configuration of the newly-generated chiral carbon for the transamination product (*S*,*R*)-**3r** was determined as *R* by X-ray analysis (Supplementary Figure 3).

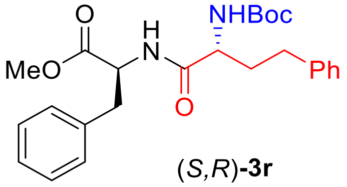

(*S*,*R*)-**3r**

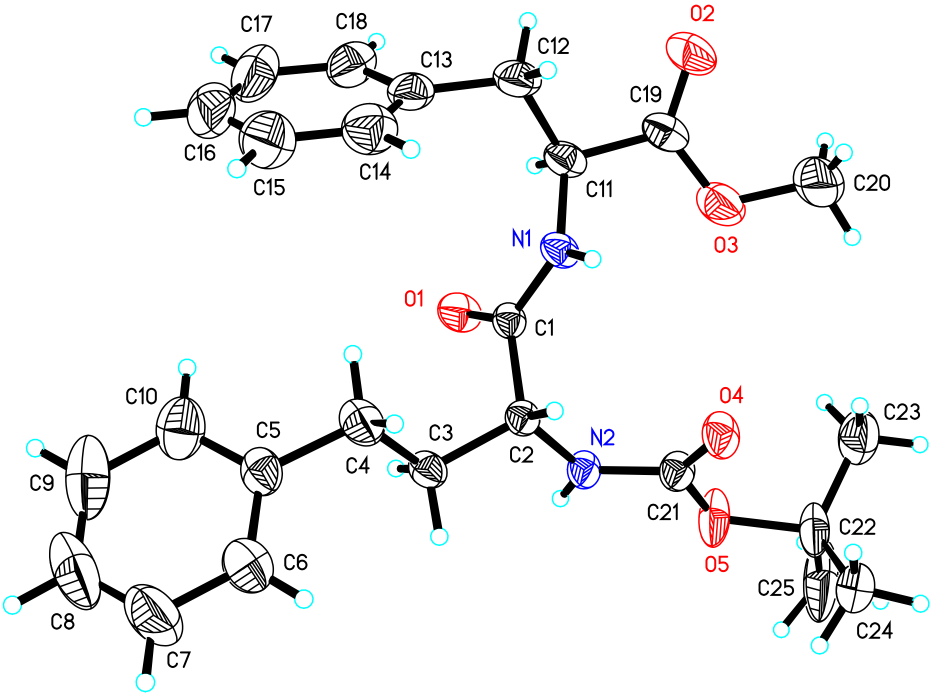

Supplementary Figure 3. X-ray structure of compound (*S*,*R*)-3r

Supplementary Table 3. Crystal Data and Structure Refinement for Compound (*S*,*R*)-3r (CCDC 2036529)

Identification code	cu_d8v20504_0m	
Empirical formula	$C_{25}H_{32}N_2O_5$	
Formula weight	440.52	
Temperature	293(2) K	
Wavelength	1.54178 Å	
Crystal system	Monoclinic	
Space group	P 21	
Unit cell dimensions	a = 5.1625(2) Å	α = 90°.
	b = 17.9141(6) Å	β = 91.927(2)°.
	c = 13.4076(5) Å	γ = 90°.
Volume	1239.25(8) $Å^3$	
Z	2	
Density (calculated)	1.181 Mg/m^3	
Absorption coefficient	0.667 mm^{-1}	
F(000)	472	
Crystal size	0.160 x 0.100 x 0.060 mm^3	
Theta range for data collection	4.937 to 67.485°.	
Index ranges	-6<=h<=5, -21<=k<=21, -16<=l<=16	
Reflections collected	15052	
Independent reflections	4367 [R(int) = 0.0624]	
Completeness to theta = 67.679°	98.0 %	
Absorption correction	Semi-empirical from equivalents	
Max. and min. transmission	0.7533 and 0.3422	
Refinement method	Full-matrix least-squares on F^2	
Data / restraints / parameters	4367 / 1 / 294	
Goodness-of-fit on F^2	1.041	
Final R indices [I>2sigma(I)]	R1 = 0.0475, wR2 = 0.1242	
R indices (all data)	R1 = 0.0511, wR2 = 0.1290	
Absolute structure parameter	0.05(15)	
Extinction coefficient	0.022(6)	
Largest diff. peak and hole	0.122 and -0.140 e.$Å^{-3}$	

10.NMR Spectra

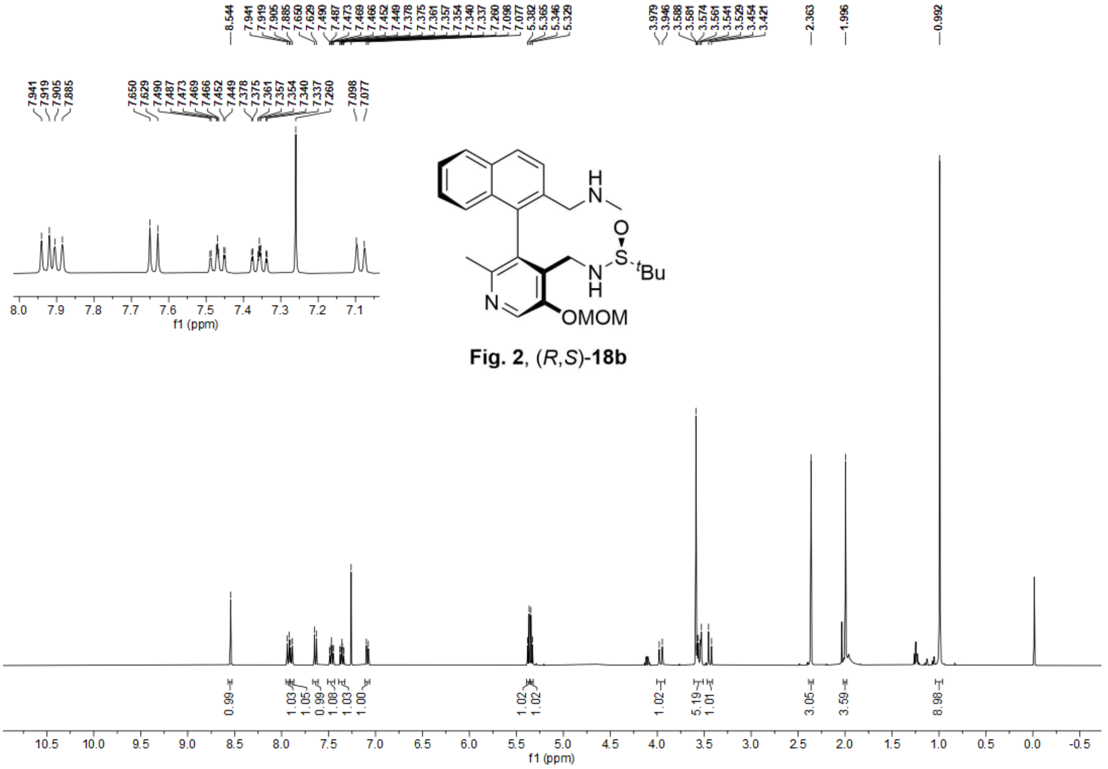

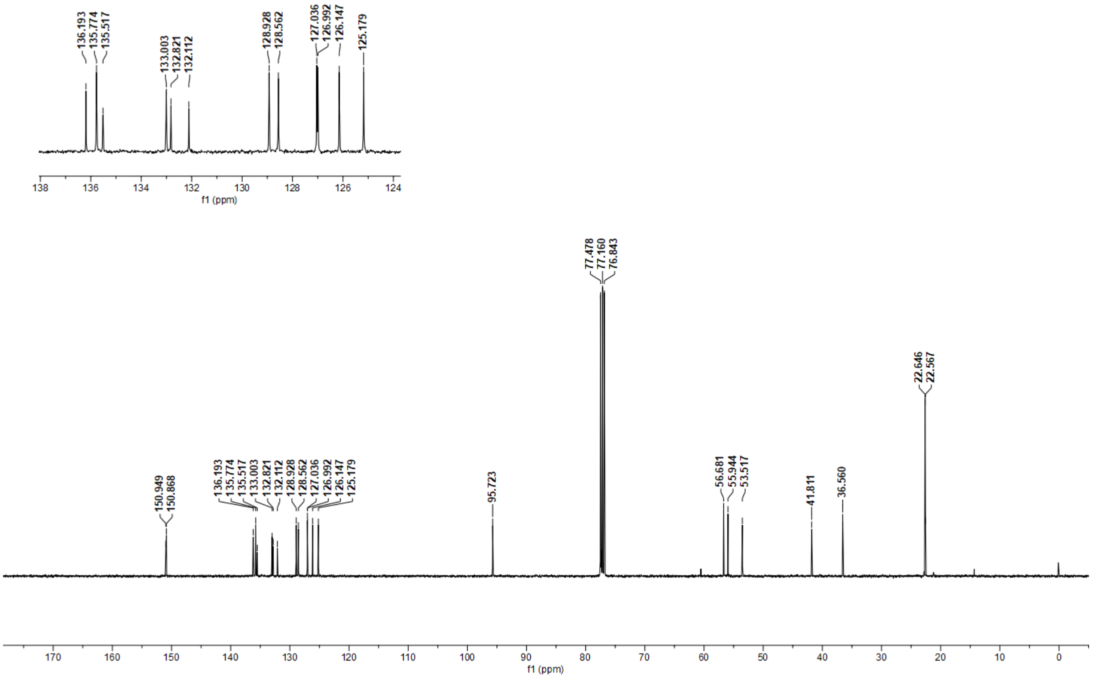

Supplementary Figure 4. ^{1}H NMR and ^{13}C NMR spectra of compound (*R*,*S*)**-18b**

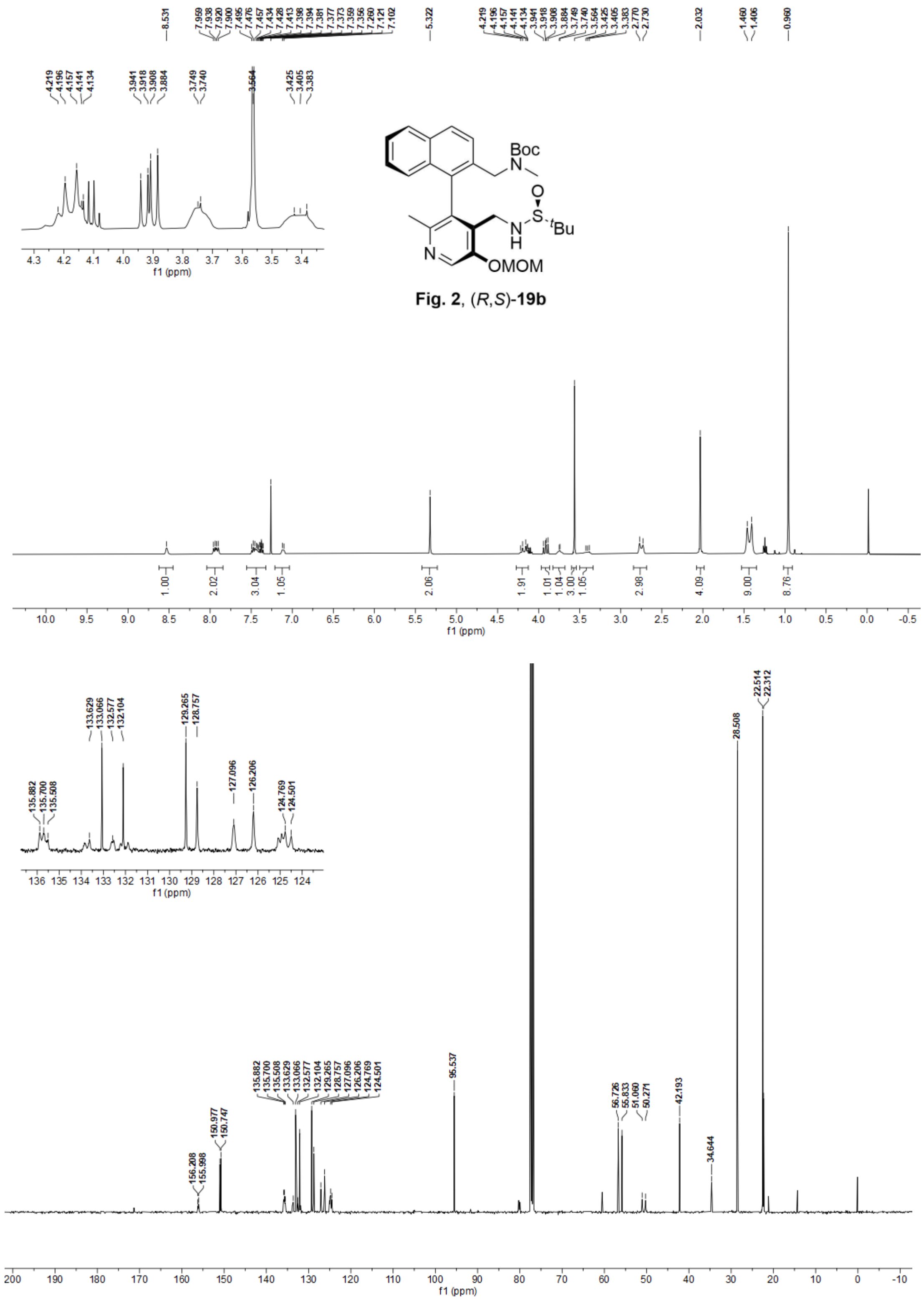

Supplementary Figure 5. ^{1}H NMR and ^{13}C NMR spectra of compound (*R*,*S*)**-19b**

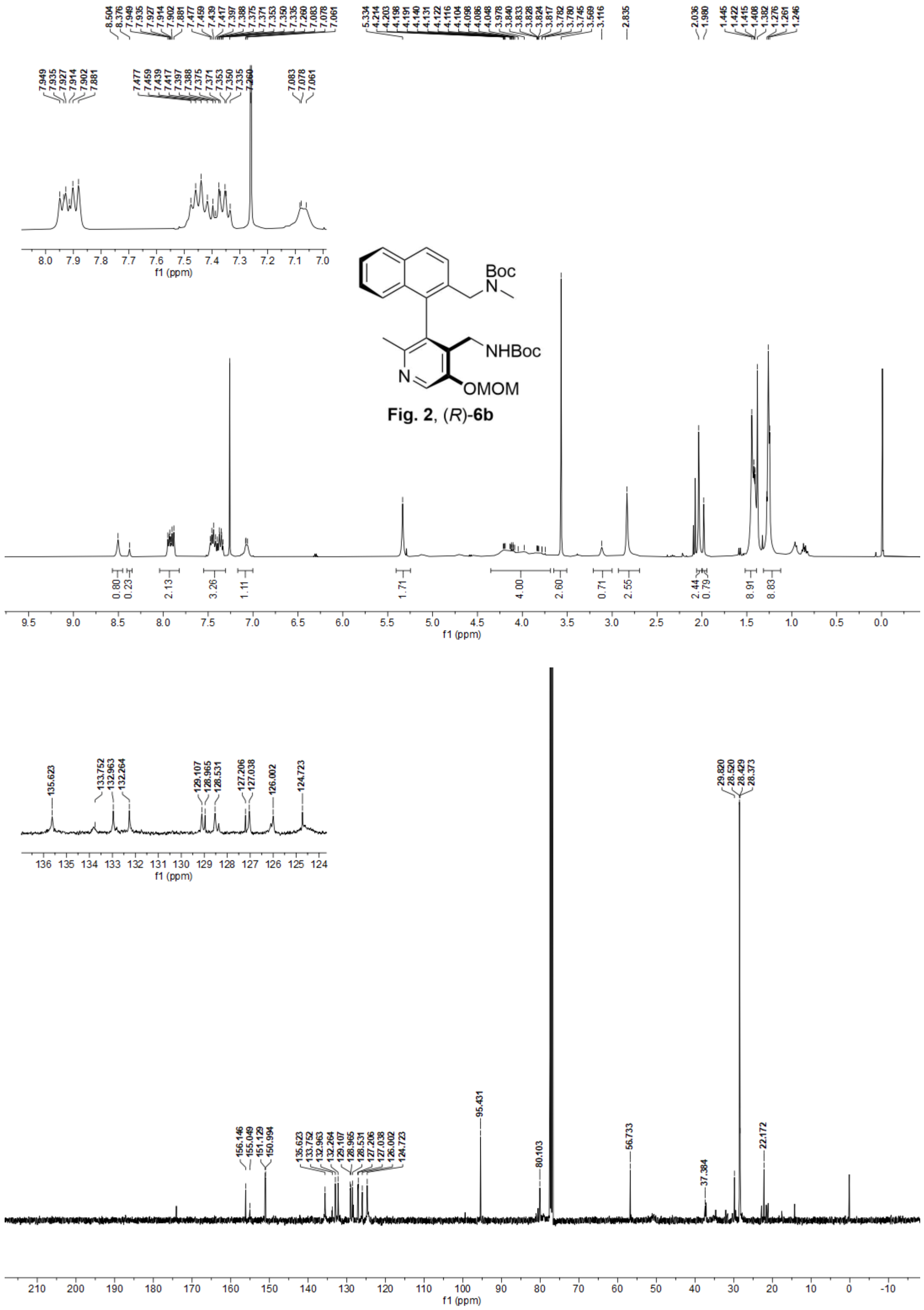

Supplementary Figure 6. ^{1}H NMR and ^{13}C NMR spectra of compound (*R*)**-6b**

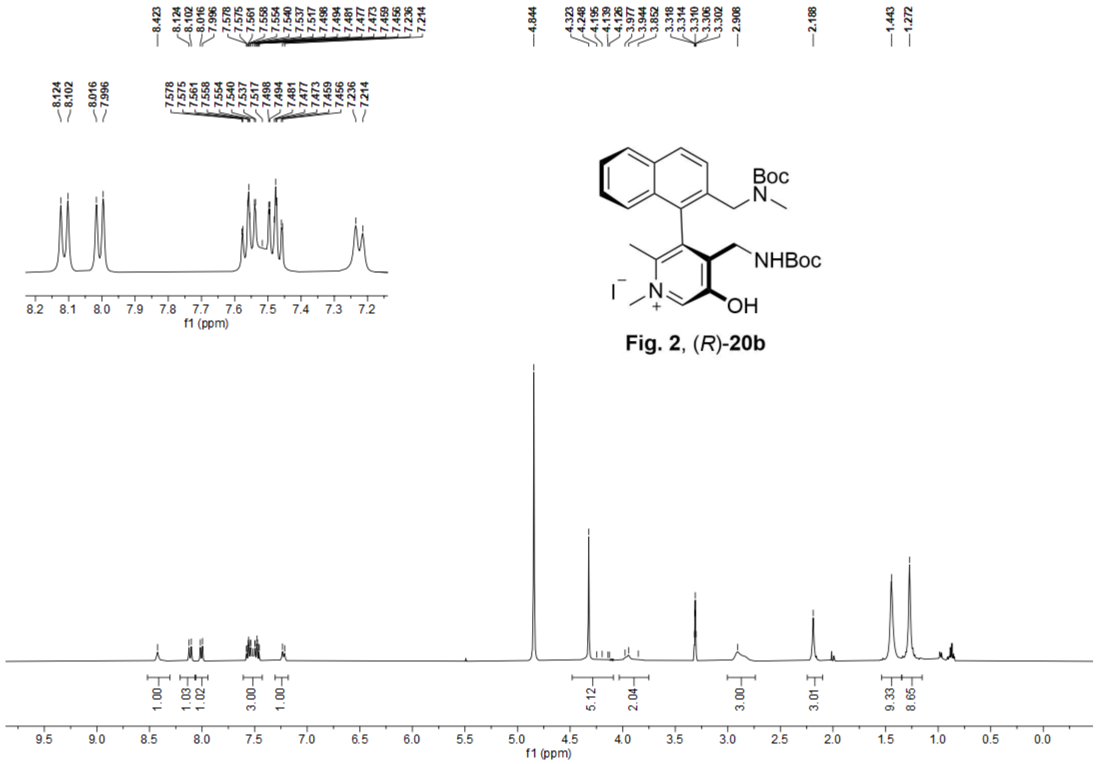

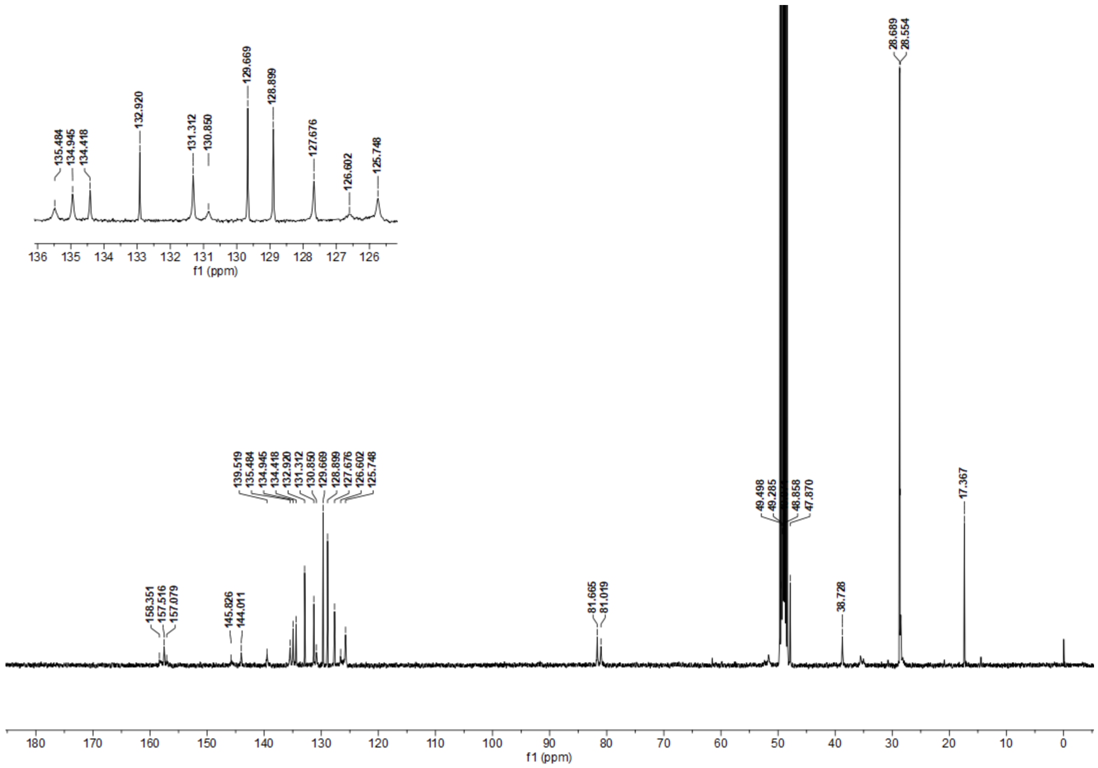

Supplementary Figure 7. ^{1}H NMR and ^{13}C NMR spectra of compound (*R*)**-20b**

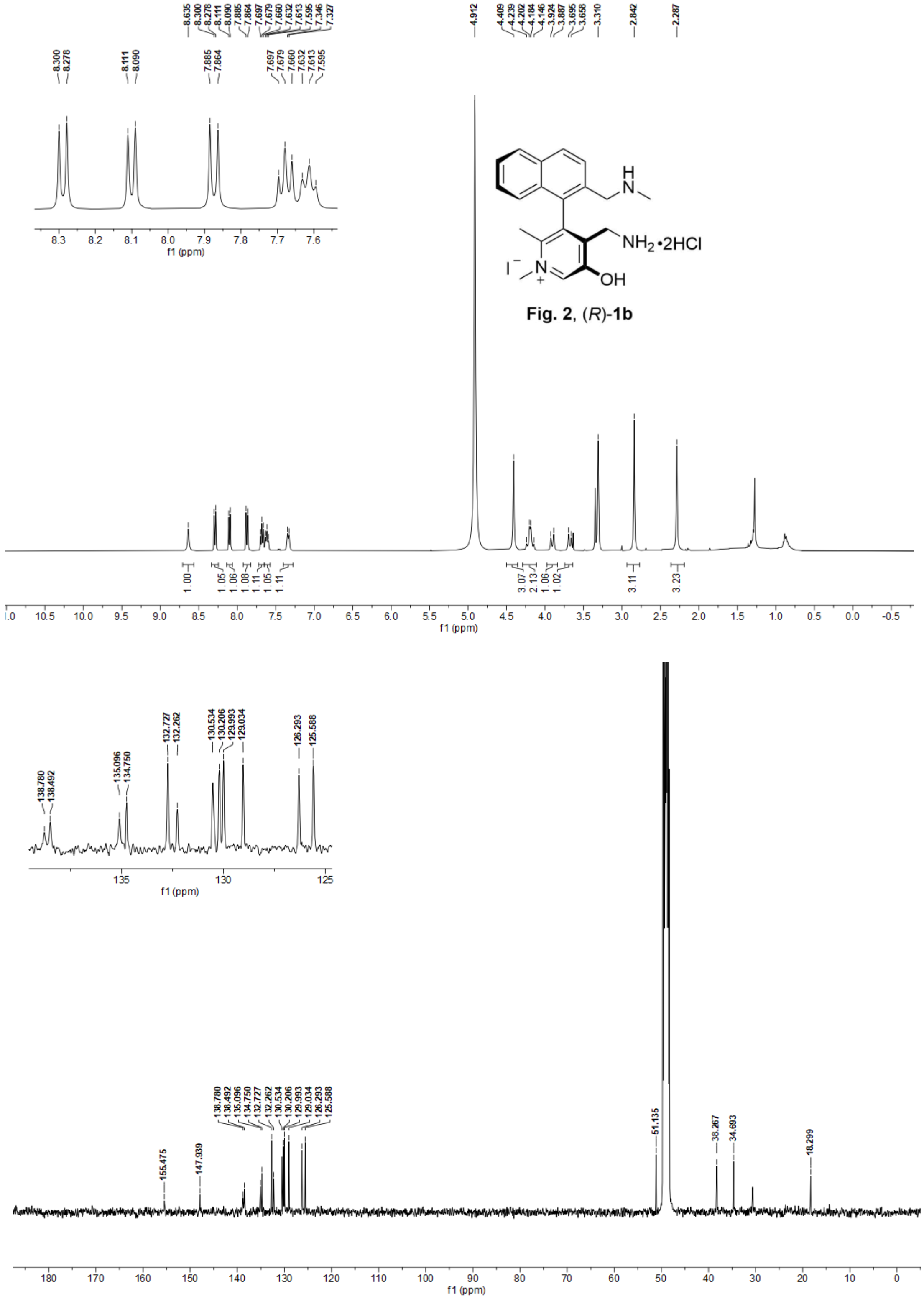

Supplementary Figure 8. ^{1}H NMR and ^{13}C NMR spectra of compound (*R*)**-1b**

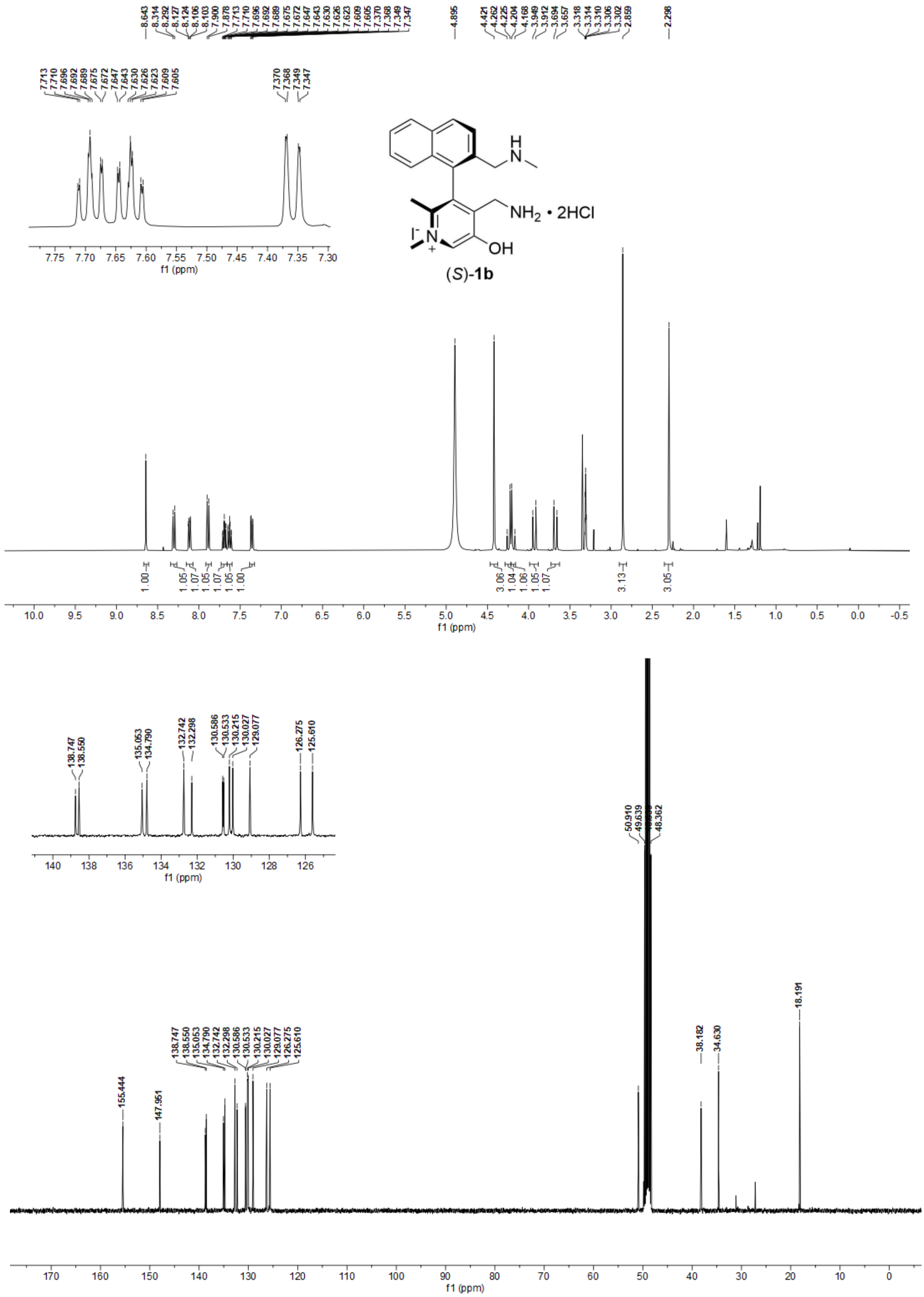

Supplementary Figure 9. ^{1}H NMR and ^{13}C NMR spectra of compound (*S*)-**1b**

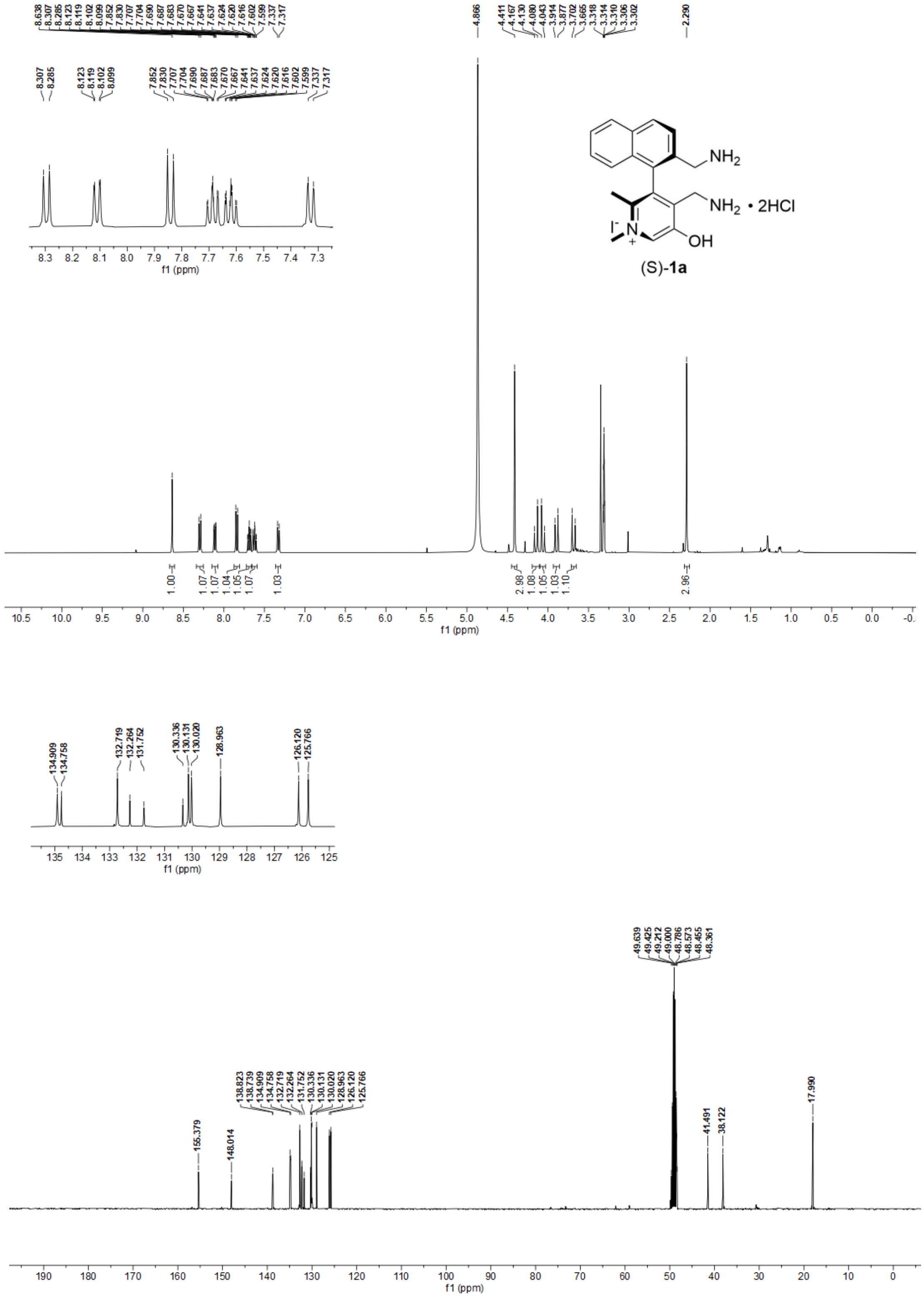

Supplementary Figure 10. ^{1}H NMR and ^{13}C NMR spectra of compound (*S*)-**1a**

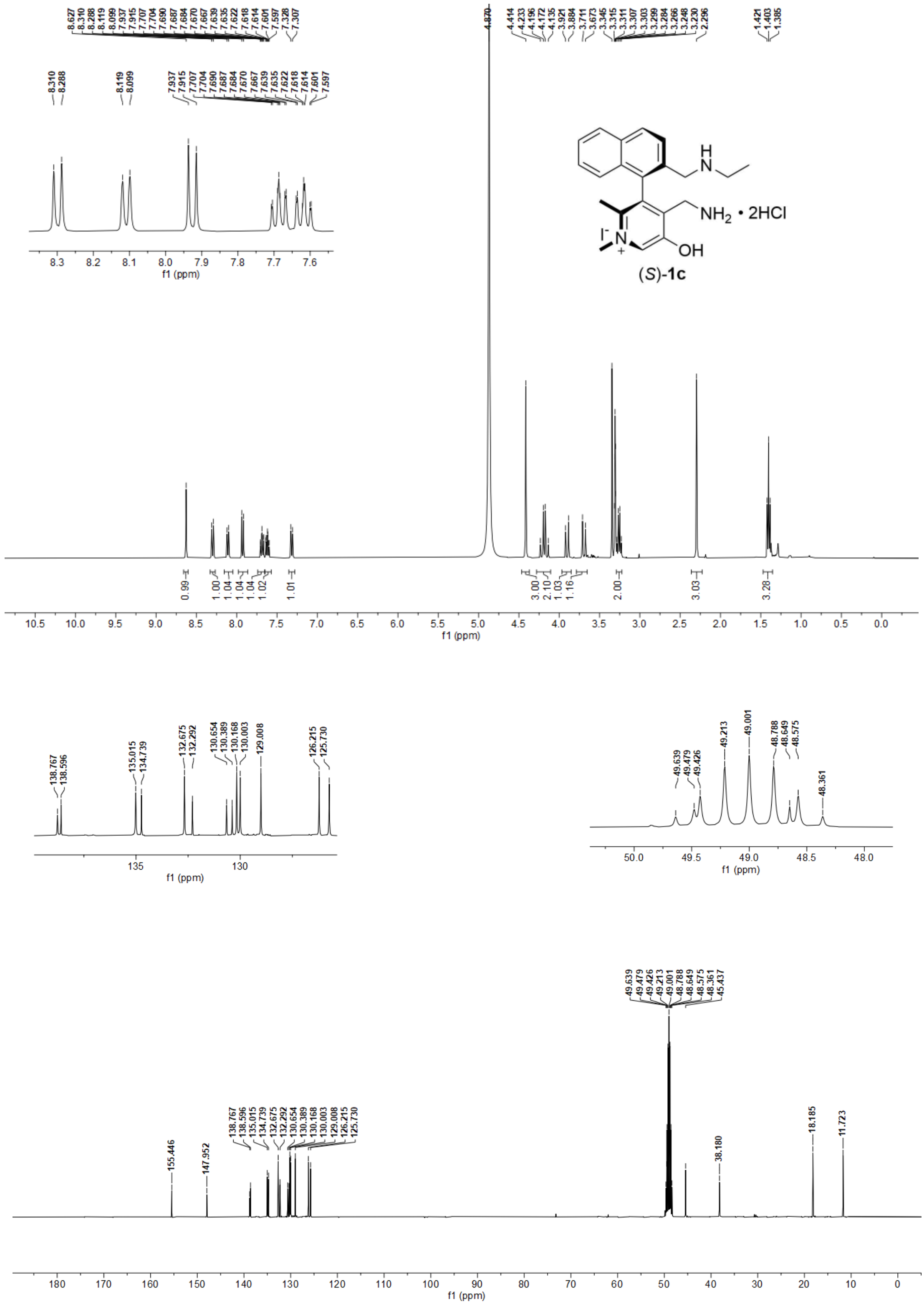

Supplementary Figure 11. ^{1}H NMR and ^{13}C NMR spectra of compound (*S*)-**1c**

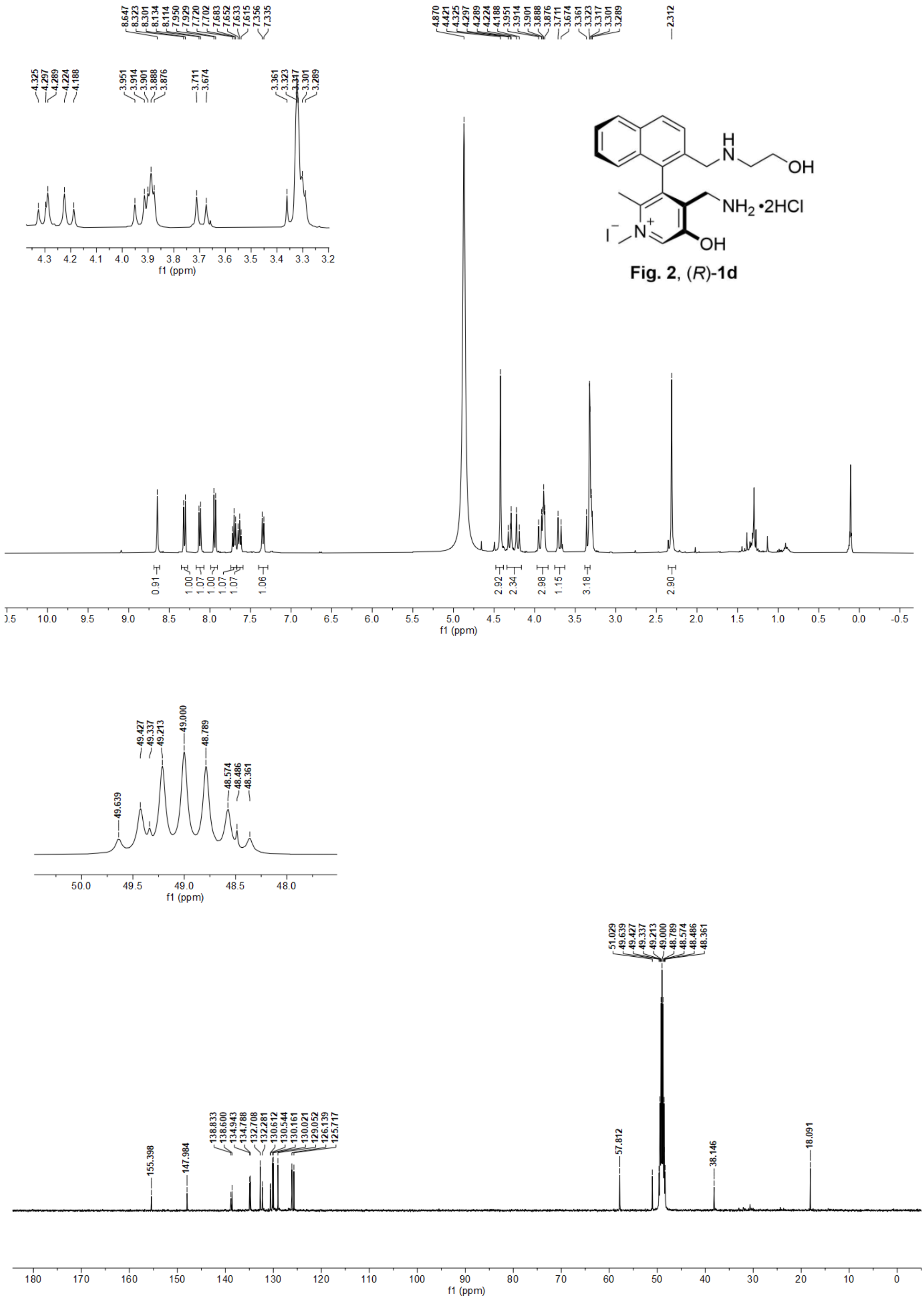

Supplementary Figure 12. ^{1}H NMR and ^{13}C NMR spectra of compound (*R*)**-1d**

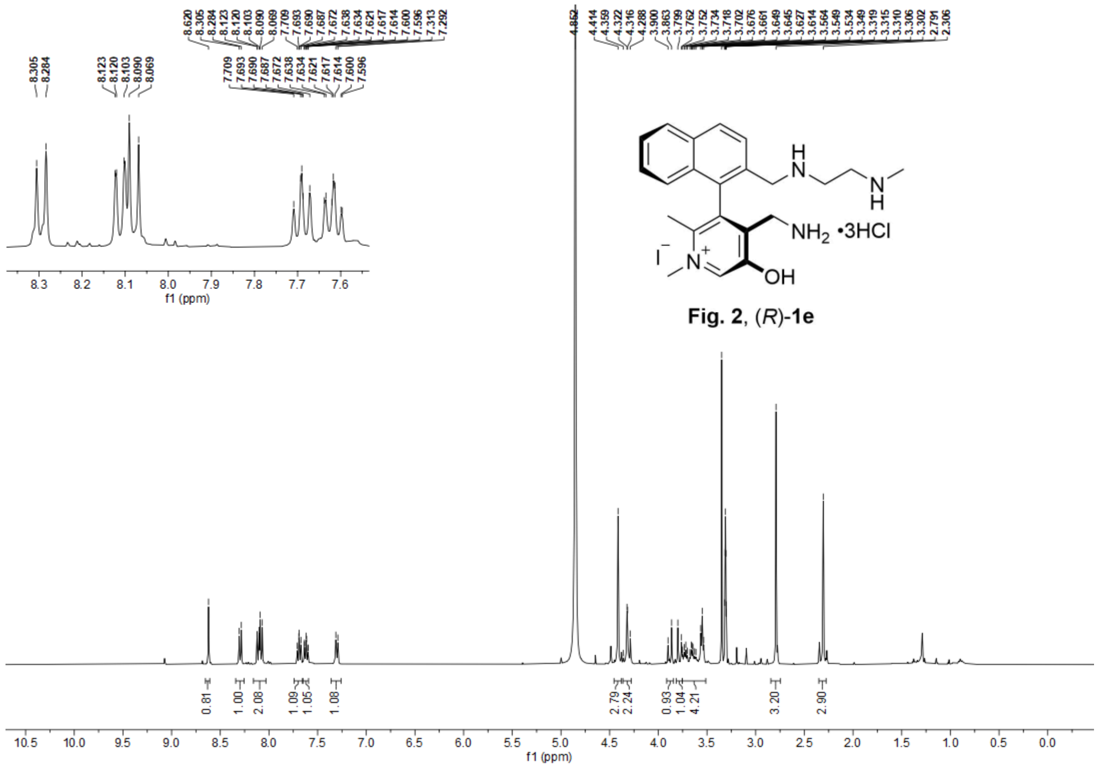

Fig. 2, (*R*)-**1e**

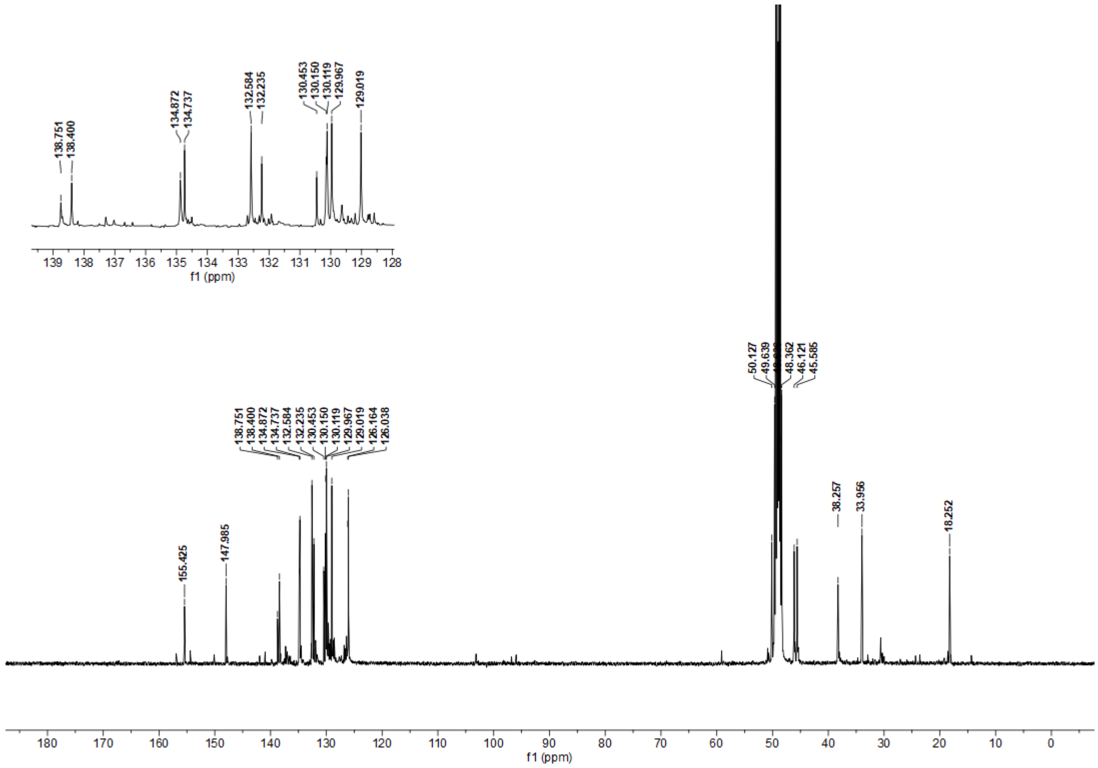

Supplementary Figure 13. ^{1}H NMR and ^{13}C NMR spectra of compound (*R*)-**1e**

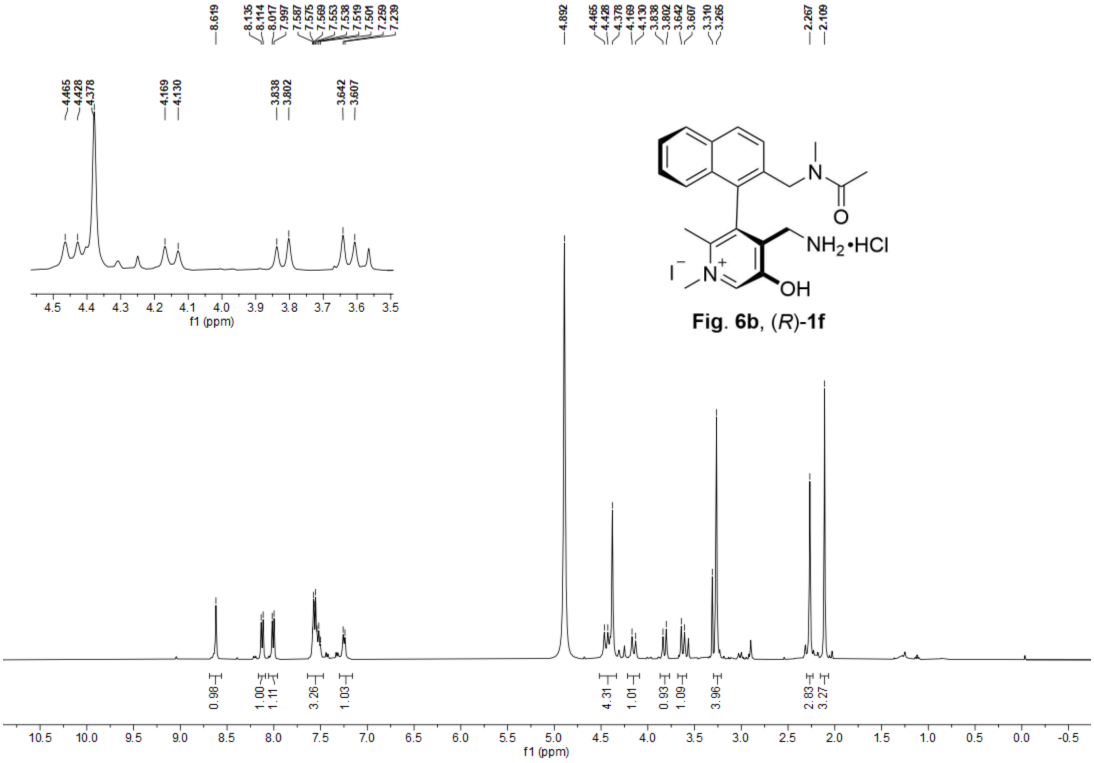

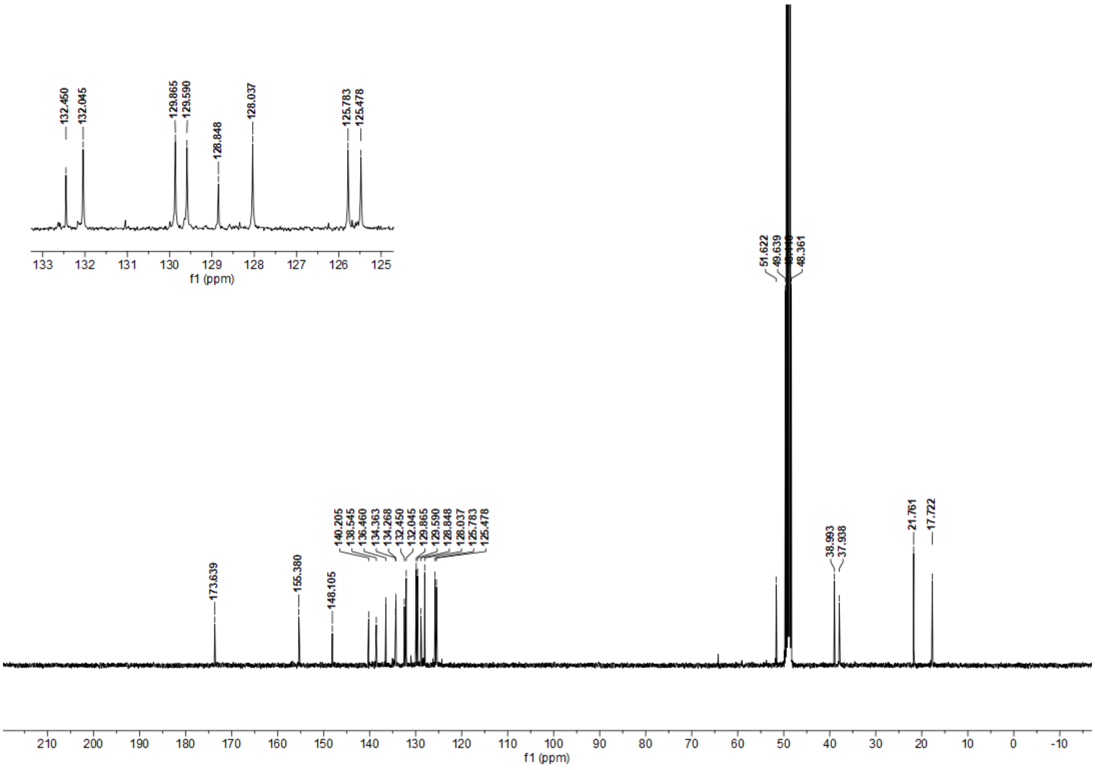

Supplementary Figure 14. ^{1}H NMR and ^{13}C NMR spectra of compound (*R*)-**1f**

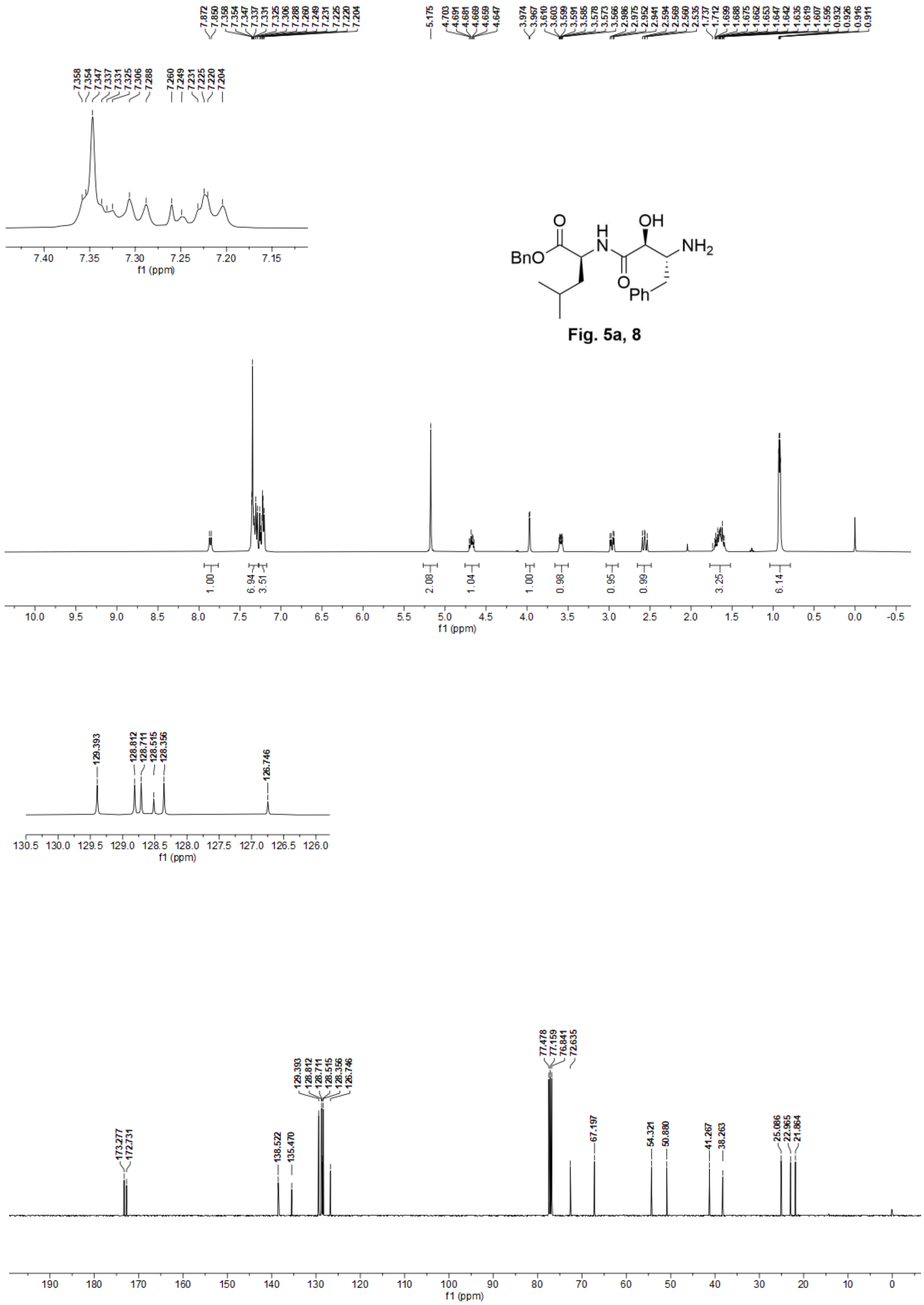

Supplementary Figure 15. ^{1}H NMR and ^{13}C NMR spectra of compound **8**

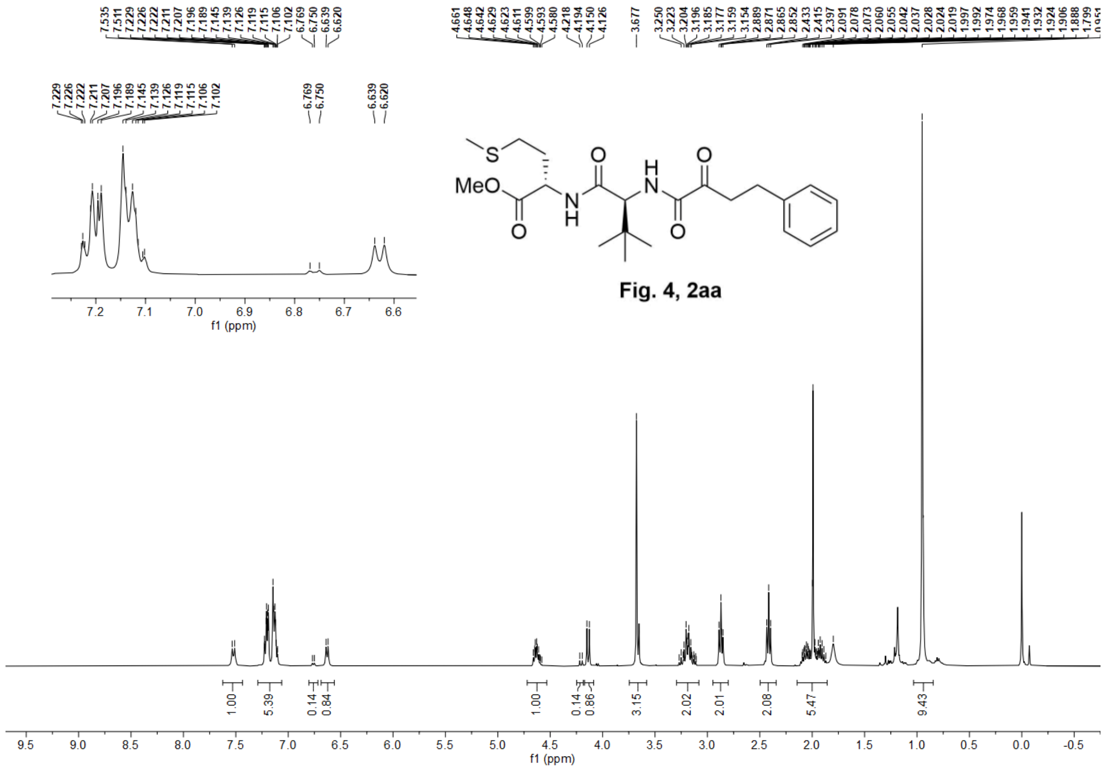

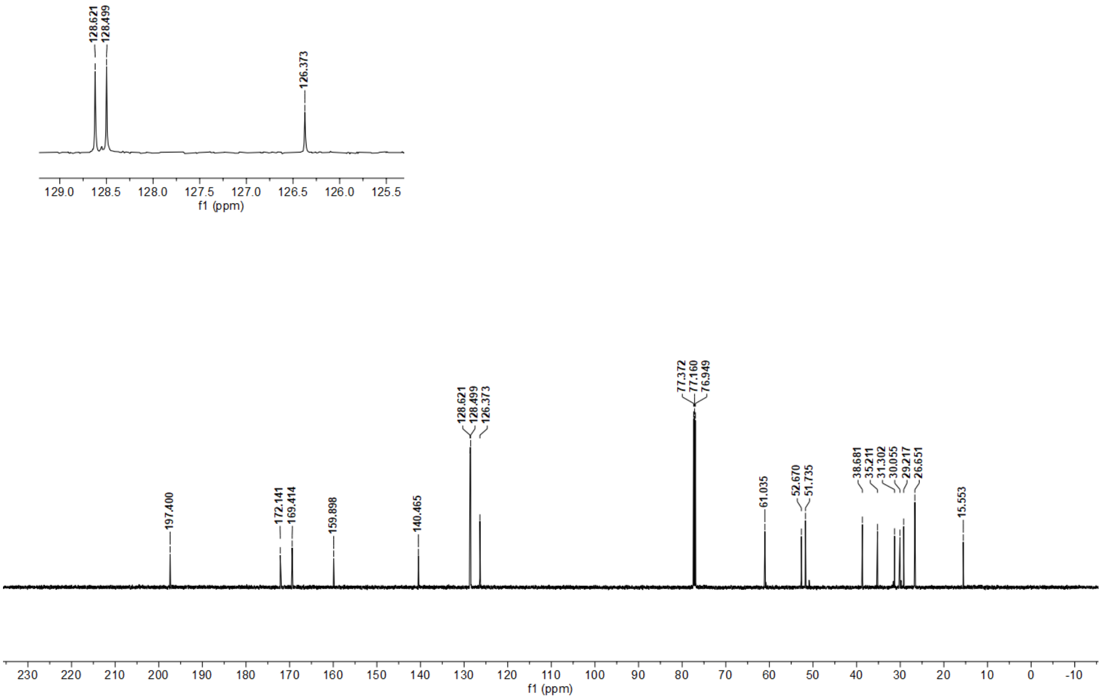

Supplementary Figure 16. ^{1}H NMR and ^{13}C NMR spectra of compound **2aa**

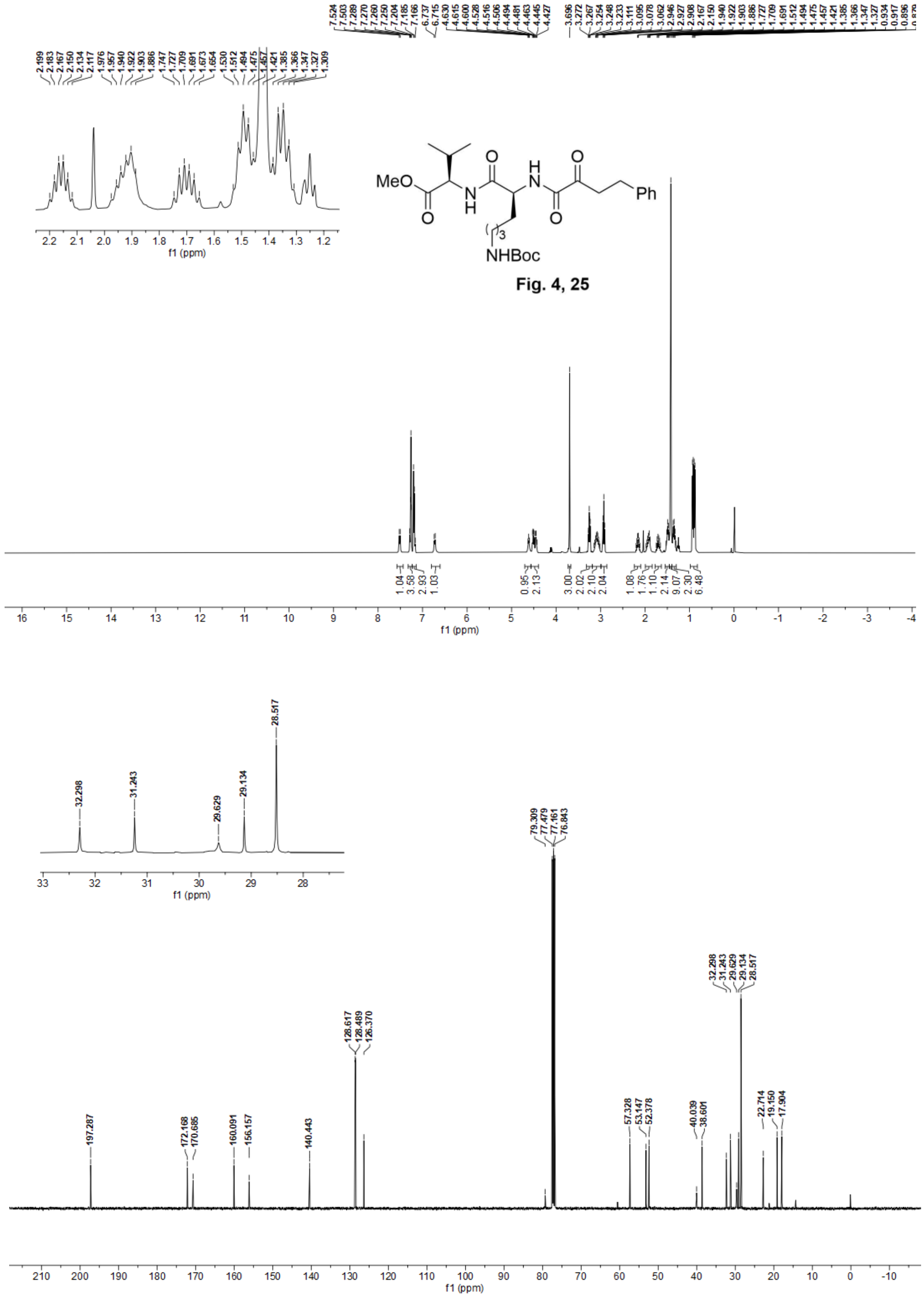

Supplementary Figure 17. ^{1}H NMR and ^{13}C NMR spectra of compound **25**

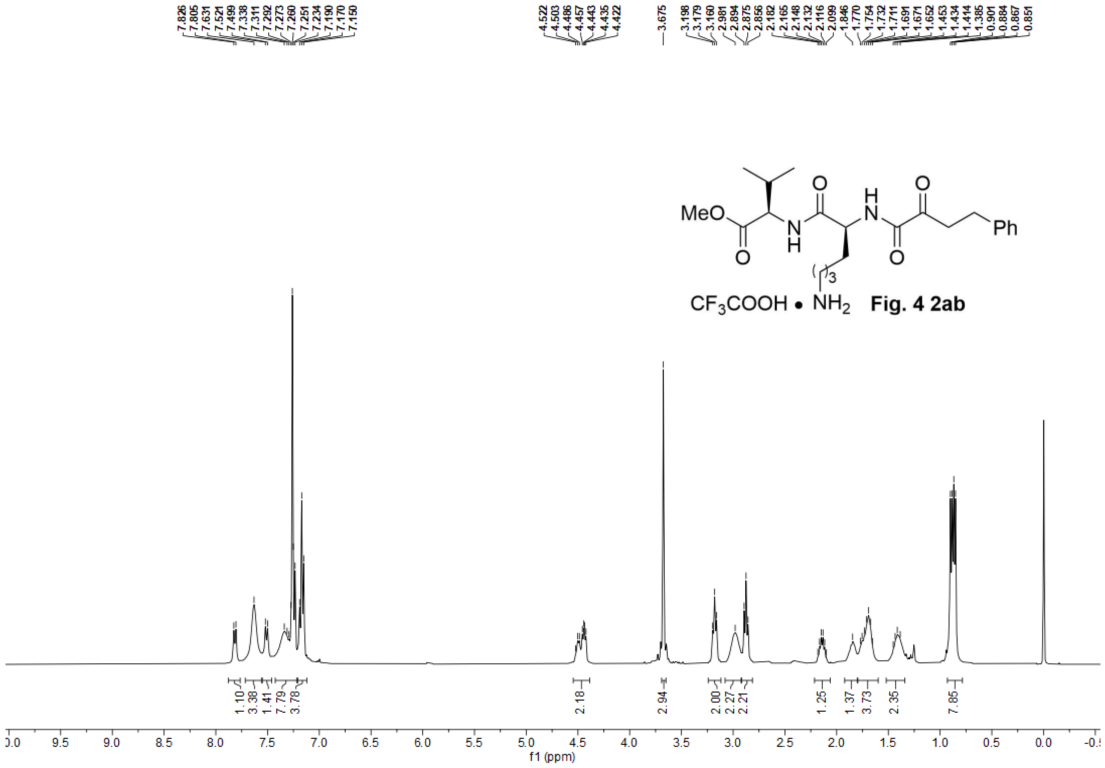

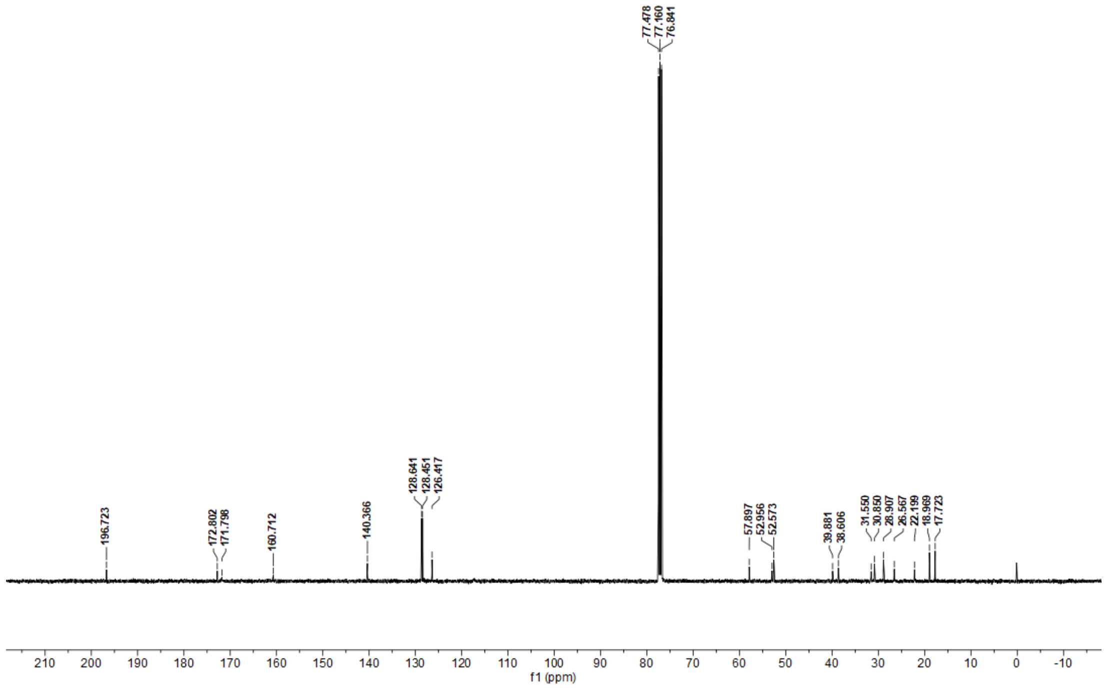

Supplementary Figure 18. ^{1}H NMR and ^{13}C NMR spectra of compound **2ab**

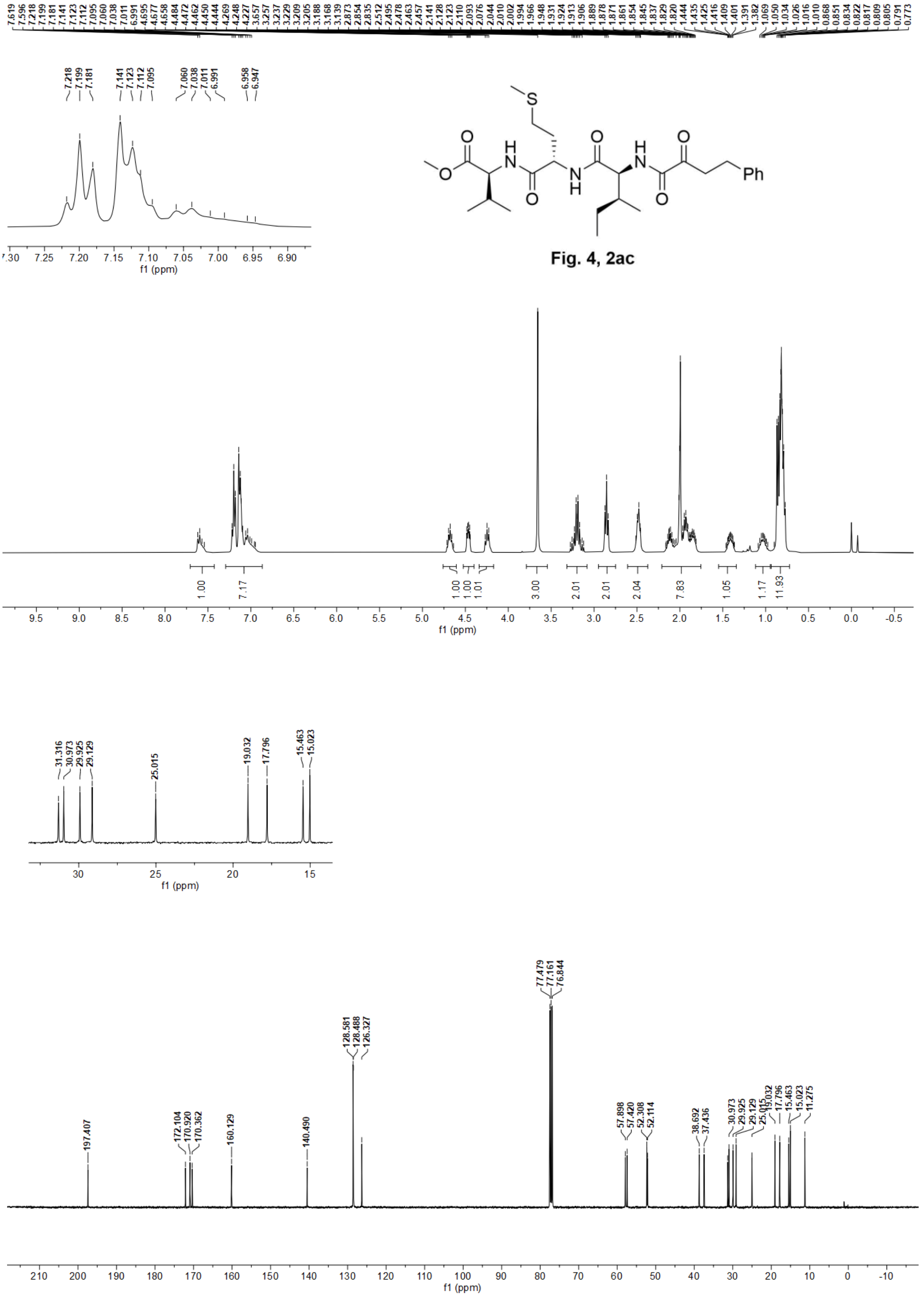

Supplementary Figure 19. ^{1}H NMR and ^{13}C NMR spectra of compound **2ac**

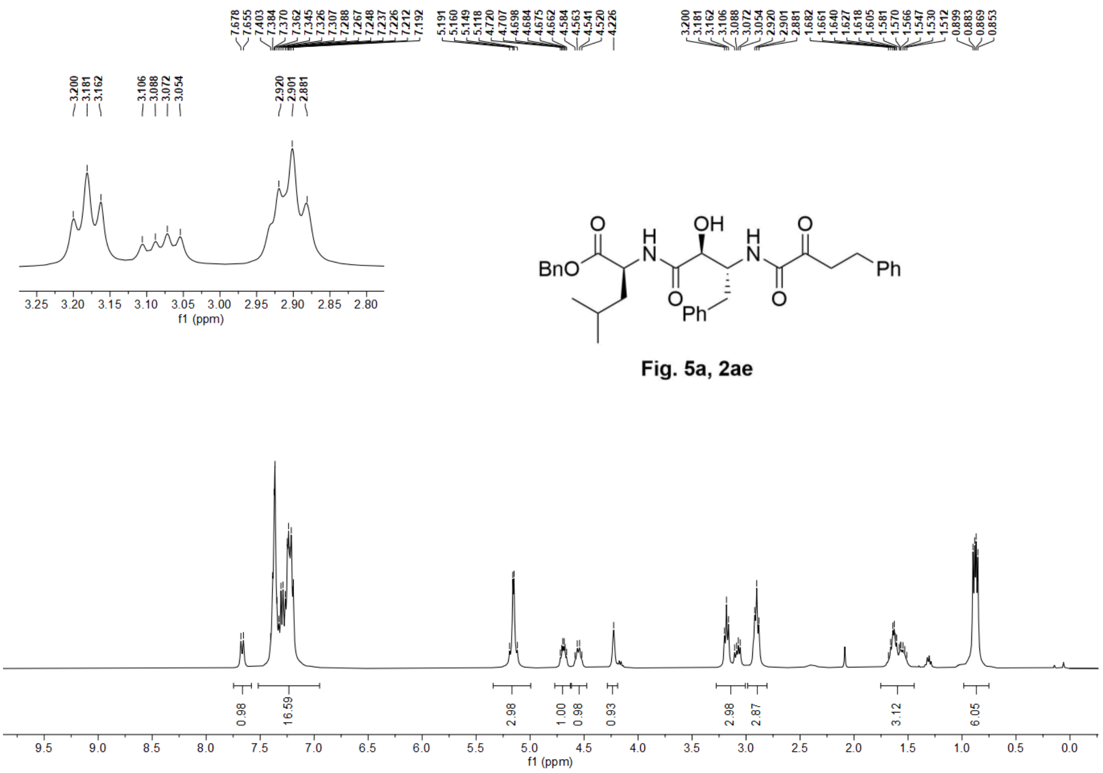

Fig. 5a, 2ae

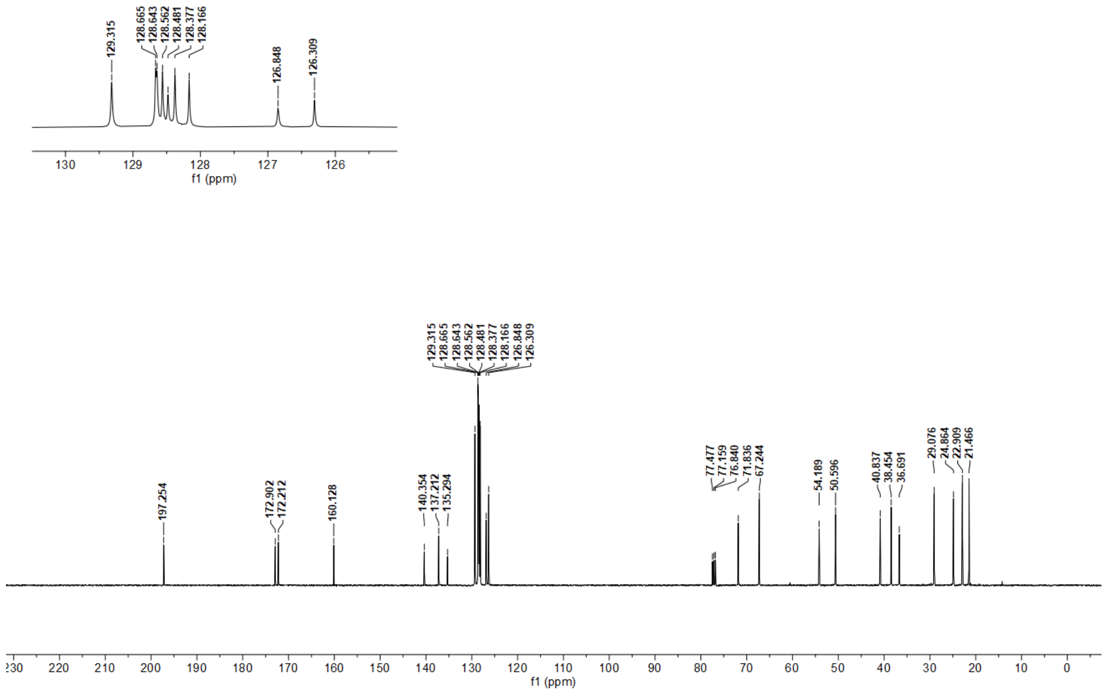

Supplementary Figure 20. ^{1}H NMR and ^{13}C NMR spectra of compound **2ae**

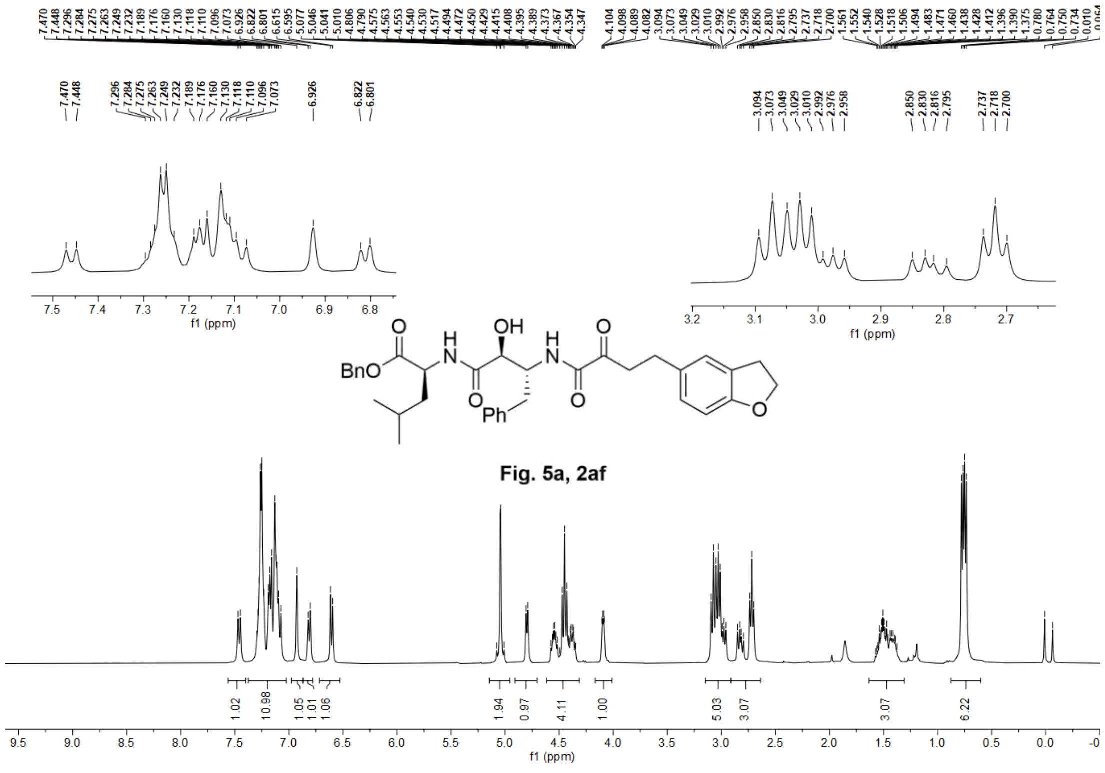

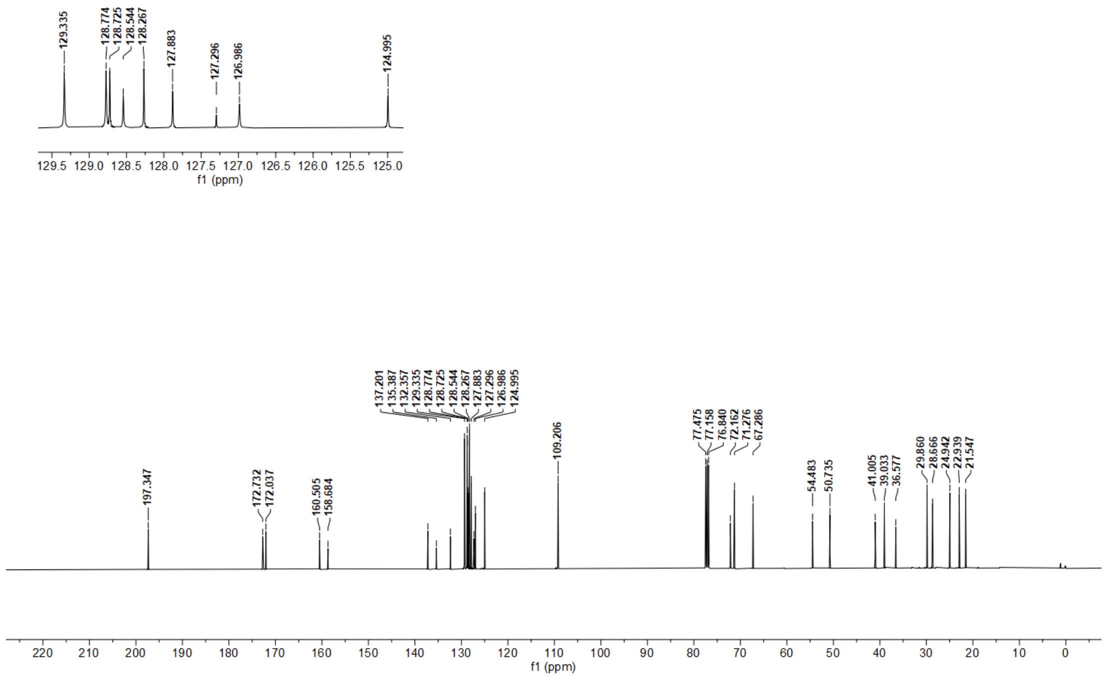

Supplementary Figure 21. ^{1}H NMR and ^{13}C NMR spectra of compound **2af**

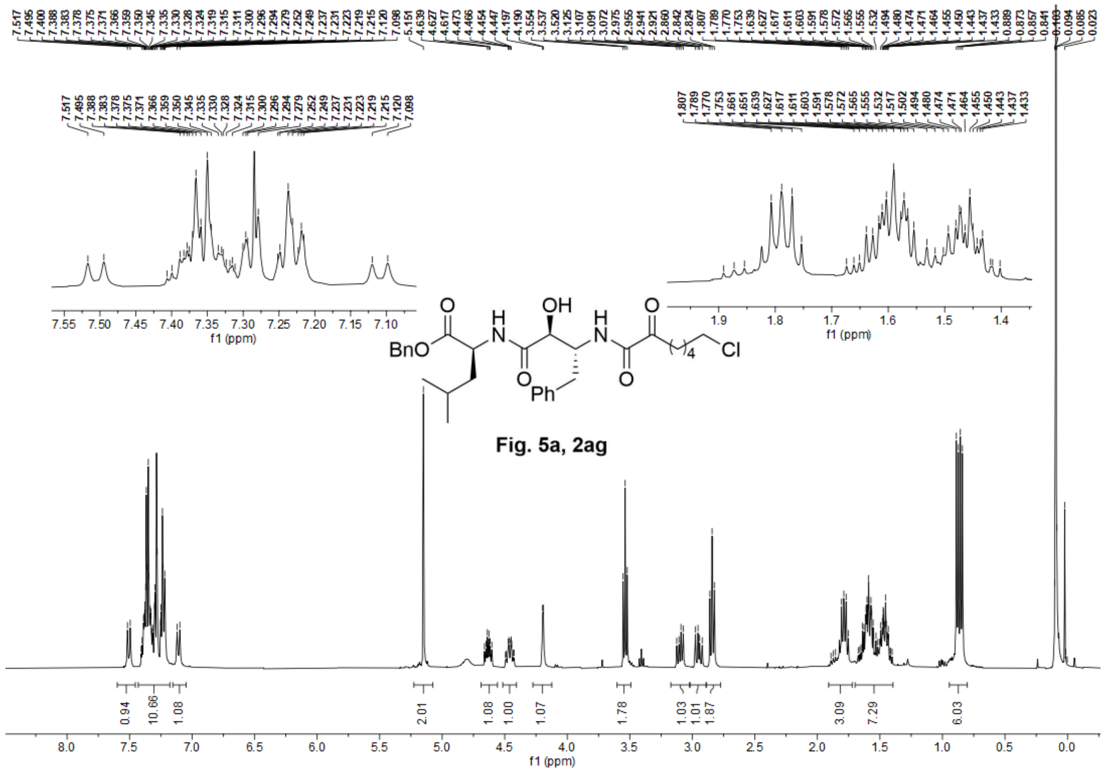

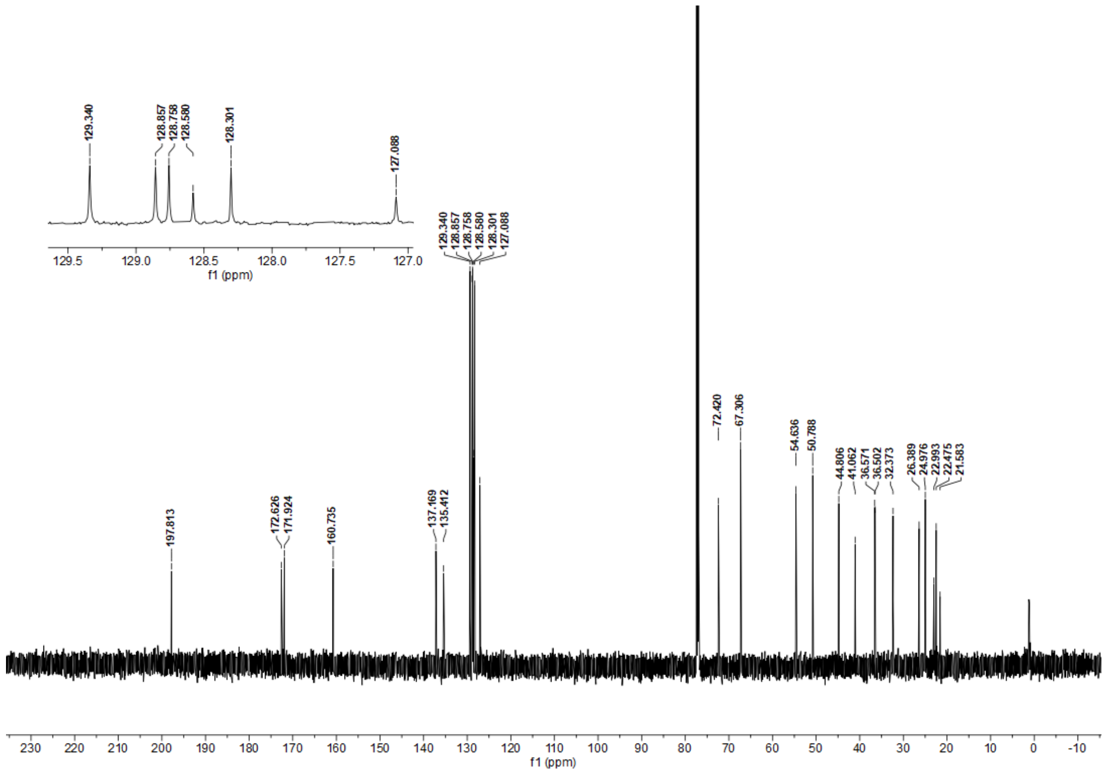

Supplementary Figure 22. ^{1}H NMR and ^{13}C NMR spectra of compound **2ag**

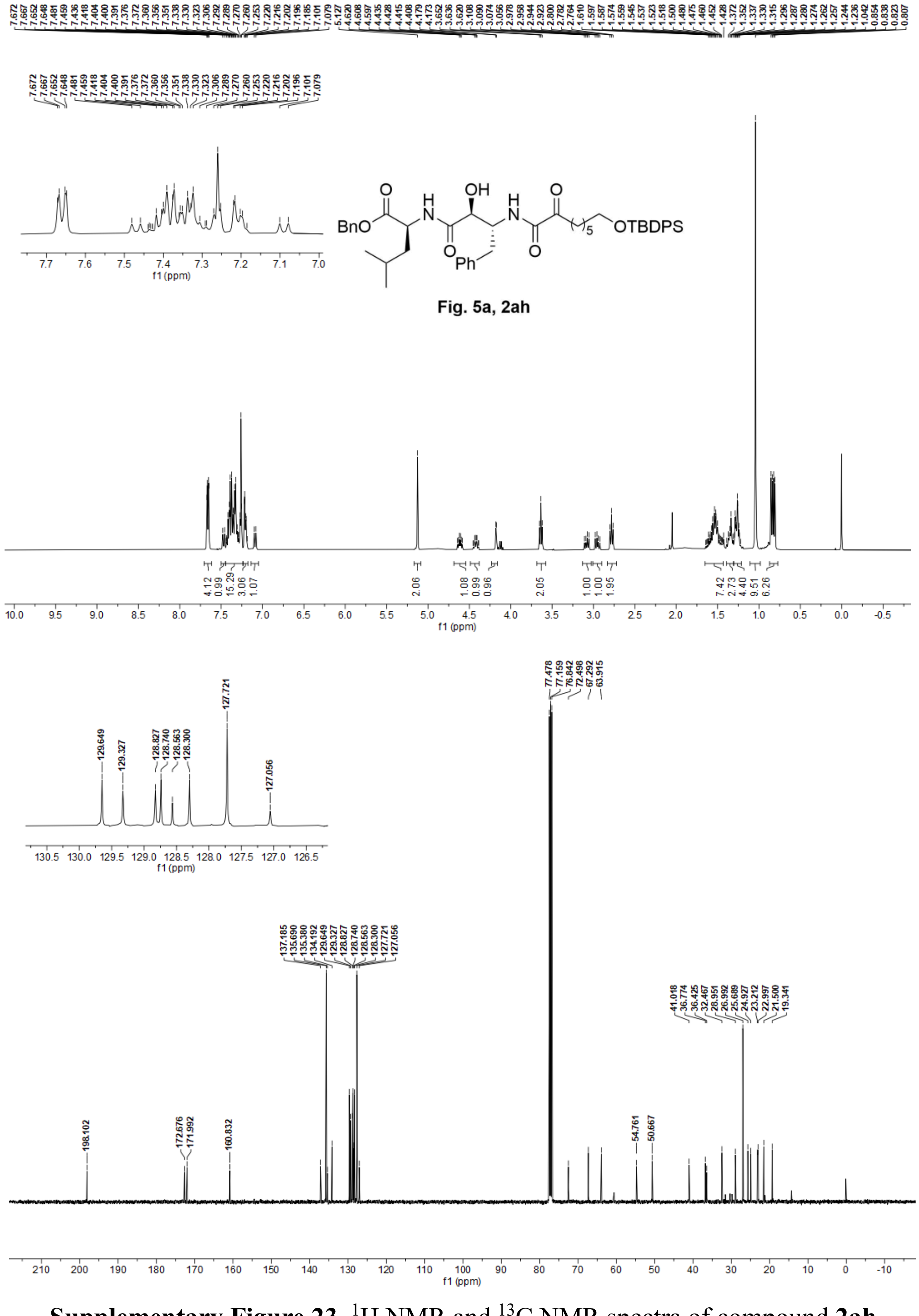

Supplementary Figure 23. ^{1}H NMR and ^{13}C NMR spectra of compound **2ah**

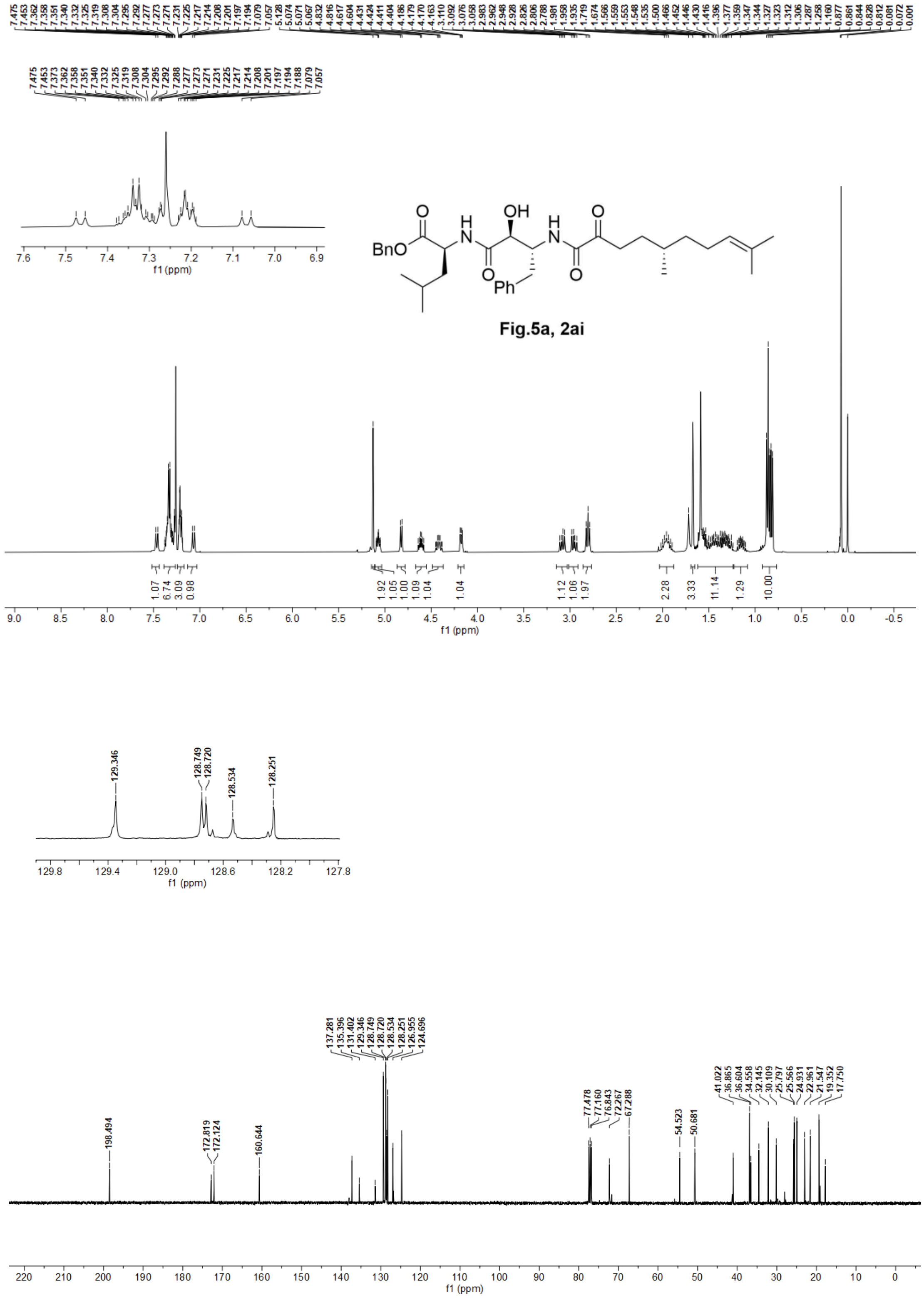

Supplementary Figure 24. ^{1}H NMR and ^{13}C NMR spectra of compound **2ai**

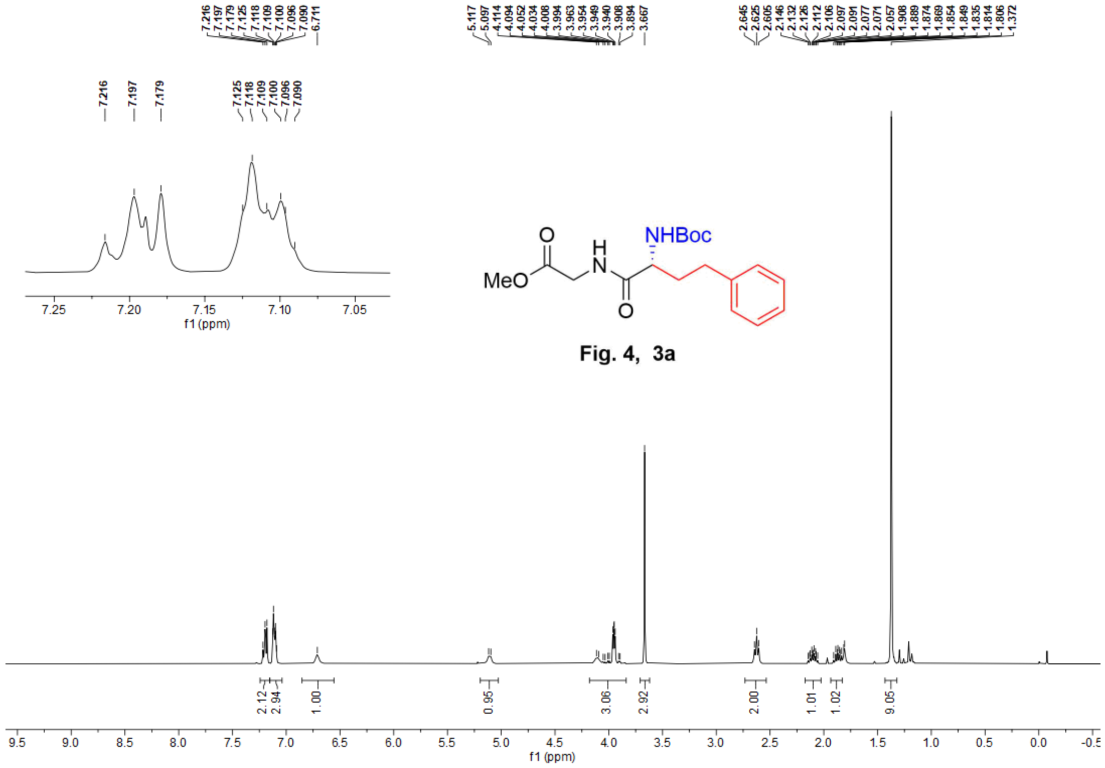

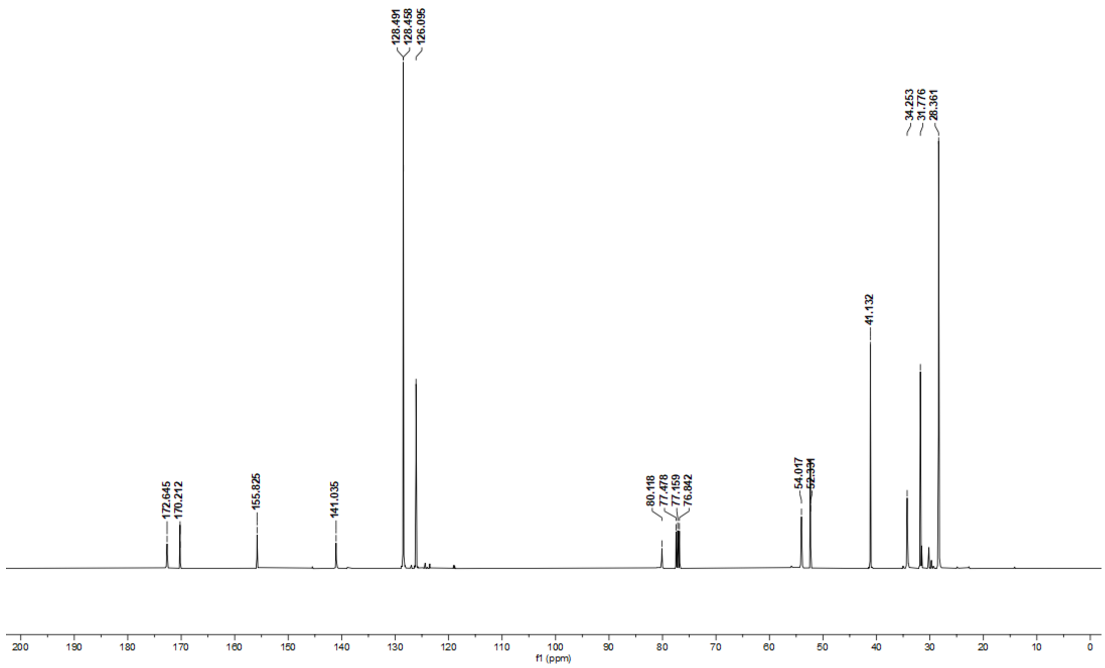

Supplementary Figure 25. ^{1}H NMR and ^{13}C NMR spectra of compound **3a**

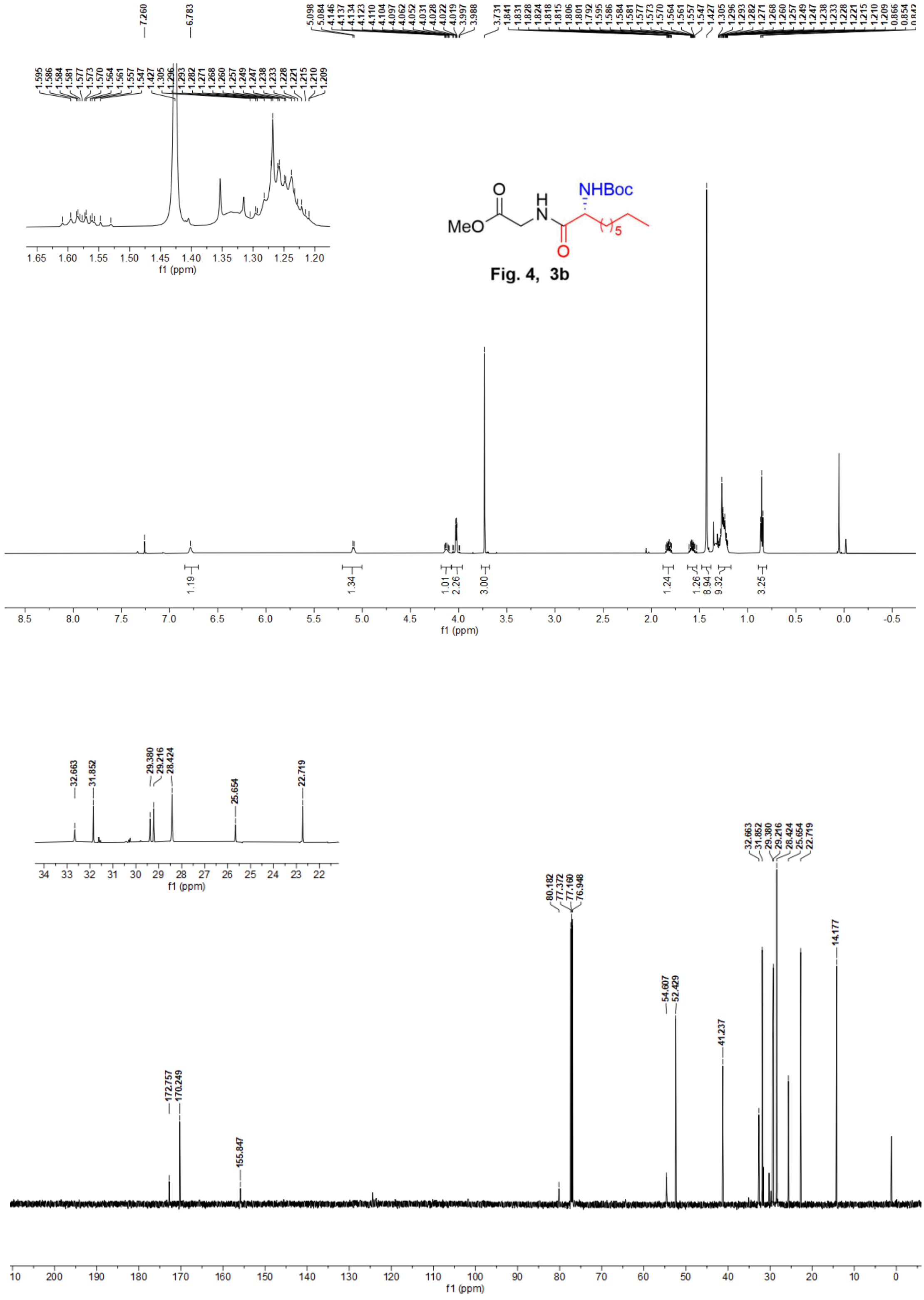

Supplementary Figure 26. ^{1}H NMR and ^{13}C NMR spectra of compound **3b**

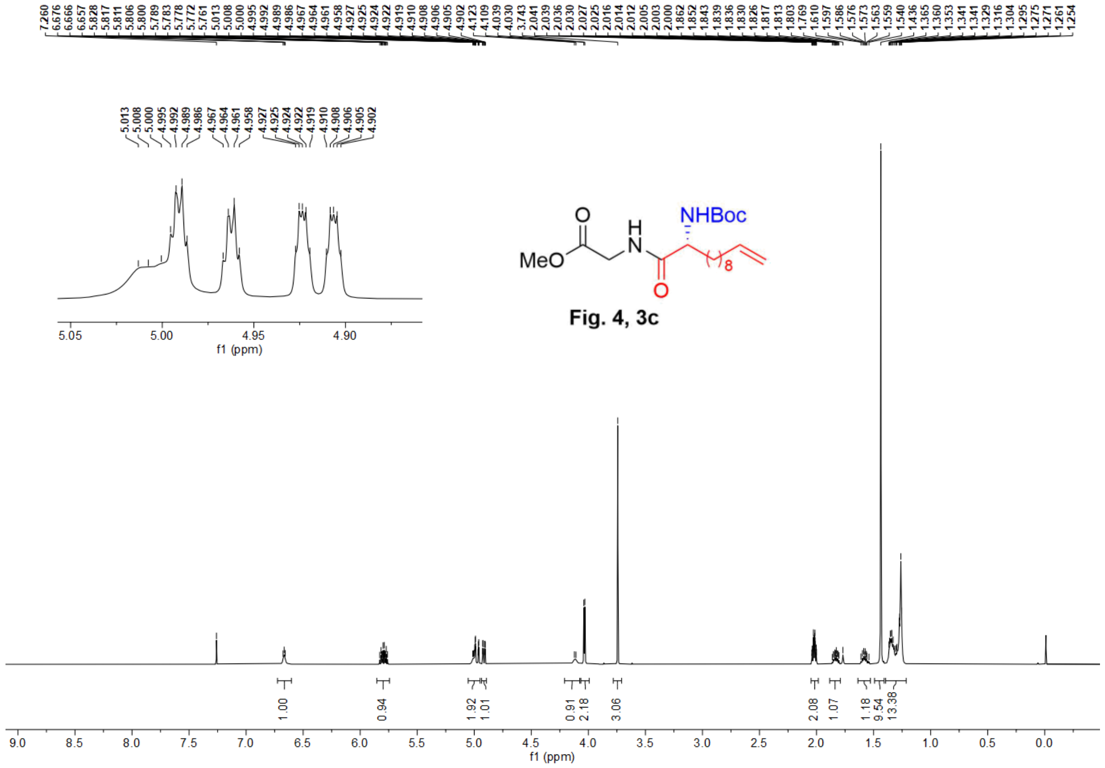

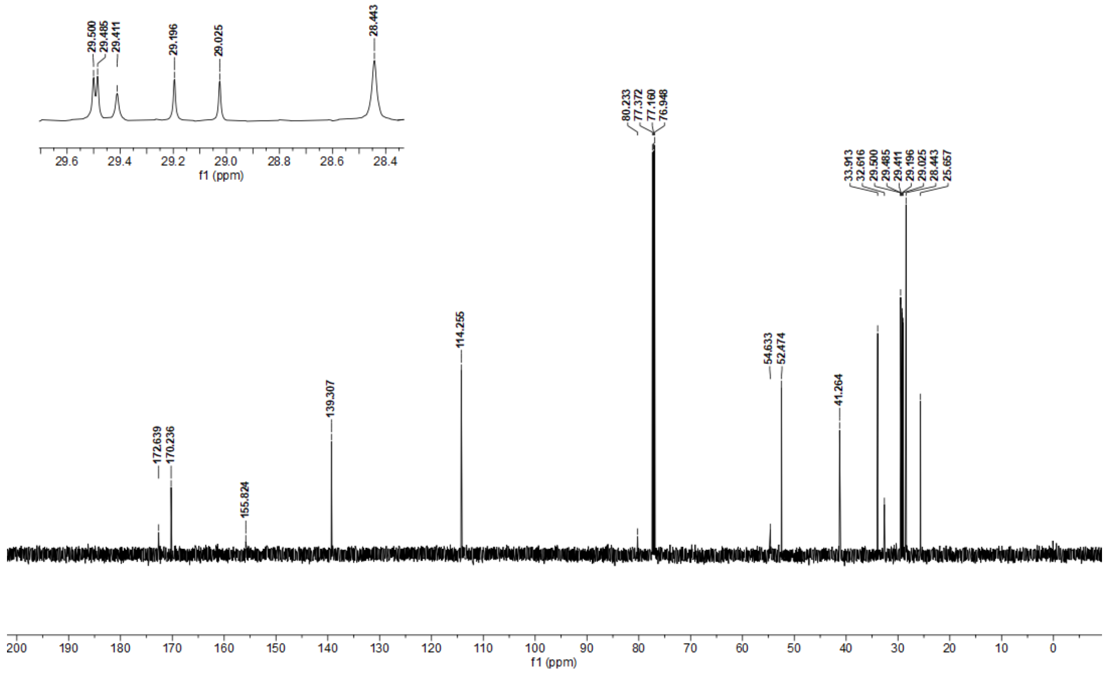

Supplementary Figure 27. ^{1}H NMR and ^{13}C NMR spectra of compound **3c**

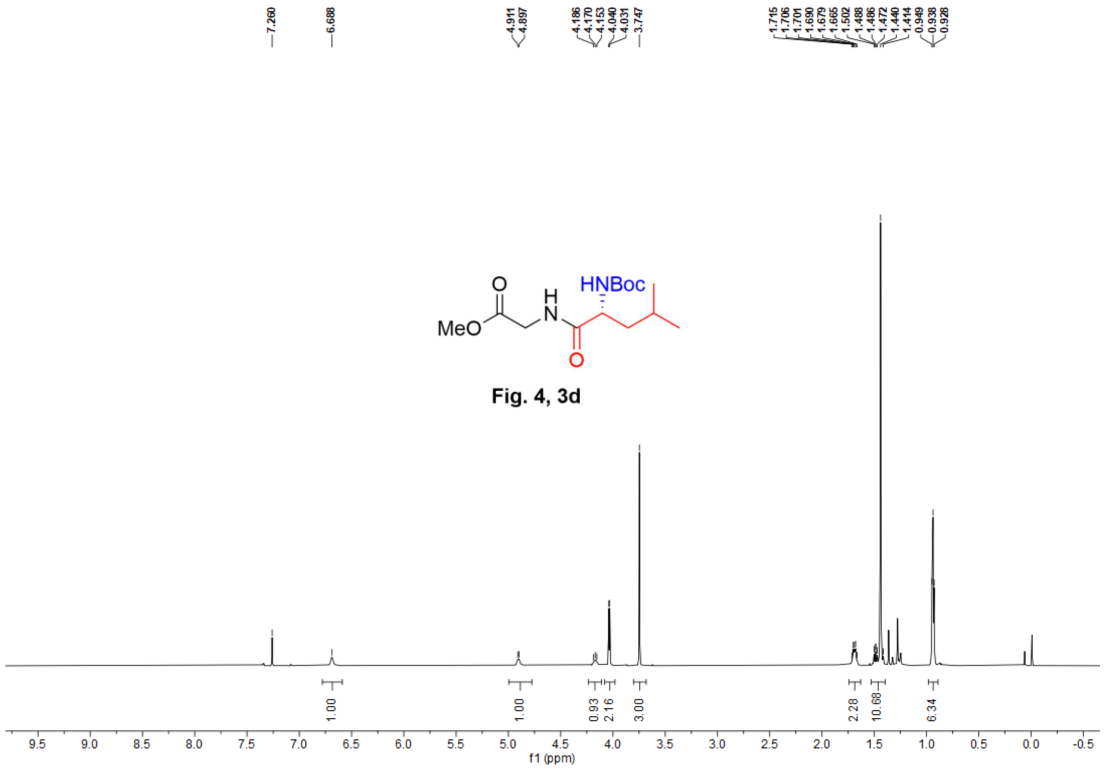

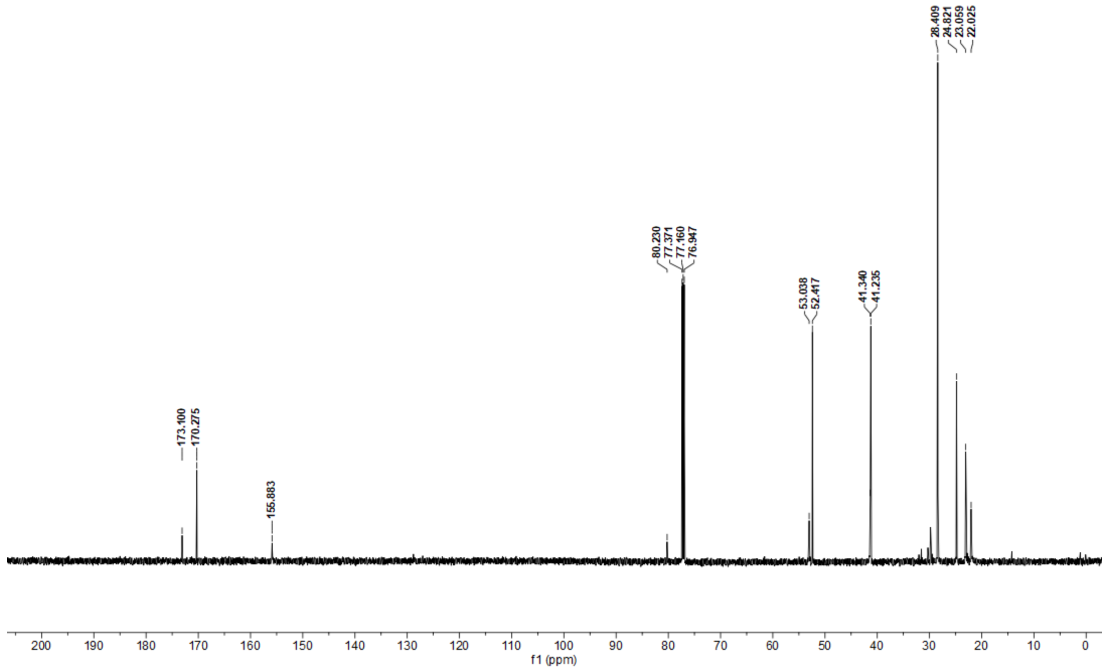

Supplementary Figure 28. ^{1}H NMR and ^{13}C NMR spectra of compound **3d**

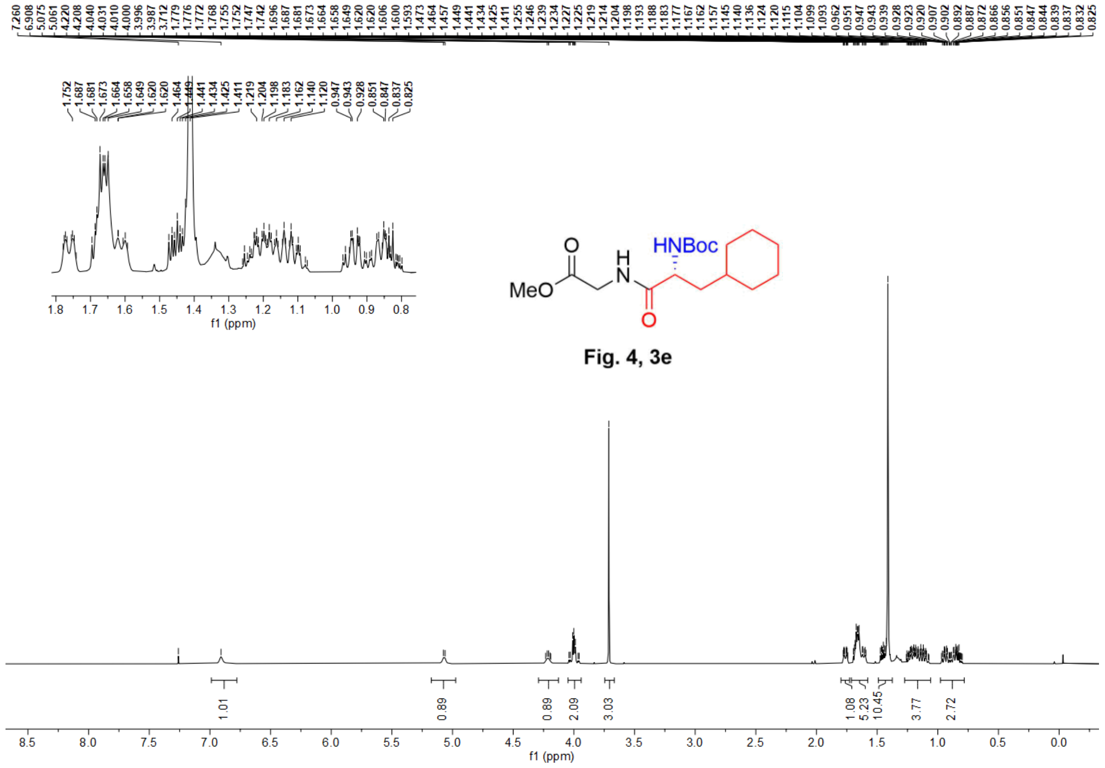

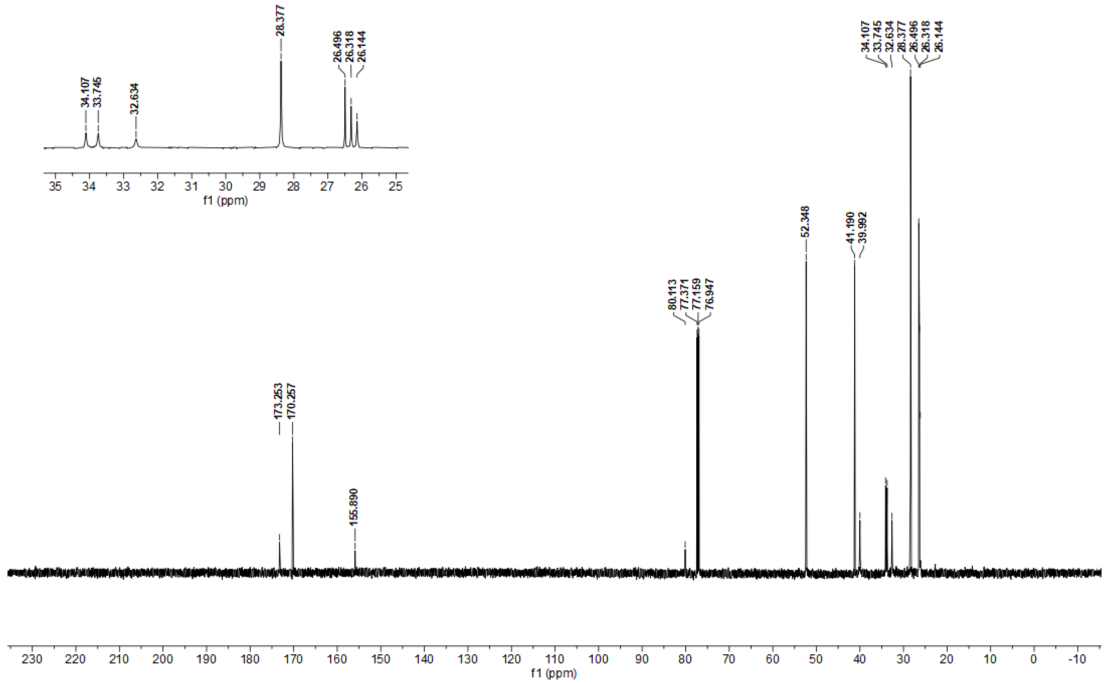

Supplementary Figure 29. ^{1}H NMR and ^{13}C NMR spectra of compound **3e**

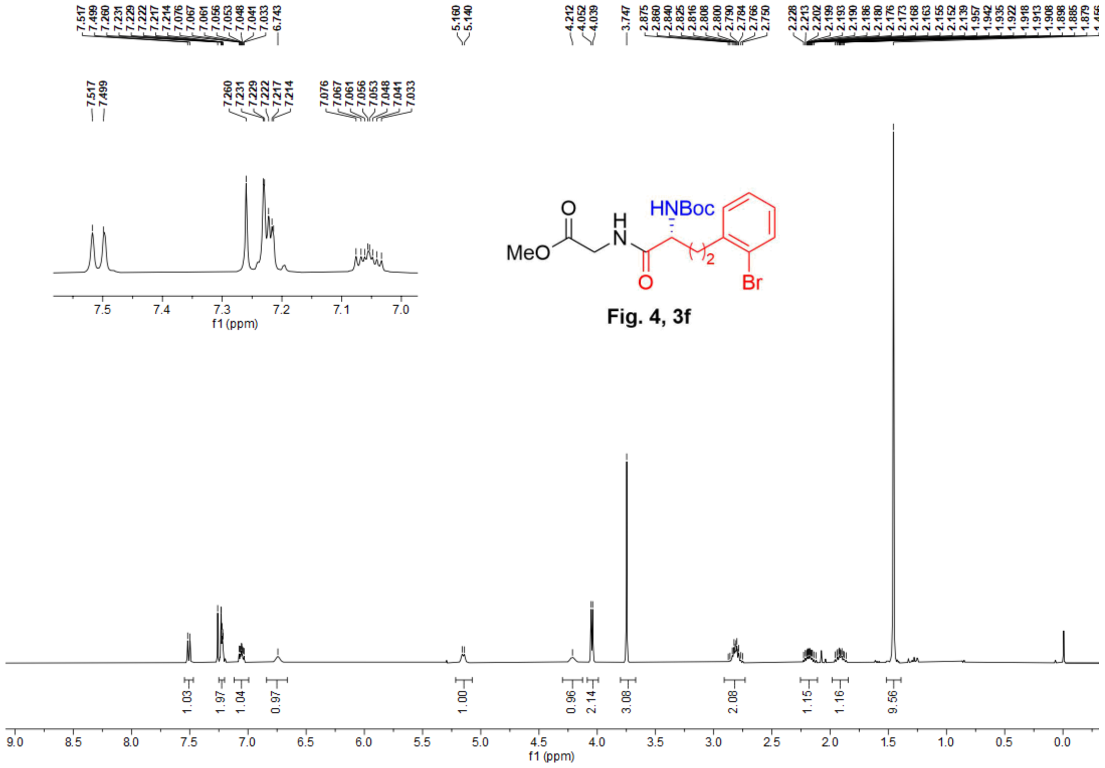

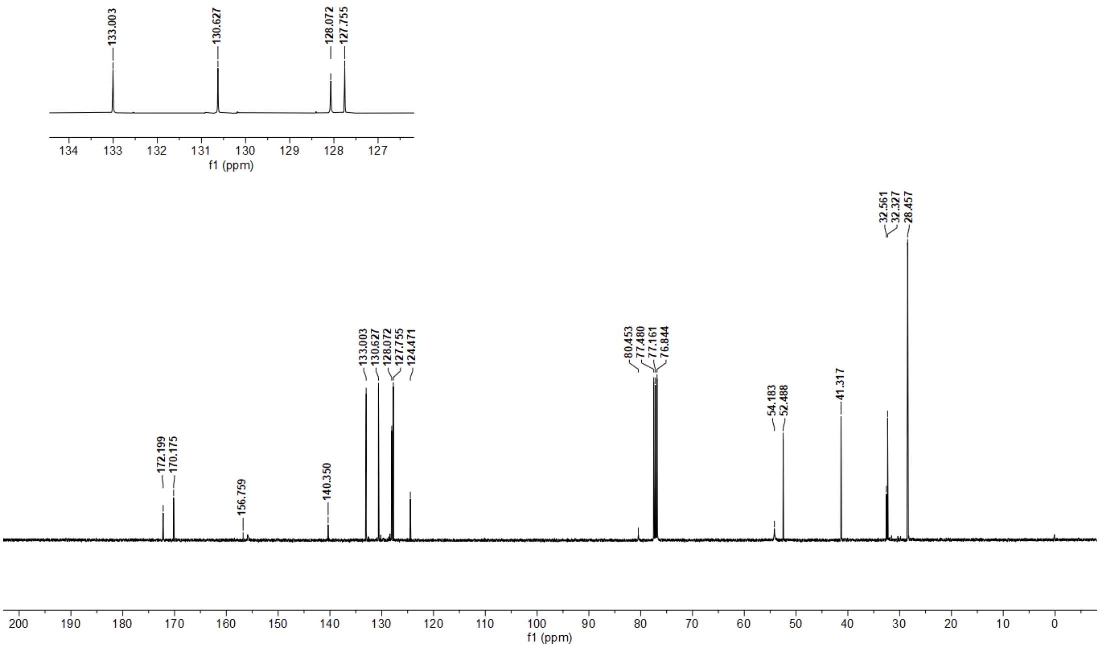

Supplementary Figure 30. ^{1}H NMR and ^{13}C NMR spectra of compound **3f**

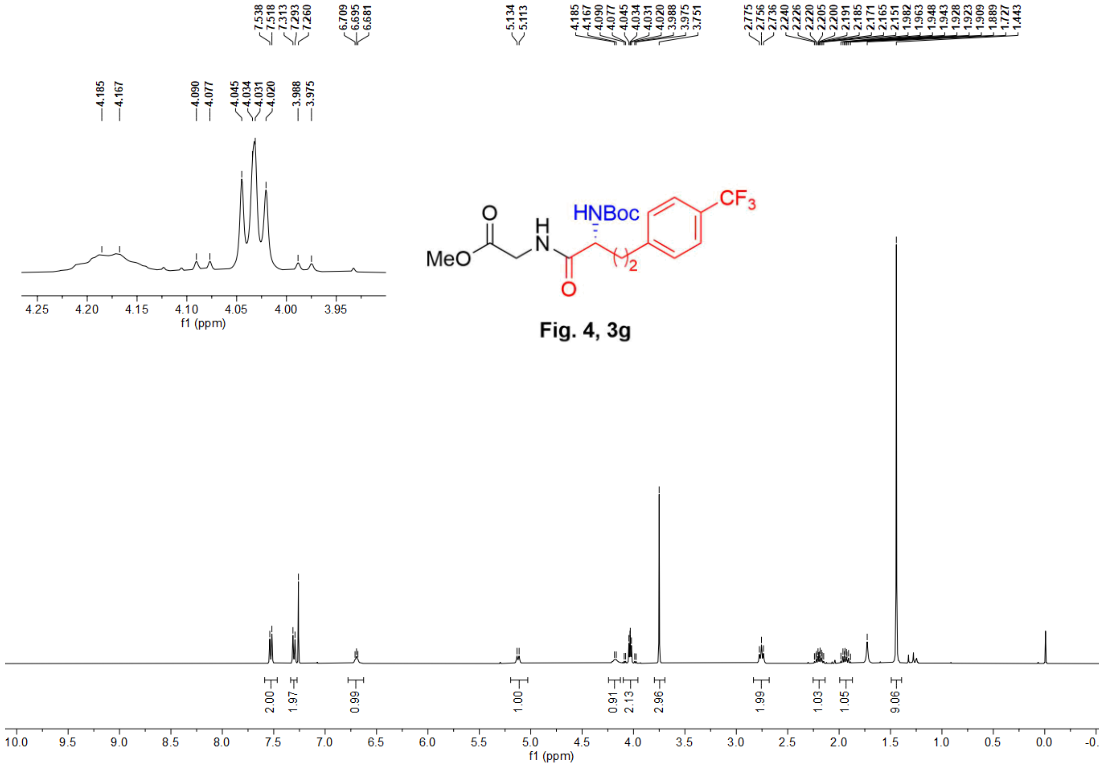

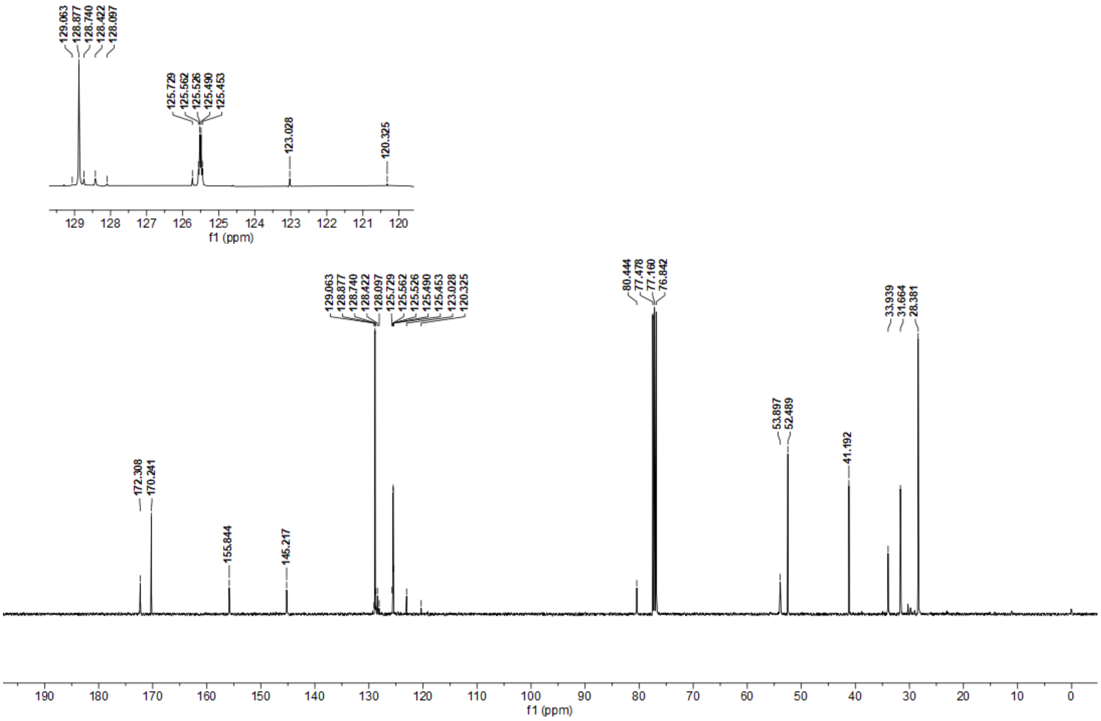

Supplementary Figure 31. ^{1}H NMR and ^{13}C NMR spectra of compound **3g**

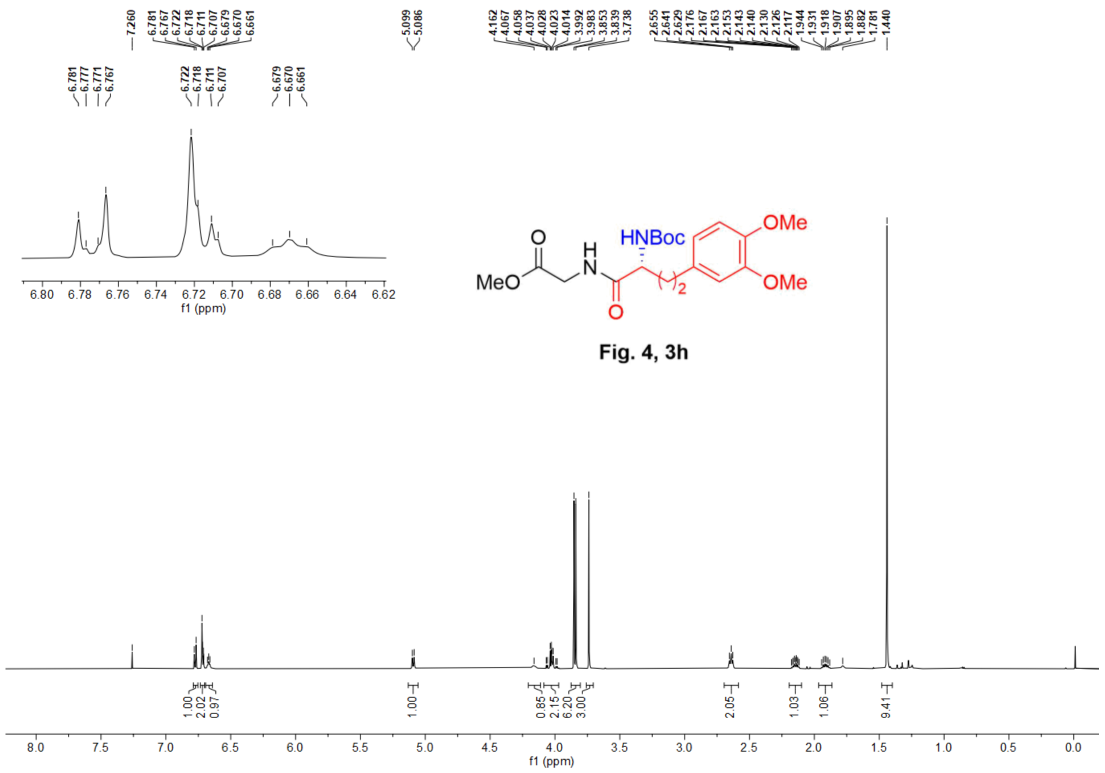

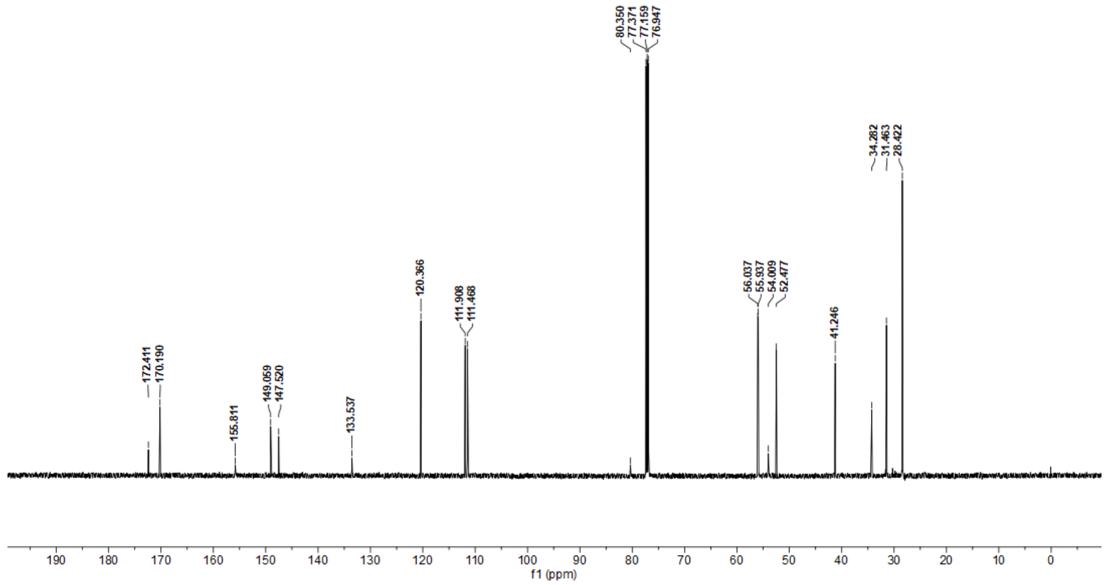

Supplementary Figure 32. ^{1}H NMR and ^{13}C NMR spectra of compound **3h**

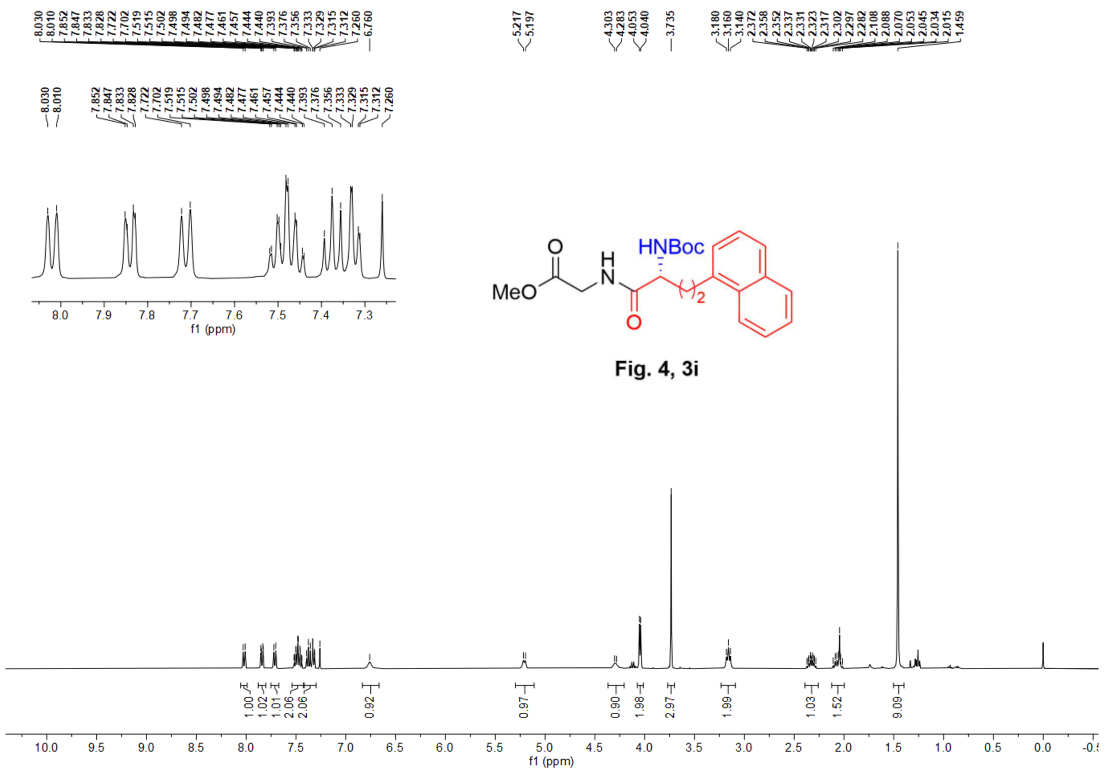

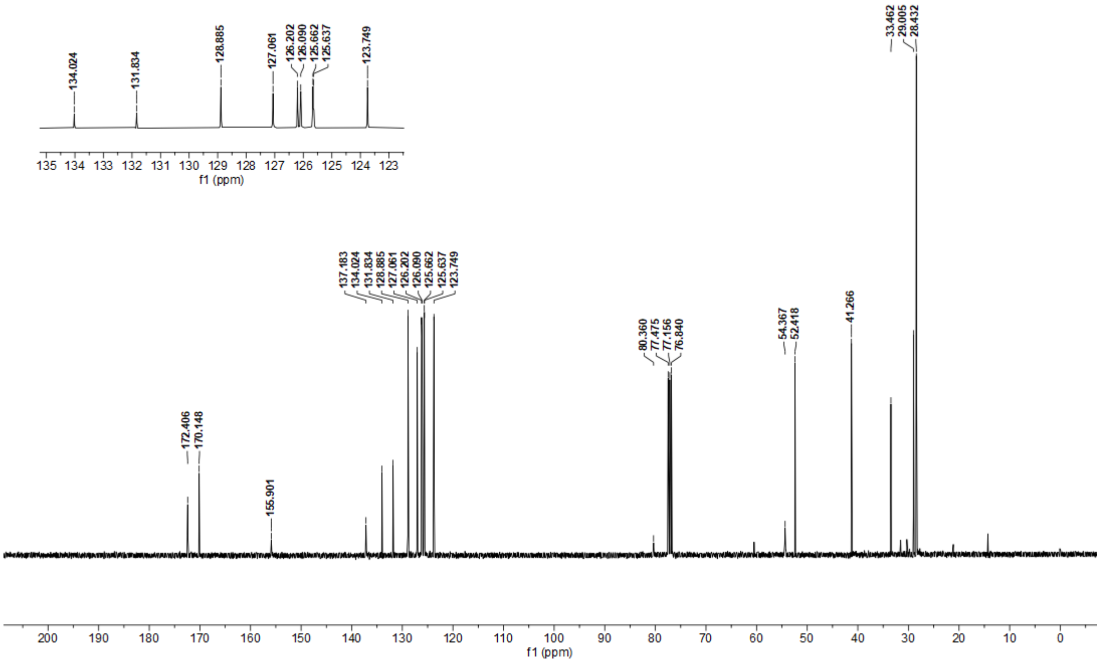

Supplementary Figure 33. ^{1}H NMR and ^{13}C NMR spectra of compound **3i**

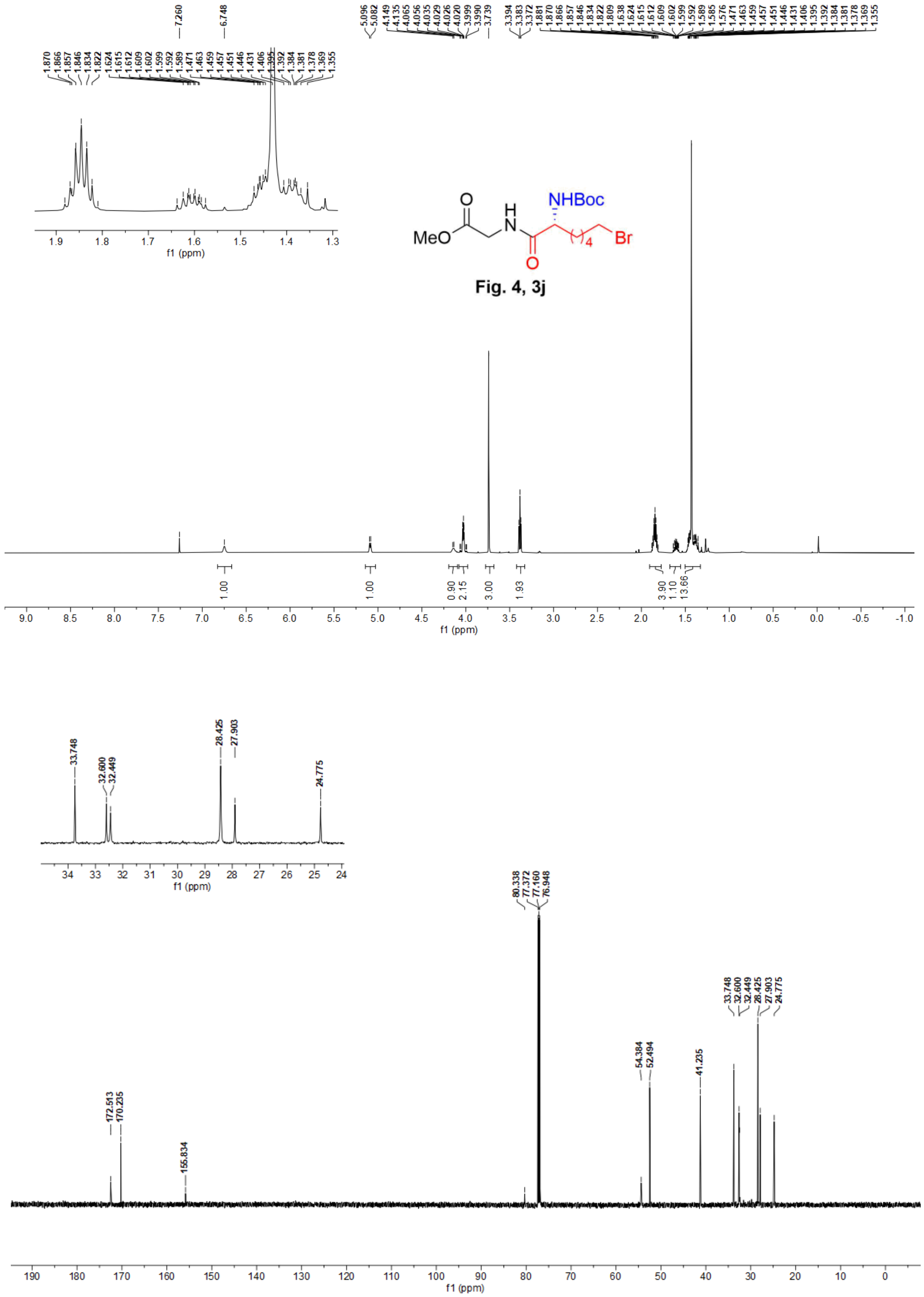

Supplementary Figure 34. ^{1}H NMR and ^{13}C NMR spectra of compound **3j**

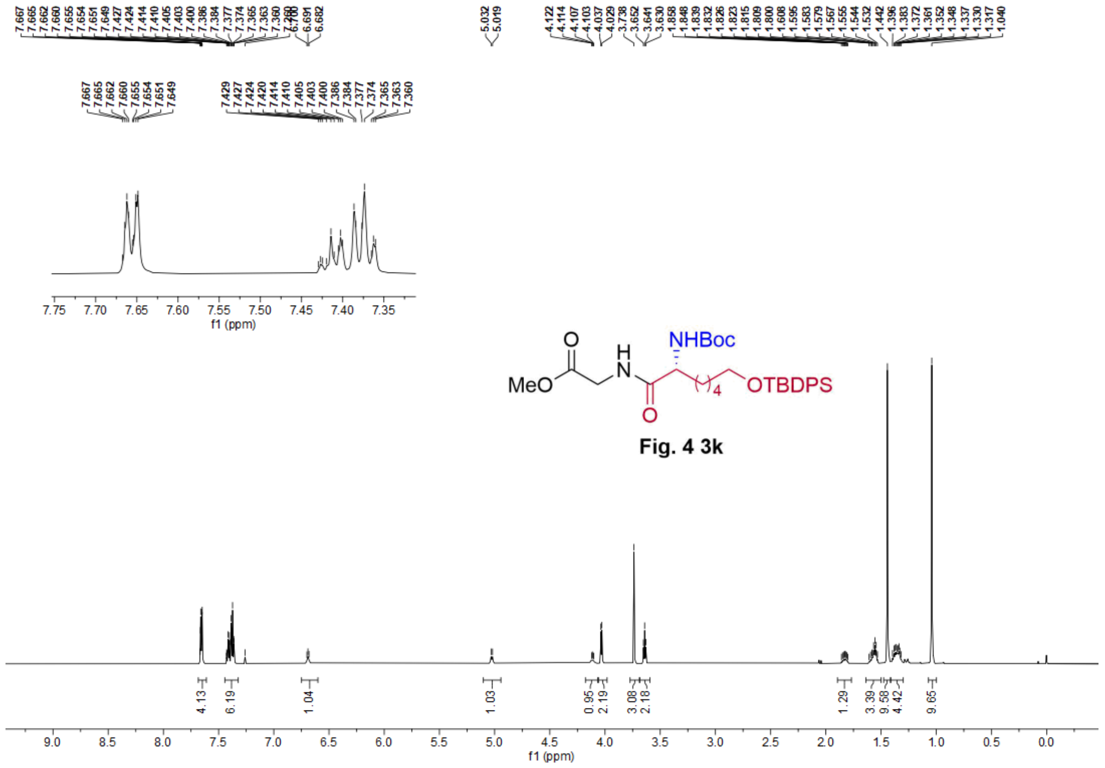

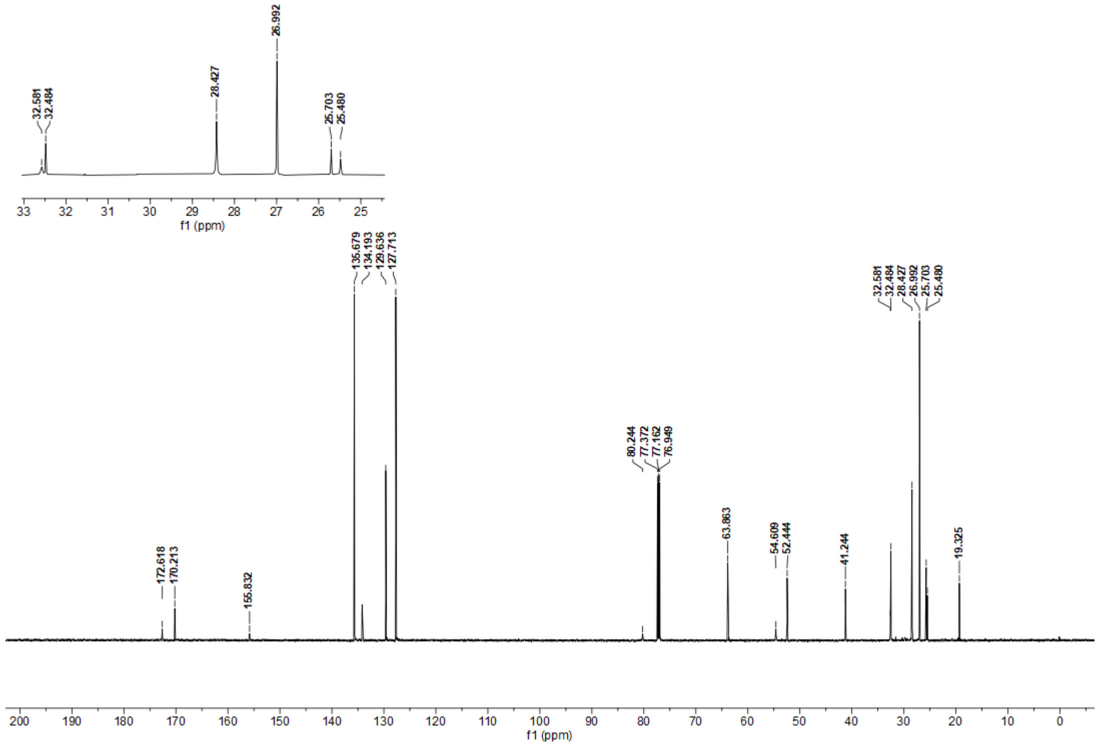

Supplementary Figure 35. ^{1}H NMR and ^{13}C NMR spectra of compound **3k**

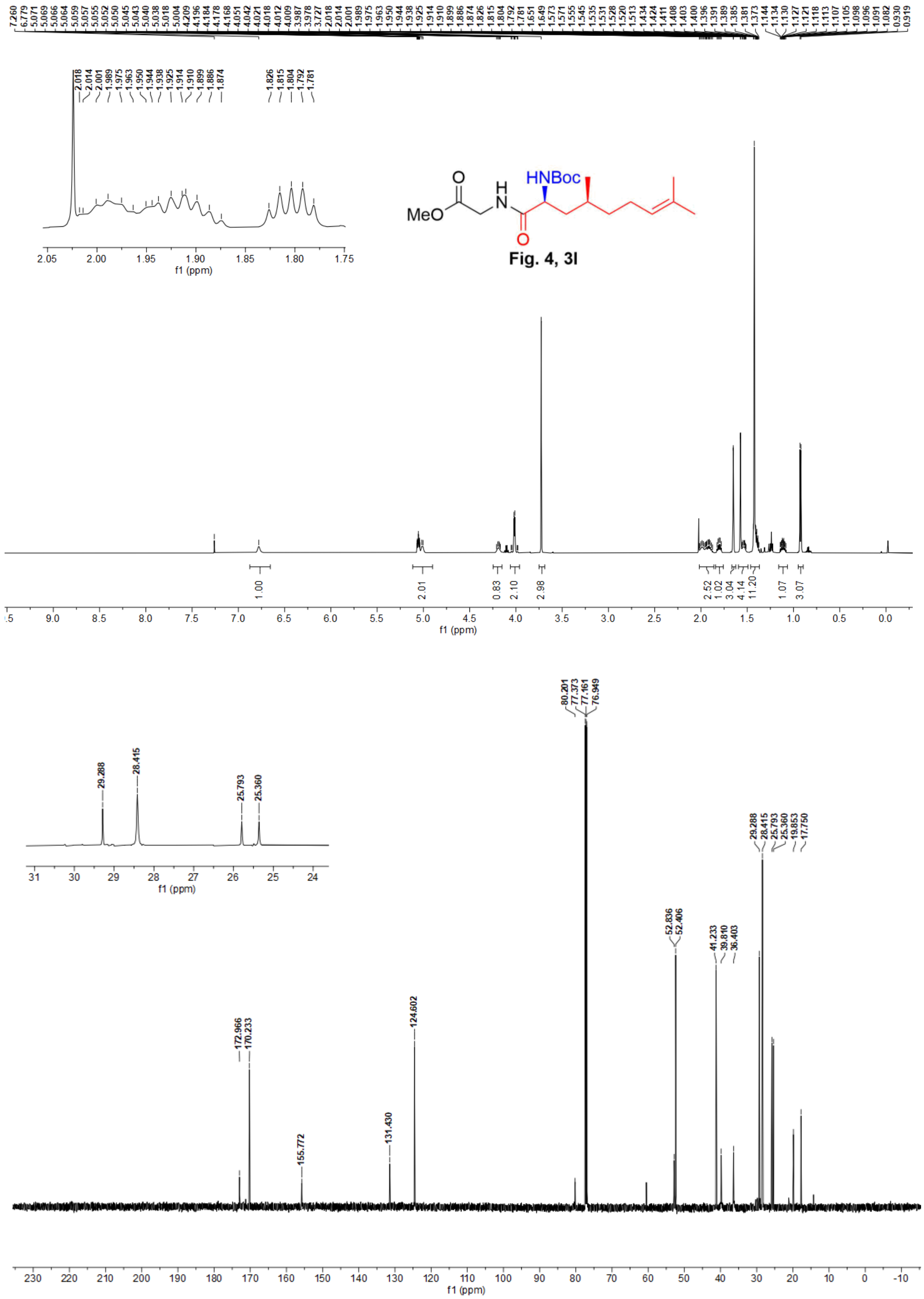

Supplementary Figure 36. ^{1}H NMR and ^{13}C NMR spectra of compound **3l**

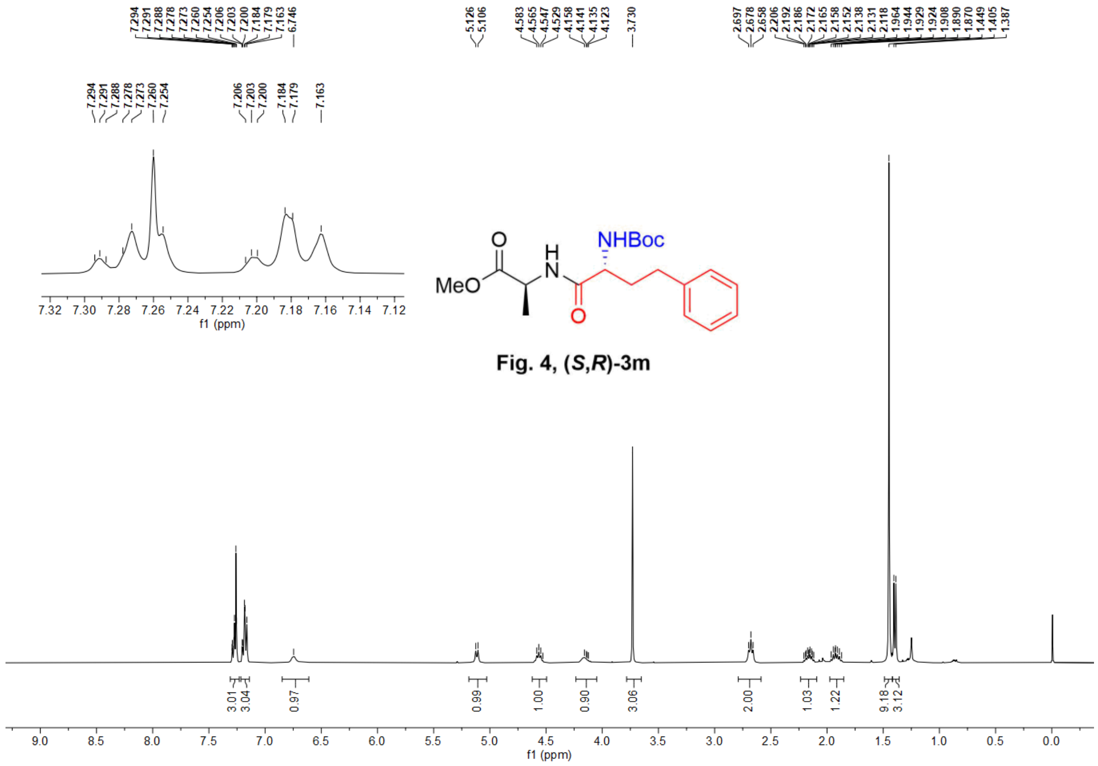

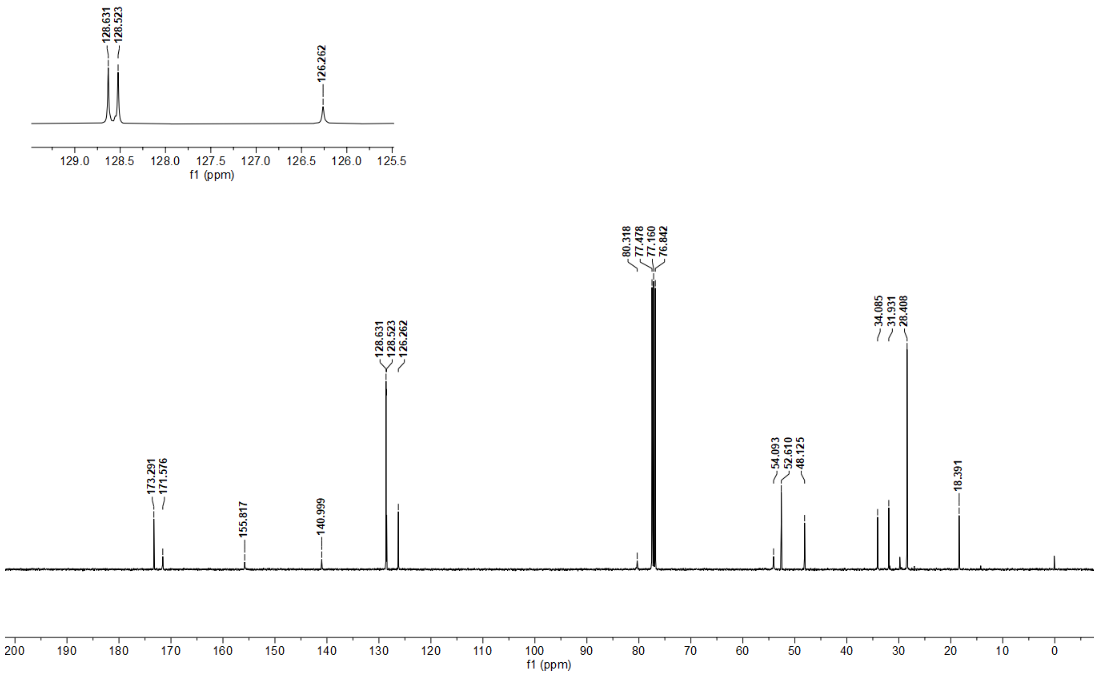

Supplementary Figure 37. ^{1}H NMR and ^{13}C NMR spectra of compound (*S*,*R*)-**3m**

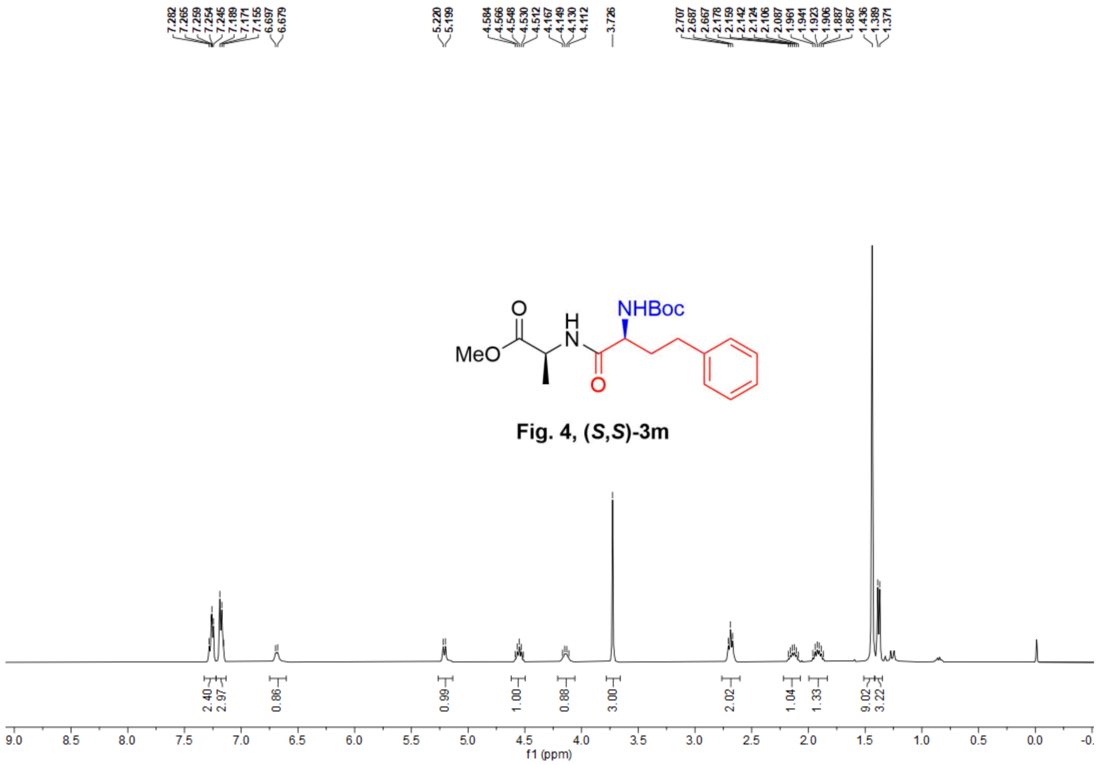

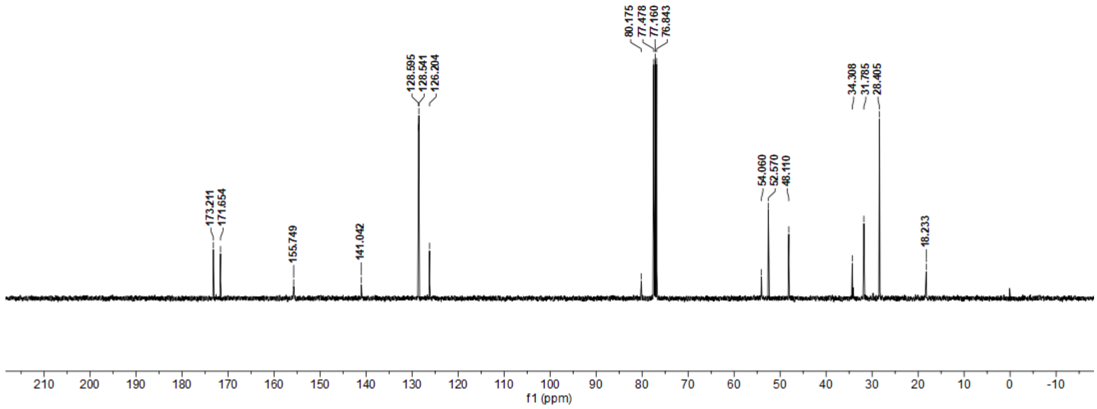

Supplementary Figure 38. ^{1}H NMR and ^{13}C NMR spectra of compound (*S*,*S*)-**3m**

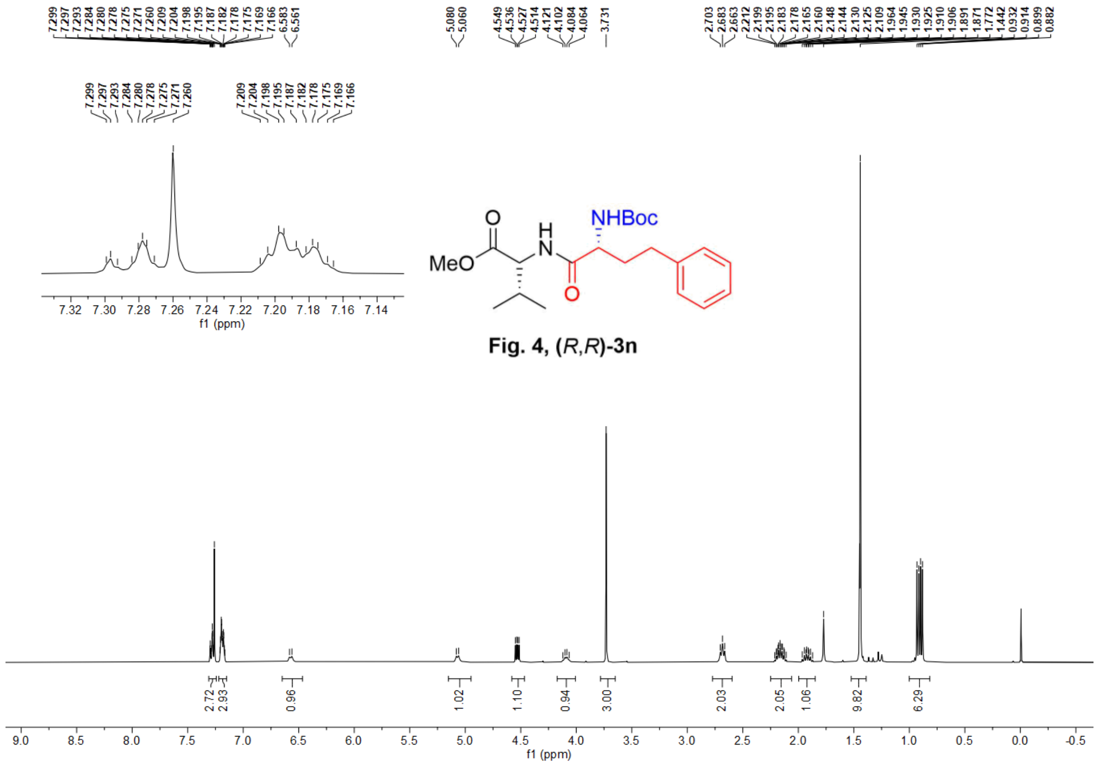

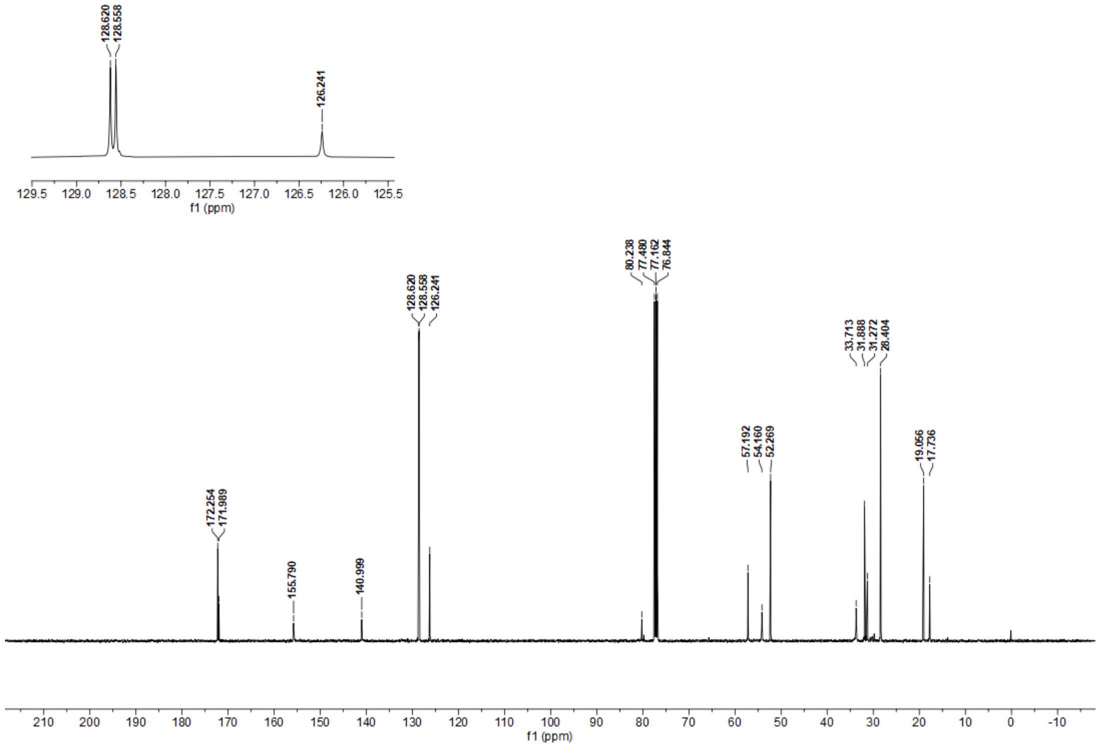

Supplementary Figure 39. ^{1}H NMR and ^{13}C NMR spectra of compound (*R*,*R*)-**3n**

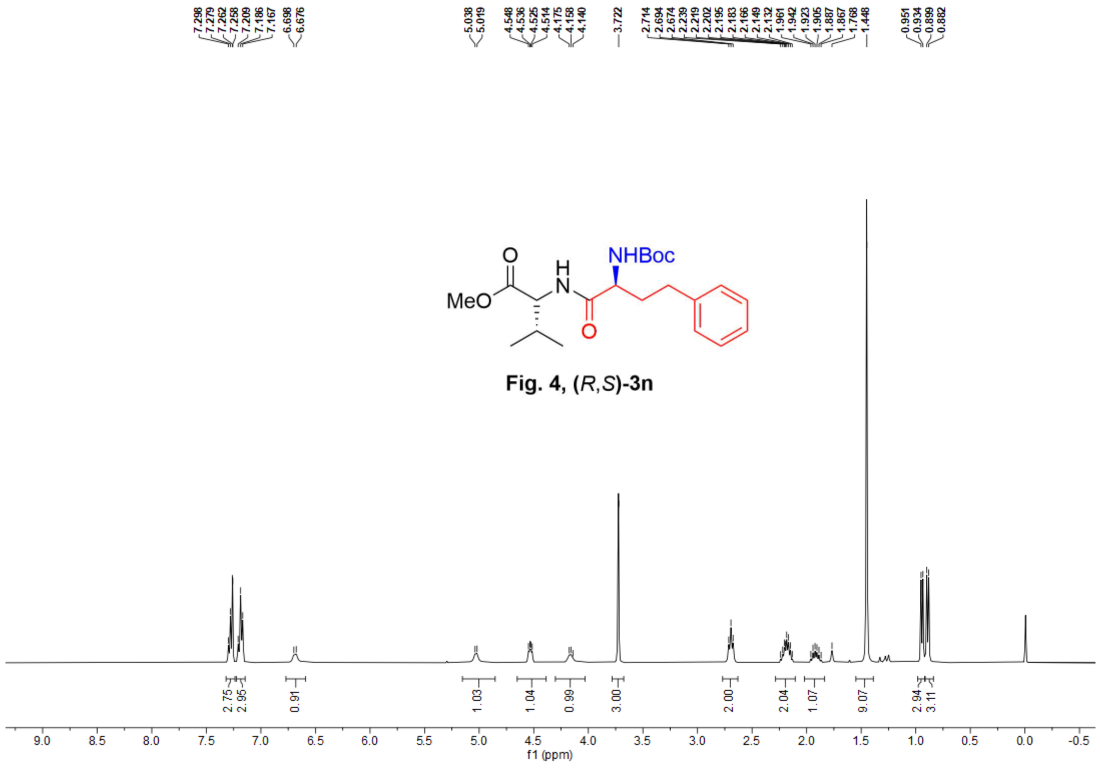

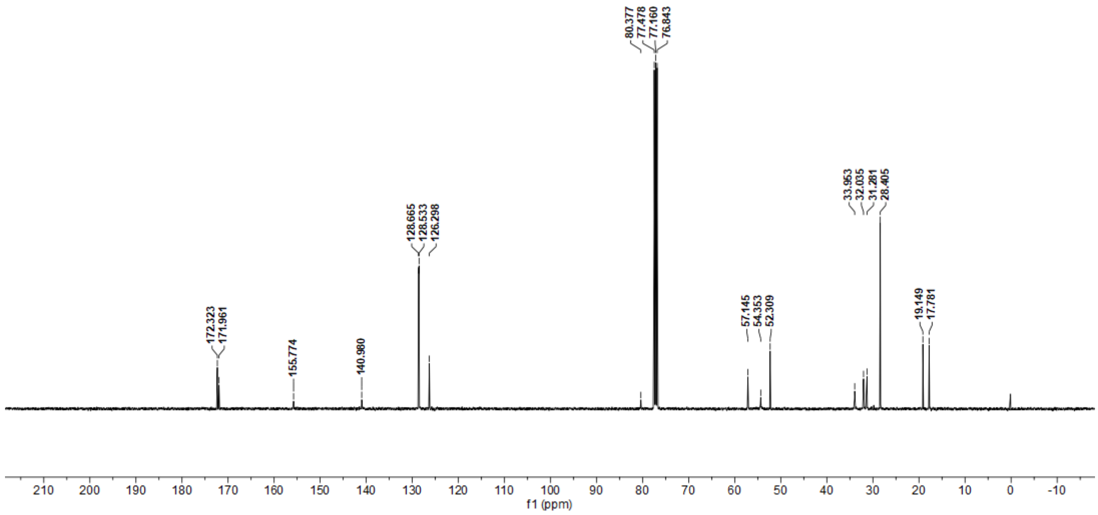

Supplementary Figure 40. ^{1}H NMR and ^{13}C NMR spectra of compound (*R*,*S*)-**3n**

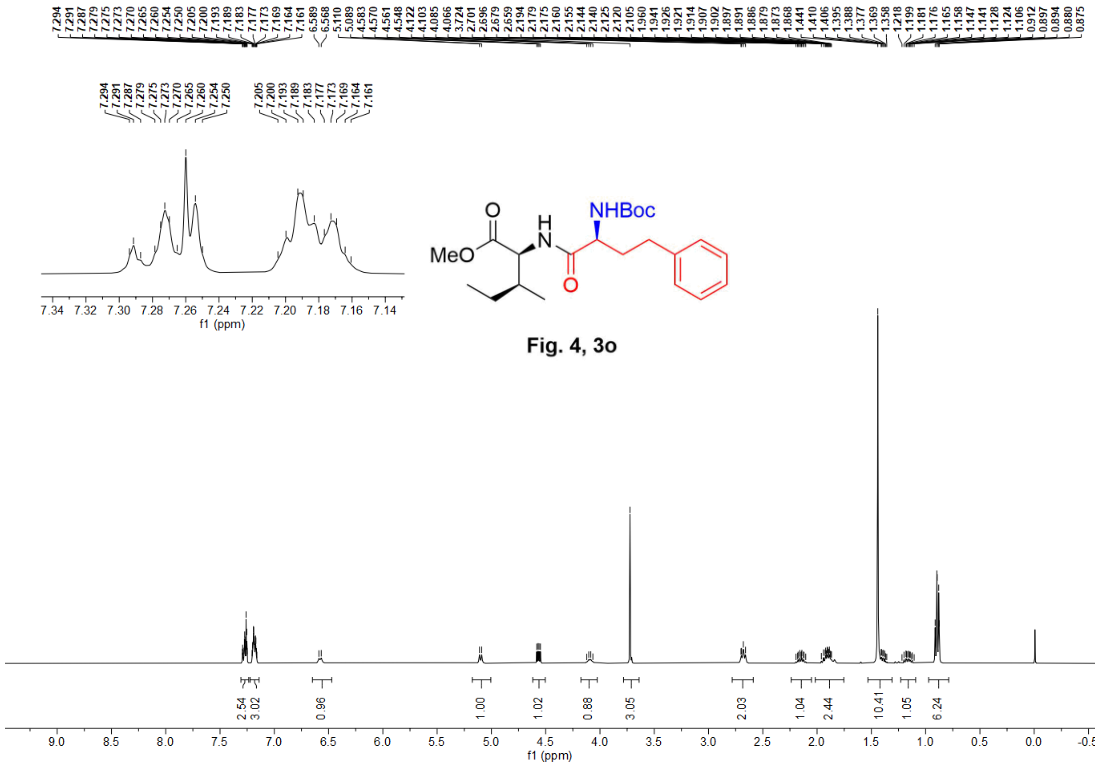

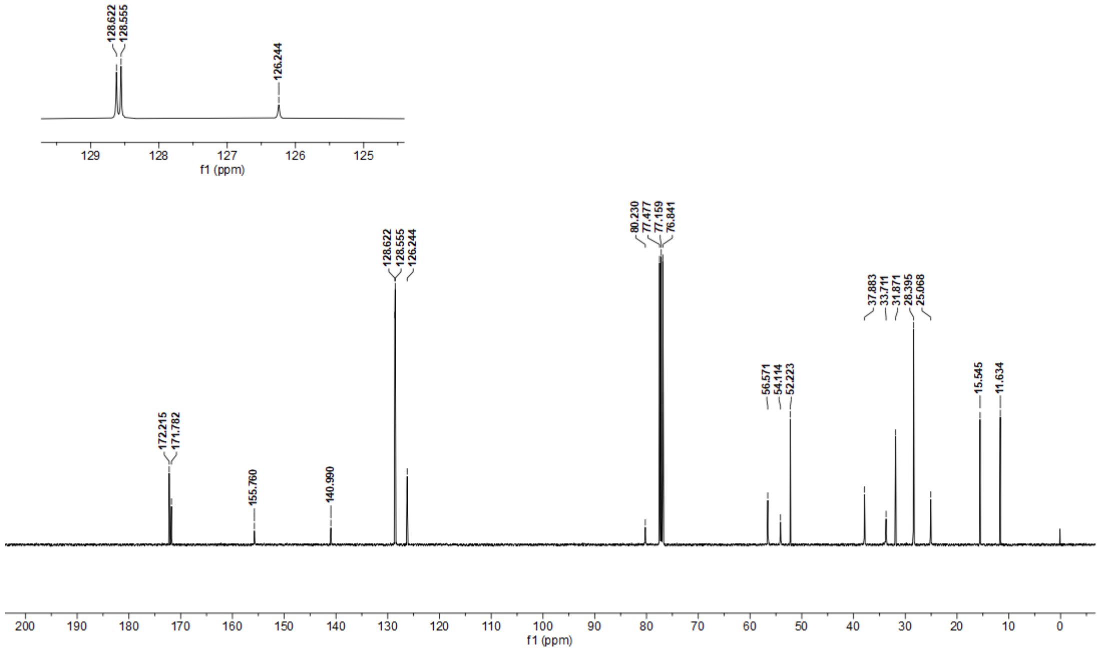

Supplementary Figure 41. ^{1}H NMR and ^{13}C NMR spectra of compound **3o**

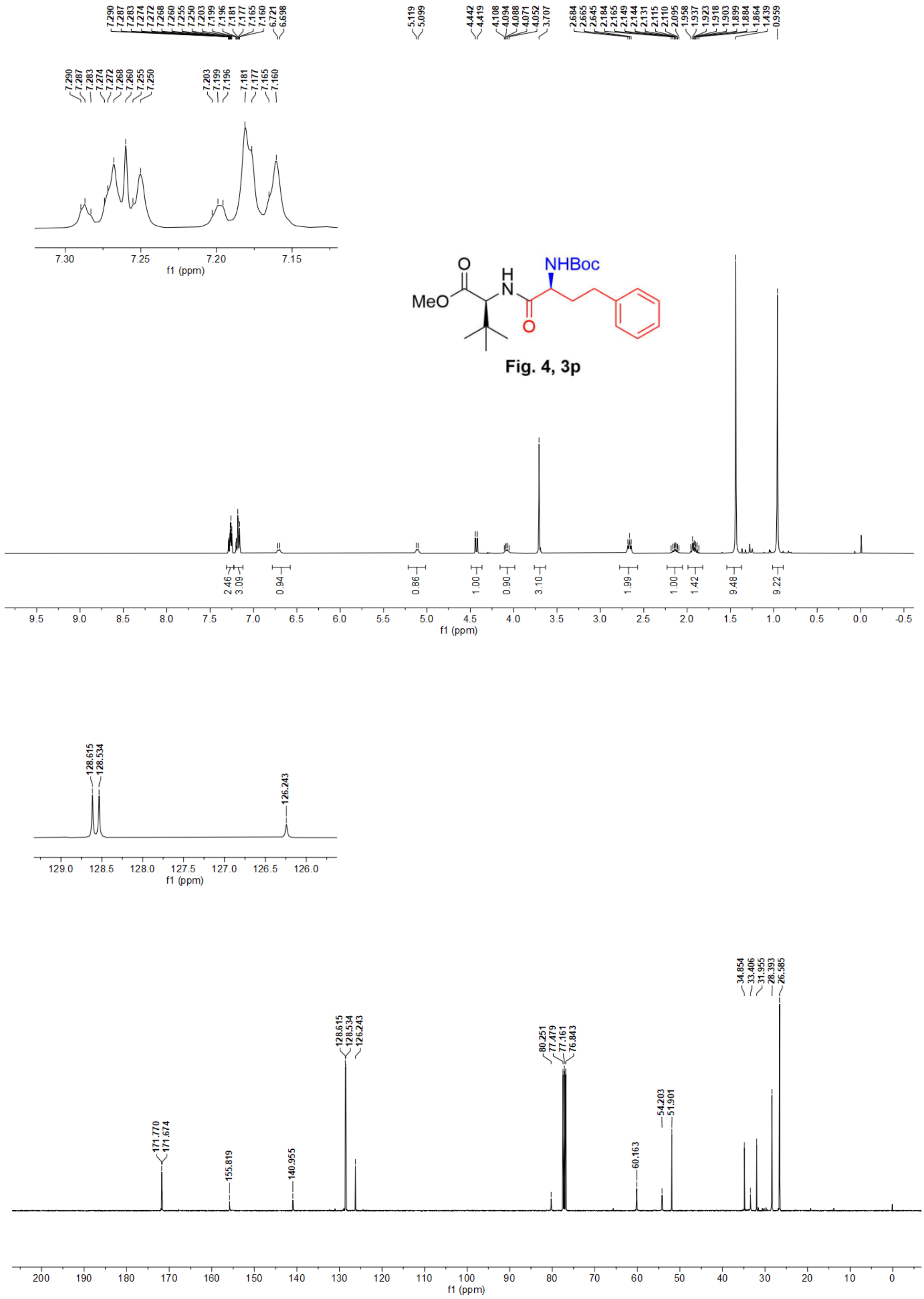

Supplementary Figure 42. ^{1}H NMR and ^{13}C NMR spectra of compound **3p**

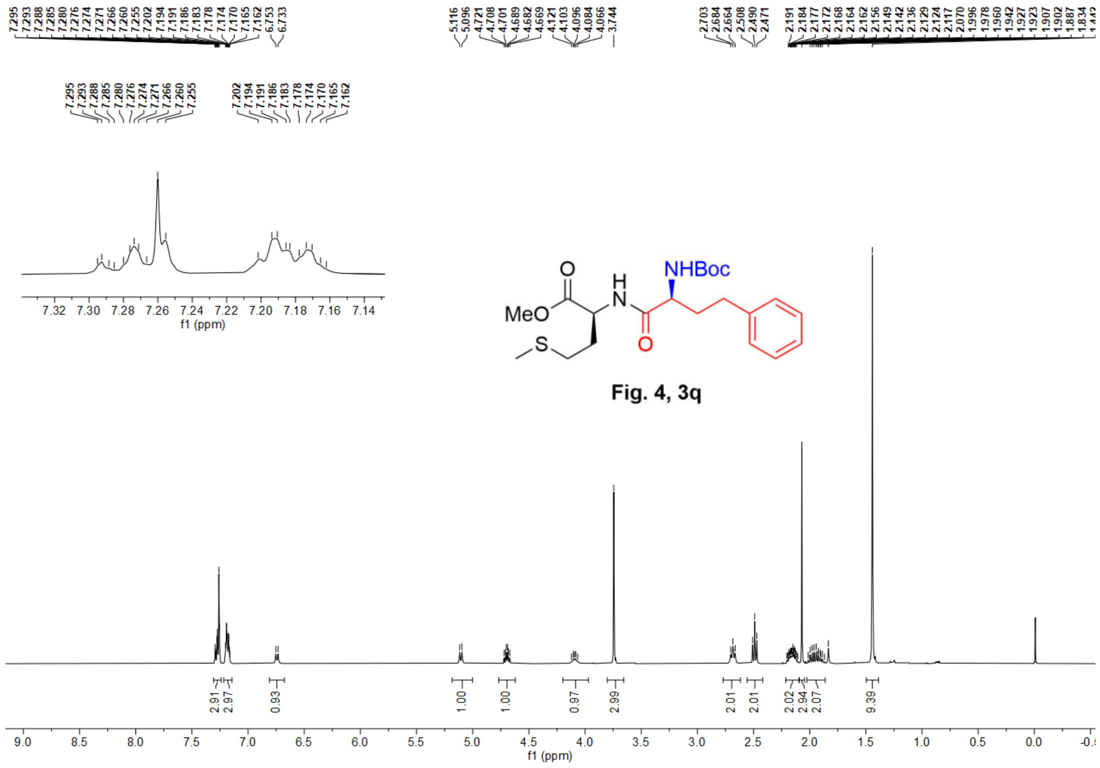

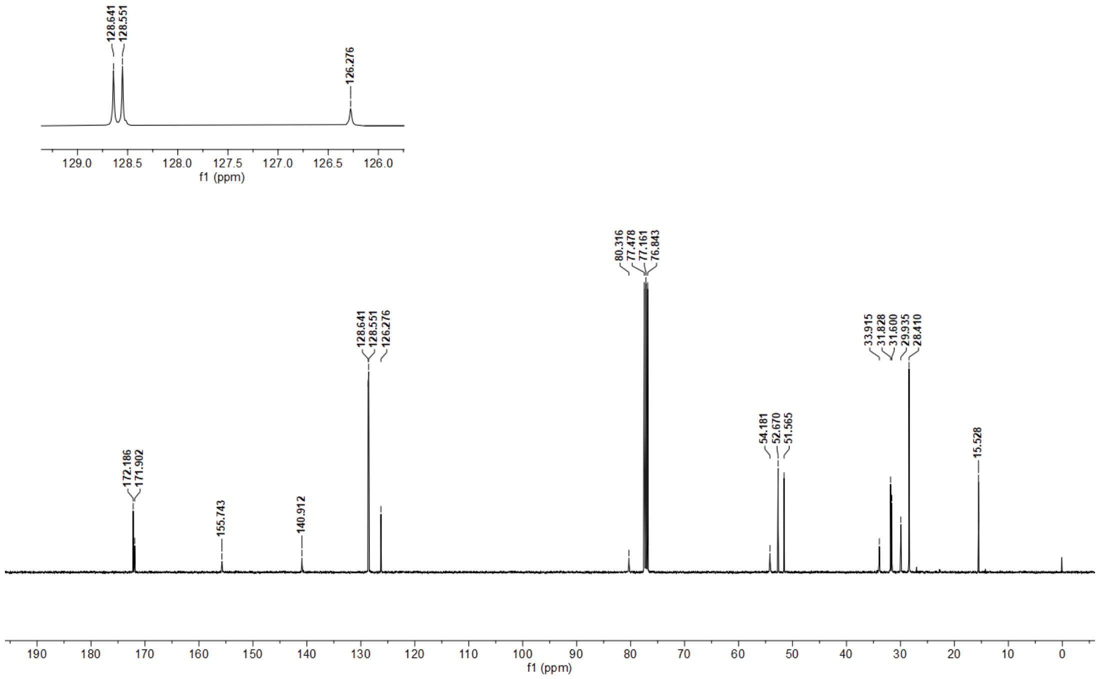

Supplementary Figure 43. ^{1}H NMR and ^{13}C NMR spectra of compound **3q**

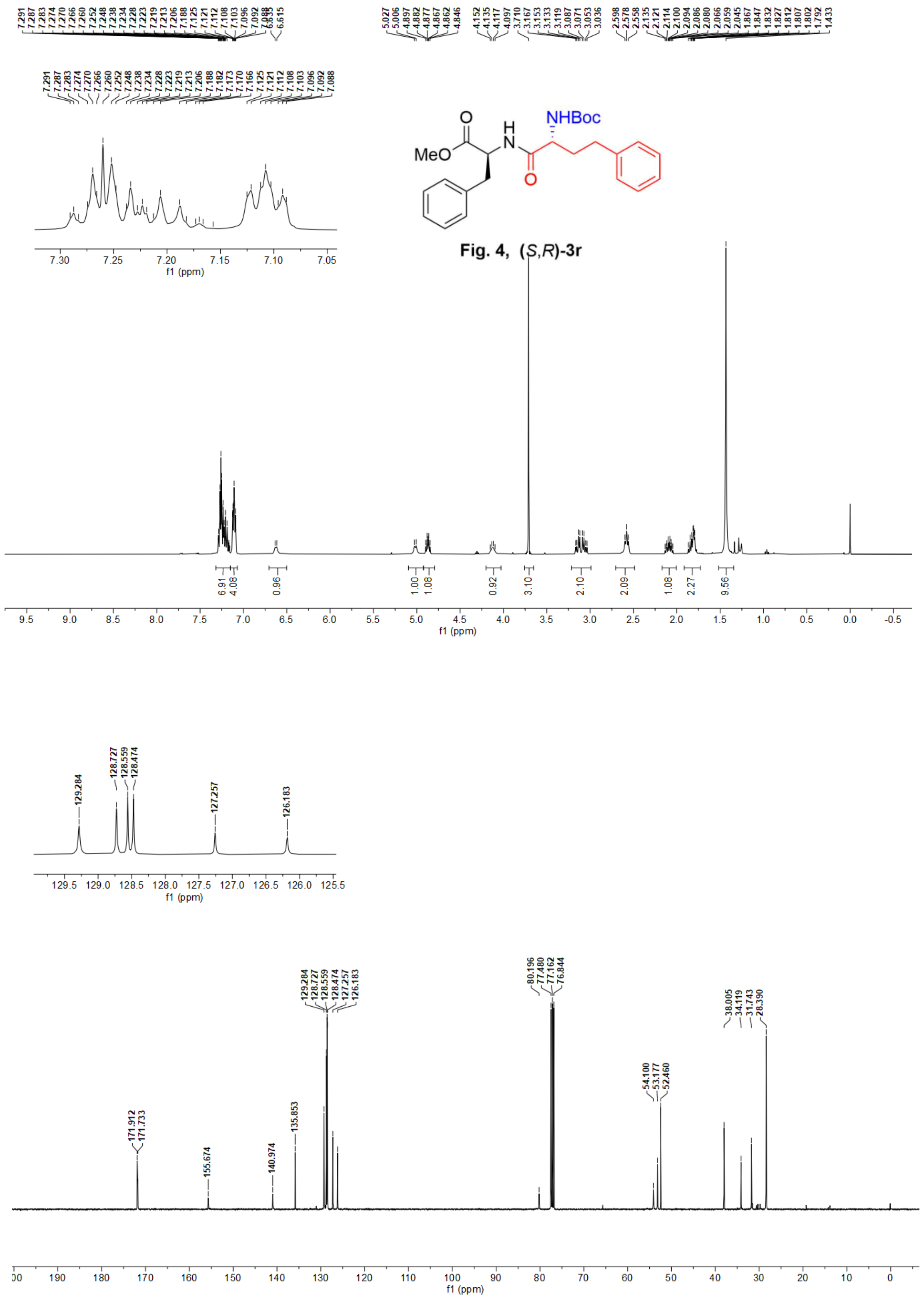

Supplementary Figure 44. ^{1}H NMR and ^{13}C NMR spectra of compound (*S*,*R*)-**3r**

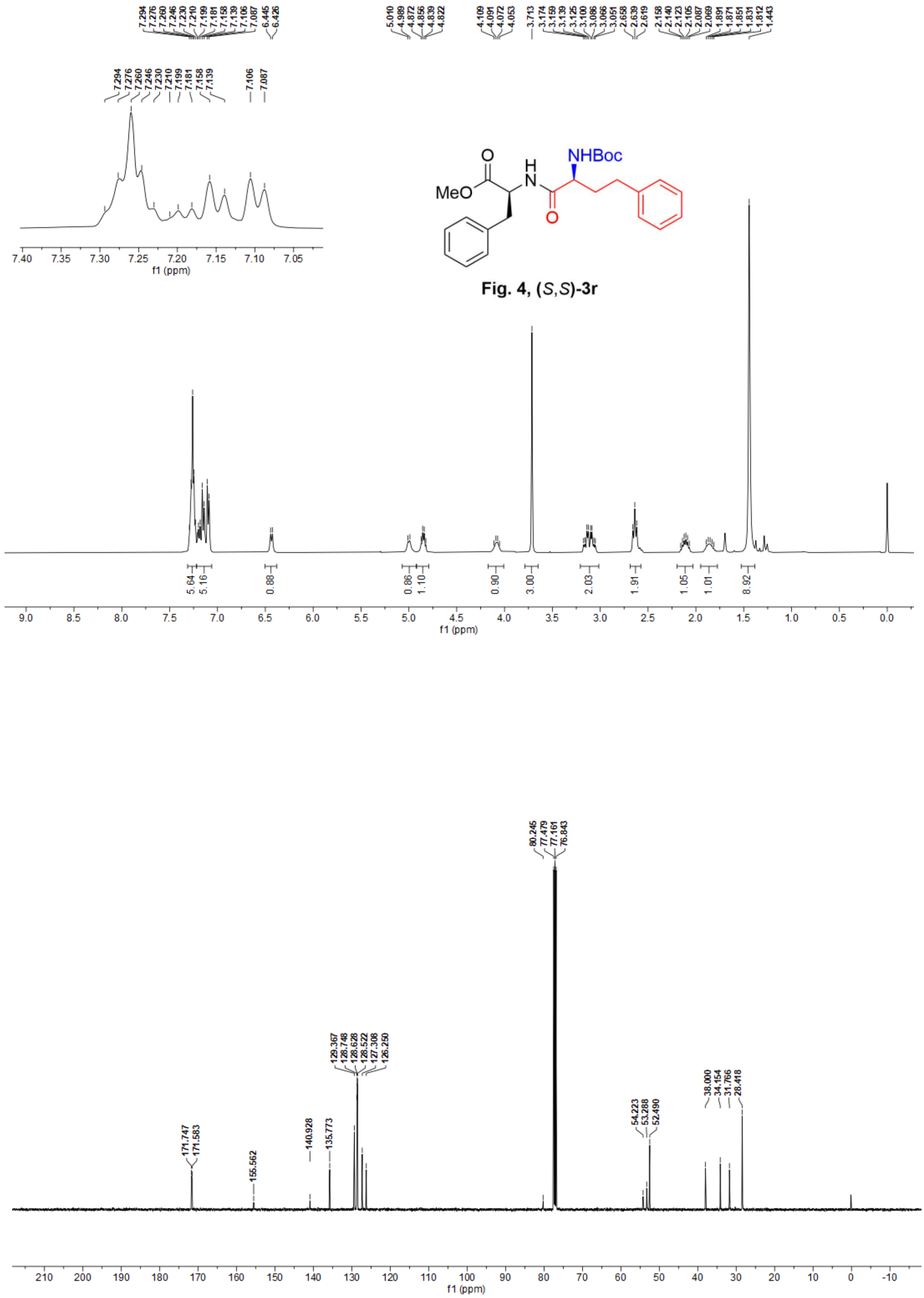

Supplementary Figure 45. ^{1}H NMR and ^{13}C NMR spectra of compound (*S*,*S*)-**3r**

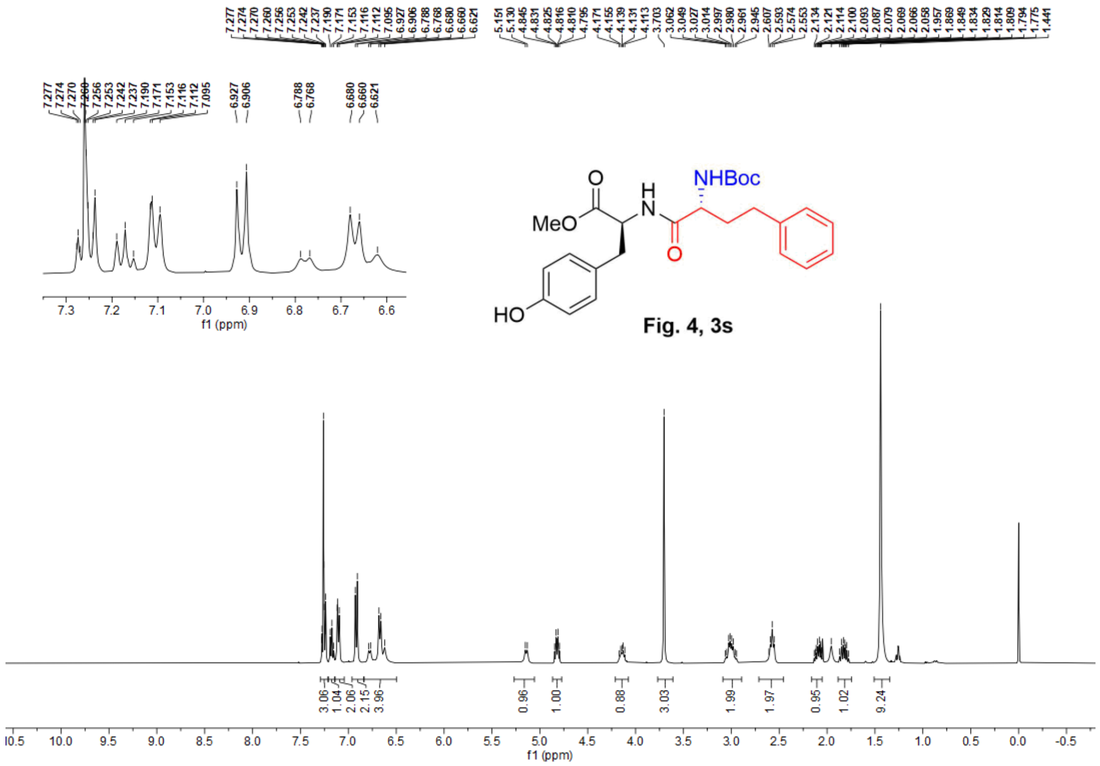

Fig. 4, 3s

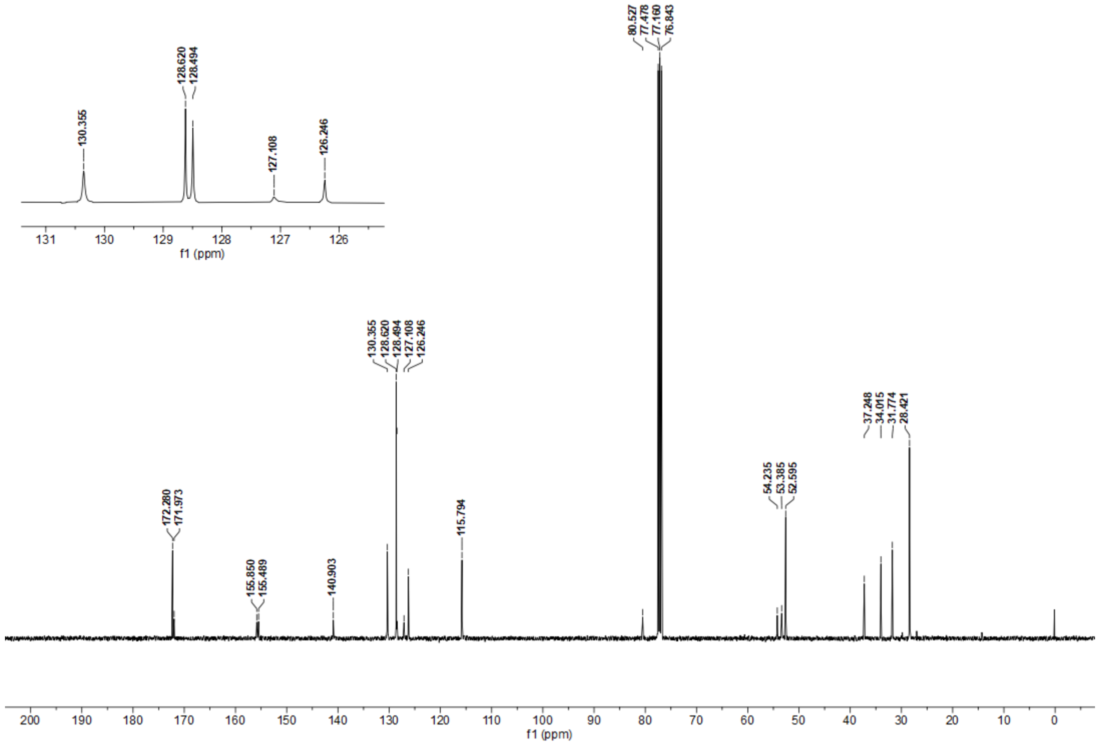

Supplementary Figure 46. ^{1}H NMR and ^{13}C NMR spectra of compound **3s**

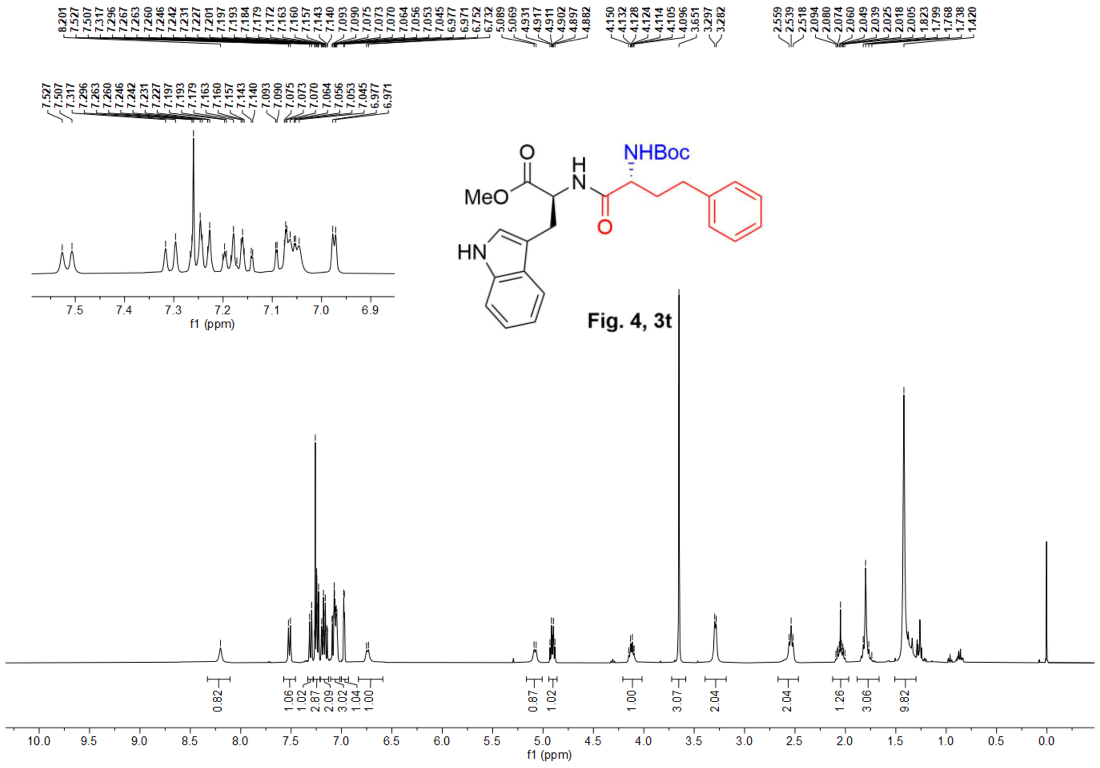

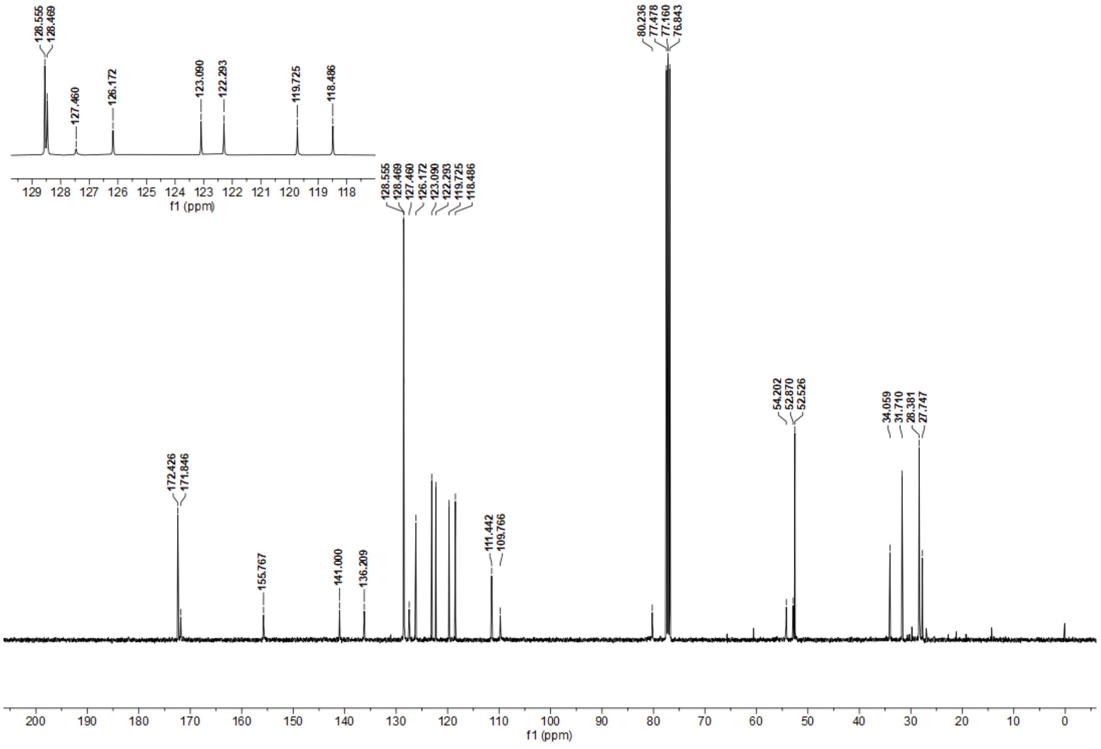

Supplementary Figure 47. ^{1}H NMR and ^{13}C NMR spectra of compound **3t**

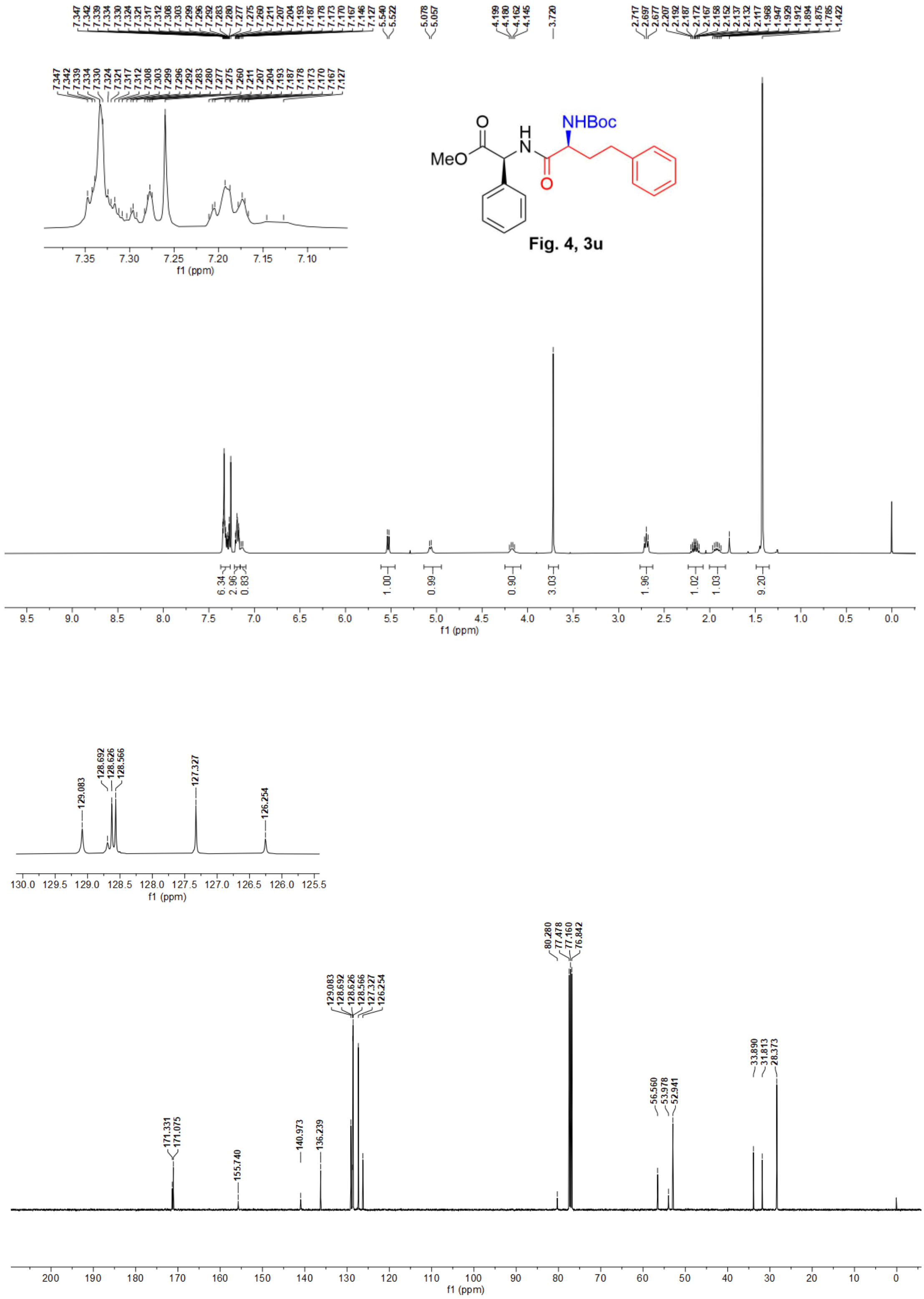

Supplementary Figure 48. ^{1}H NMR and ^{13}C NMR spectra of compound **3u**

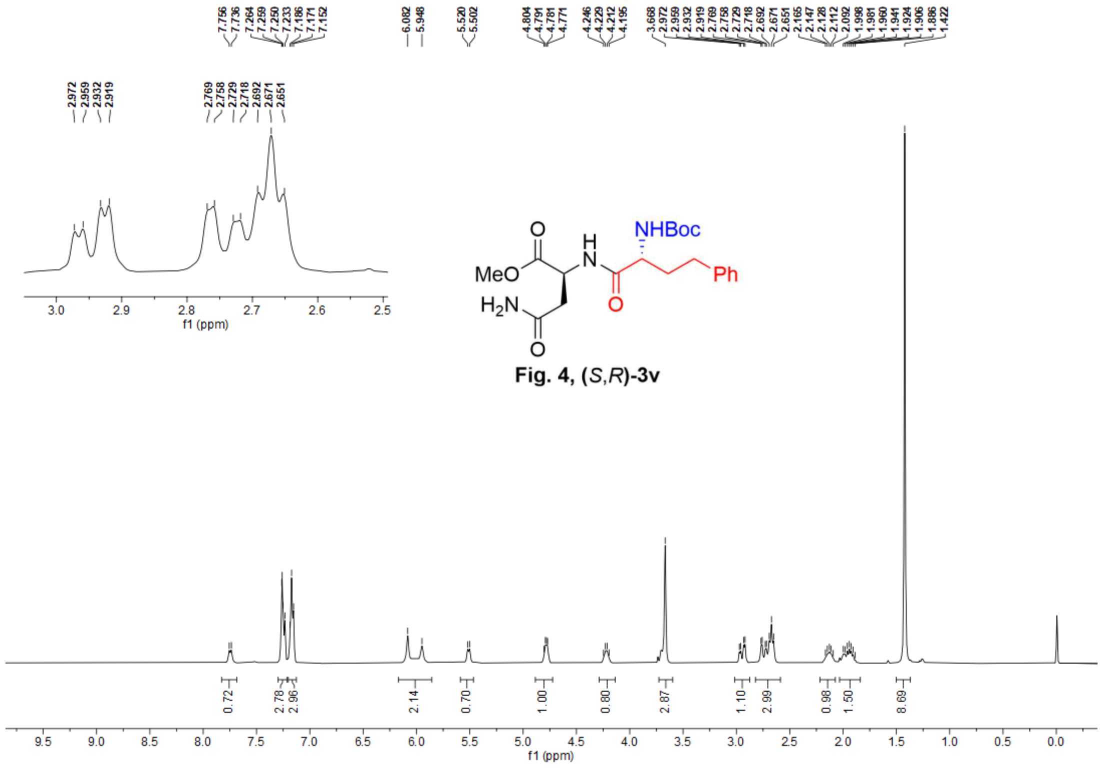

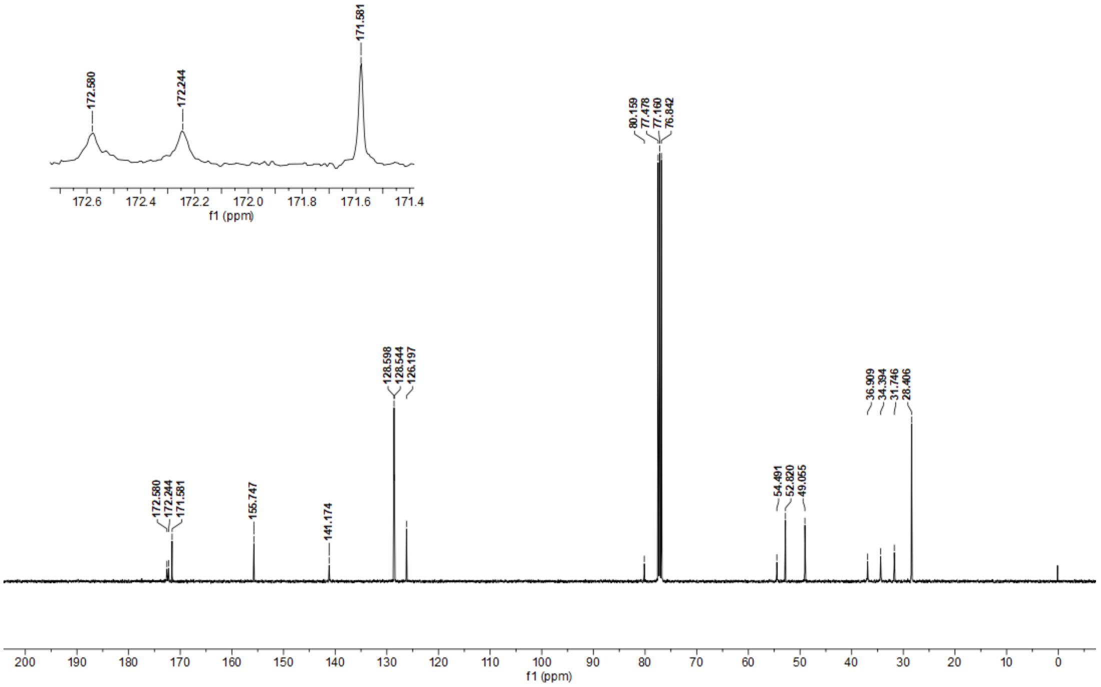

Supplementary Figure 49. ^{1}H NMR and ^{13}C NMR spectra of compound **(***S***,***R***)-3v**

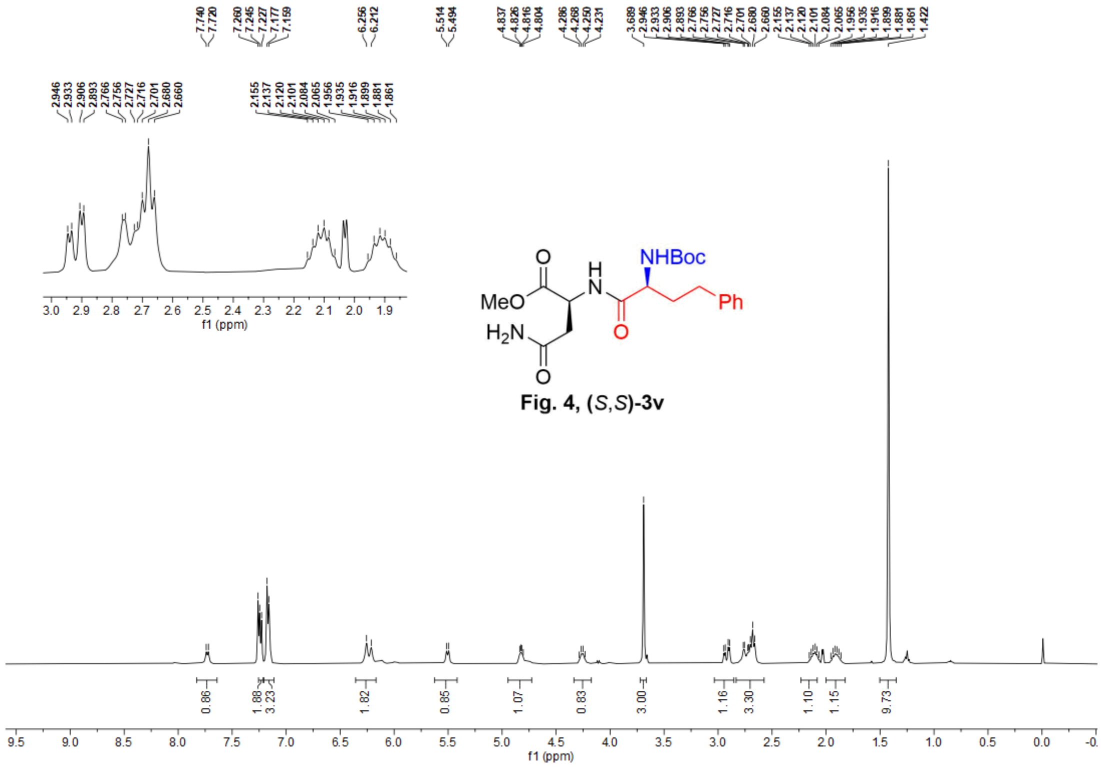

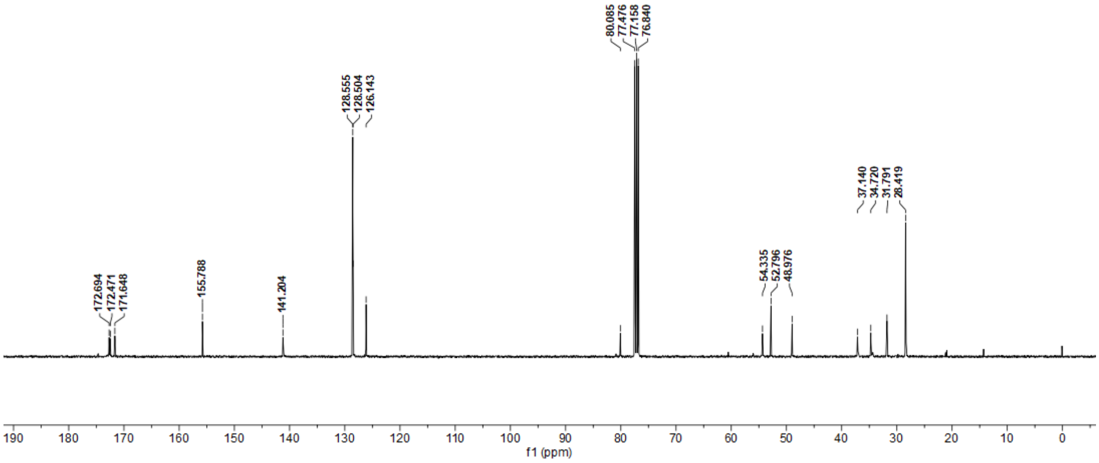

Supplementary Figure 50. ^{1}H NMR and ^{13}C NMR spectra of compound **(*S*,*S*)-3v**

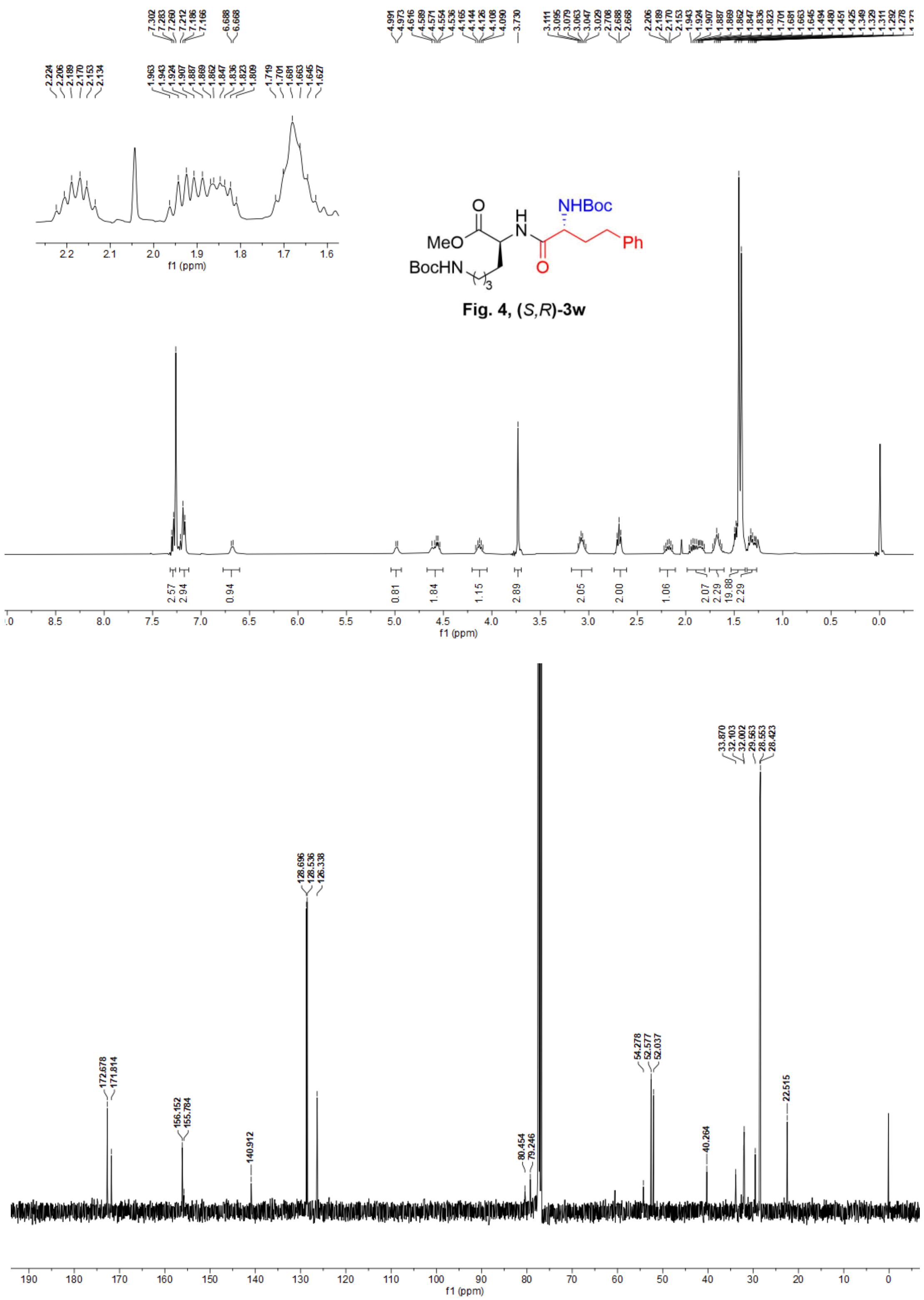

Supplementary Figure 51. ^{1}H NMR and ^{13}C NMR spectra of compound **(*S*,*R*)-3w**

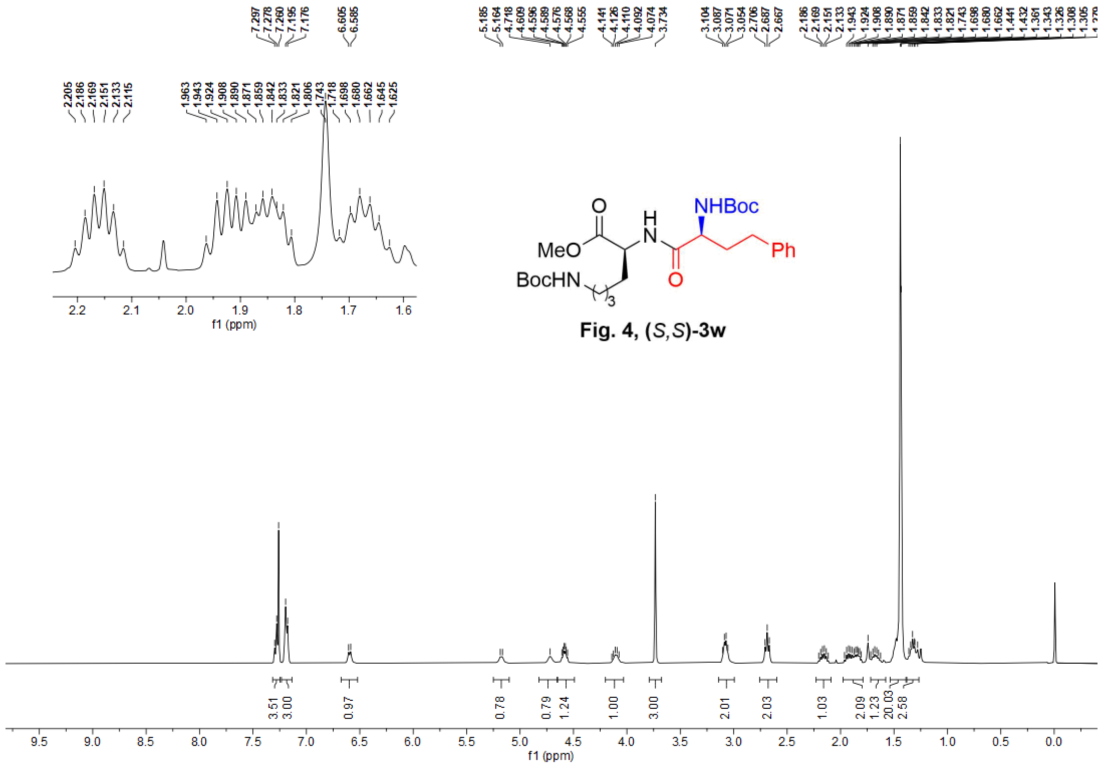

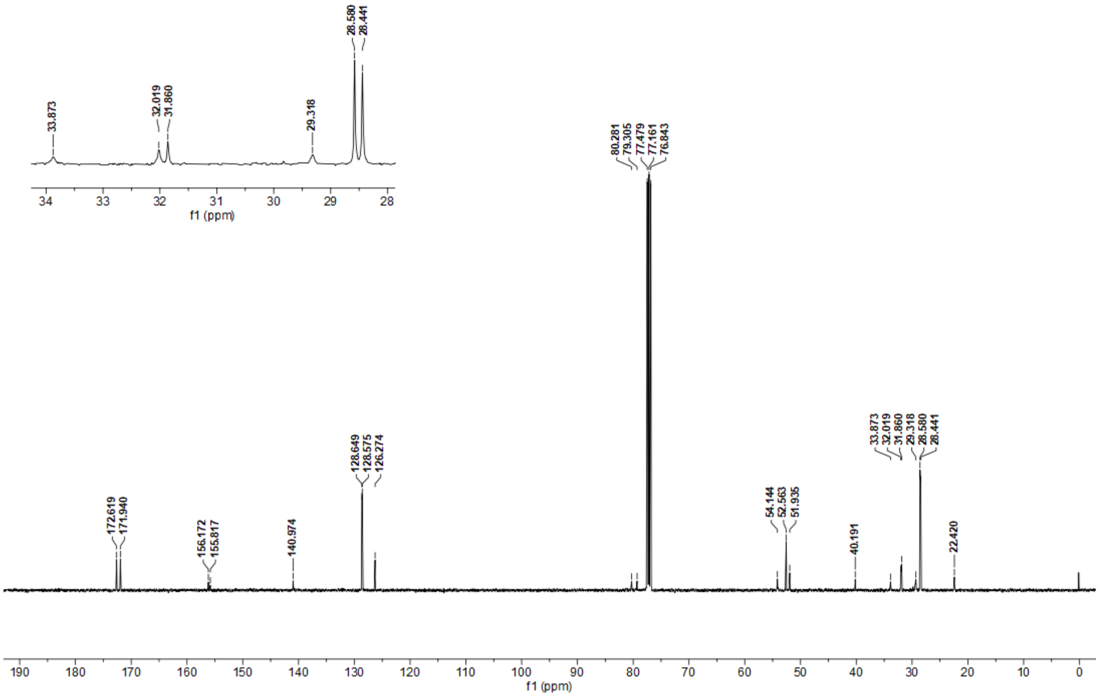

Supplementary Figure 52. ^{1}H NMR and ^{13}C NMR spectra of compound **(*S*,*S*)-3w**

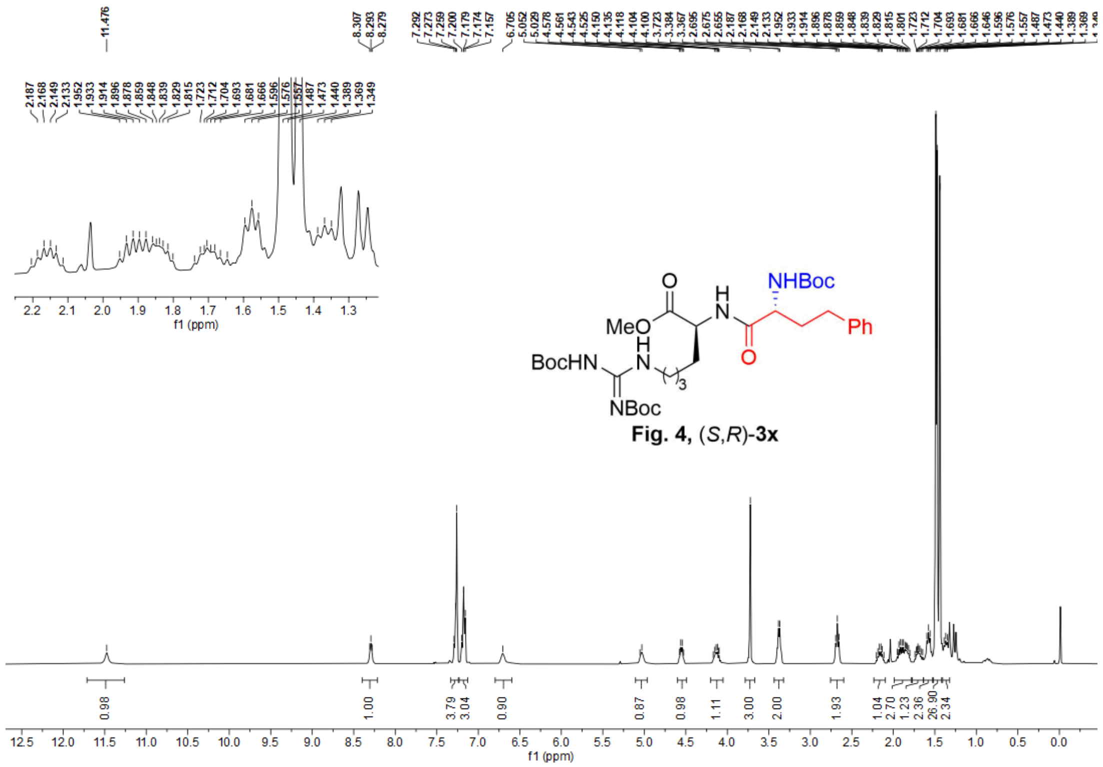

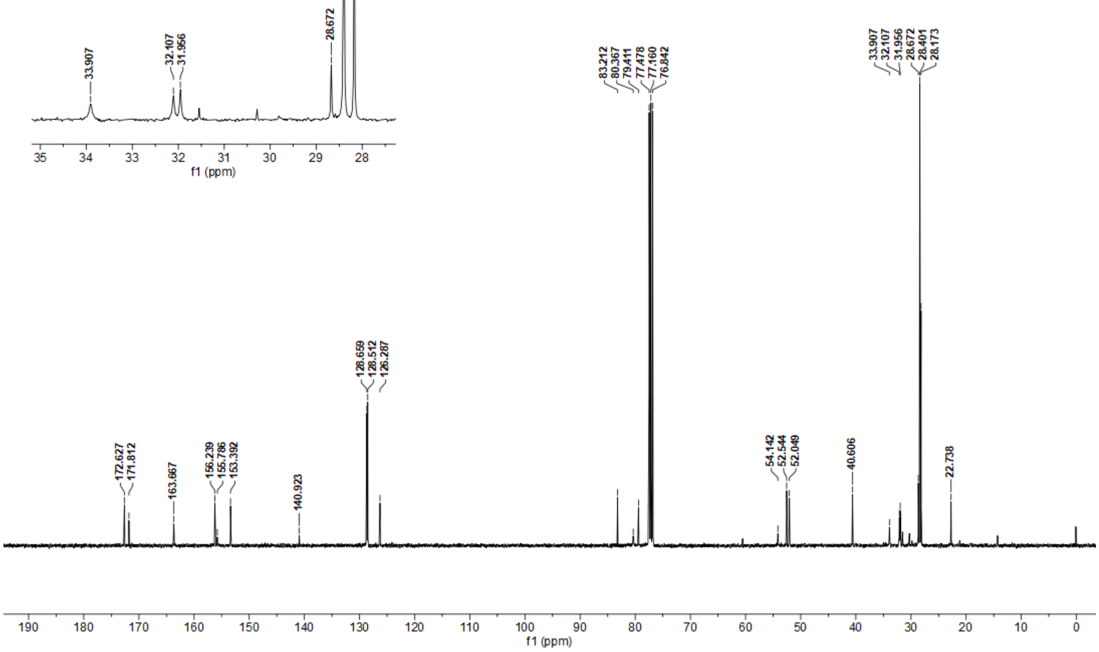

Supplementary Figure 53. ^{1}H NMR and ^{13}C NMR spectra of compound **(*S*,*R*)-3x**

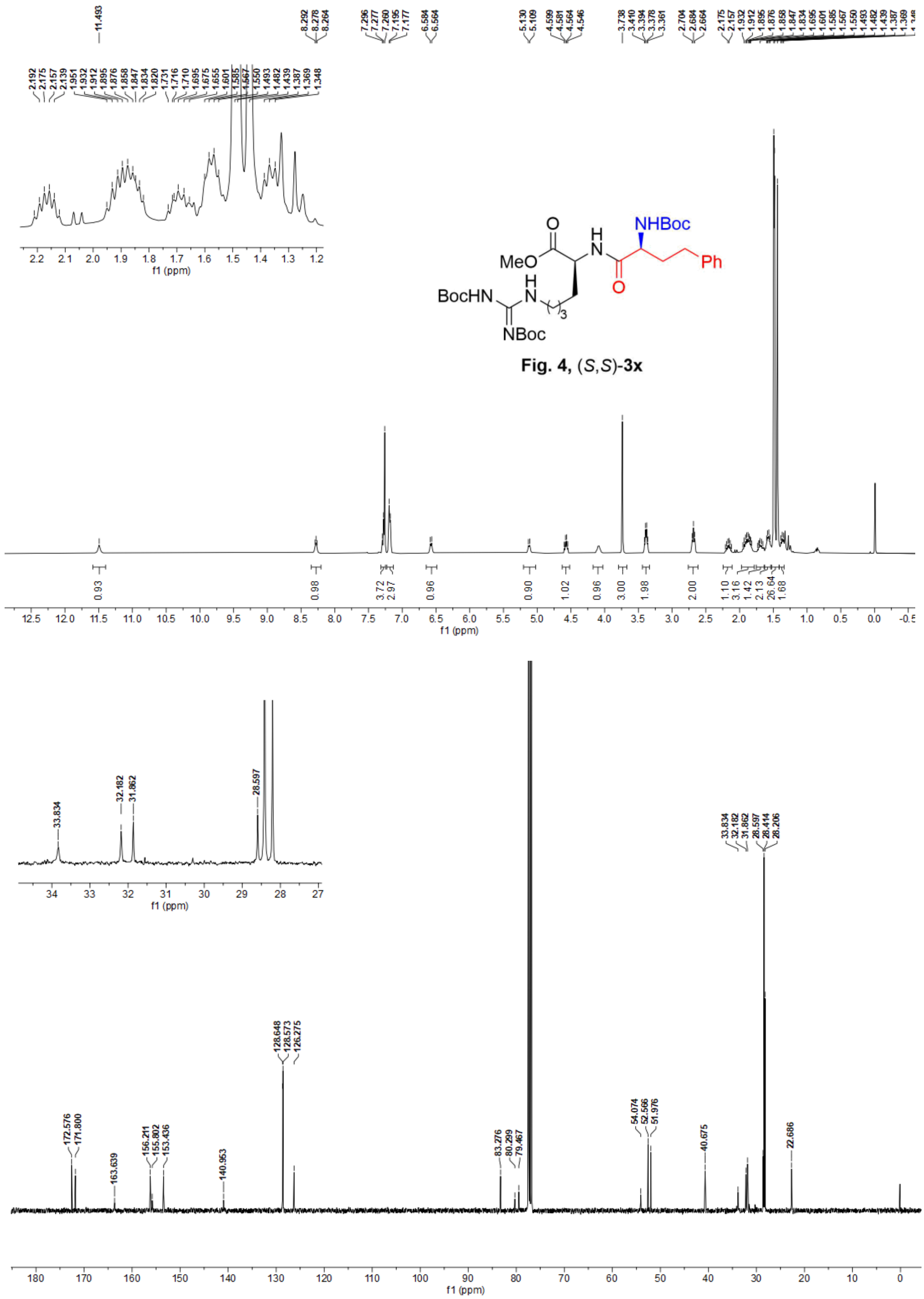

Supplementary Figure 54. ^{1}H NMR and ^{13}C NMR spectra of compound **(*S*,*S*)-3x**

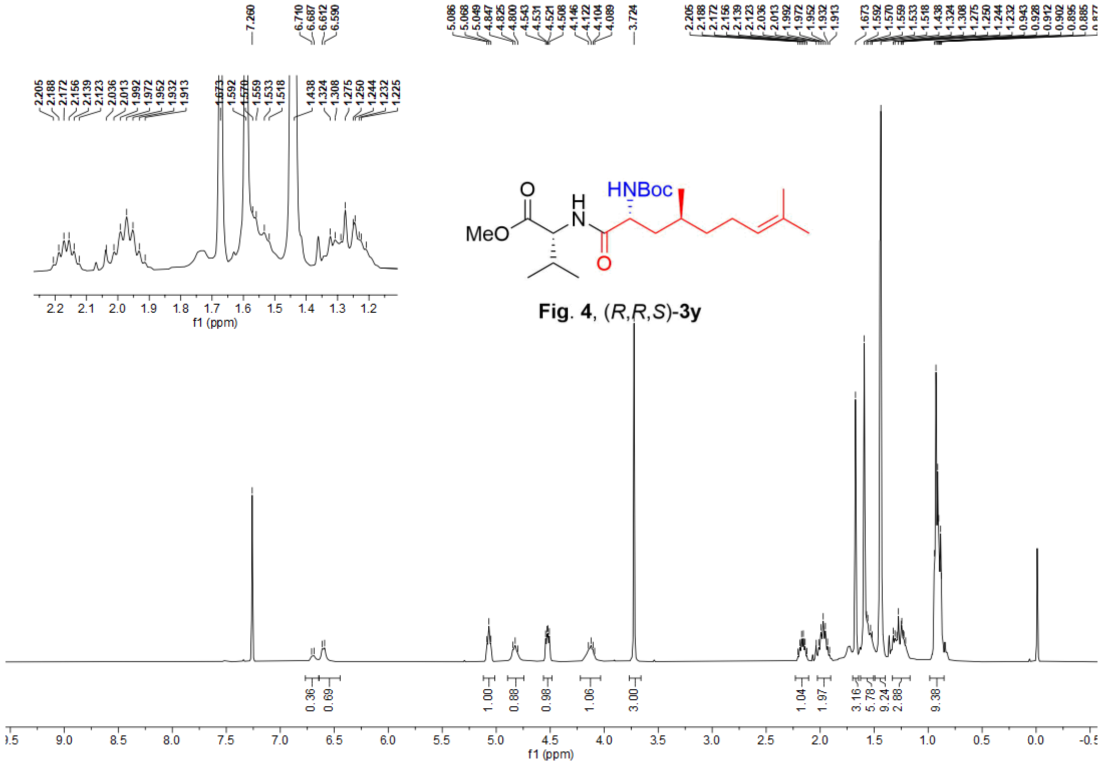

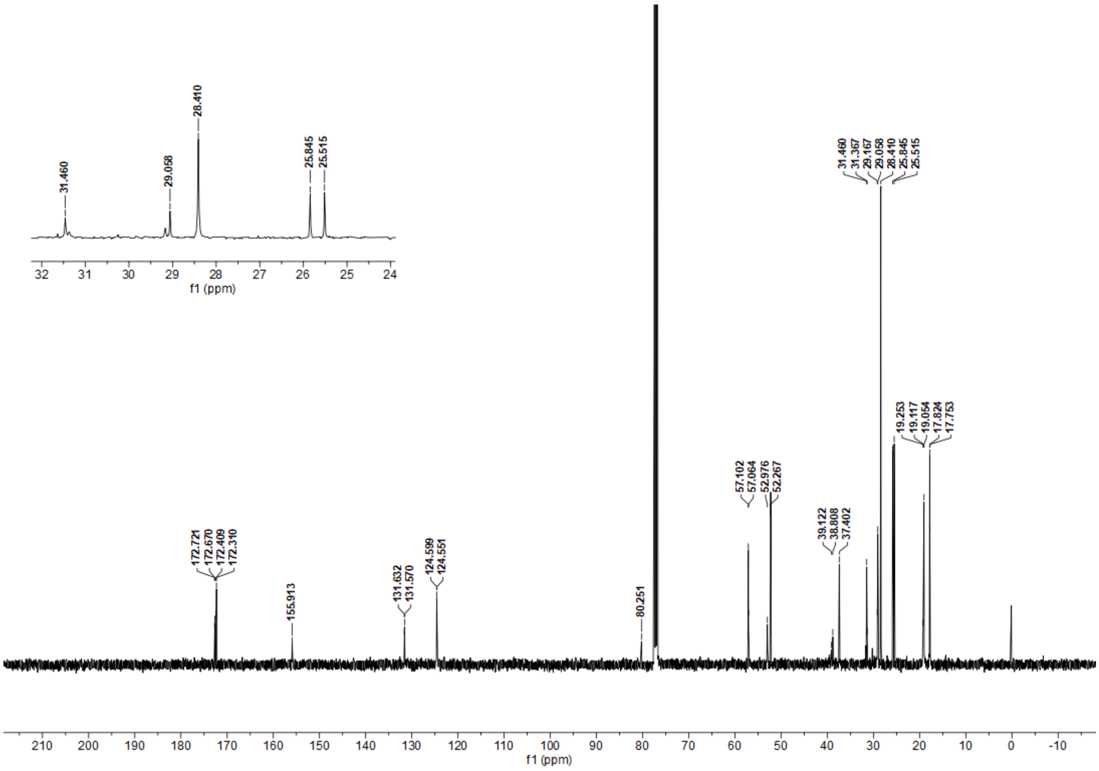

Supplementary Figure 55. ^{1}H NMR and ^{13}C NMR spectra of compound (*R*,*R*,*S*)**-3y**

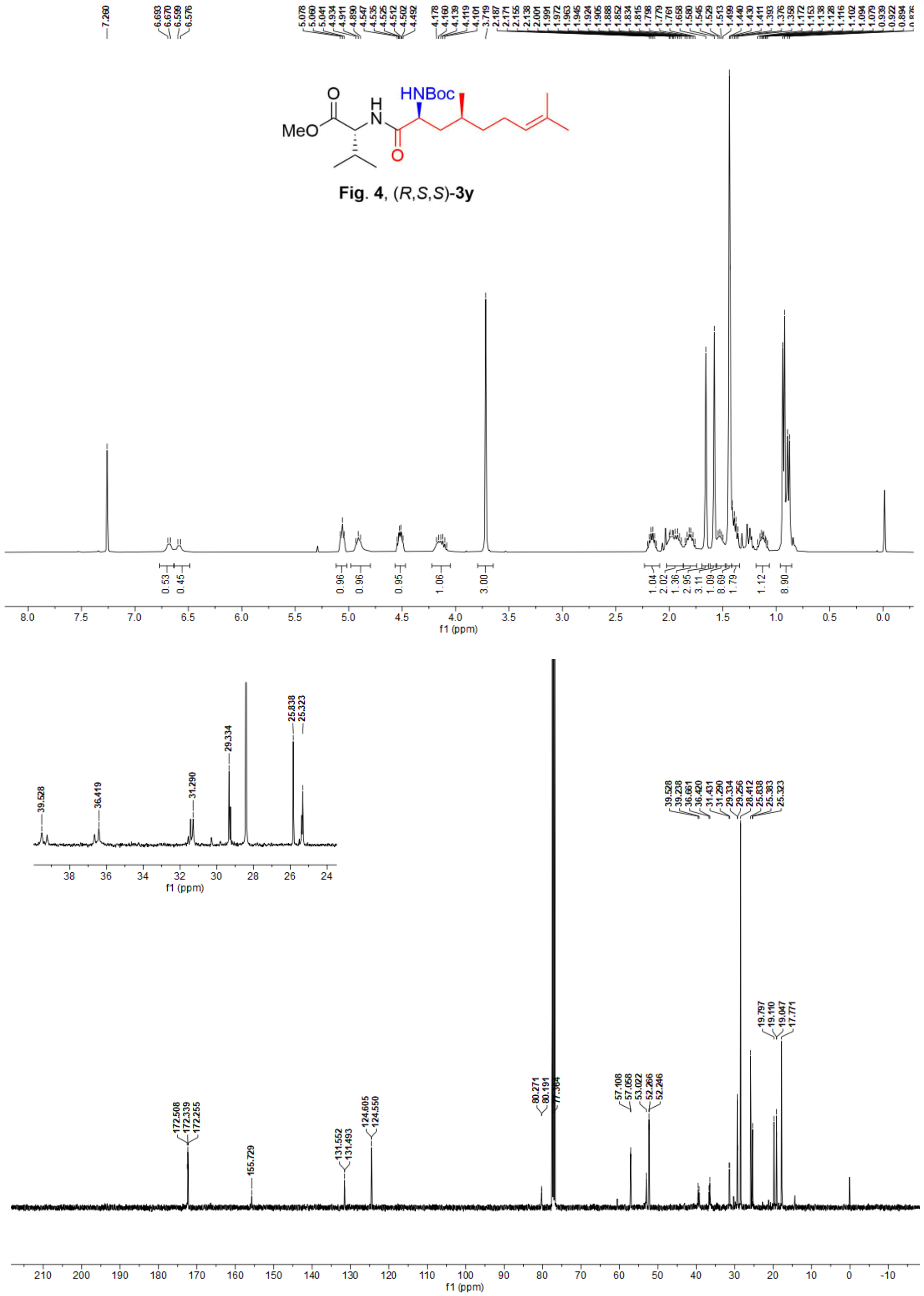

Supplementary Figure 56. ^{1}H NMR and ^{13}C NMR spectra of compound (*R*,*S*,*S*)**-3y**

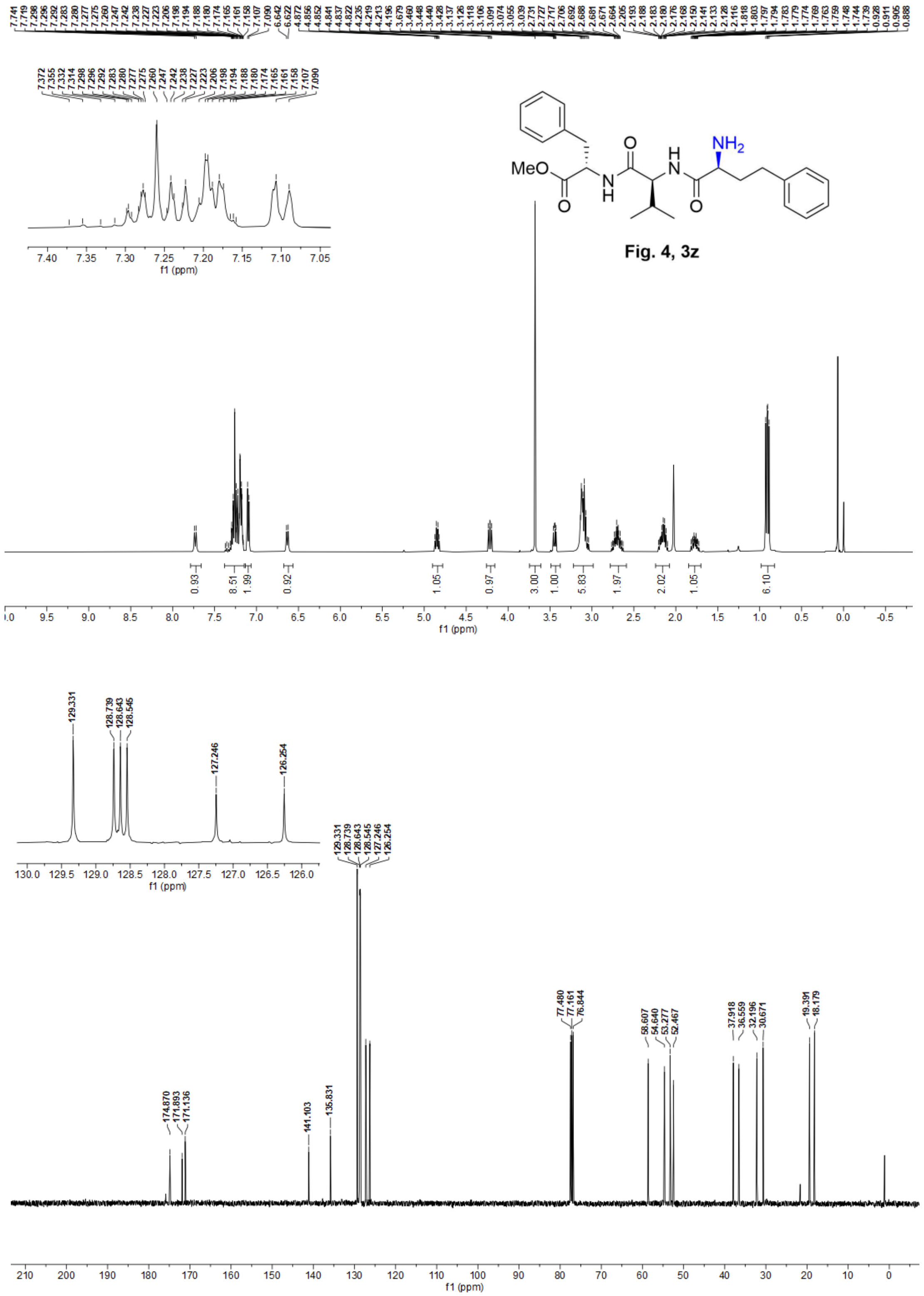

Supplementary Figure 57. ^{1}H NMR and ^{13}C NMR spectra of compound **3z**

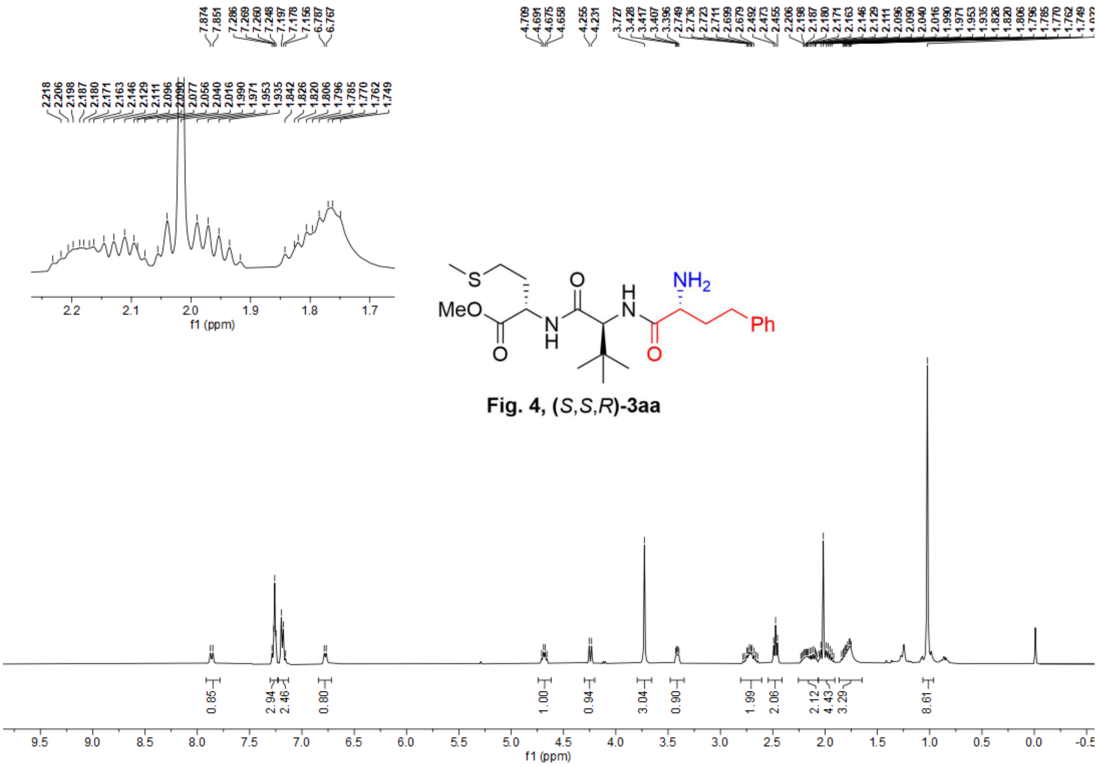

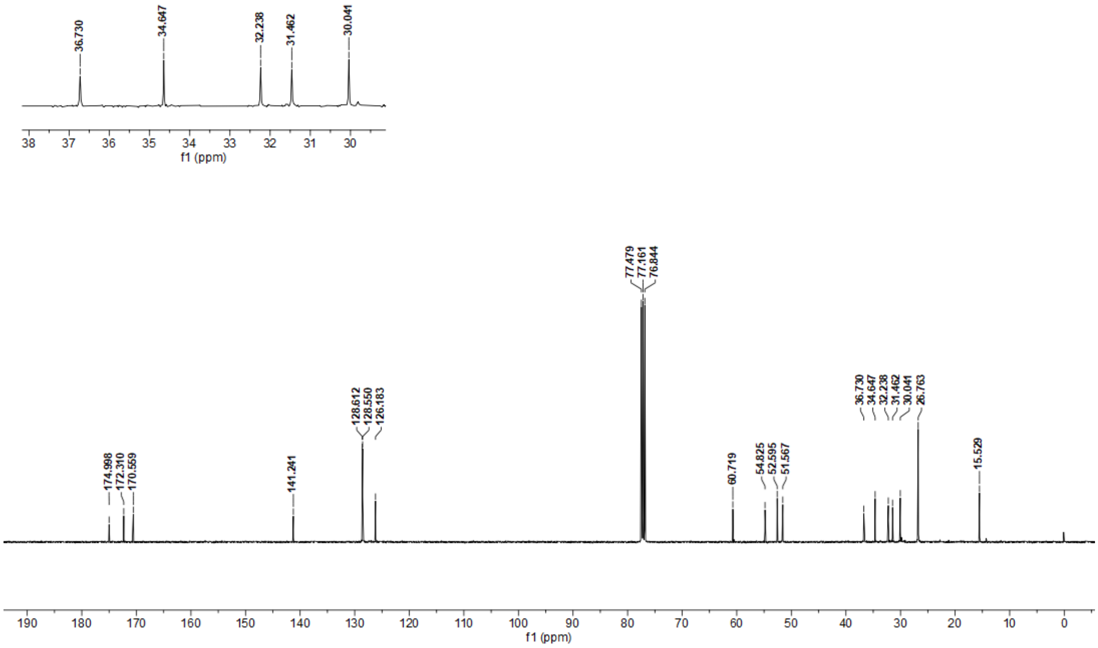

Supplementary Figure 58. ^{1}H NMR and ^{13}C NMR spectra of compound (*S*,*S*,*R*)**-3aa**

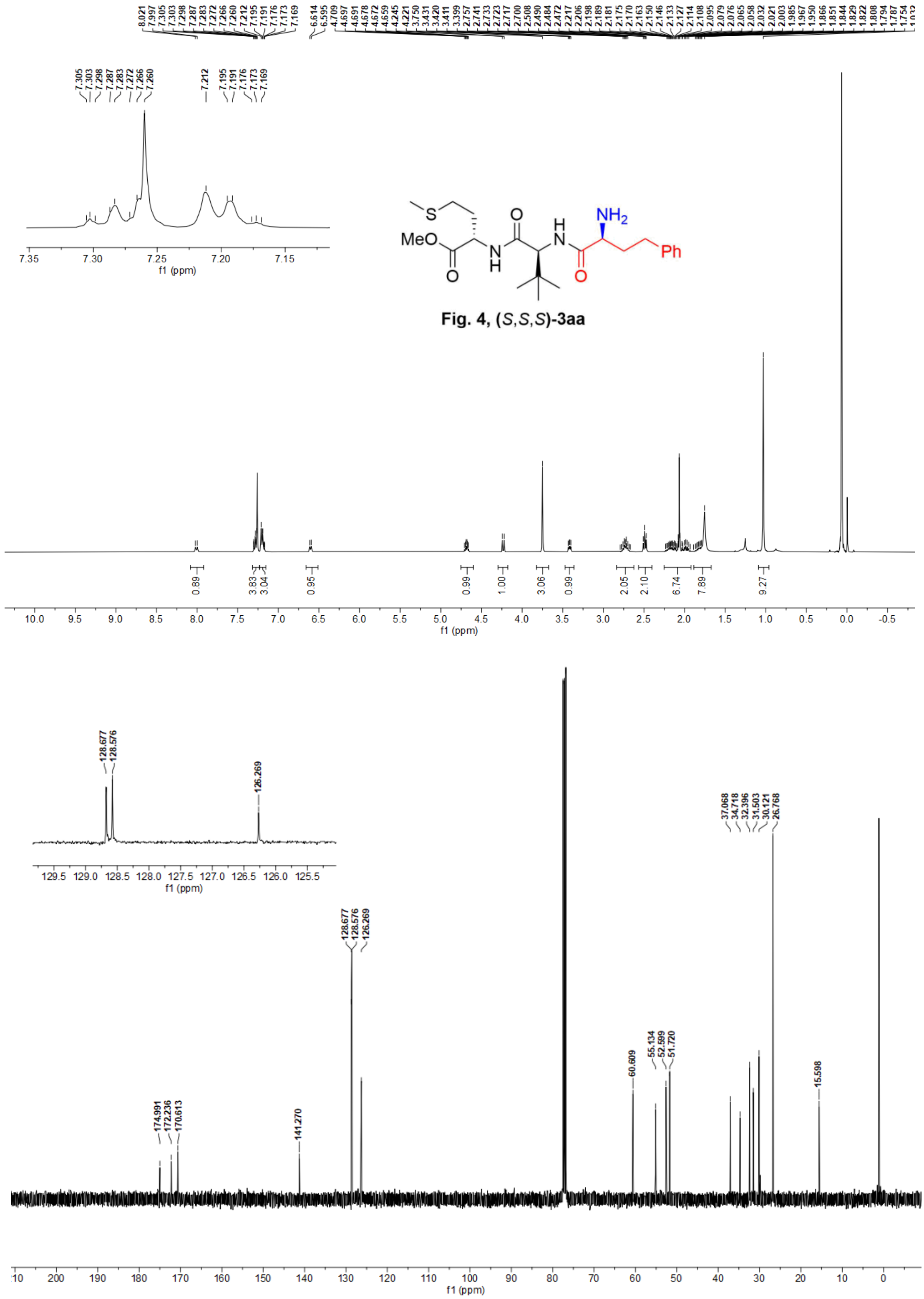

Supplementary Figure 59. ^{1}H NMR and ^{13}C NMR spectra of compound (*S*,*S*,*S*)**-3aa**

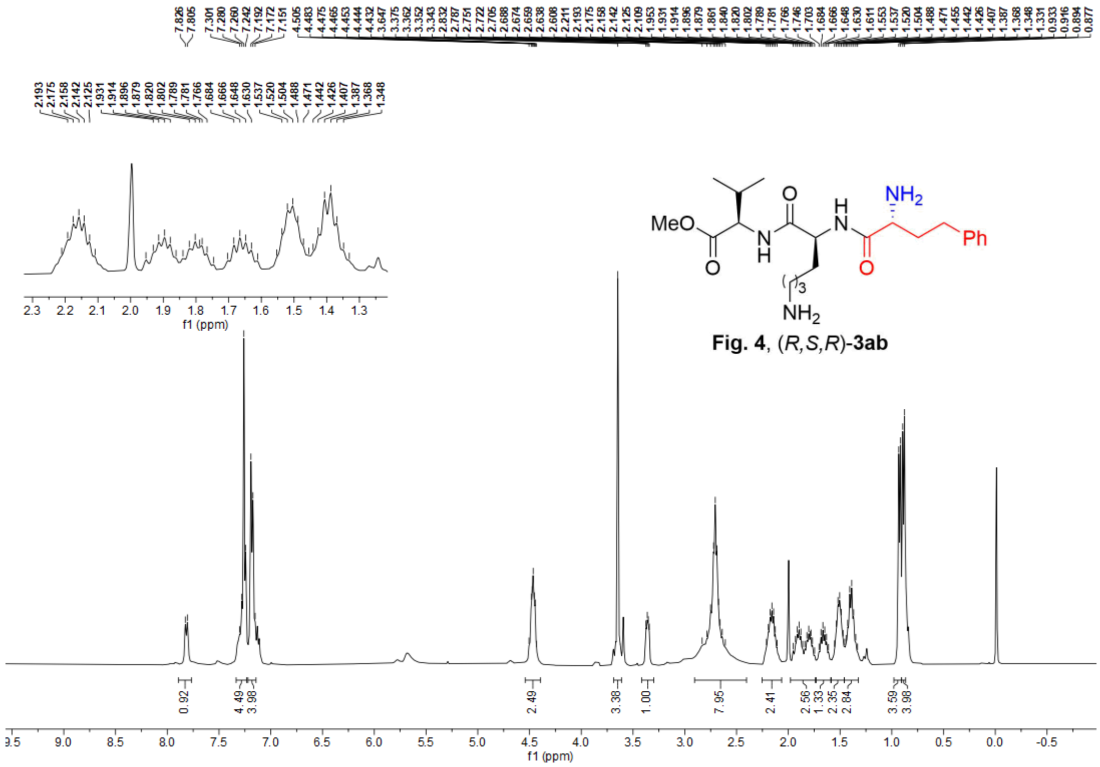

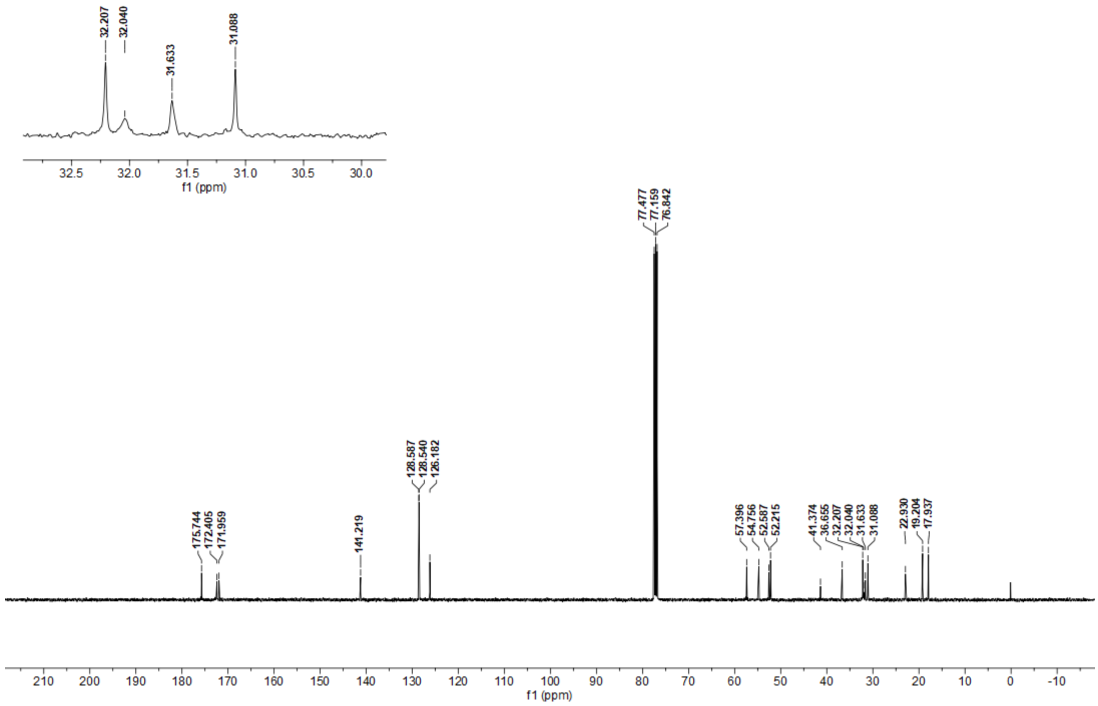

Supplementary Figure 60. ^{1}H NMR and ^{13}C NMR spectra of compound (*R*,*S*,*R*)**-3ab**

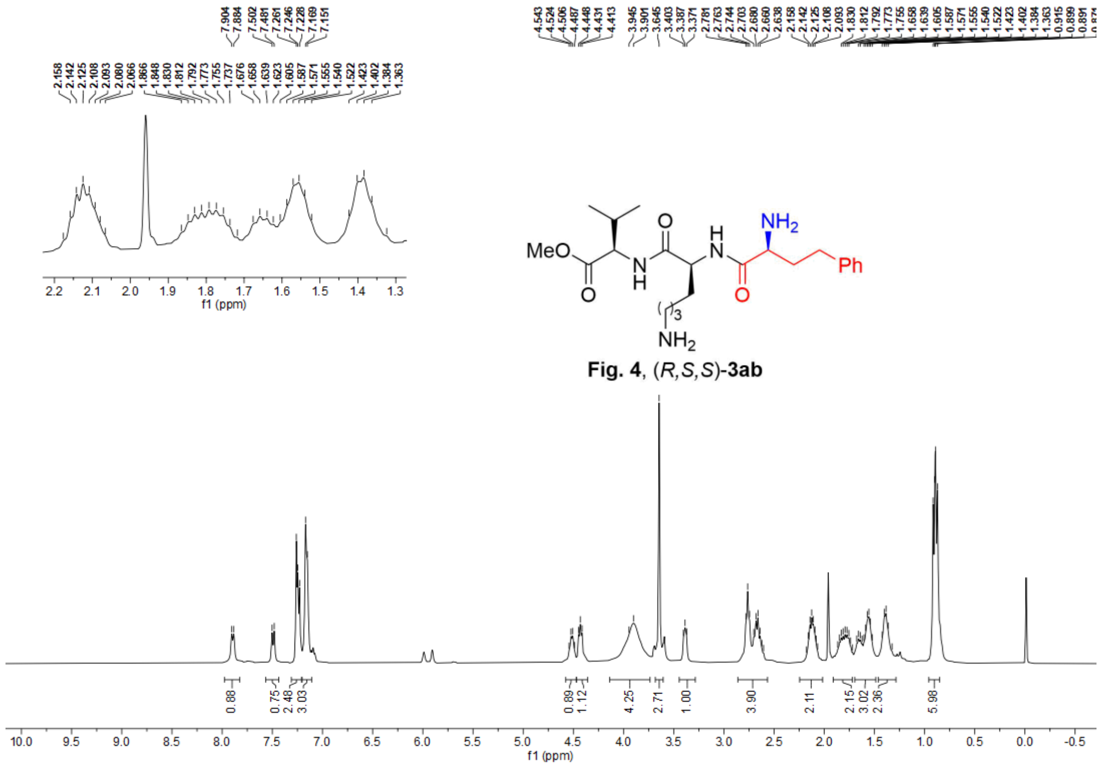

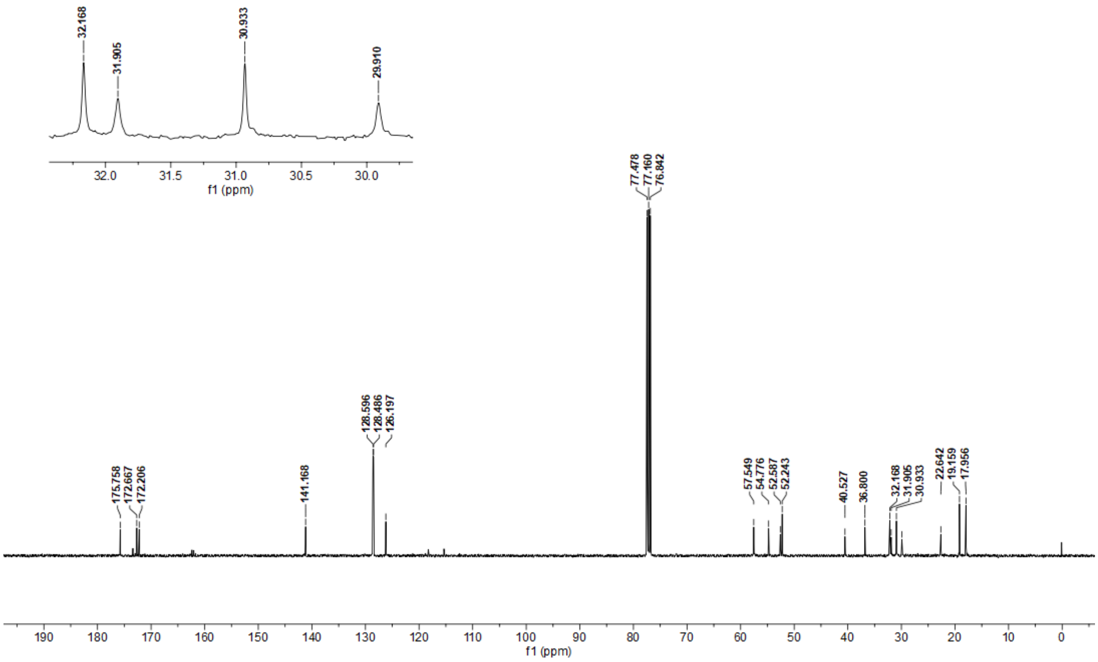

Supplementary Figure 61. ^{1}H NMR and ^{13}C NMR spectra of compound (*R*,*S*,*S*)-**3ab**

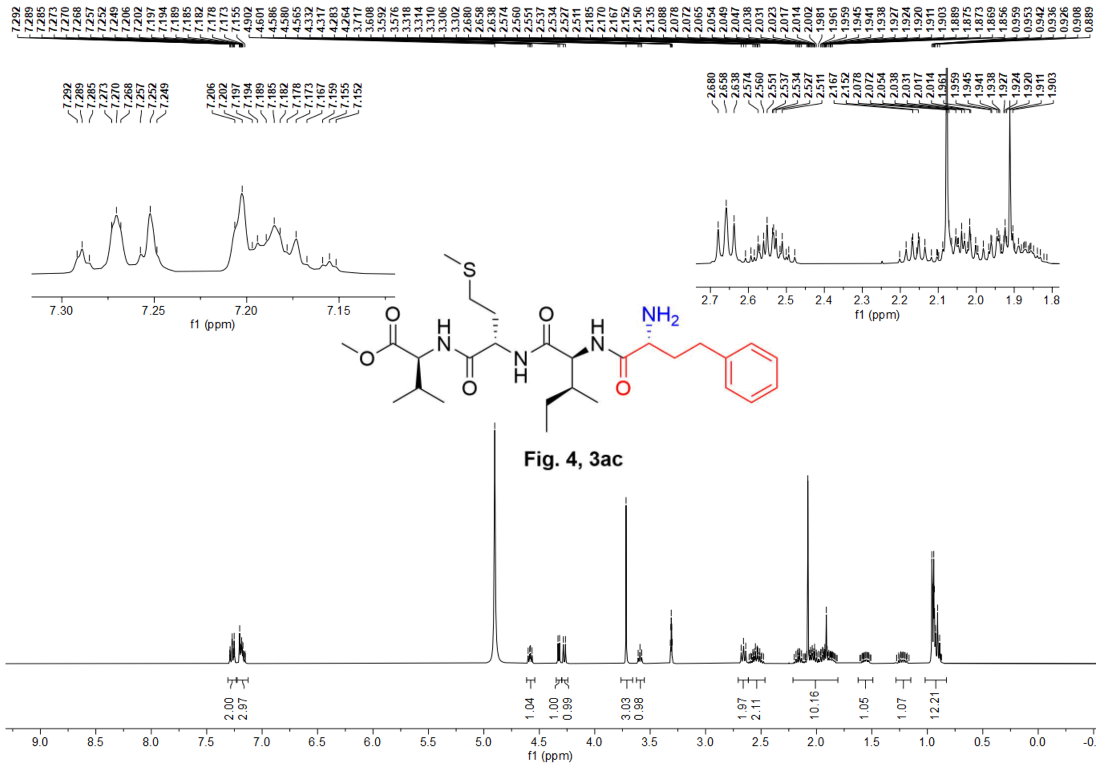

Fig. 4, 3ac

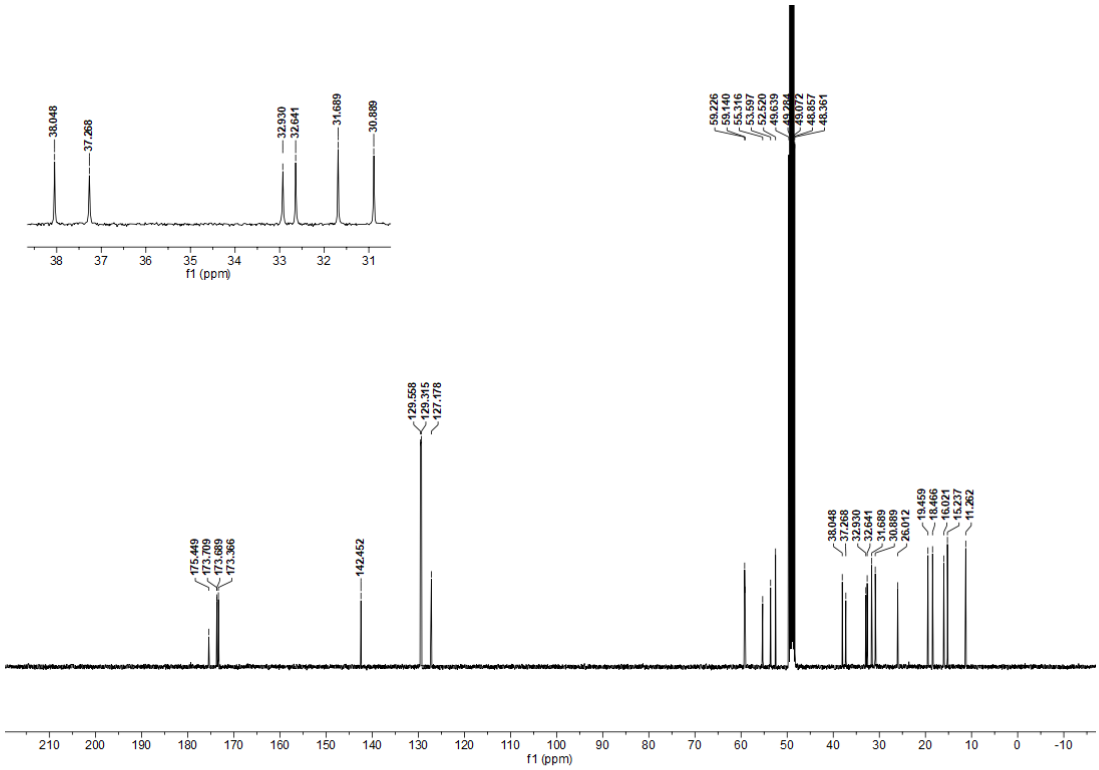

Supplementary Figure 62. ^{1}H NMR and ^{13}C NMR spectra of compound **3ac**

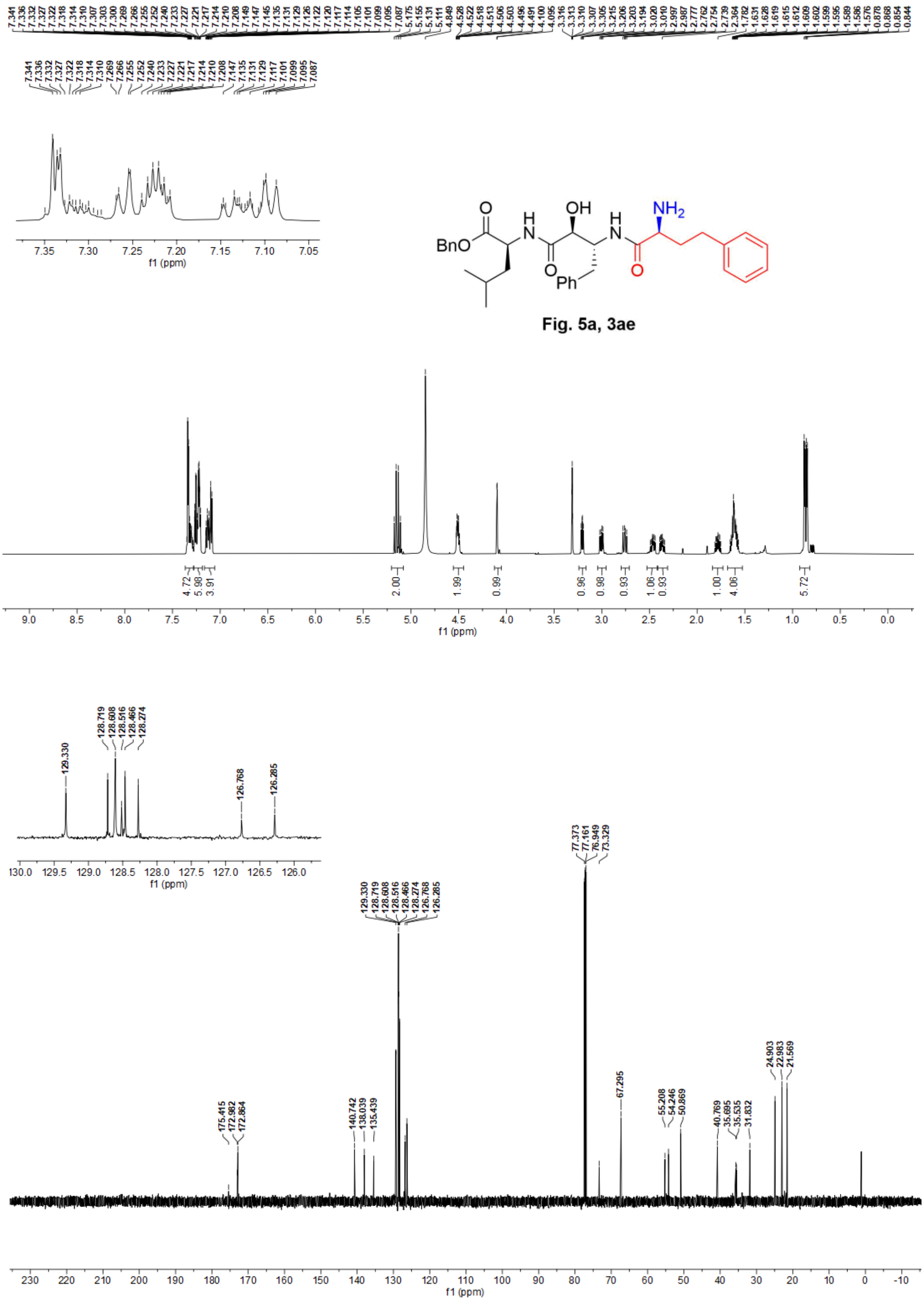

Supplementary Figure 63. ^{1}H NMR and ^{13}C NMR spectra of compound **3ae**

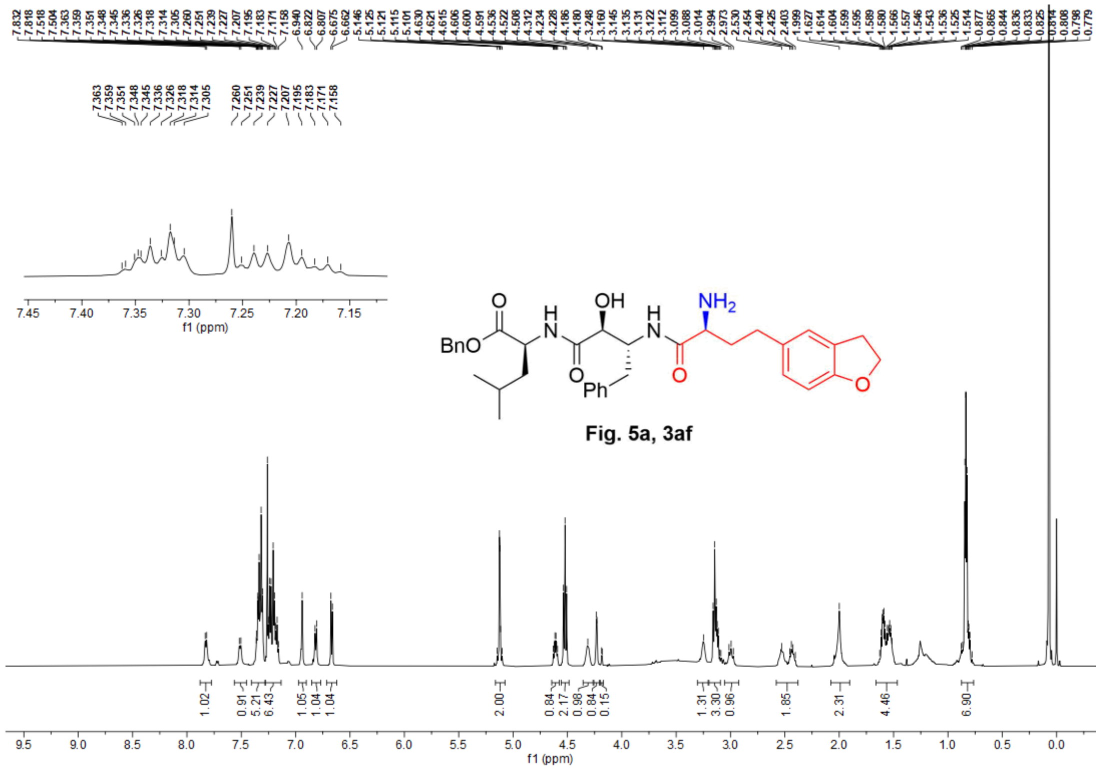

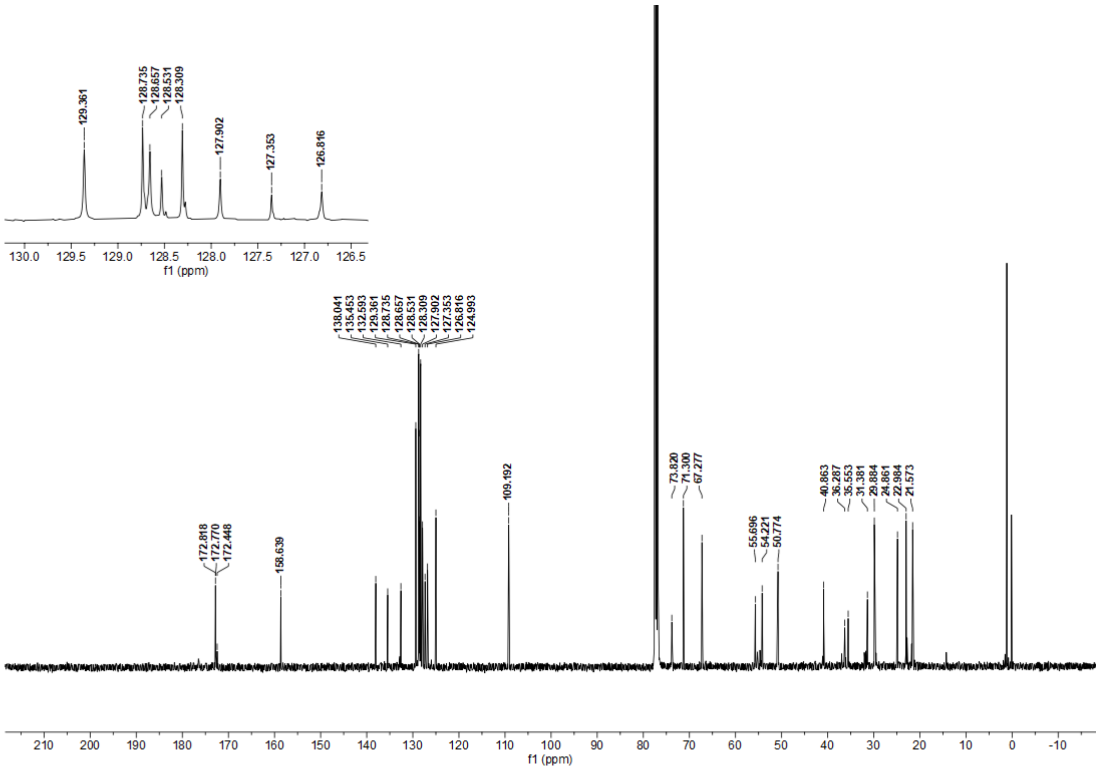

Supplementary Figure 64. ^{1}H NMR and ^{13}C NMR spectra of compound **3af**

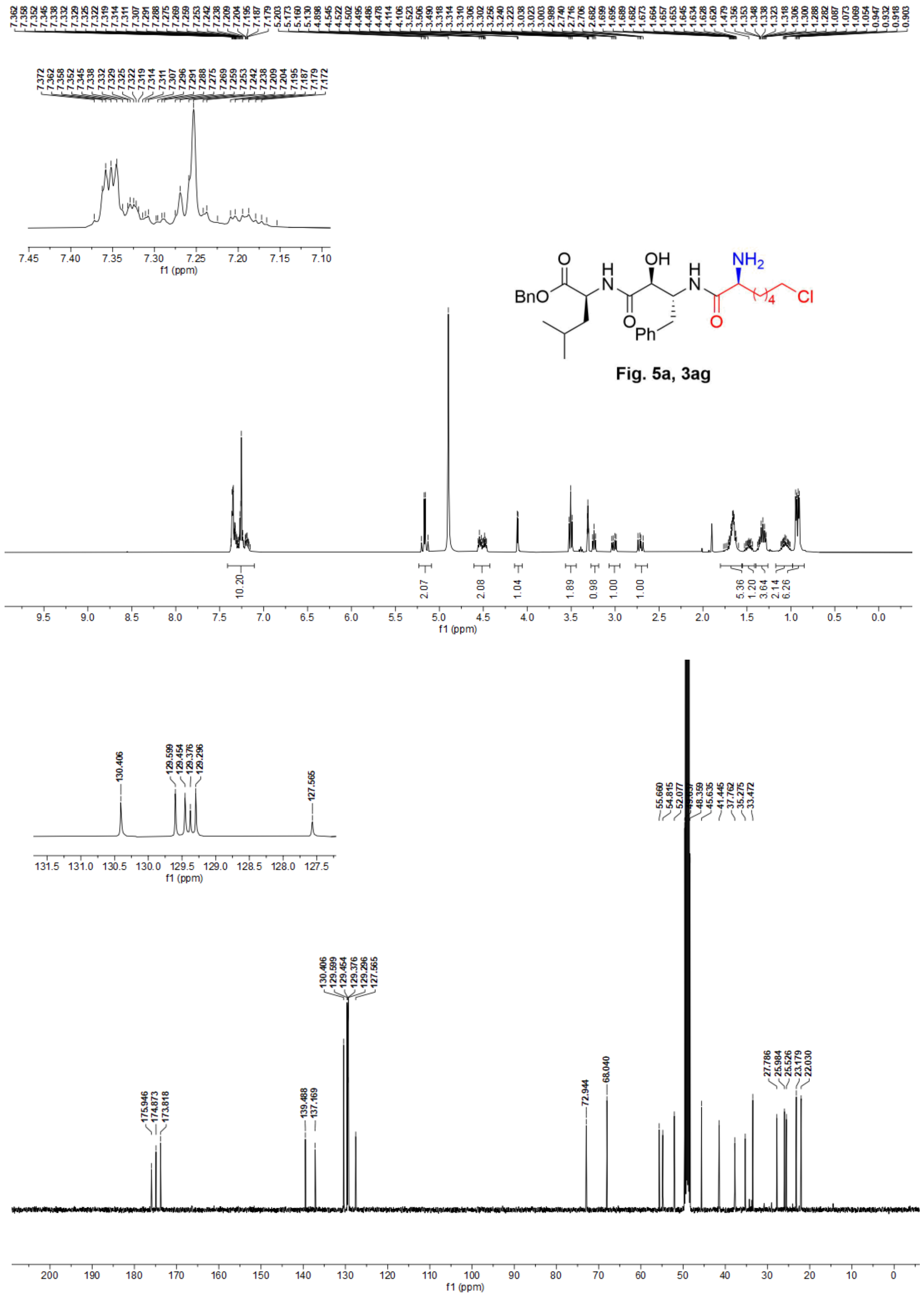

Supplementary Figure 65. ^{1}H NMR and ^{13}C NMR spectra of compound **3ag**

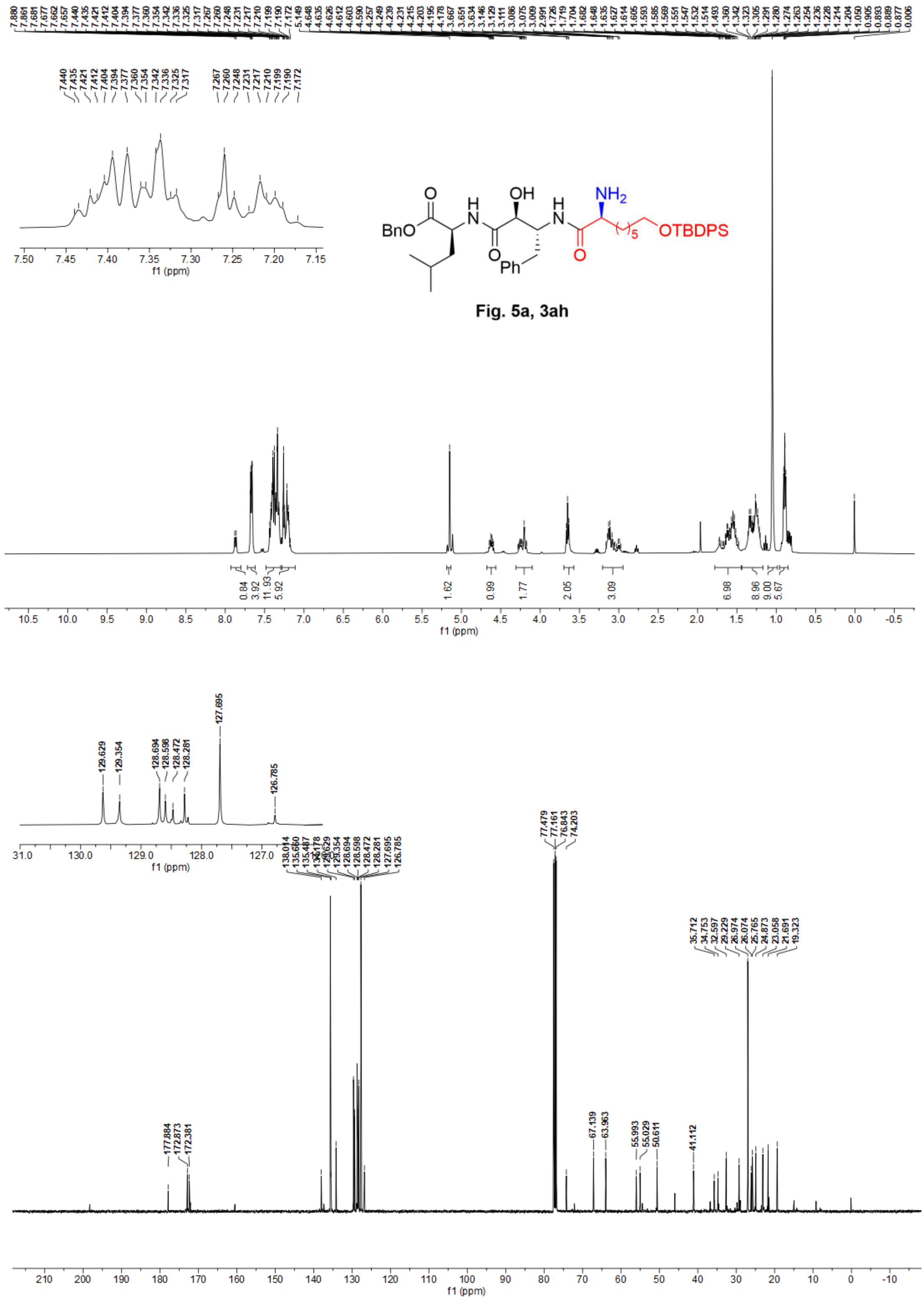

Supplementary Figure 66. ^{1}H NMR and ^{13}C NMR spectra of compound **3ah**

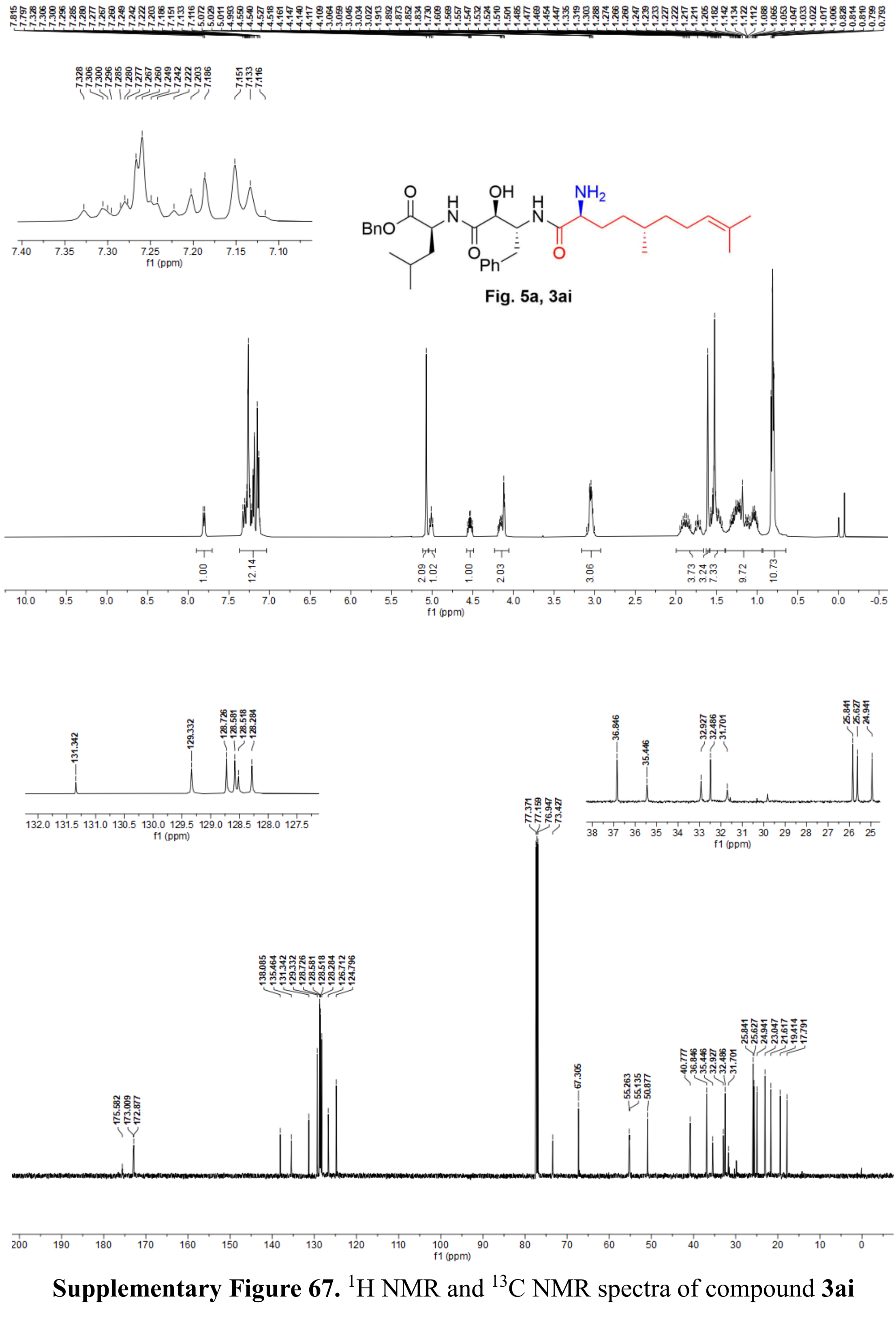

Supplementary Figure 67. ^{1}H NMR and ^{13}C NMR spectra of compound **3ai**

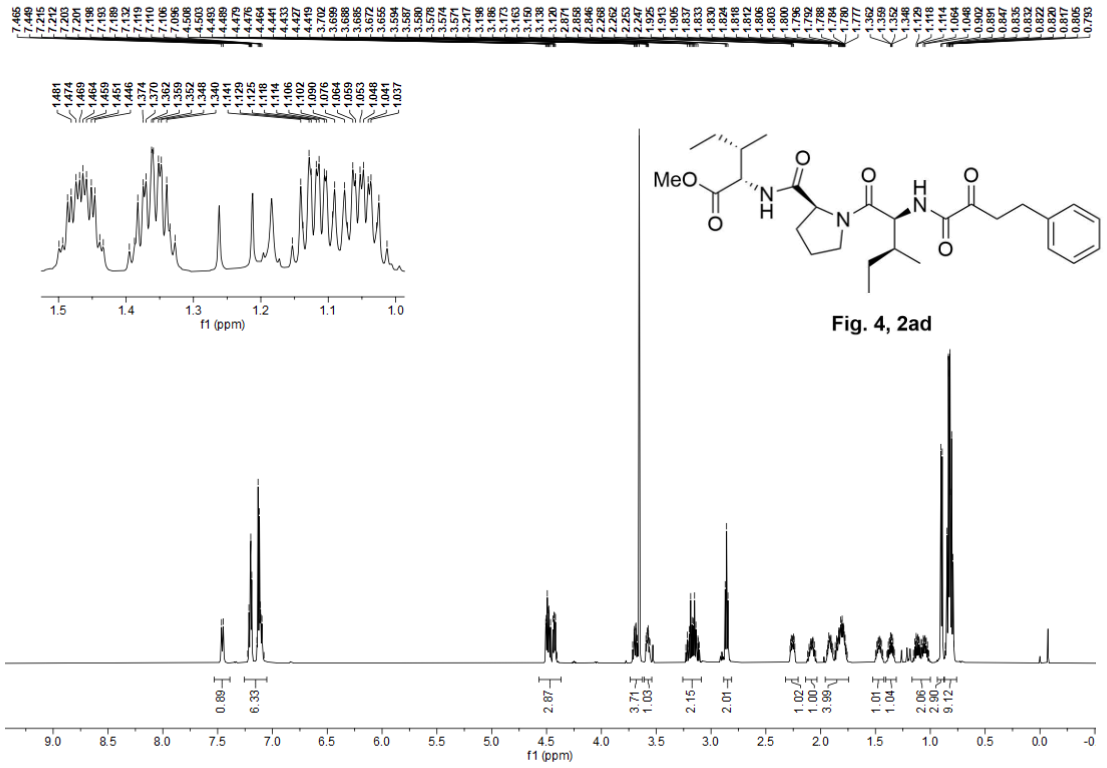

Fig. 4, 2ad

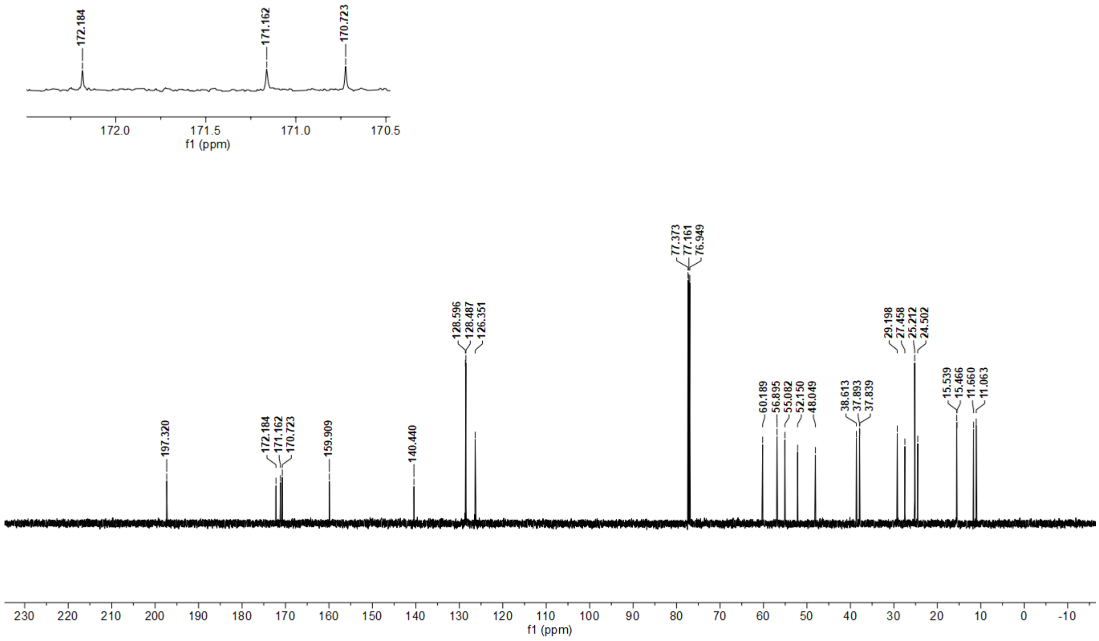

Supplementary Figure 68. ^{1}H NMR and ^{13}C NMR spectra of compound **2ad**

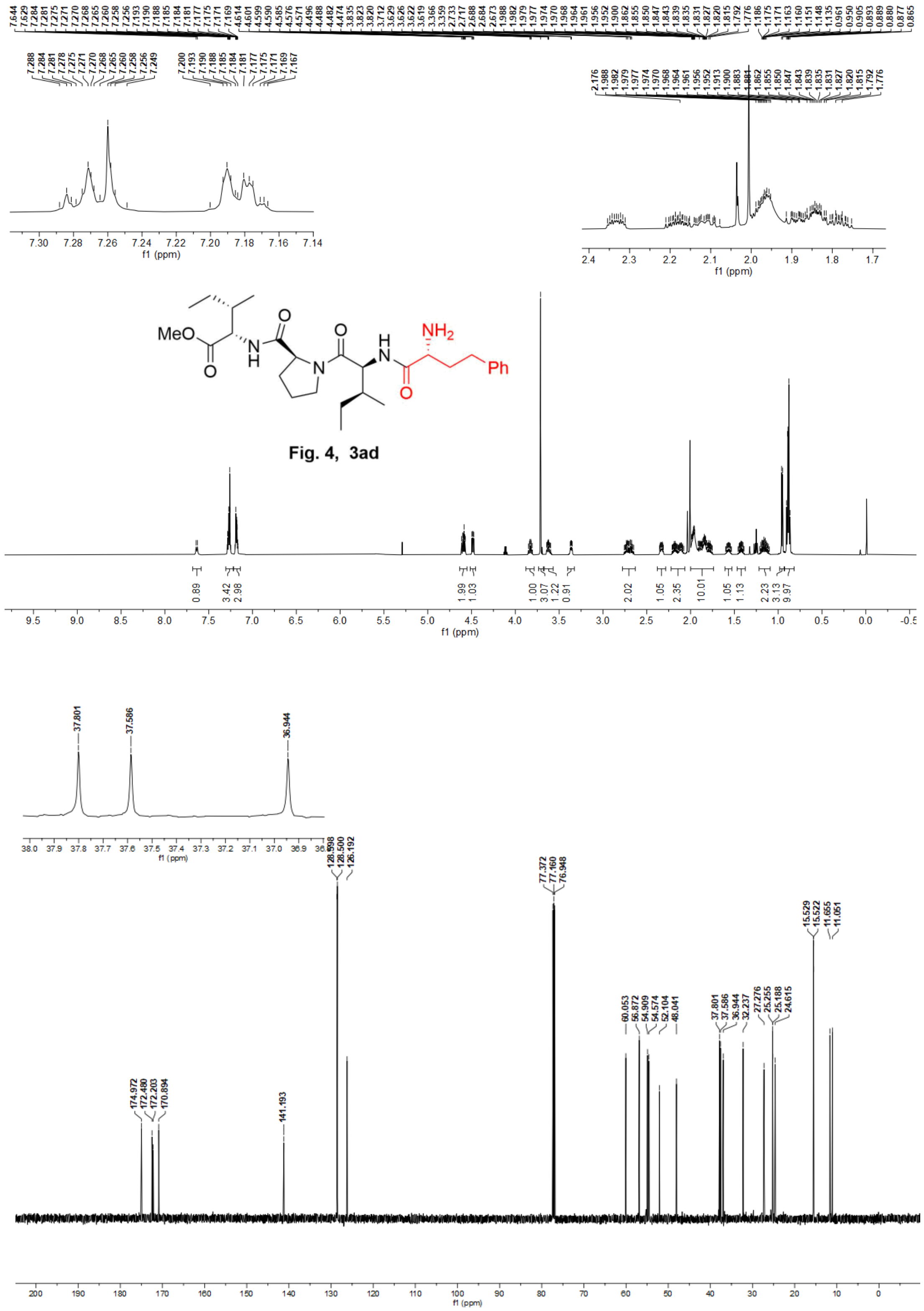

Supplementary Figure 69. ^{1}H NMR and ^{13}C NMR spectra of compound **3ad**

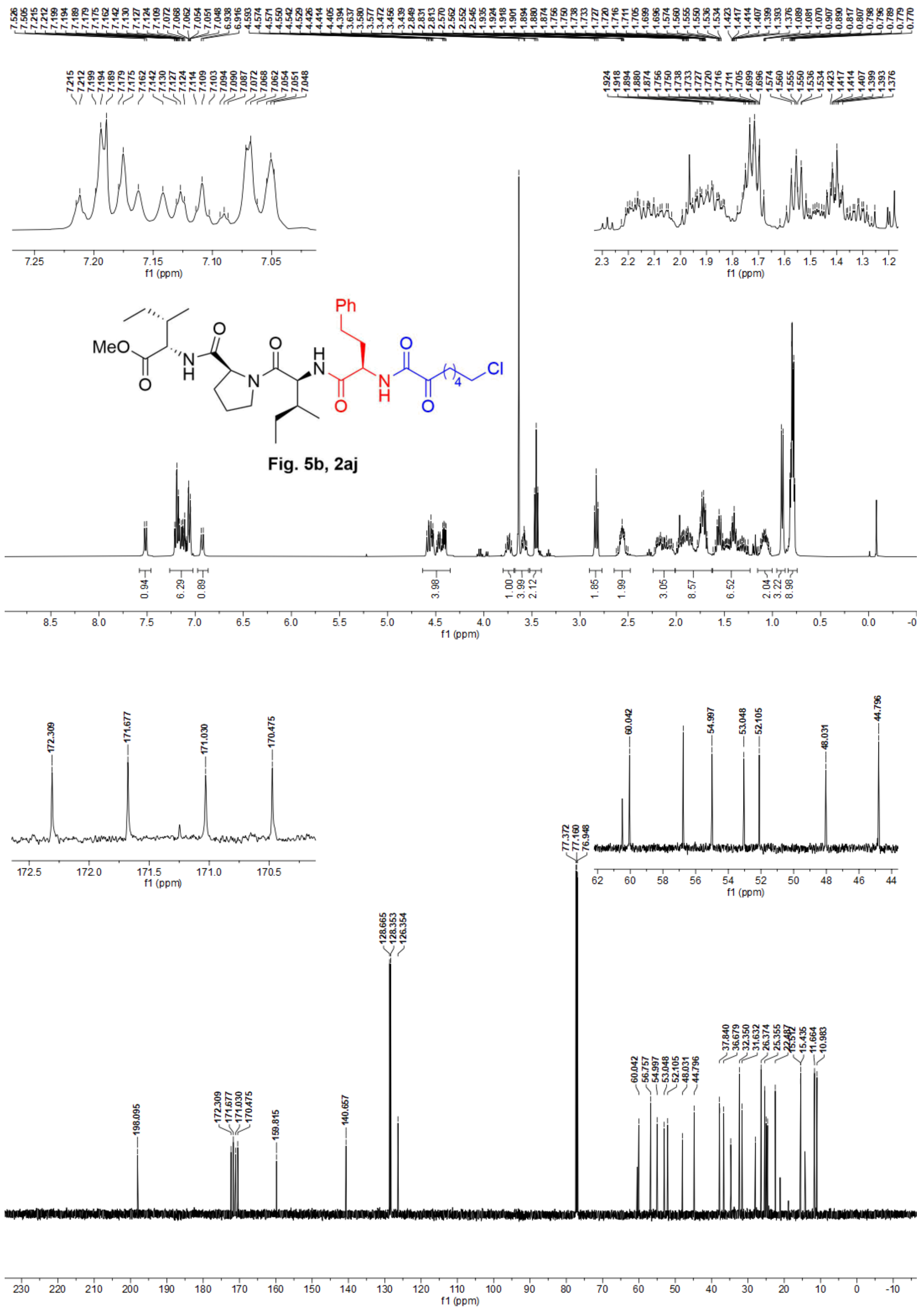

Supplementary Figure 70. ^{1}H NMR and ^{13}C NMR spectra of compound **2aj**

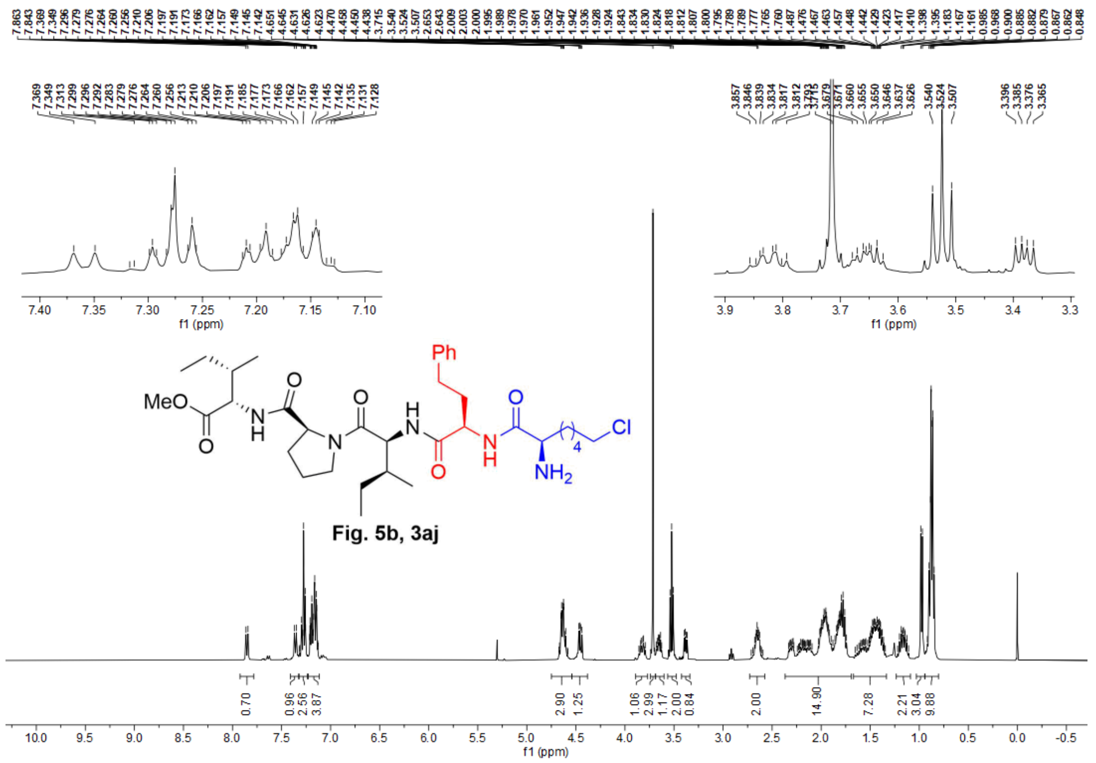

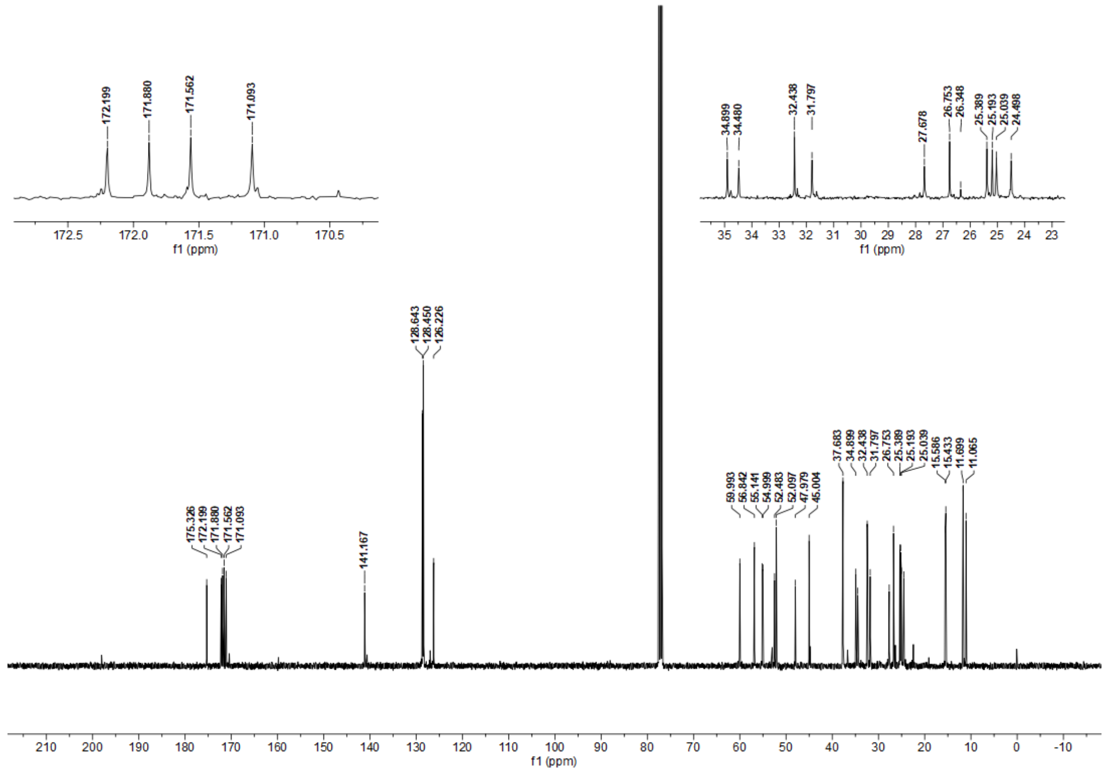

Supplementary Figure 71. ^{1}H NMR and ^{13}C NMR spectra of compound **3aj**

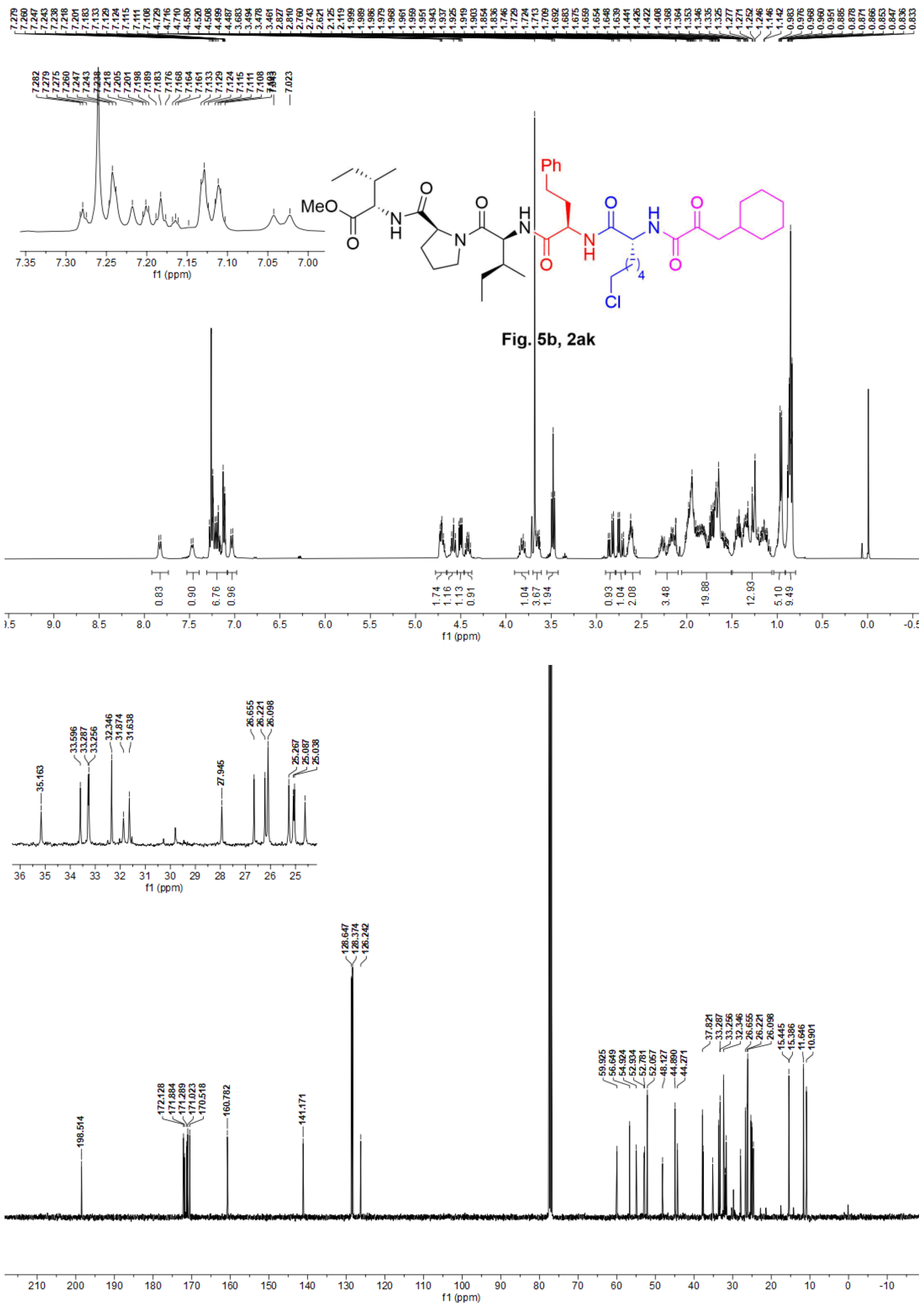

Supplementary Figure 72. ^{1}H NMR and ^{13}C NMR spectra of compound **2ak**

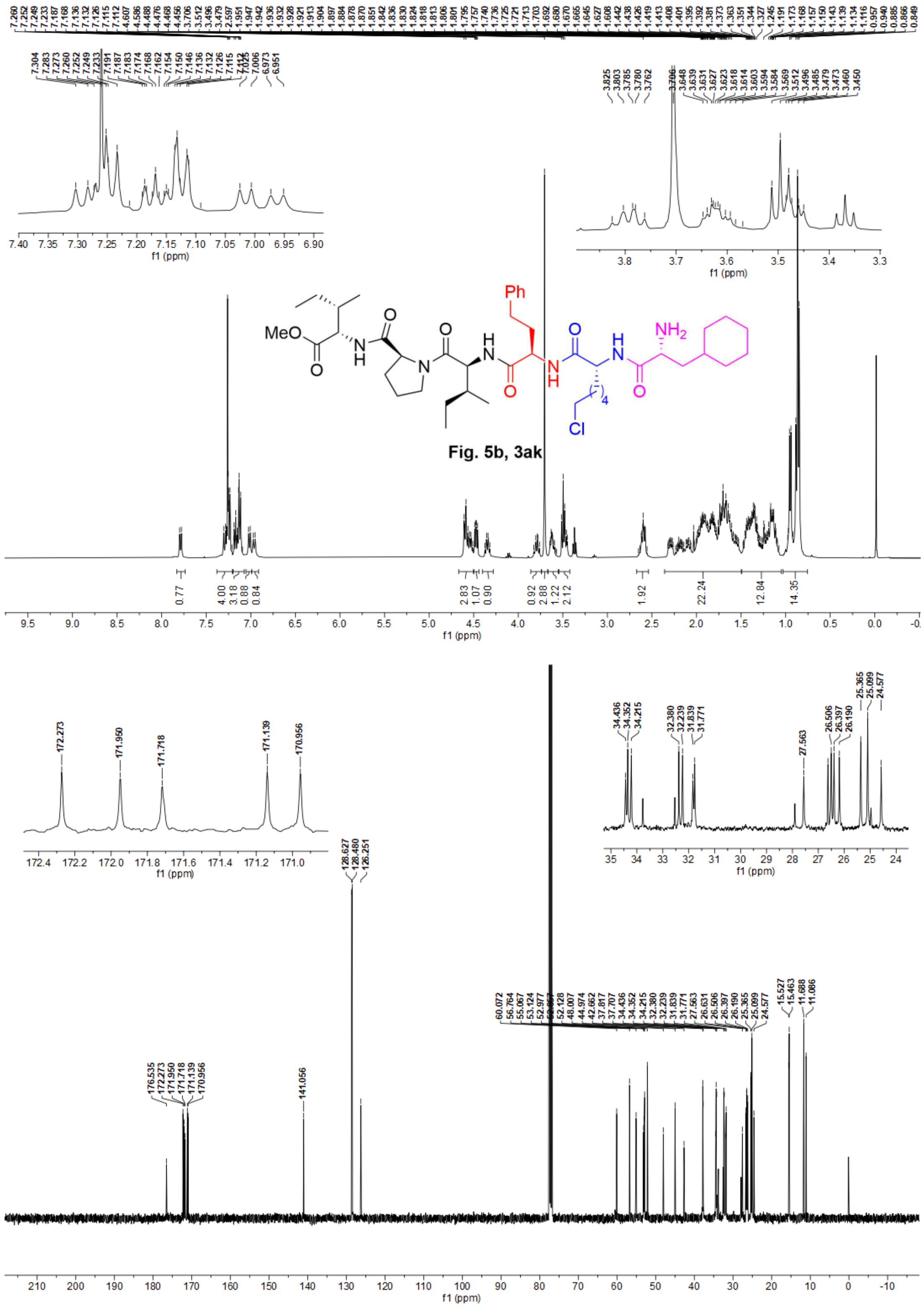

Supplementary Figure 73. ^{1}H NMR and ^{13}C NMR spectra of compound **3ak**

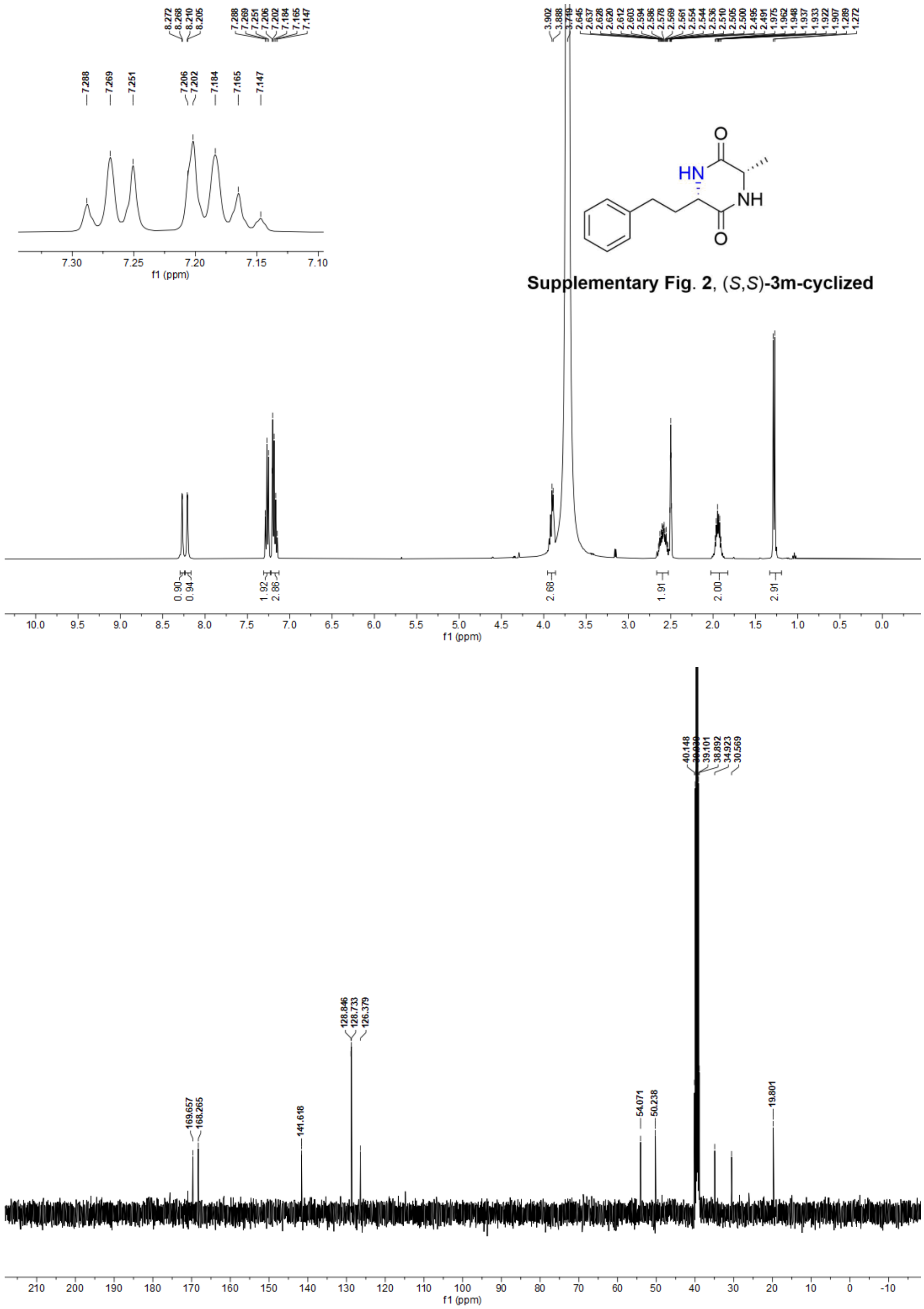

Supplementary Figure 2. ^{1}H NMR and ^{13}C NMR spectra of compound (*S*,*S*)**-3m-cyclized**

11.Chromatograms for Determination of ee and dr Values

Compound 3a (Fig. 4)

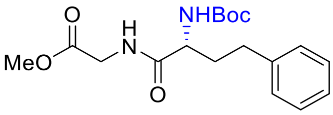

3a

HPLC Conditions

Column: Chiralcel AD-H, Daicel Chemical Industries, Ltd.

Eluent: Hexanes/Isopropanol (95:5)

Flow rate: 1.0 mL/min

Detection: UV 214 nm

Racemic

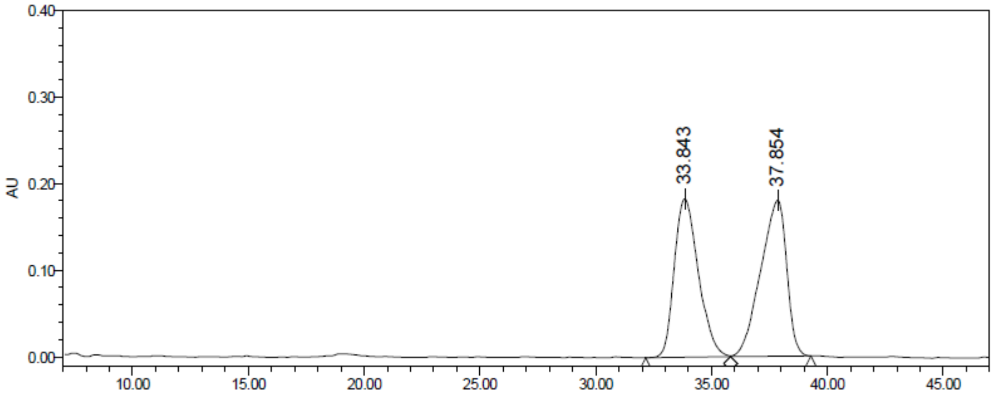

Detector A Ch1 214nm

	ret.time	area	area %
1	33.843	13246412	48.555
2	37.854	14034633	51.445
Sum		27281045	100.000

Chiral

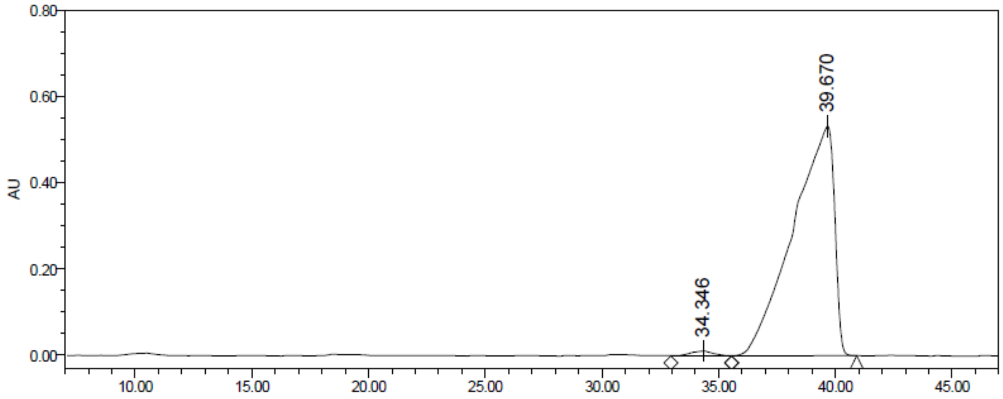

Detector A Ch1 214nm

	ret.time	area	area %
1	34.346	754058	1.132
2	39.670	65878319	98.868
Sum		66632377	100.000

Supplementary Figure 74. HPLC data of compound **3a**

Compound 3b (Fig. 4)

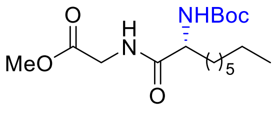

3b

HPLC Conditions

Column: Chiralcel AD-H, Daicel Chemical Industries, Ltd.

Eluent: Hexanes/Isopropanol (90:10)

Flow rate: 1.0 mL/min

Detection: UV 214 nm

Racemic

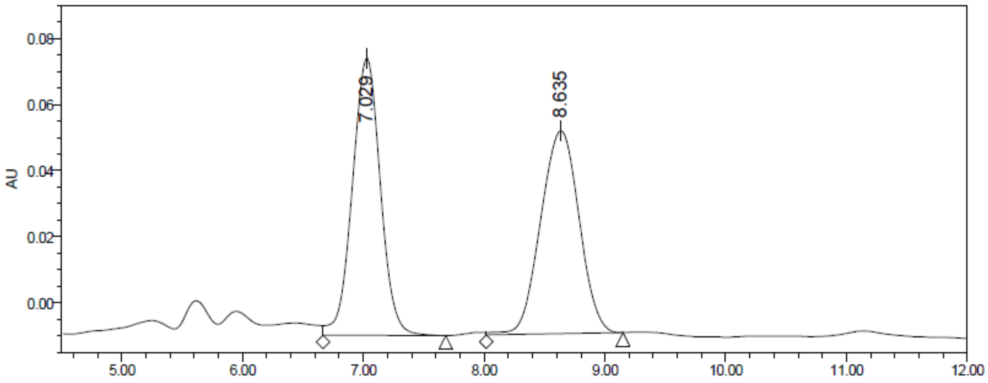

Detector A Ch1 214nm

	ret.time	area	area %
1	7.029	1356693	49.991
2	8.635	1357178	50.009
Sum		2713871	100.000

Chiral

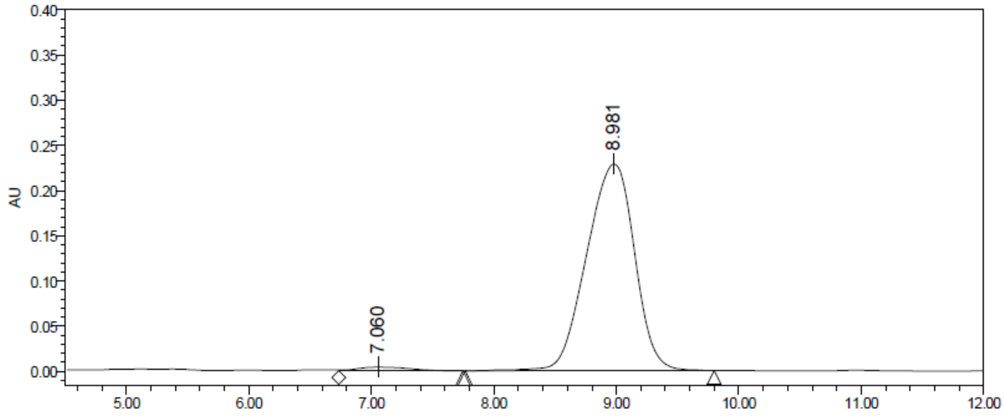

Detector A Ch1 214nm

	ret.time	area	area %
1	7.060	113206	1.781
2	8.981	6243269	98.219
Sum		6356475	100.000

Supplementary Figure 75. HPLC data of compound **3b**

Compound 3c (Fig. 4)

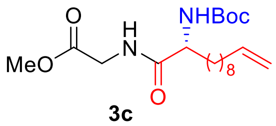

3c

HPLC Conditions

Column: Chiralcel AD-H, Daicel Chemical Industries, Ltd.

Eluent: Hexanes/Isopropanol (94:6)

Flow rate: 0.9 mL/min

Detection: UV 214 nm

Racemic

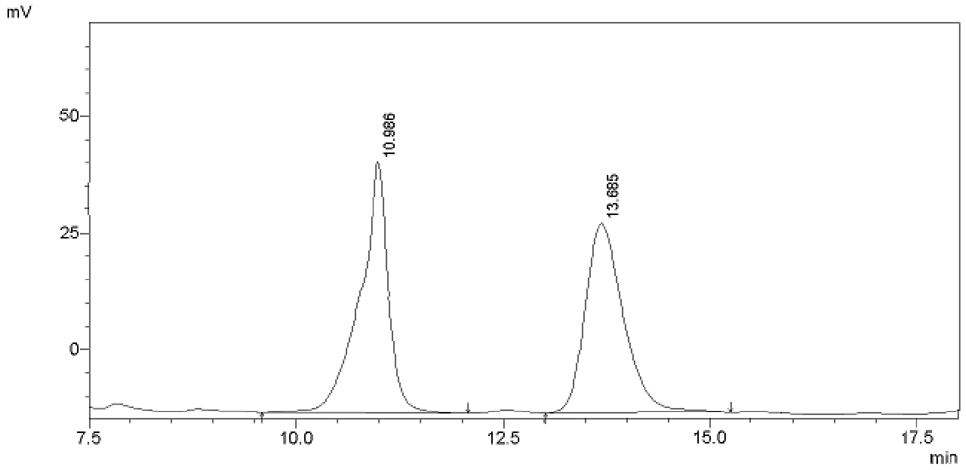

Detector A Ch1 214nm

Peak	Time	Area	Area %
1	10. 986	1282385	49. 816
2	13. 685	1291844	50. 184
		2574229	100. 000

Chiral

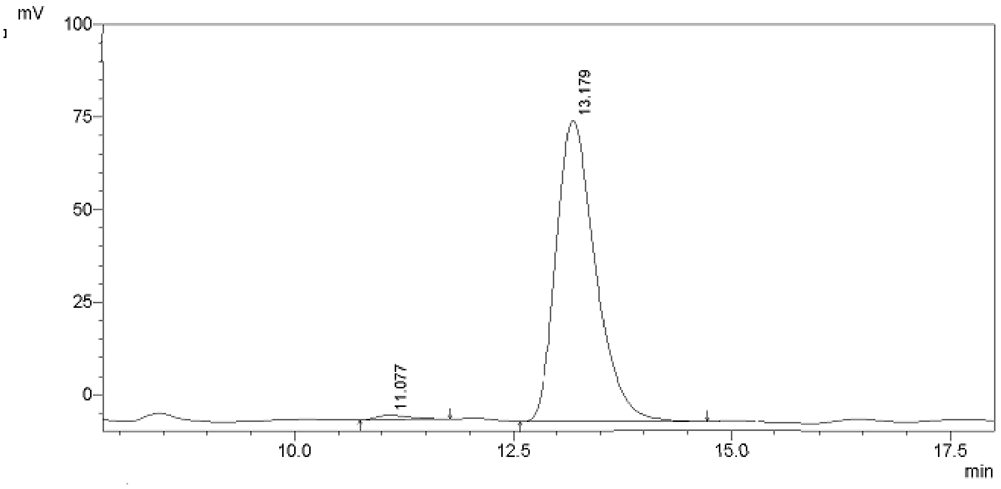

Detector A Ch1 214nm

Peak	Time	Area	Area %
1	11. 077	33297	1. 291
2	13. 179	2545171	98. 709
		2578469	100. 000

Supplementary Figure 76. HPLC data of compound **3c**

Compound 3d (Fig. 4)

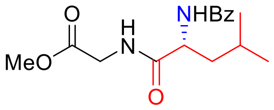

HPLC Conditions

Column: Chiralcel AD-H, Daicel Chemical Industries, Ltd.
Eluent: Hexanes/Isopropanol (90:10)
Flow rate: 1.0 mL/min
Detection: UV 230 nm

Racemic

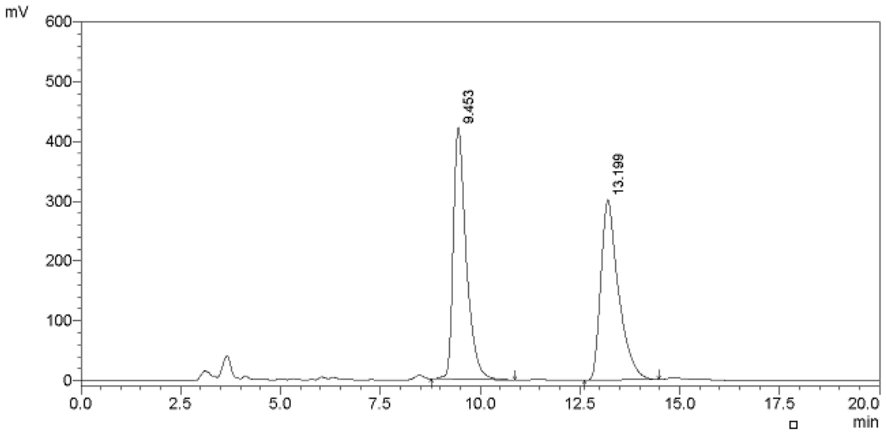

Detector A Ch1 230nm

Peak	Time	Area	Area %
1	9.453	9494256	50.508
2	13.199	9303193	49.492
		18797449	100.000

Chiral

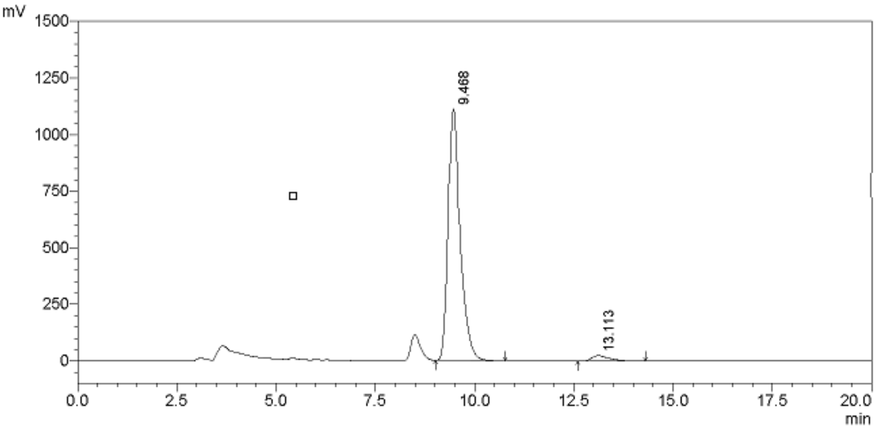

Detector A Ch1 230nm

Peak	Time	Area	Area %
1	9.468	23504568	96.829
2	13.113	769677	3.171
		24274246	100.000

Supplementary Figure 77. HPLC data of compound **3d**

Compound 3e (Fig. 4)

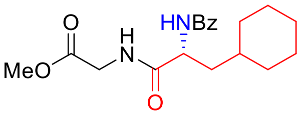

HPLC Conditions

Column: Chiralcel AD-H, Daicel Chemical Industries, Ltd.

Eluent: Hexanes/Isopropanol (90:10)

Flow rate: 1.0 mL/min

Detection: UV 214 nm

Racemic

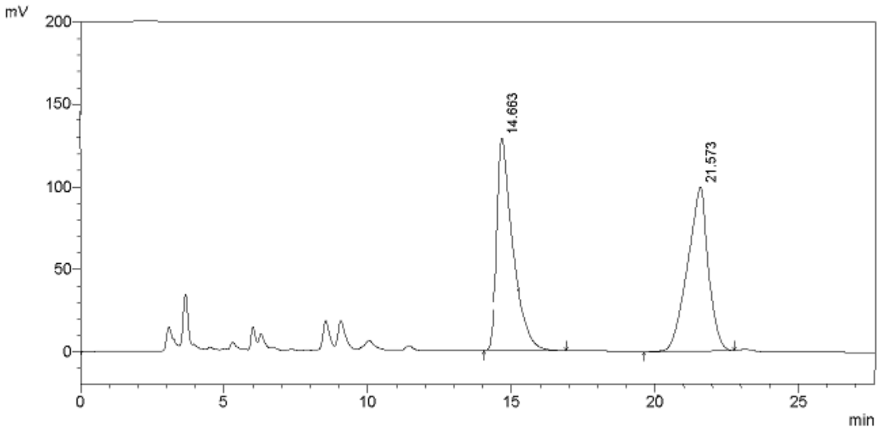

Detector A Ch1 214nm

Peak	Time	Area	Area %
1	14.663	5029166	50.265
2	21.573	4976143	49.735
		10005309	100.000

Chiral

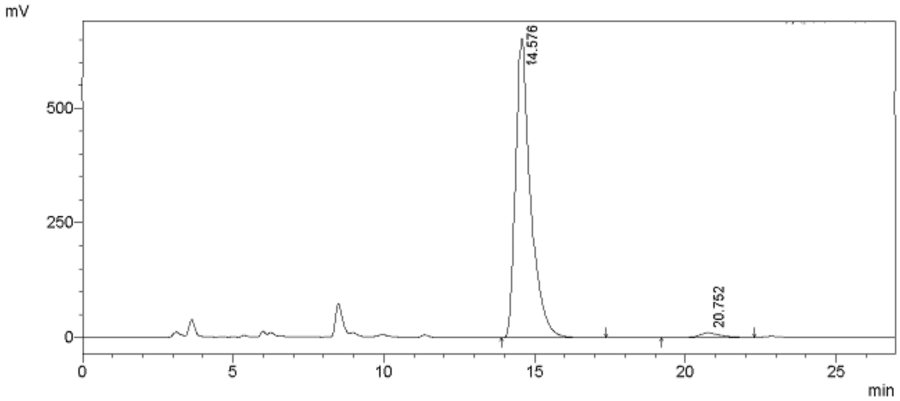

Detector A Ch1 214nm

Peak	Time	Area	Area %
1	14.576	22627602	97.889
2	20.752	487885	2.111
		23115487	100.000

Supplementary Figure 78. HPLC data of compound **3e**

Compound 3f (Fig. 4)

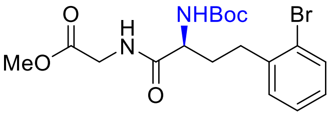

3f

HPLC Conditions

Column: Chiralcel OD-H, Daicel Chemical Industries, Ltd.
Eluent: Hexanes/Isopropanol (90:10)
Flow rate: 1.0 mL/min
Detection: UV 214 nm

Racemic

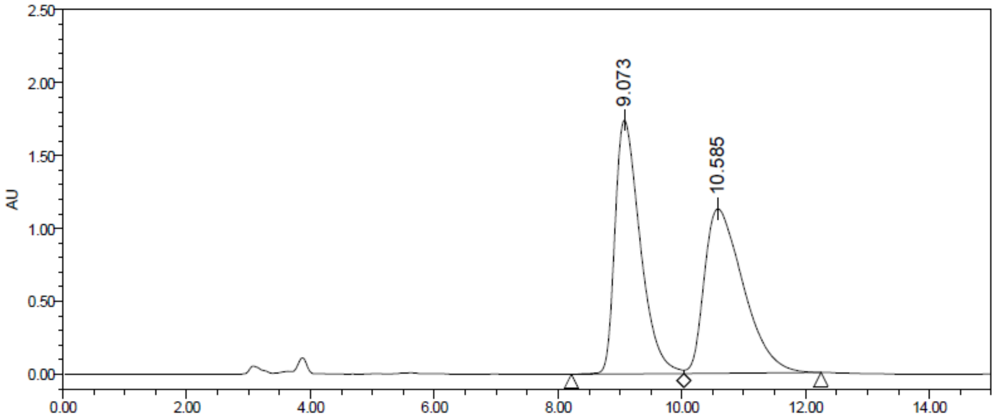

Detector A Ch1 214nm

	ret.time	area	area %
1	9.073	49419542	50.018
2	10.585	49384035	49.982
Sum		98803577	100.000

Chiral

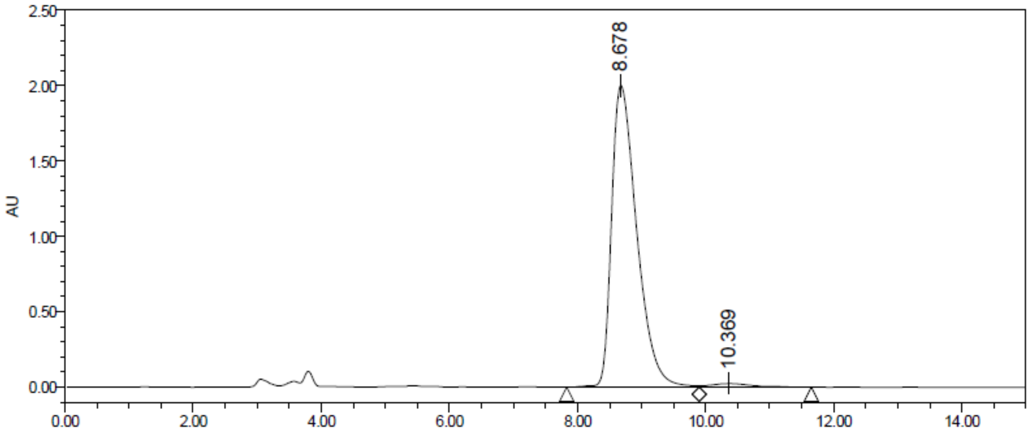

Detector A Ch1 214nm

	ret.time	area	area %
1	8.678	54607739	98.539
2	10.369	809430	1.461
Sum		55417170	100.000

Supplementary Figure 79. HPLC data of compound **3f**

Compound 3g (Fig. 4)

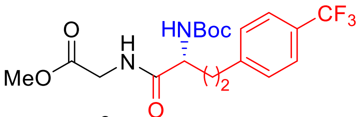

3g

HPLC Conditions

Column: Chiralcel AD-H, Daicel Chemical Industries, Ltd.

Eluent: Hexanes/Isopropanol (90:10)

Flow rate: 1.0 mL/min

Detection: UV 230 nm

Racemic

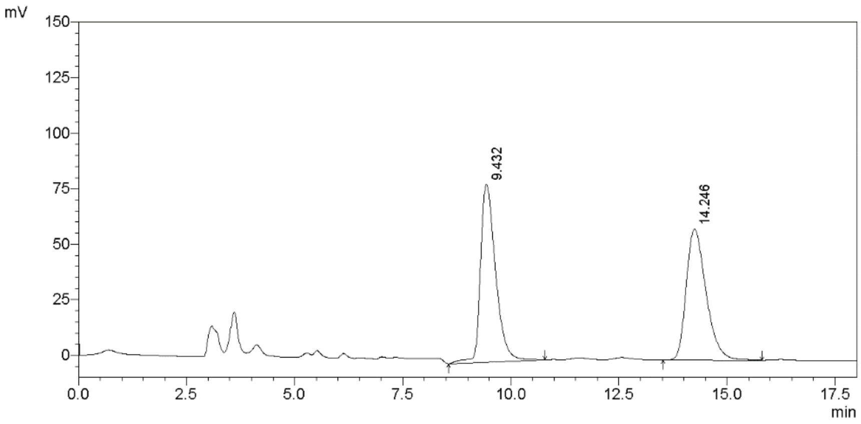

Detector A Ch1 230nm

Peak	Time	Area	Area %
1	9.432	1948084	50.838
2	14.246	1883859	49.162
		3831943	100.000

Chiral

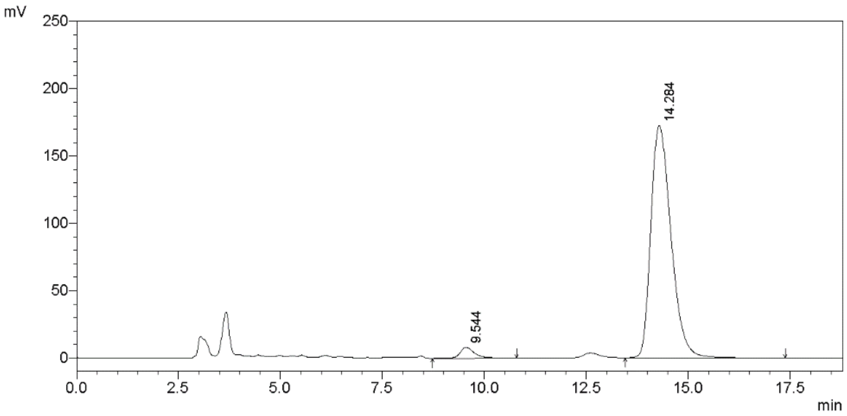

Detector A Ch1 230nm

Peak	Time	Area	Area %
1	9.544	196888	3.212
2	14.284	5932332	96.788
		6129220	100.000

Supplementary Figure 80. HPLC data of compound **3g**

Compound 3h (Fig. 4)

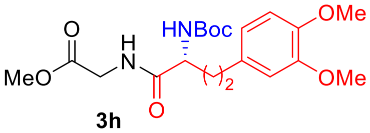

3h

HPLC Conditions

Column: Chiralcel OJ-H, Daicel Chemical Industries, Ltd.

Eluent: Hexanes/Isopropanol (90:10)

Flow rate: 1.0 mL/min

Detection: UV 230 nm

Racemic

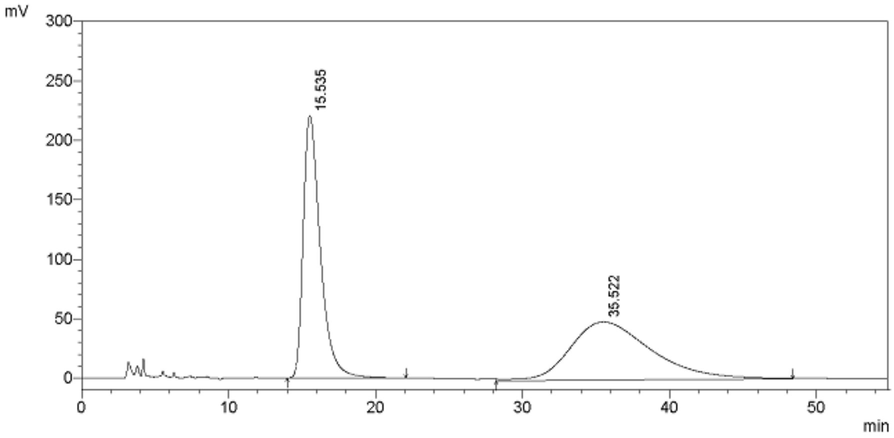

Detector A Ch1 230nm

Peak	Time	Area	Area %
1	15.535	17809909	48.716
2	35.522	18748365	51.284
		36558273	100.000

Chiral

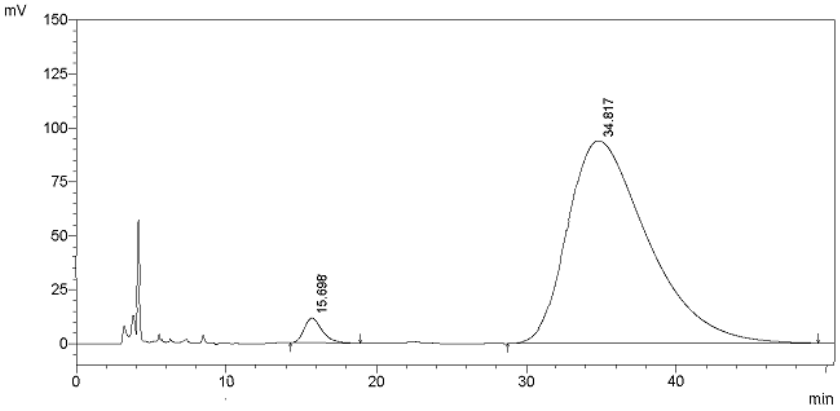

Detector A Ch1 230nm

Peak	Time	Area	Area %
1	15.698	942625	2.642
2	34.817	34736581	97.358
		35679205	100.000

Supplementary Figure 81. HPLC data of compound **3h**

Compound 3i (Fig. 4)

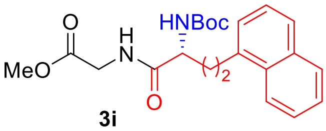

3i

HPLC Conditions

Column: Chiralcel OD-H, Daicel Chemical Industries, Ltd.

Eluent: Hexanes/Isopropanol (85:15)

Flow rate: 1.0 mL/min

Detection: UV 214 nm

Racemic

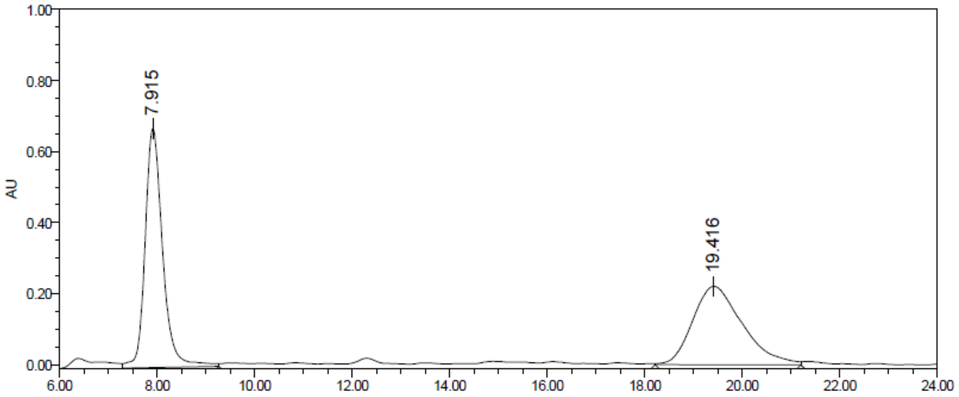

Detector A Ch1 214nm

	ret time	area	area %
1	7.915	16850498	51.838
2	19.416	15655582	48.162
Sum		32506080	100.000

Chiral

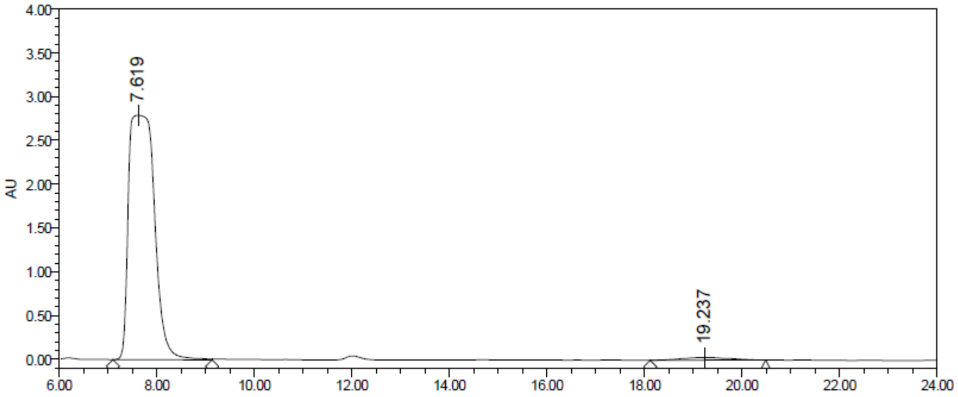

Detector A Ch1 214nm

	ret time	area	area %
1	7.619	103408974	98.083
2	19.237	2020602	1.917
Sum		105429576	100.000

Supplementary Figure 82. HPLC data of compound **3i**

Compound 3j (Fig. 4)

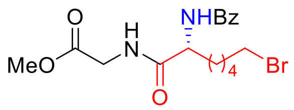

HPLC Conditions

Column: Chiralcel AD-H, Daicel Chemical Industries, Ltd.

Eluent: Hexanes/Isopropanol (90:10)

Flow rate: 1.0 mL/min

Detection: UV 230 nm

Racemic

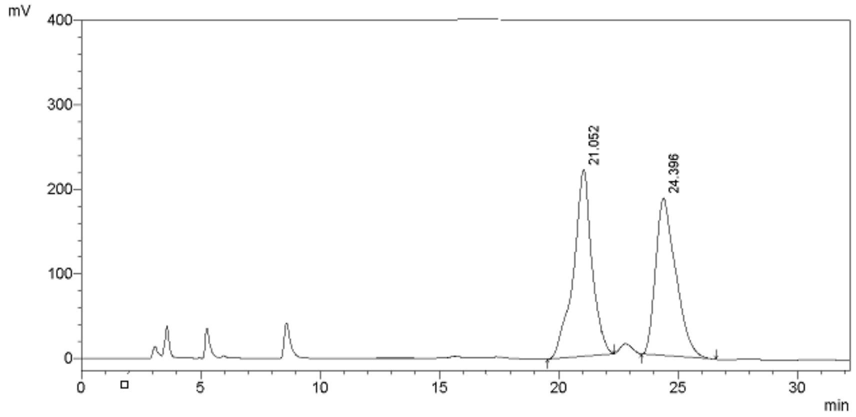

Detector A Ch1 230nm

Peak	Time	Area	Area %
1	21.052	11818360	51.861
2	24.396	10970093	48.139
		22788452	100.000

Chiral

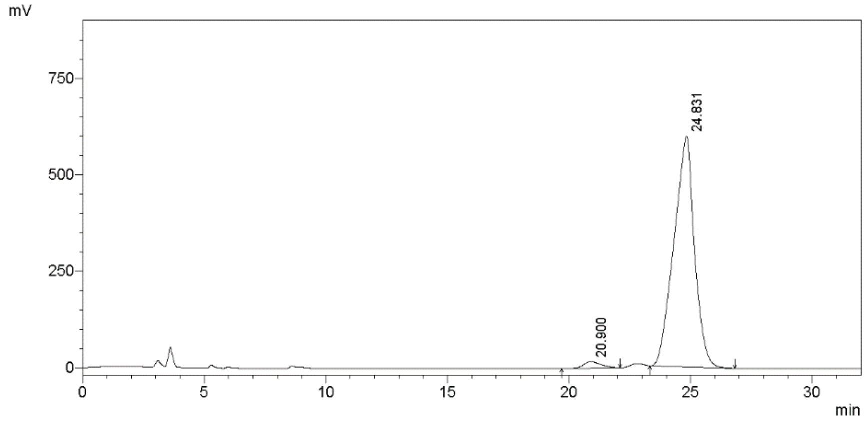

Detector A Ch1 230nm

Peak	Time	Area	Area %
1	20.900	851358	2.369
2	24.831	35085076	97.631
		35936434	100.000

Supplementary Figure 83. HPLC data of compound **3j**

Compound 3k (Fig. 4)

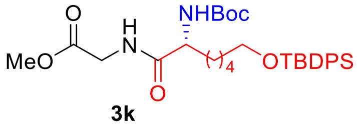

3k

HPLC Conditions

Column: Chiralcel OD-H, Daicel Chemical Industries, Ltd.

Eluent: Hexanes/Isopropanol (95:5)

Flow rate: 1.0 mL/min

Detection: UV 214 nm

Racemic

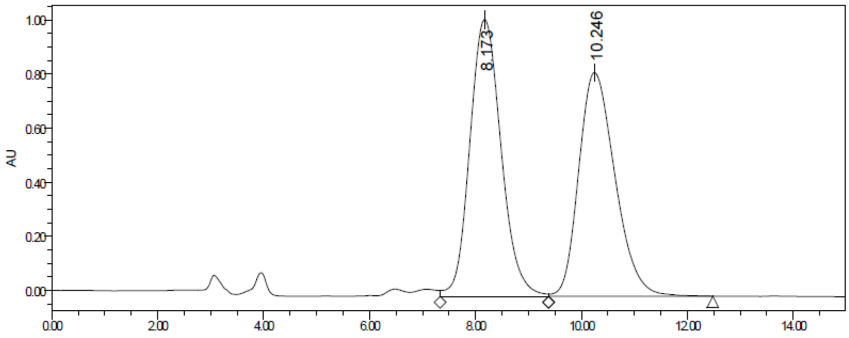

Detector A Ch1 214nm

	ret.time	area	area%
1	8.173	42012443	51.649
2	10.246	39330266	48.351
Sum		81342709	100.000

Chiral

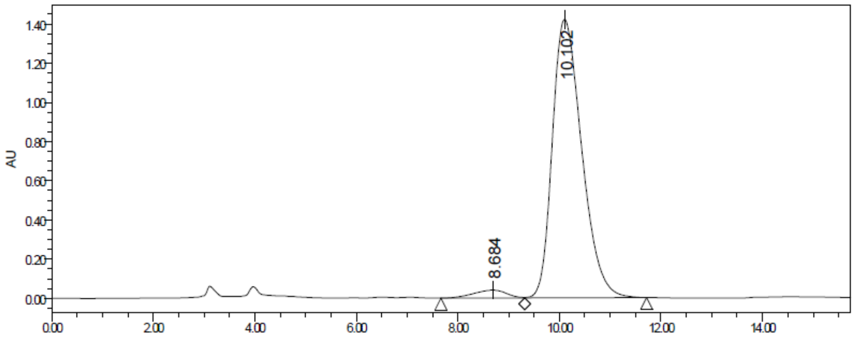

Detector A Ch1 214nm

	ret.time	area	area%
1	8.684	1844574	3.125
2	10.102	57184164	96.875
Sum		59028738	100.000

Supplementary Figure 84. HPLC data of compound **3k**

Compound 3l (Fig. 4)

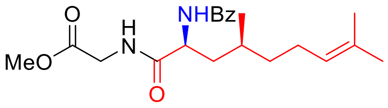

HPLC Conditions

Column: Chiralcel AD-H, Daicel Chemical Industries, Ltd.

Eluent: Hexanes/Isopropanol (90:10)

Flow rate: 1.0 mL/min

Detection: UV 254 nm

Transamination catalyzed by achiral catalyst 7

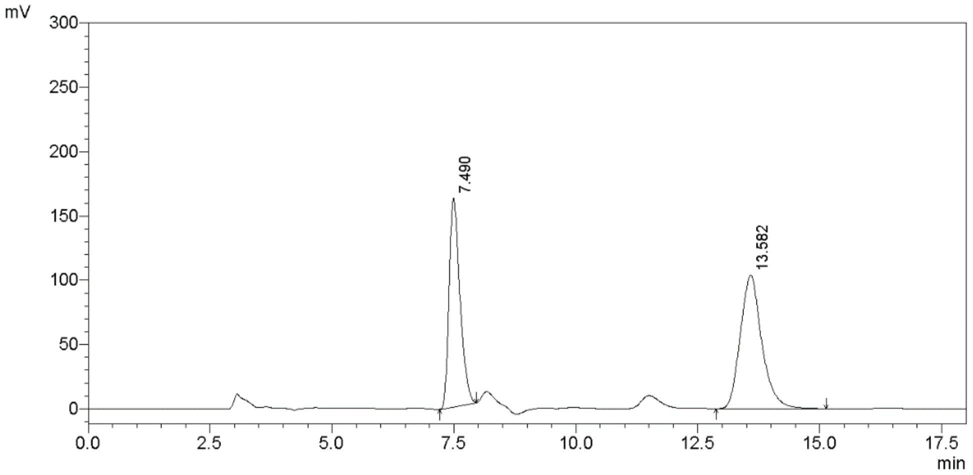

Detector A Ch1 254nm

Peak#	Time	Area	Area %
1	7.490	2519976	44.223
2	13.582	3178343	55.777
		5698319	100.000

Chiral

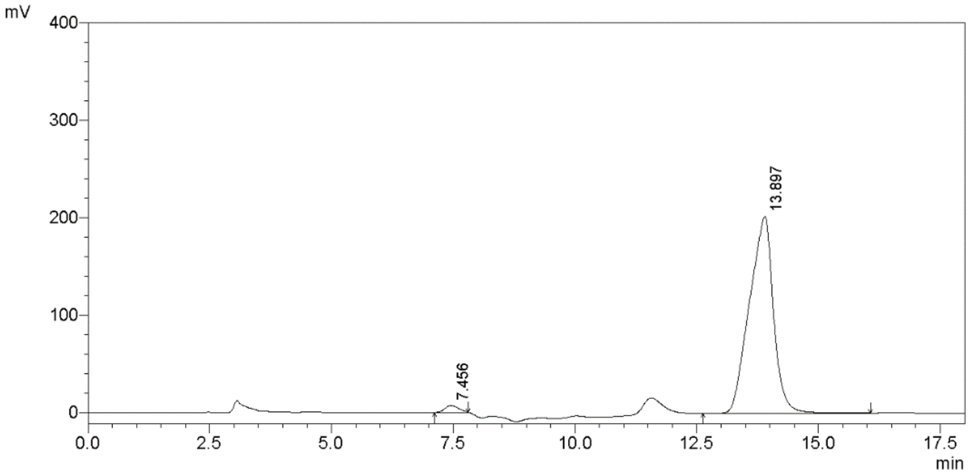

Detector A Ch1 254nm

Peak#	Time	Area	Area %
1	7.456	156945	2.214
2	13.897	6932967	97.786
		7089913	100.000

Supplementary Figure 85. HPLC data of compound **3l**

Compound (*S*,*R*)-3m (Fig. 4)

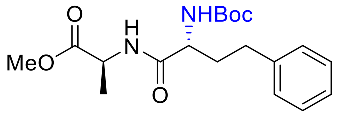

(*S*,*R*)-**3m**

HPLC Conditions
Column: Chiralcel AD-H, Daicel Chemical Industries, Ltd.
Eluent: Hexanes/Isopropanol (92:8)
Flow rate: 0.8 mL/min
Detection: UV 254 nm

Transamination catalyzed by achiral catalyst 7

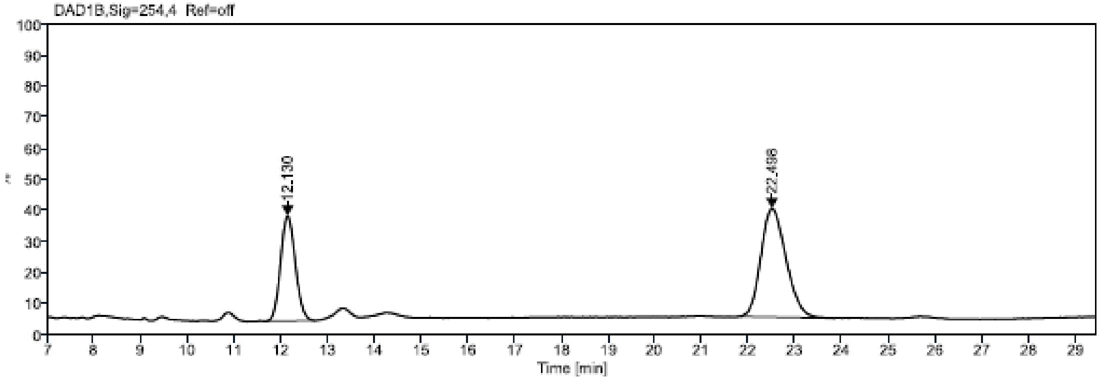

Signal:	DAD1B,Sig=254,4 Ref=off				
RT [min]	Type	Width [min]	Area	Height	Area%
12.130	BB	1.07	47.60	2.18	36.19
22.498	BB	2.02	83.92	2.25	63.81
		Sum	131.52		

Chiral

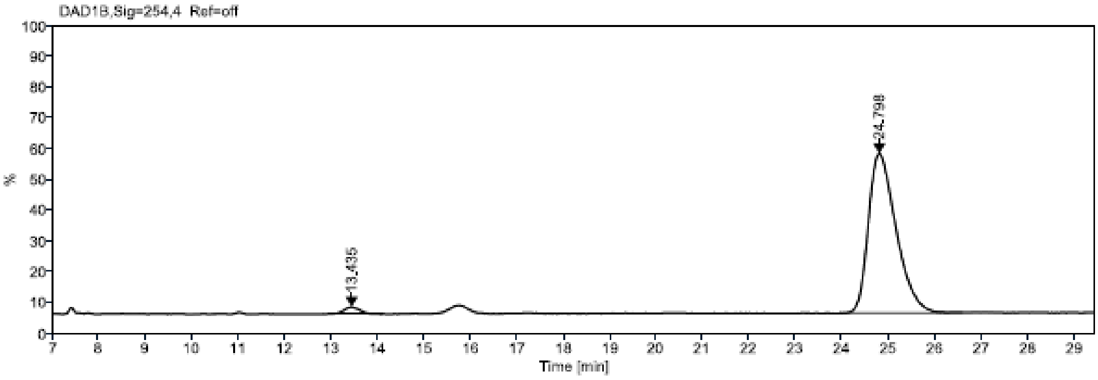

Signal:	DAD1B,Sig=254,4 Ref=off				
RT [min]	Type	Width [min]	Area	Height	Area%
13.435	MM m	0.29	6.09	0.26	2.36
24,798	MM m	0,61	252,23	6,09	97,64
		Sum	258.32		

Supplementary Figure 86. HPLC data of compound (*S*,*R*)-**3m**

Compound (*S*,*S*)-3m (Fig. 4)

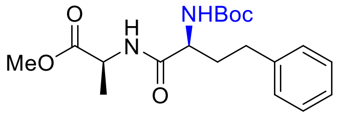

(*S*,*S*)-**3m**

HPLC Conditions

Column: Chiralcel AD-H, Daicel Chemical Industries, Ltd.
Eluent: Hexanes/Isopropanol (92:8)
Flow rate: 0.8 mL/min
Detection: UV 254 nm

Transamination catalyzed by achiral catalyst 7

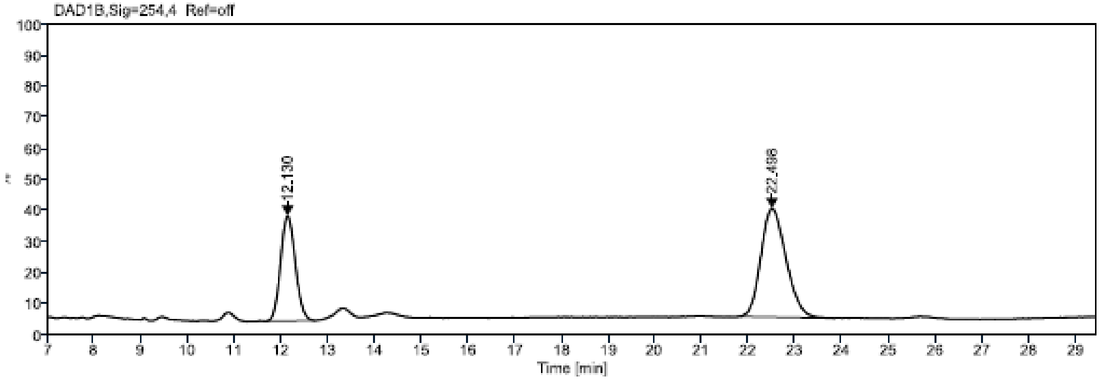

Signal: DAD1B,Sig=254,4 Ref=off

RT [min]	Type	Width [min]	Area	Height	Area%
12.130	BB	1.07	47.60	2.18	36.19
22.498	BB	2.02	83.92	2.25	63.81
		Sum	131.52		

Chiral

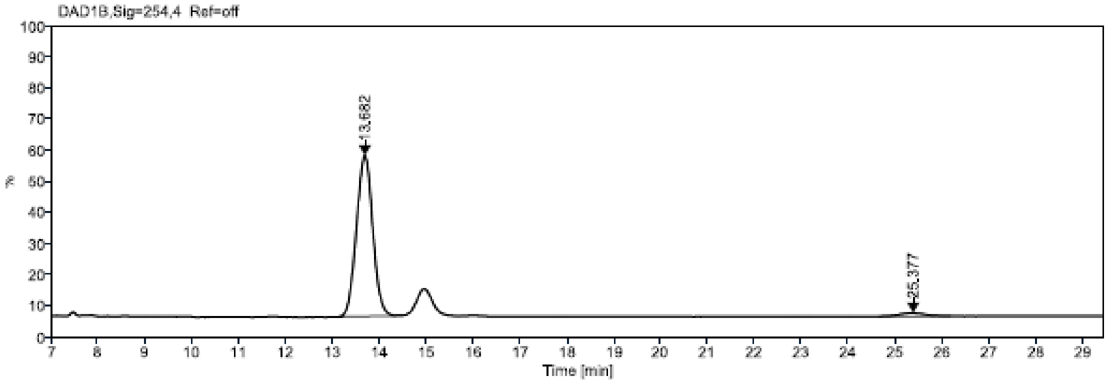

Signal: DAD1B,Sig=254,4 Ref=off

RT [min]	Type	Width [min]	Area	Height	Area%
13.682	MM m	0.38	230.45	9.55	96.97
25.377	MM m	0.46	7.19	0.19	3.03
		Sum	237.64		

Supplementary Figure 87. HPLC data of compound (*S*,*S*)-**3m**

Compound (*R*,*R*)-3n (Fig. 4)

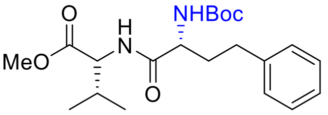

(*R*,*R*)-**3n**

HPLC Conditions

Column: Chiralcel AD-H, Daicel Chemical Industries, Ltd.
Eluent: Hexanes/Isopropanol (90:10)
Flow rate: 1.0 mL/min
Detection: UV 254 nm

Transamination catalyzed by achiral catalyst 7

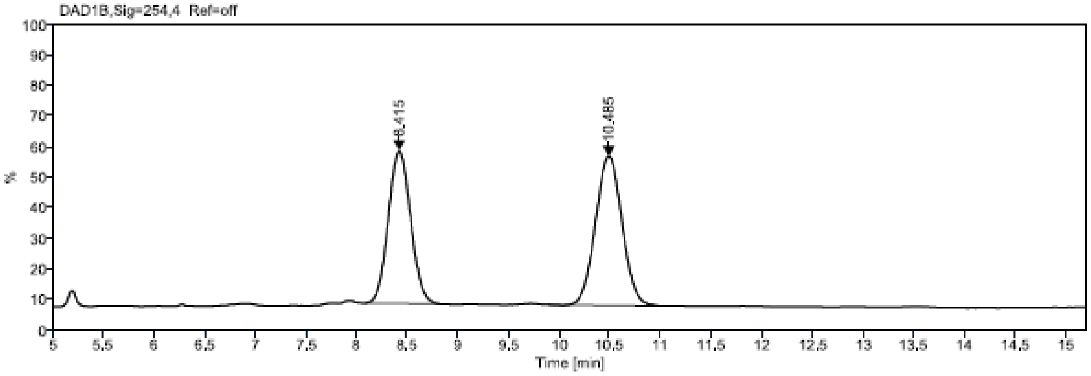

Signal: DAD1B,Sig=254,4 Ref=off

RT [min]	Type	Width [min]	Area	Height	Area%
8.415	BM m	0.24	71.10	4.65	46.07
10.485	MB m	0.29	83.22	4.56	53.93
		Sum	154.32		

Chiral

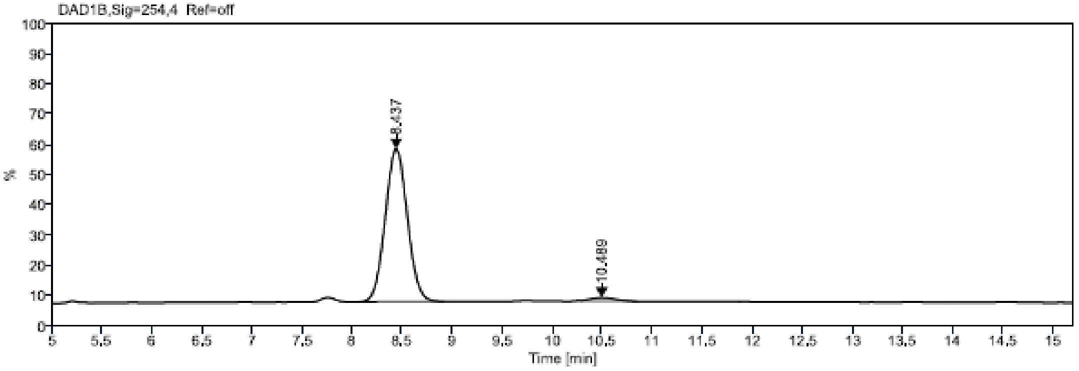

Signal: DAD1B,Sig=254,4 Ref=off

RT [min]	Type	Width [min]	Area	Height	Area%
8.437	BB	0.90	129.99	8.43	97.61
10.489	MM m	0.21	3.18	0.19	2.39
		Sum	133.17		

Supplementary Figure 88. HPLC data of compound (*R*,*R*)-**3n**

Compound (*R*,*S*)-3n (Fig. 4)

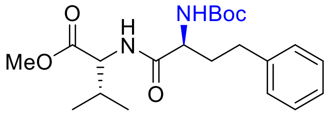

(*R*,*S*)-**3n**

HPLC Conditions
Column: Chiralcel AD-H, Daicel Chemical Industries, Ltd.
Eluent: Hexanes/Isopropanol (90:10)
Flow rate: 1.0 mL/min
Detection: UV 254 nm

Transamination catalyzed by achiral catalyst 7

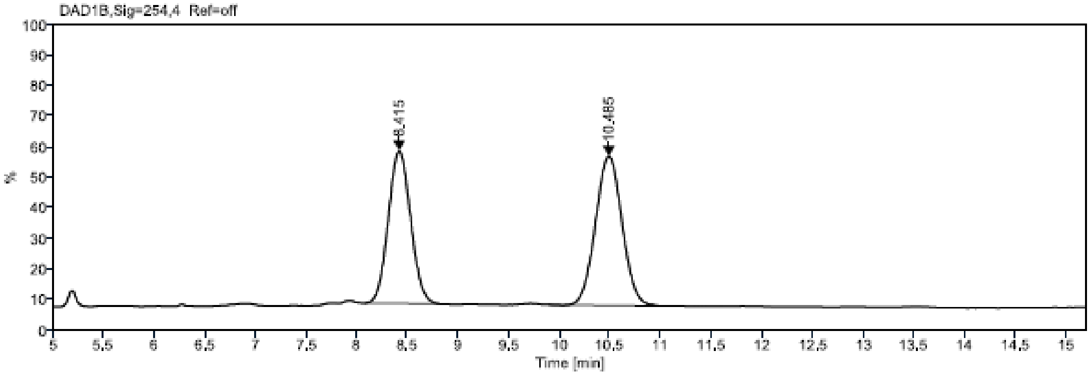

Signal: DAD1B,Sig=254,4 Ref=off

RT [min]	Type	Width [min]	Area	Height	Area%
8.415	BM m	0.24	71.10	4.65	46.07
10.485	MB m	0.29	83.22	4.56	53.93
		Sum	154.32		

Chiral

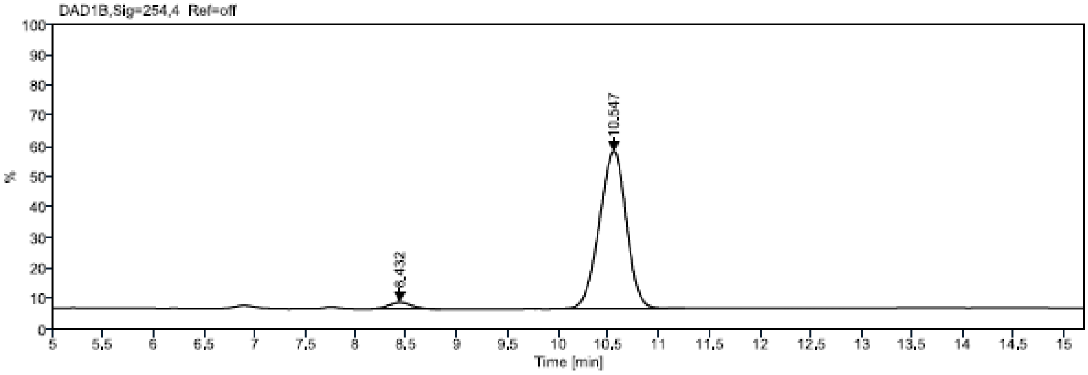

Signal: DAD1B,Sig=254,4 Ref=off

RT [min]	Type	Width [min]	Area	Height	Area%
8.432	MM m	0.23	10.11	0.67	3.32
10.547	BB	1.21	294.19	15.82	96.68
		Sum	304.31		

Supplementary Figure 89. HPLC data of compound (*R*,*S*)-**3n**

Compound 3o (Fig. 4)

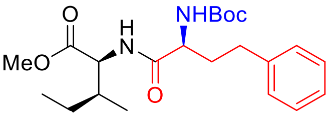

3o

HPLC Conditions

Column: Chiralcel AD-H, Daicel Chemical Industries, Ltd.

Eluent: Hexanes/Isopropanol (90:10)

Flow rate: 1.0 mL/min

Detection: UV 254 nm

Transamination catalyzed by achiral catalyst 7

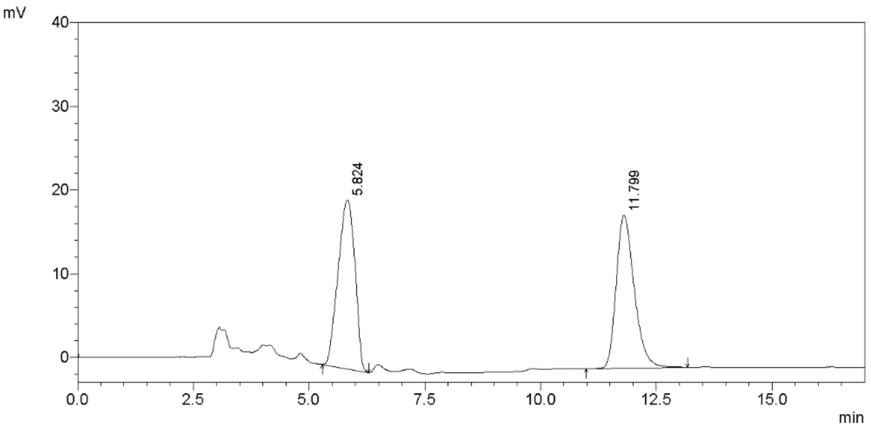

Detector A Ch1 254nm

Peak	Time	Area	Area %
1	5.824	510884	50.719
2	11.799	496394	49.281
		1007279	100.000

Chiral

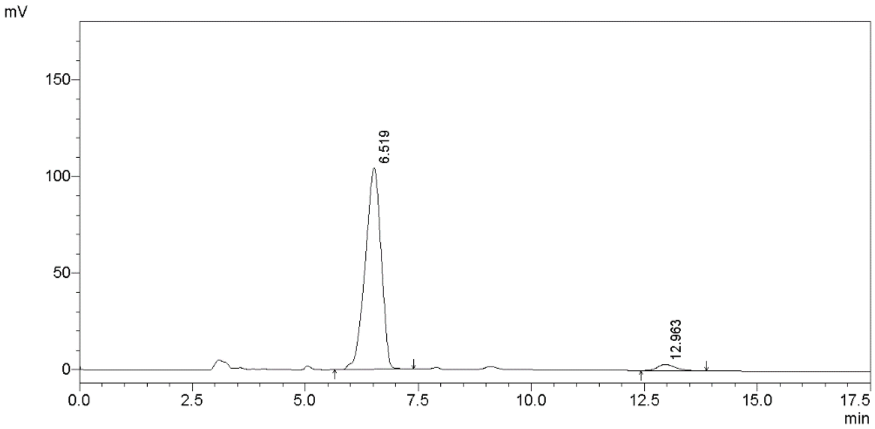

Detector A Ch1 254nm

Peak	Time	Area	Area %
1	6.519	2545074	96.576
2	12.963	90246	3.424
		2635320	100.000

Supplementary Figure 90. HPLC data of compound **3o**

Compound 3p (Fig. 4)

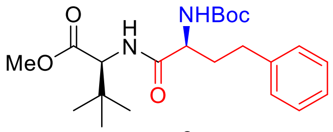

3p

HPLC Conditions

Column: Chiralcel AD-H, Daicel Chemical Industries, Ltd.

Eluent: Hexanes/Isopropanol (90:10)

Flow rate: 1.0 mL/min

Detection: UV 254 nm

Transamination catalyzed by achiral catalyst 7

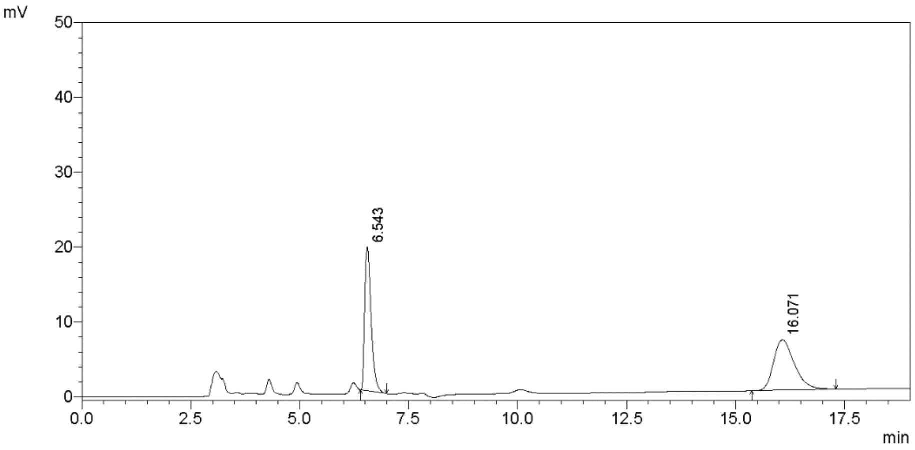

Detector A Ch1 254nm

Peak	Time	Area	Area %
1	6.543	194276	46.433
2	16.071	224126	53.567
		418401	100.000

Chiral

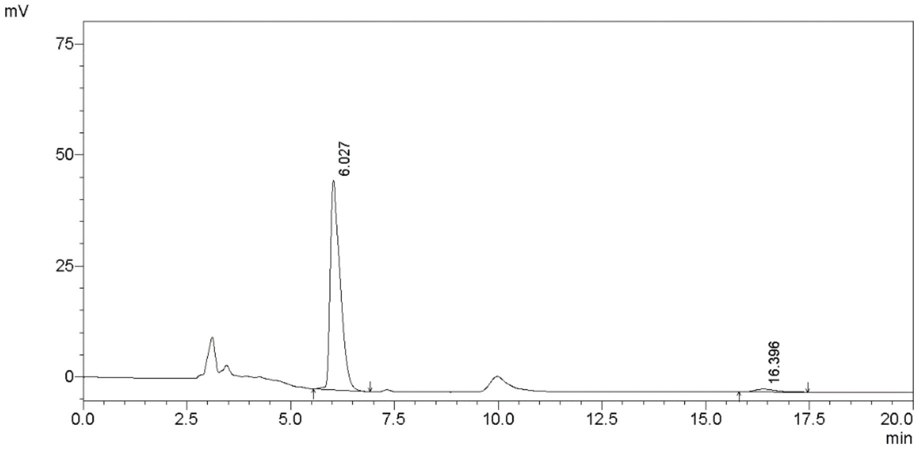

Detector A Ch1 254nm

Peak	Time	Area	Area %
1	6.027	788073	97.081
2	16.396	23693	2.919
		811766	100.000

Supplementary Figure 91. HPLC data of compound **3p**

Compound 3q (Fig. 4)

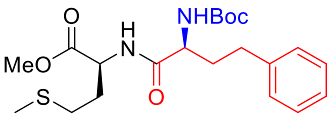

3q

HPLC Conditions

Column: Chiralcel AD-H, Daicel Chemical Industries, Ltd.
Eluent: Hexanes/Isopropanol (90:10)
Flow rate: 1.0 mL/min
Detection: UV 254 nm

Transamination catalyzed by achiral catalyst 7

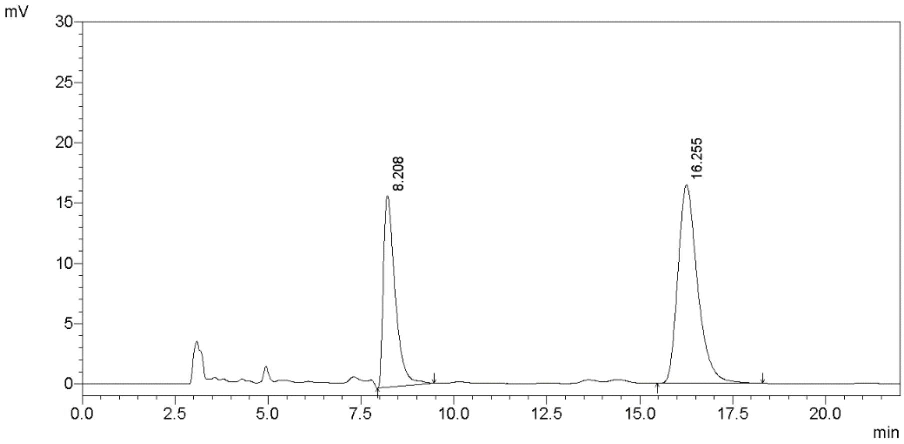

Detector A Ch1 254nm

Peak	Time	Area	Area %
1	8.208	349272	36.623
2	16.255	604432	63.377
		953704	100.000

Chiral

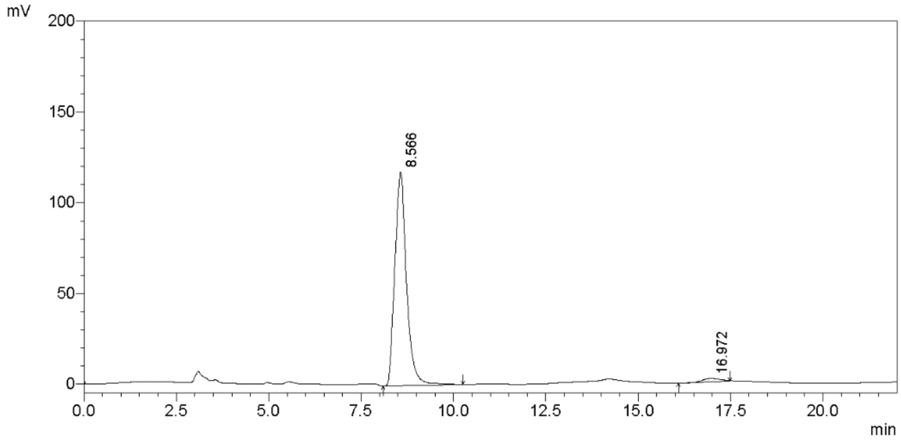

Detector A Ch1 254nm

Peak	Time	Area	Area %
1	8.566	2574653	97.268
2	16.972	72317	2.732
		2646969	100.000

Supplementary Figure 92. HPLC data of compound **3q**

Compound (*S*,*R*)-3r (Fig. 4)

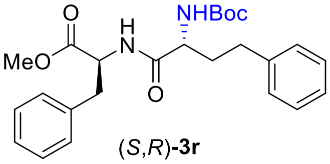

(*S*,*R*)-**3r**

HPLC Conditions

Column: Chiralcel AD-H, Daicel Chemical Industries, Ltd.
Eluent: Hexanes/Isopropanol (88:12)
Flow rate: 1.0 mL/min
Detection: UV 254 nm

Transamination catalyzed by achiral catalyst 7

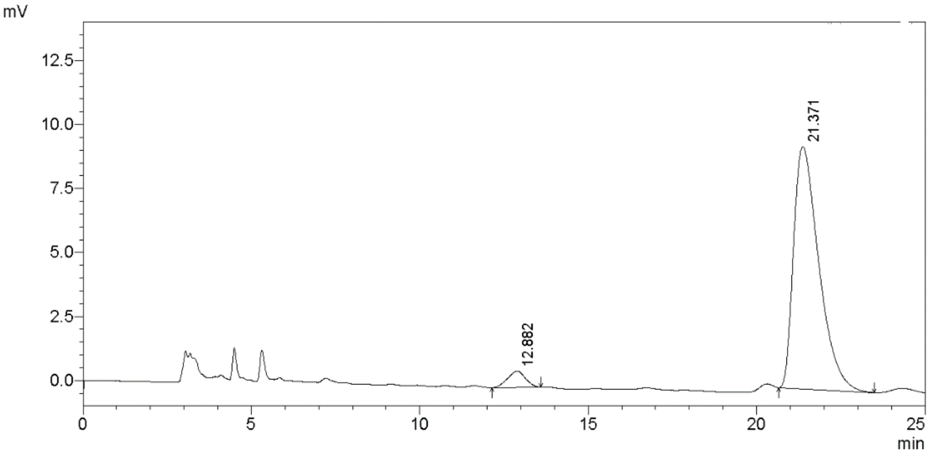

Detector A Ch1 254nm

Peak	Time	Area	Area %
1	12.882	23437	4.523
2	21.371	494711	95.477
		518148	100.000

Chiral

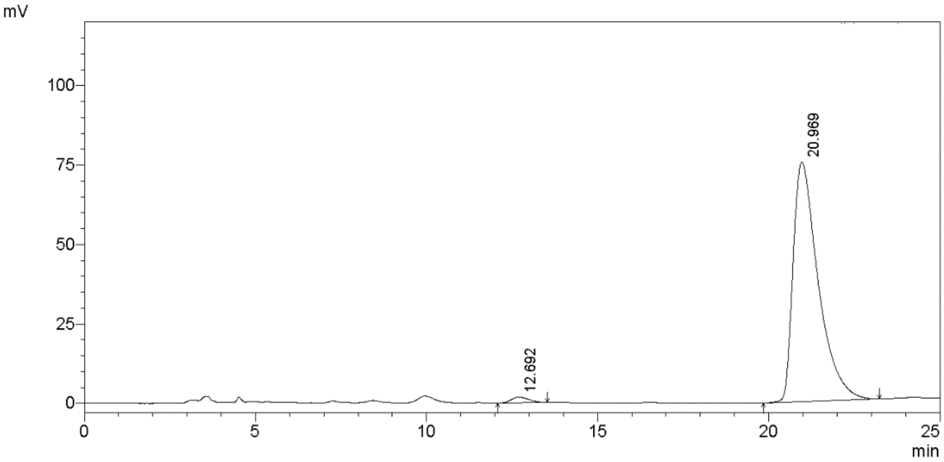

Detector A Ch1 254nm

Peak	Time	Area	Area %
1	12.692	64677	1.649
2	20.969	3858241	98.351
		3922918	100.000

Supplementary Figure 93. HPLC data of compound (*S*,*R*)-**3r**

Compound (*S*,*S*)-3r (Fig. 4)

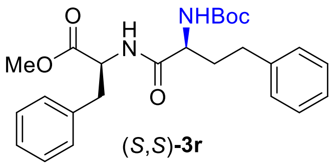

(*S*,*S*)-**3r**

HPLC Conditions
Column: Chiralcel AD-H, Daicel Chemical Industries, Ltd.
Eluent: Hexanes/Isopropanol (88:12)
Flow rate: 1.0 mL/min
Detection: UV 254 nm

Transamination catalyzed by achiral catalyst 7

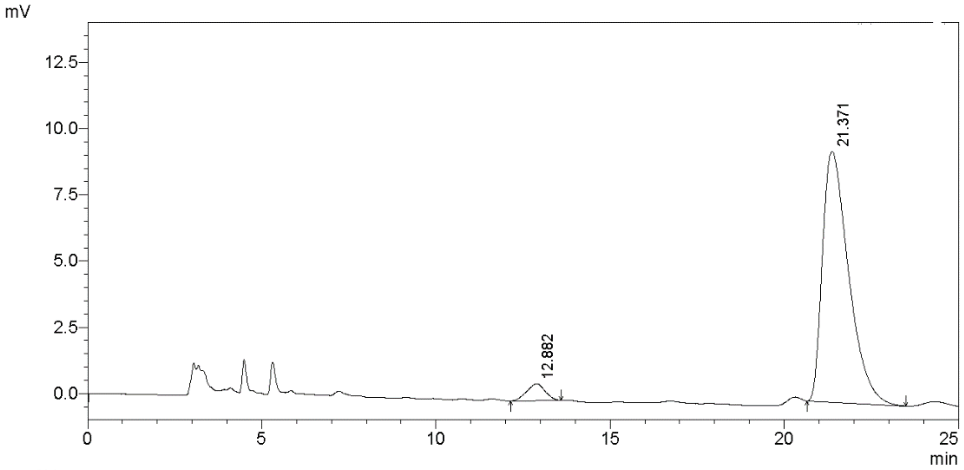

Detector A Ch1 254nm

Peak	Time	Area	Area %
1	12.882	23437	4.523
2	21.371	494711	95.477
		518148	100.000

Chiral

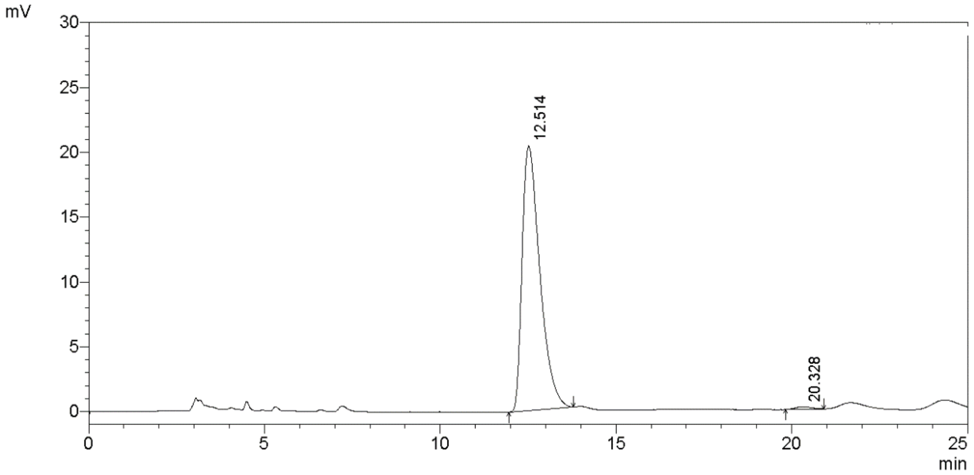

Peak#	Time	Area	Area %
1	12.514	715436	99.160
2	20.328	6060	0.840
		721496	100.000

Supplementary Figure 94. HPLC data of compound (*S*,*S*)-**3r**

Compound 3s (Fig. 4)

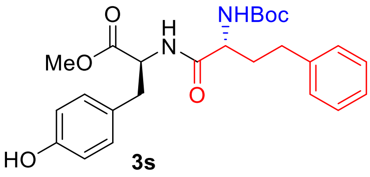

HPLC Conditions

Column: Chiralcel AD-H, Daicel Chemical Industries, Ltd.

Eluent: Hexanes/Isopropanol (88:12)

Flow rate: 1.0 mL/min

Detection: UV 254 nm

Transamination catalyzed by achiral catalyst 7

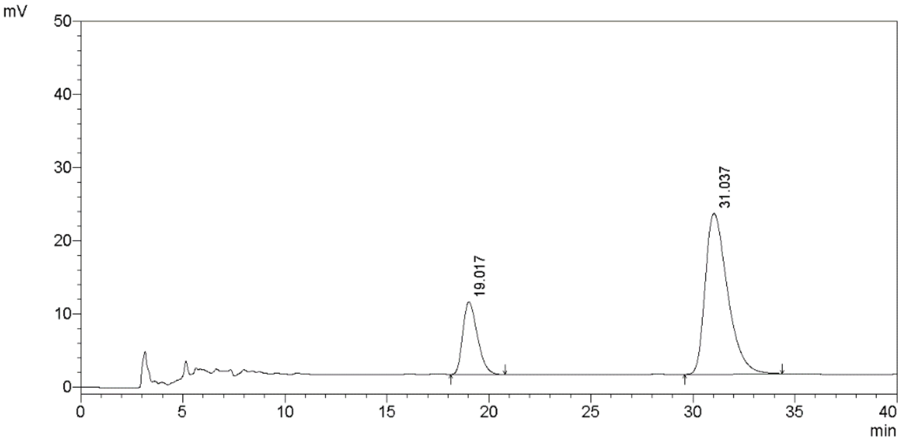

Detector A Ch1 254nm

Peak	Time	Area	Area %
1	19.017	491660	22.729
2	31.037	1671473	77.271
		2163133	100.000

Chiral

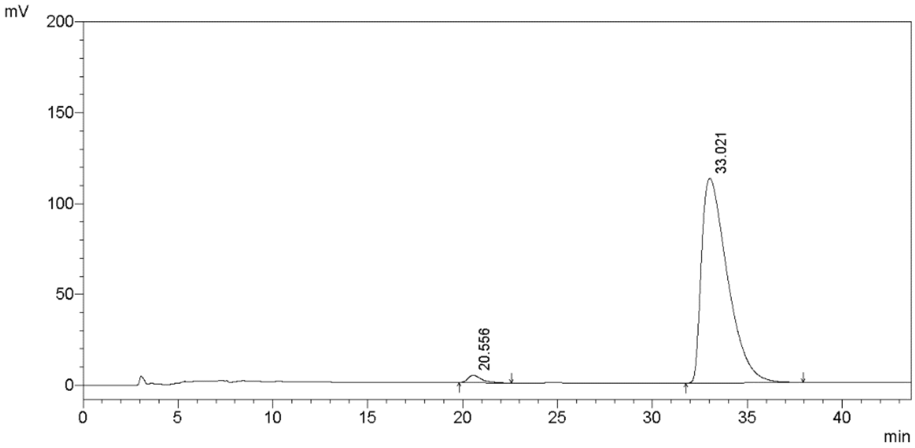

Detector A Ch1 254nm

Peak	Time	Area	Area %
1	20.556	201638	1.843
2	33.021	10736273	98.157
		10937911	100.000

Supplementary Figure 95. HPLC data of compound **3s**

Compound 3t (Fig. 4)

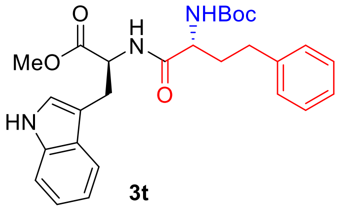

HPLC Conditions

Column: Chiralcel AD-H, Daicel Chemical Industries, Ltd.

Eluent: Hexanes/Isopropanol (88:12)

Flow rate: 1.0 mL/min

Detection: UV 230 nm

Transamination catalyzed by achiral catalyst 7

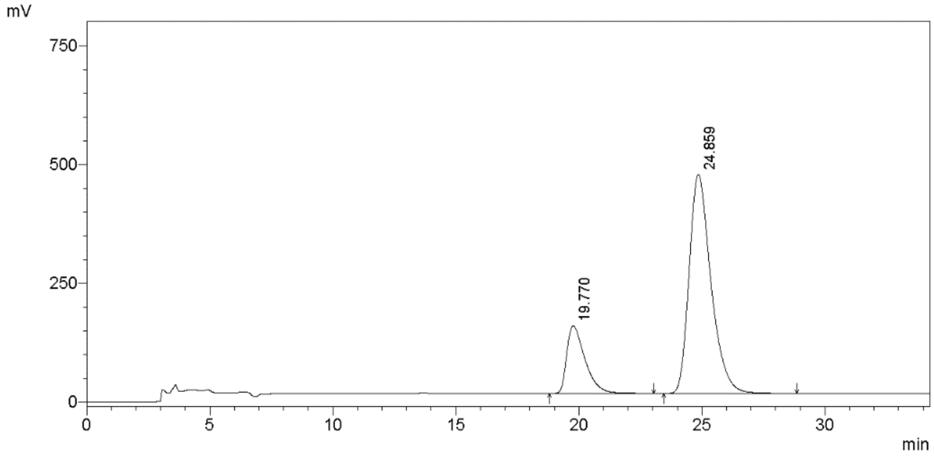

Detector A Ch1 230nm

Peak	Time	Area	Area %
1	19.770	7316465	20.469
2	24.859	28427326	79.531
		35743790	100.000

Chiral

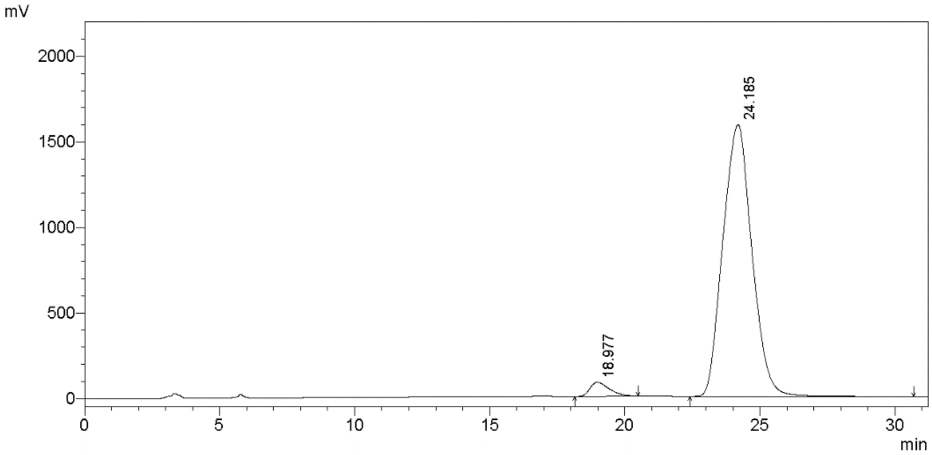

Detector A Ch1 230nm

Peak	Time	Area	Area %
1	18.977	4038864	3.296
2	24.185	118509997	96.704
		122548861	100.000

Supplementary Figure 96. HPLC data of compound **3t**

Compound 3u (Fig. 4)

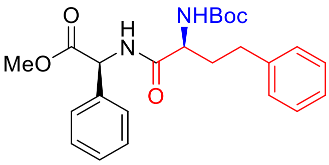

3u

HPLC Conditions

Column: Chiralcel AD-H, Daicel Chemical Industries, Ltd.

Eluent: Hexanes/Isopropanol (90:10)

Flow rate: 1.0 mL/min

Detection: UV 254 nm

Transamination catalyzed by achiral catalyst 7

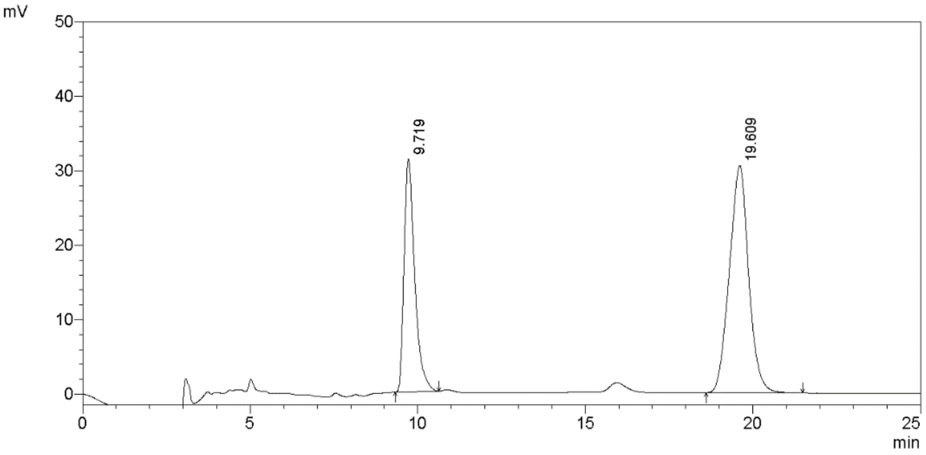

Detector A Ch1 254nm

Peak	Time	Area	Area %
1	9.719	690469	36.138
2	19.609	1220189	63.862
		1910658	100.000

Chiral

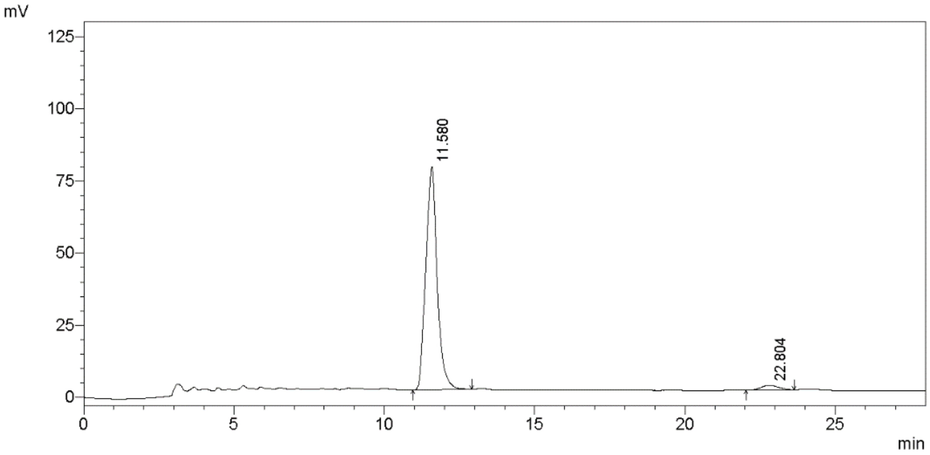

Detector A Ch1 254nm

Peak	Time	Area	Area %
1	11.580	2035800	96.914
2	22.804	64833	3.086
		2100632	100.000

Supplementary Figure 97. HPLC data of compound **3u**

Compound (*S*,*R*)-3v (Fig. 4)

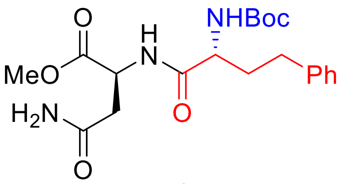

(*S*,*R*)-**3v**

HPLC Conditions
Column: Chiralcel OD-3, Daicel Chemical Industries, Ltd.
Eluent: Hexanes/Isopropanol (92:8)
Flow rate: 0.7 mL/min
Detection: UV 214 nm

Transamination catalyzed by achiral catalyst 7

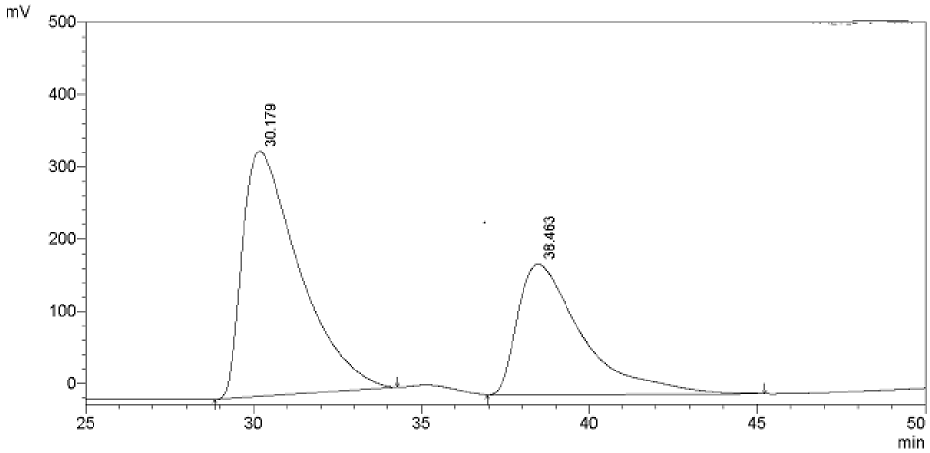

Detector A Ch1 214nm

Peak#	Time	Area	Area %
1	30.179	40109018	62.302
2	38.463	24269356	37.698
		64378374	100.000

Chiral

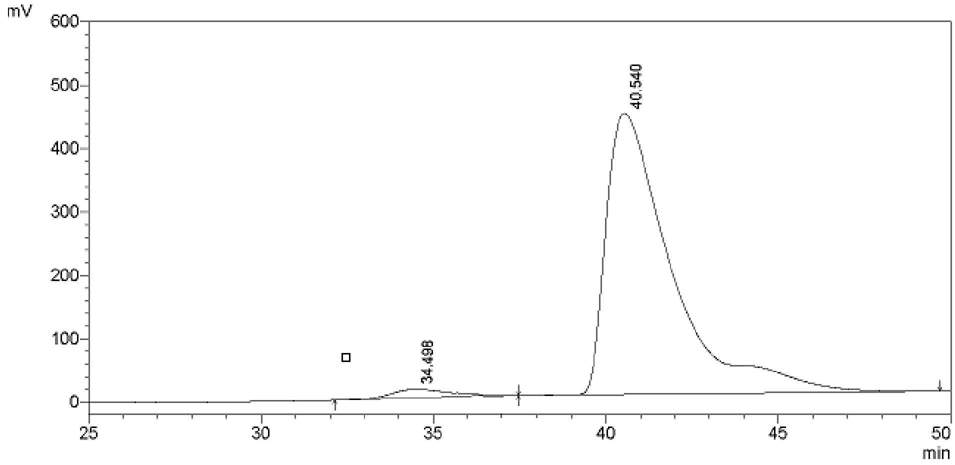

Detector A Ch1 214nm

Peak#	Time	Area	Area %
1	34.498	1621828	2.689
2	40.540	58698995	97.311
		60320823	100.000

Supplementary Figure 98. HPLC data of compound (*S*,*R*)-**3v**

Compound (*S*,*S*)-3v (Fig. 4)

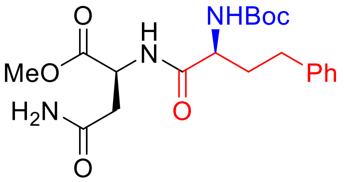

(*S*,*S*)-**3v**

HPLC Conditions
Column: Chiralcel OD-3, Daicel Chemical Industries, Ltd.
Eluent: Hexanes/Isopropanol (92:8)
Flow rate: 0.7 mL/min
Detection: UV 214 nm

Transamination catalyzed by achiral catalyst 7

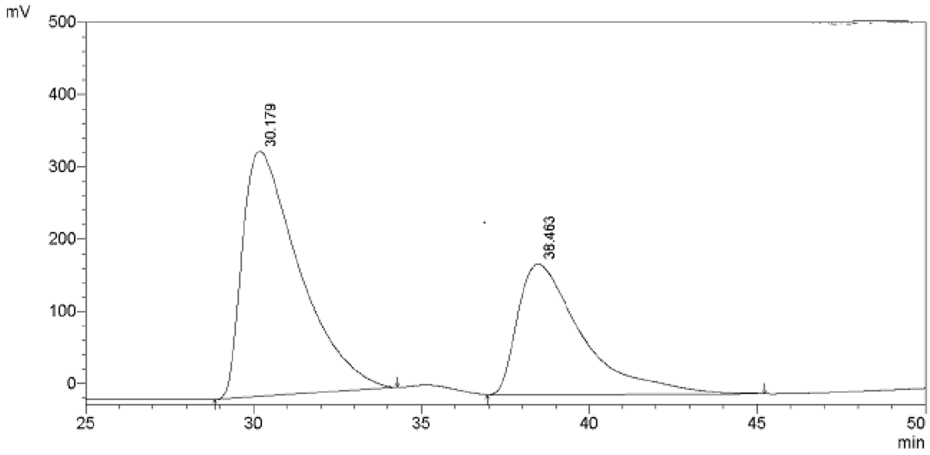

Detector A Ch1 214nm

Peak#	Time	Area	Area %
1	30.179	40109018	62.302
2	38.463	24269356	37.698
		64378374	100.000

Chiral

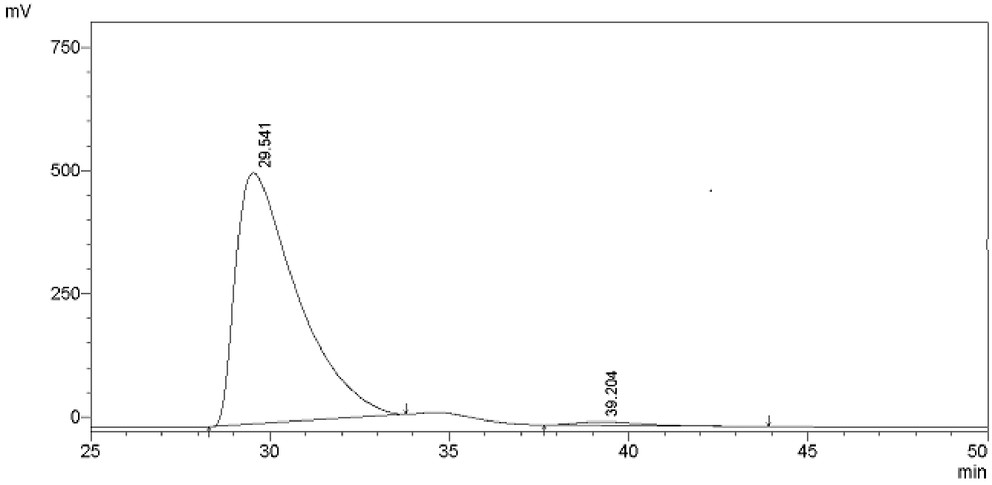

Detector A Ch1 214nm

Peak#	Time	Area	Area %
1	29.541	60810586	98.487
2	39.204	934098	1.513
		61744685	100.000

Supplementary Figure 99. HPLC data of compound (*S*,*S*)-**3v**

Compound (*S*,*R*)-3w (Fig. 4)

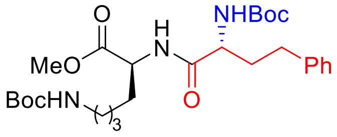

(*S*,*R*)-**3w**

HPLC Conditions

Column: Chiralcel AD-H, Daicel Chemical Industries, Ltd.

Eluent: Hexanes/Isopropanol (90:10)

Flow rate: 1.0 mL/min

Detection: UV 230 nm

Transamination catalyzed by achiral catalyst 7

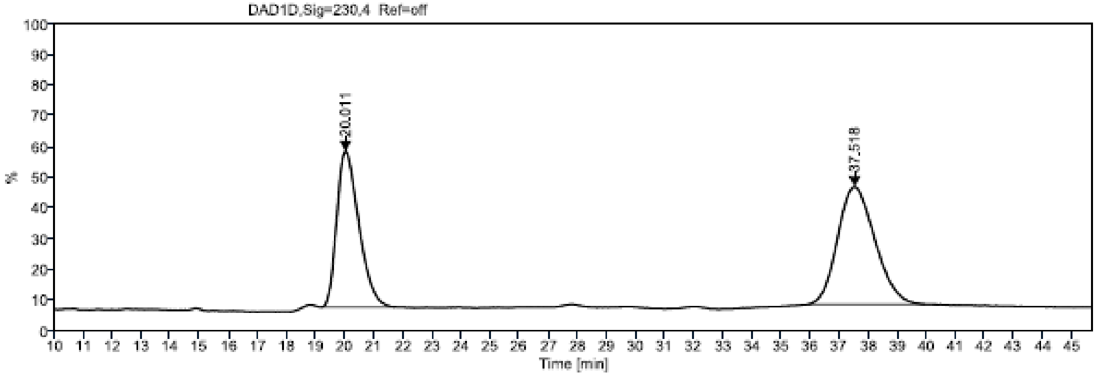

Signal: DAD1D,Sig=230,4 Ref=off

RT [min]	Type	Width [min]	Area	Height	Area%
20.011	BB	2.52	195.20	3.70	43.93
37.518	BB	3.99	249.15	2.79	56.07
		Sum	444.36		

Chiral

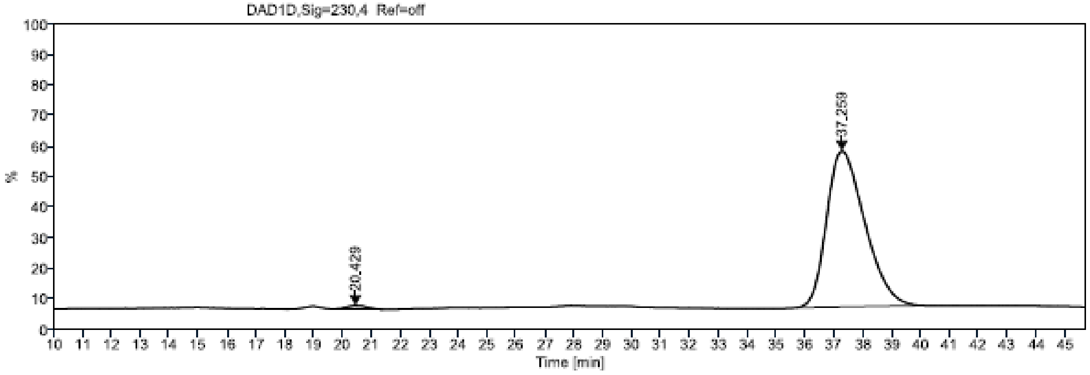

Detector A Ch1 230 nm

Signal: DAD1D,Sig=230,4 Ref=off

RT [min]	Type	Width [min]	Area	Height	Area%
20.429	MM m	0.56	8.95	0.19	1.22
37.259	BB	4.36	723.86	7.88	98.78
		Sum	732.81		

Supplementary Figure 100. HPLC data of compound (*S*,*R*)-**3w**

Compound (*S*,*S*)-3w (Fig. 4)

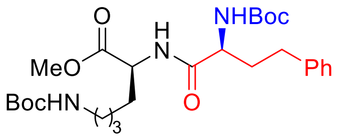

(*S*,*S*)-**3w**

HPLC Conditions

Column: Chiralcel AD-H, Daicel Chemical Industries, Ltd.
Eluent: Hexanes/Isopropanol (90:10)
Flow rate: 1.0 mL/min
Detection: UV 230 nm

Transamination catalyzed by achiral catalyst 7

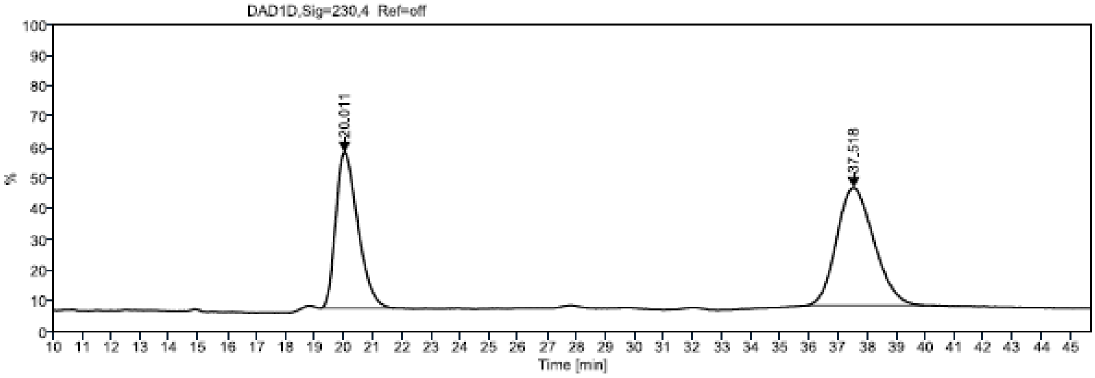

Signal:	DAD1D,Sig=230,4 Ref=off				
RT [min]	Type	Width [min]	Area	Height	Area%
20.011	BB	2.52	195.20	3.70	43.93
37.518	BB	3.99	249.15	2.79	56.07
		Sum	444.36		

Chiral

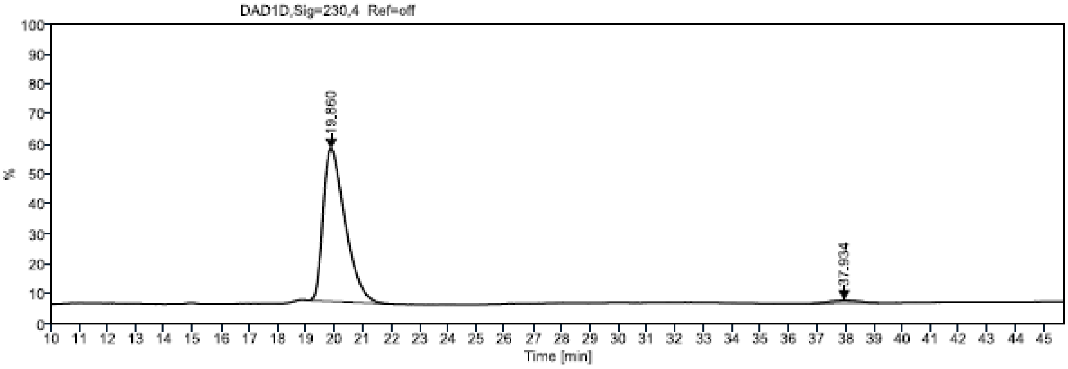

Signal:	DAD1D,Sig=230,4 Ref=off				
RT [min]	Type	Width [min]	Area	Height	Area%
19.860	BB	3.01	473.08	8.75	97.53
37.934	MM m	0.85	12.00	0.16	2.47
		Sum	485.08		

Supplementary Figure 101. HPLC data of compound (*S*,*S*)-**3w**

Compound (*S*,*R*)-3x (Fig. 4)

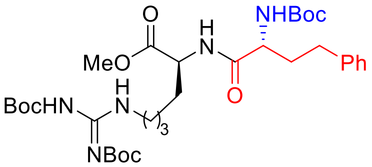

(*S*,*R*)-**3x**

HPLC Conditions

Column: Chiralcel IC, Daicel Chemical Industries, Ltd.

Eluent: Hexanes/Isopropanol (90:10)

Flow rate: 1.0 mL/min

Detection: UV 230 nm

Transamination catalyzed by achiral catalyst 7

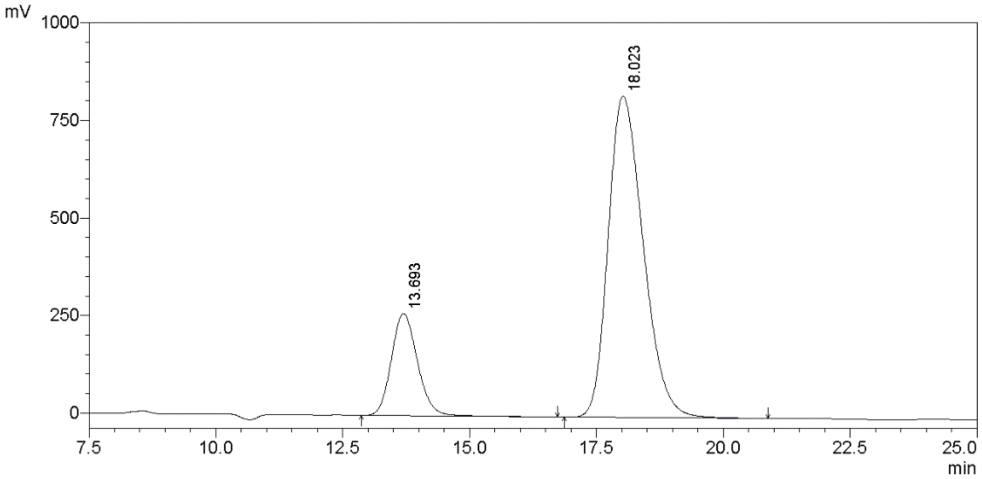

Detector A Ch1 230 nm

Peak#	Time	Area	Area %
1	13.693	9426858	19.194
2	18.023	39687352	80.806
		49114210	100.000

Chiral

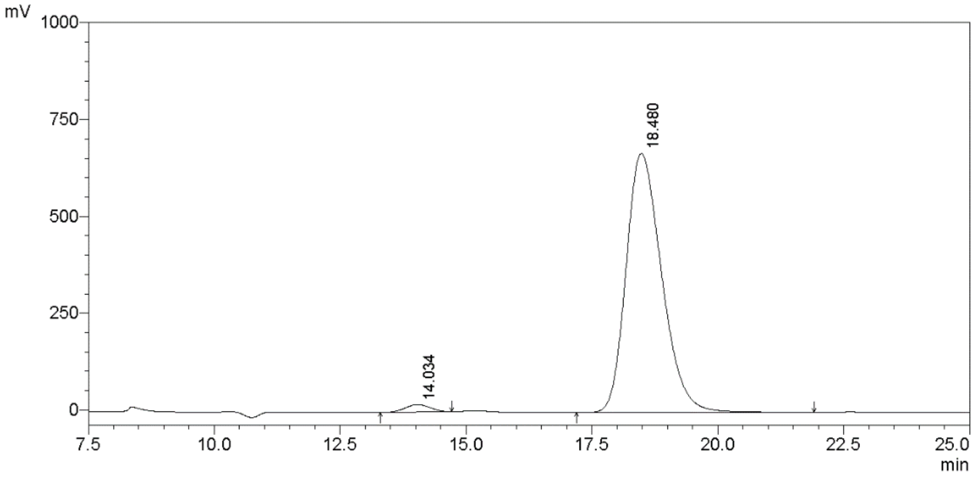

Detector A Ch1 230nm

Peak#	Time	Area	Area %
1	14.034	661063	1.968
2	18.480	32927150	98.032
		33588212	100.000

Supplementary Figure 102. HPLC data of compound (*S*,*R*)-**3w**

Compound (*S*,*S*)-3x (Fig. 4)

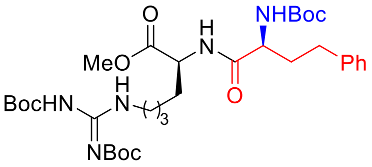

(*S*,*S*)-**3x**

HPLC Conditions
Column: Chiralcel IC, Daicel Chemical Industries, Ltd.
Eluent: Hexanes/Isopropanol (90:10)
Flow rate: 1.0 mL/min
Detection: UV 230 nm

Transamination catalyzed by achiral catalyst 7

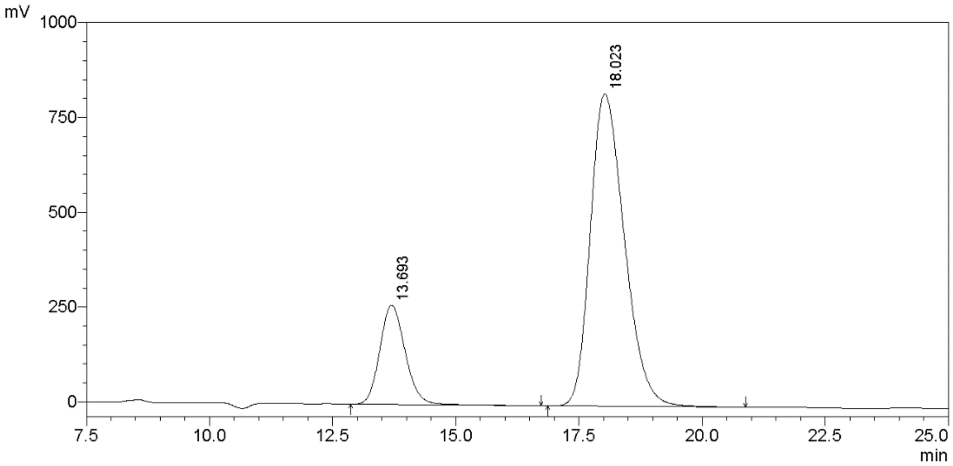

Detector A Ch1 230 nm

Peak#	Time	Area	Area %
1	13.693	9426858	19.194
2	18.023	39687352	80.806
		49114210	100.000

Chiral

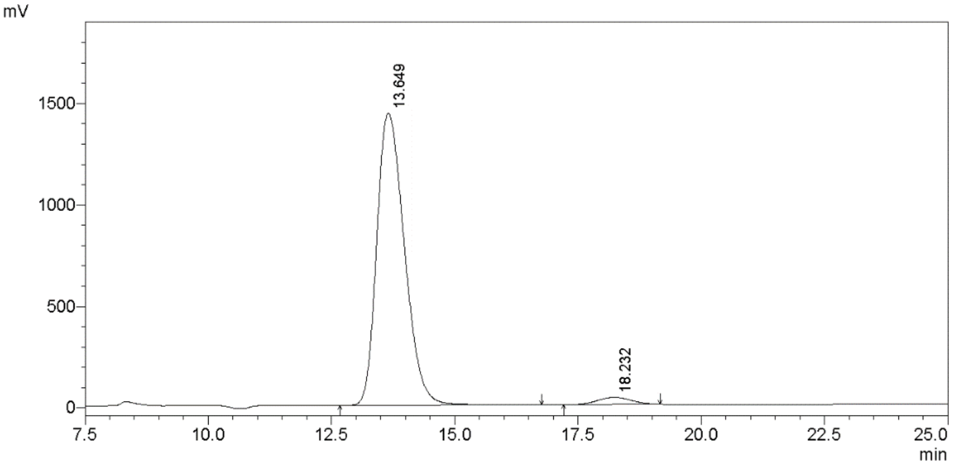

Detector A Ch1 230nm

Peak#	Time	Area	Area %
1	13.649	56193966	97.059
2	18.232	1702591	2.941
		57896557	100.000

Supplementary Figure 103. HPLC data of compound (*S*,*S*)-**3x**

Compound (*R*,*R*,*S*)-3y (Fig. 4)

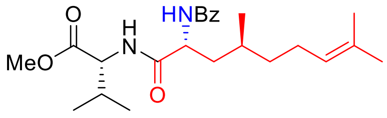

HPLC Conditions
Column: Chiralcel AD-H, Daicel Chemical Industries, Ltd.
Eluent: Hexanes/Isopropanol (90:10)
Flow rate: 1.0 mL/min
Detection: UV 230 nm

Transamination catalyzed by achiral catalyst 7

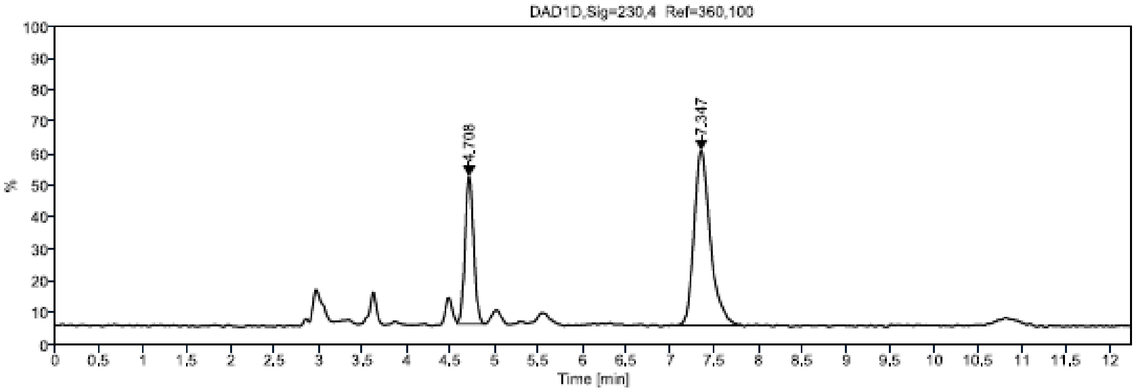

Signal: DAD1D,Sig=230,4 Ref=360,100

RT [min]	Type	Width [min]	Area	Height	Area%
4,708	MM m	0,11	103,39	15,12	30,80
7,347	MM m	0,19	232,26	17,99	69,20
		Sum	335,65		

Chiral

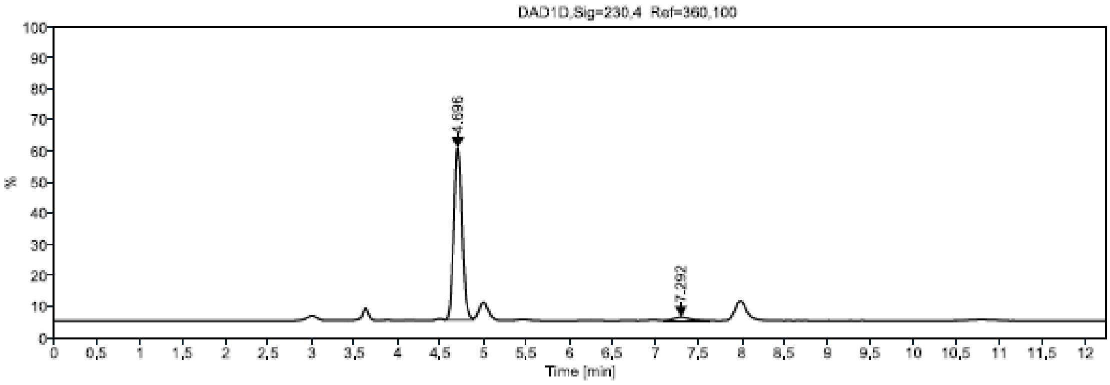

Signal: DAD1D,Sig=230,4 Ref=360,100

RT [min]	Type	Width [min]	Area	Height	Area%
4,696	MM m	0,10	1248,09	186,43	96,57
7,292	MM m	0,18	44,27	3,72	3,43
		Sum	1292,37		

Supplementary Figure 104. HPLC data of compound (*R*,*R*,*S*)-**3y**

Compound (*R*,*S*,*S*)-3y (Fig. 4)

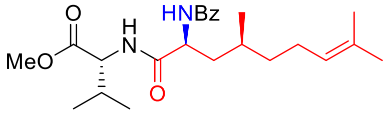

HPLC Conditions

Column: Chiralcel AD-H, Daicel Chemical Industries, Ltd.

Eluent: Hexanes/Isopropanol (90:10)

Flow rate: 1.0 mL/min

Detection: UV 230 nm

Transamination catalyzed by achiral catalyst 7

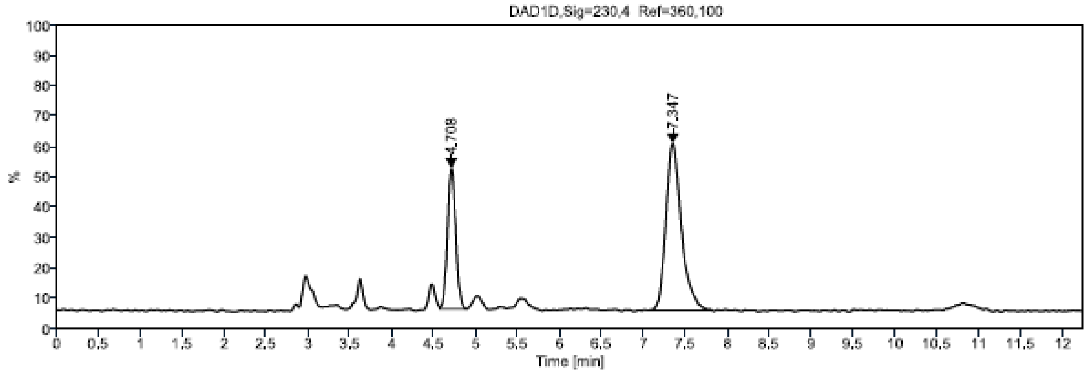

Signal: DAD1D,Sig=230,4 Ref=360,100

RT [min]	Type	Width [min]	Area	Height	Area%
4.708	MM m	0.11	103.39	15.12	30.80
7.347	MM m	0.19	232.26	17.99	69.20
		Sum	335.65		

Chiral

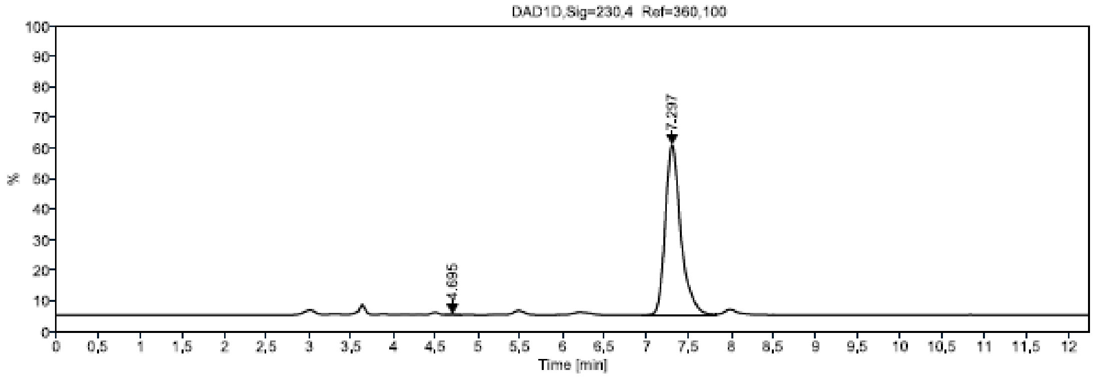

Signal: DAD1D,Sig=230,4 Ref=360,100

RT [min]	Type	Width [min]	Area	Height	Area%
4,695	MM m	0,09	1,86	0,28	0,09
7,297	MM m	0,19	2053,15	159,88	99,91
		Sum	2055,00		

Supplementary Figure 105. HPLC data of compound (*R*,*S*,*S*)-**3y**

Compound 3z (Fig. 4)

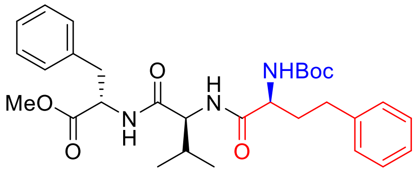

HPLC Conditions
Column: Chiralcel OD-3, Daicel Chemical Industries, Ltd.
Eluent: Hexanes/Isopropanol (97:3)
Flow rate: 0.3 mL/min
Detection: UV 230 nm

Transamination catalyzed by achiral catalyst 7

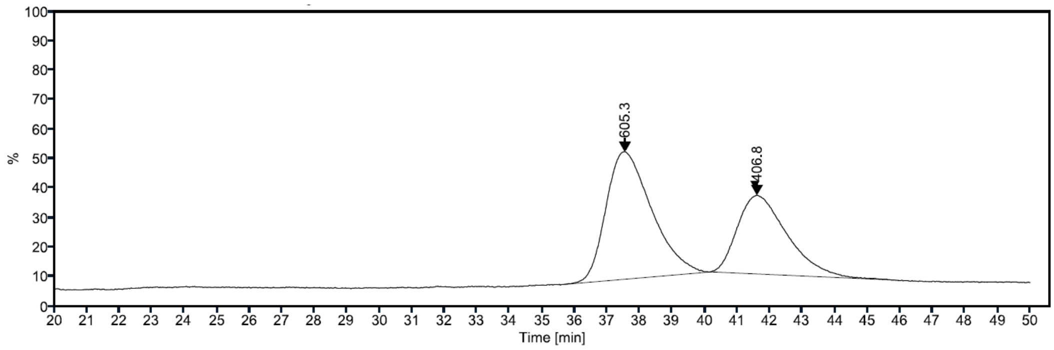

Signal: DAD1D,Sig=230,4 Ref=off

RT [min]	Type	Width [min]	Area	Height	Area%
37.539	MM m	1.14	605.31	6.21	59.81
41.602	MM m	1.25	406.76	3.84	40.19
		Sum	1012.07		

Chiral

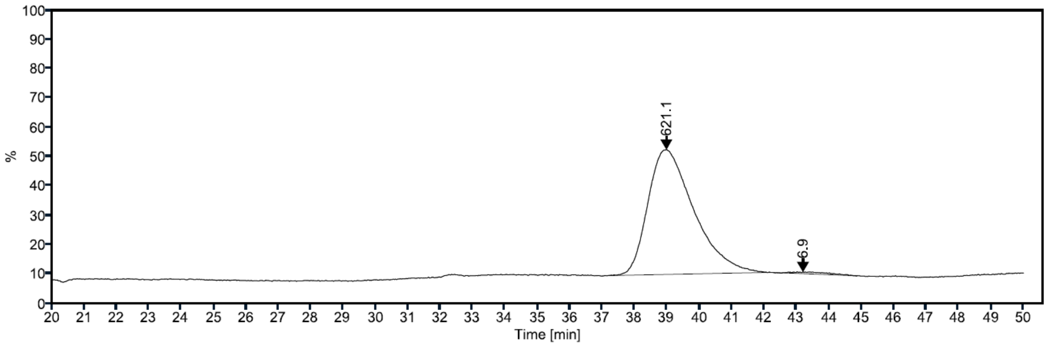

Signal: DAD1D,Sig=230,4 Ref=off

RT [min]	Type	Width [min]	Area	Height	Area%
38.978	MM m	1.16	621.12	6.31	98.90
43.194	MM m	0.99	6.92	0.08	1.10
		Sum	628.04		

Supplementary Figure 106. HPLC data of compound **3z**

Compound (*S*,*S*,*R*)-3aa (Fig. 4)

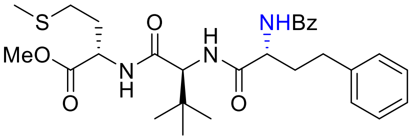

HPLC Conditions
Column: Chiralcel IC, Daicel Chemical Industries, Ltd.
Eluent: Hexanes/Isopropanol (88:12)
Flow rate: 1.0 mL/min
Detection: UV 254 nm

Transamination catalyzed by achiral catalyst 7

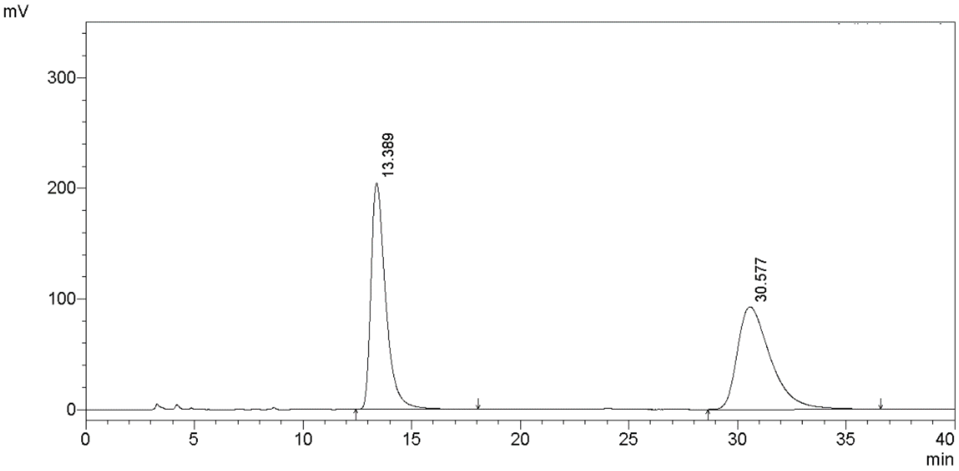

Detector A Ch1 254nm

Peak#	Time	Area	Area %
1	13.389	9634851	49.565
2	30.577	9804008	50.435
		19438859	100.000

Chiral

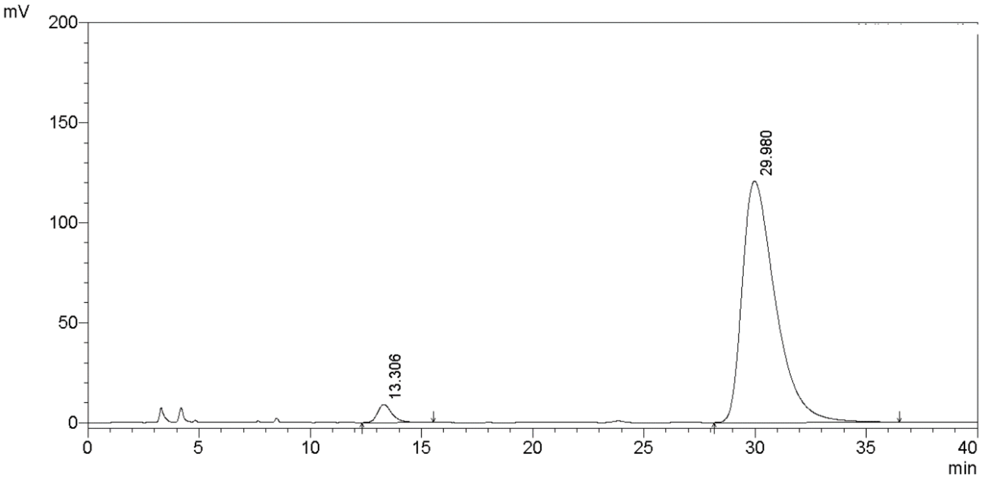

Detector A Ch1 254nm

Peak#	Time	Area	Area %
1	13.306	415951	3.213
2	29.980	12529537	96.787
		12945488	100.000

Supplementary Figure 107. HPLC data of compound (*S*,*S*,*R*)-**3aa**

Compound (*S*,*S*,*S*)-3aa (Fig. 4)

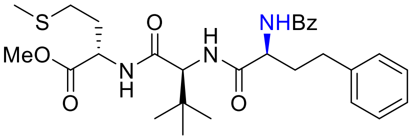

HPLC Conditions
Column: Chiralcel IC, Daicel Chemical Industries, Ltd.
Eluent: Hexanes/Isopropanol (88:12)
Flow rate: 1.0 mL/min
Detection: UV 254 nm

Transamination catalyzed by achiral catalyst 7

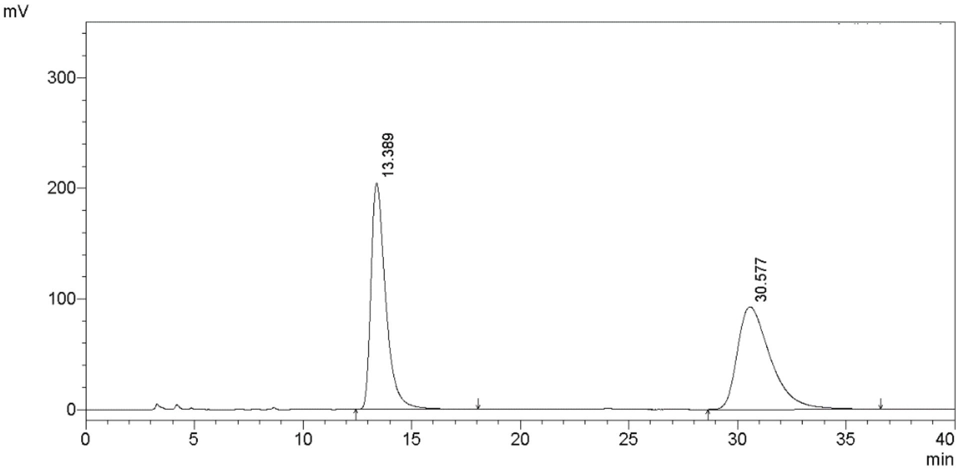

Detector A Ch1 254nm

Peak#	Time	Area	Area %
1	13.389	9634851	49.565
2	30.577	9804008	50.435
		19438859	100.000

Chiral

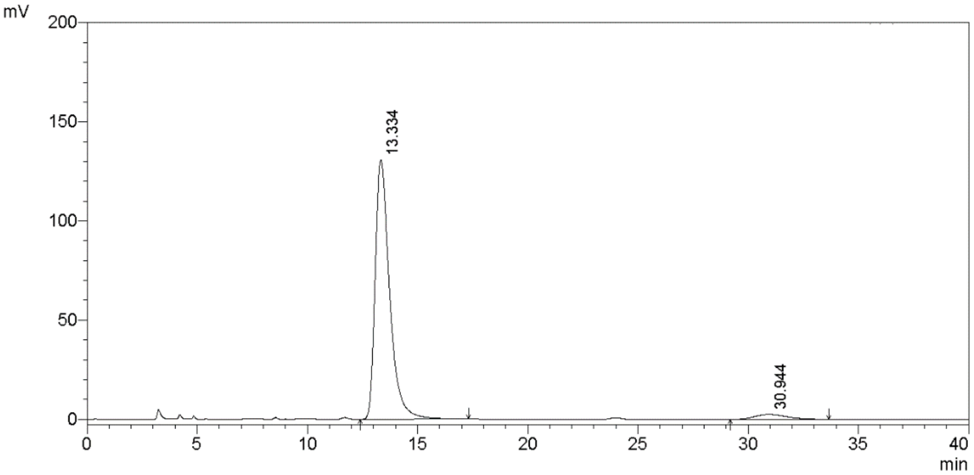

Detector A Ch1 254nm

Peak#	Time	Area	Area %
1	13.334	5956037	95.944
2	30.944	251817	4.056
		6207854	100.000

Supplementary Figure 108. HPLC data of compound (*S*,*S*,*S*)-**3aa**

Compound (*R*,*S*,*R*)-3ab (Fig. 4)

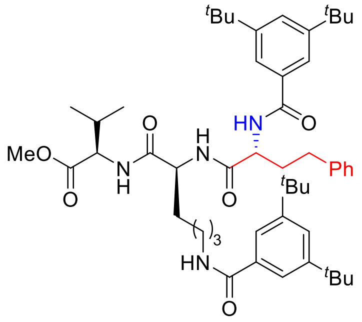

HPLC Conditions
Column: Chiralcel AD-H, Daicel Chemical Industries, Ltd.
Eluent: Hexanes/Isopropanol (94:6)
Flow rate: 0.5 mL/min
Detection: UV 230 nm

Transamination catalyzed by achiral catalyst 7

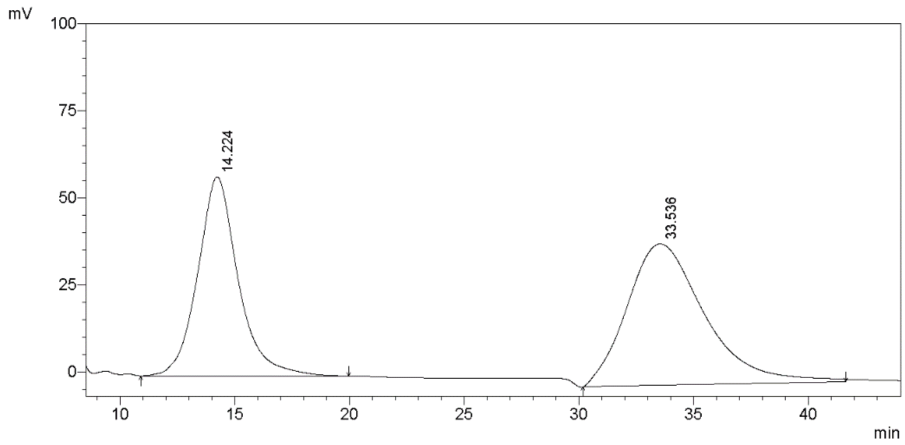

Detector A Ch1 230nm

Peak#	Time	Area	Area %
1	14.224	7037218	42.582
2	33.536	9489033	57.418
		16526250	100.000

Chiral

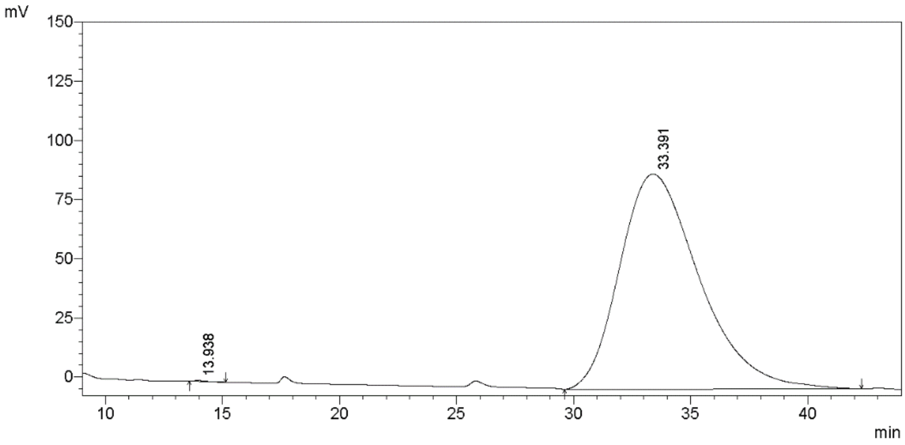

Detector A Ch1 230nm

Peak#	Time	Area	Area %
1	13.938	11922	0.055
2	33.391	21517205	99.945
		21529126	100.000

Supplementary Figure 109. HPLC data of compound (*R*,*S*,*R*)-**3ab**

Compound (*R*,*S*,*S*)-3ab (Fig. 4)

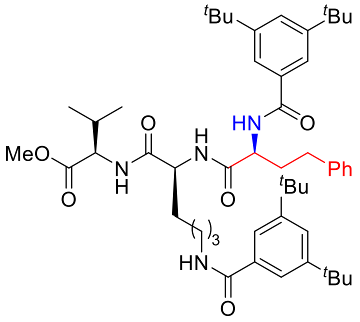

HPLC Conditions
Column: Chiralcel AD-H, Daicel Chemical Industries, Ltd.
Eluent: Hexanes/Isopropanol (94:6)
Flow rate: 0.5 mL/min
Detection: UV 230 nm

Transamination catalyzed by achiral catalyst 7

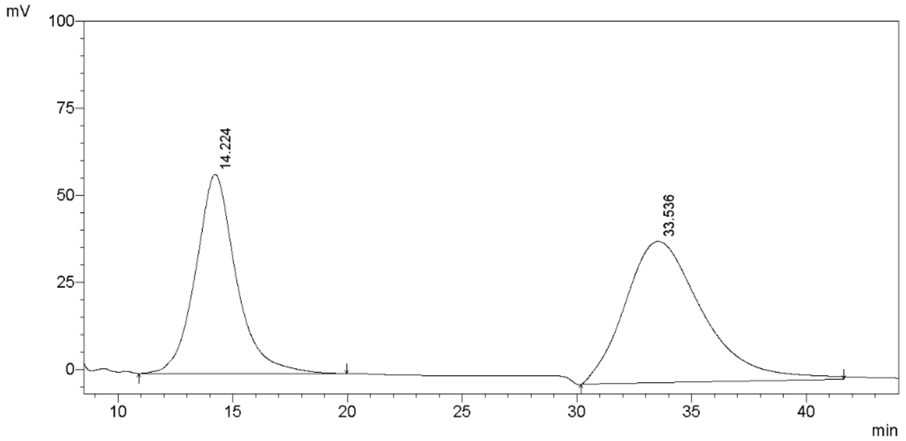

Detector A Ch1 230nm

Peak#	Time	Area	Area %
1	14.224	7037218	42.582
2	33.536	9489033	57.418
		16526250	100.000

Chiral

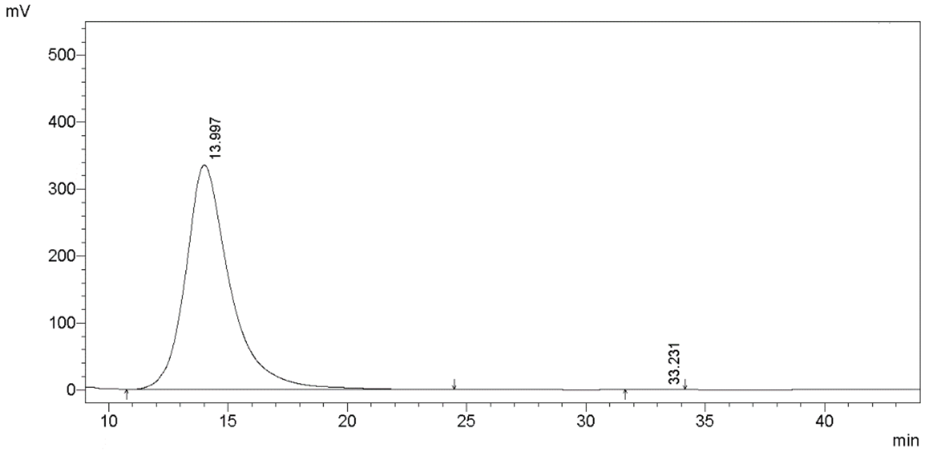

Detector A Ch1 230nm

Peak#	Time	Area	Area %
1	13.997	43695044	99.990
2	33.231	4565	0.010
		43699610	100.000

Supplementary Figure 110. HPLC data of compound (*R*,*S*,*S*)-**3ab**

Compound 3ac (Fig. 4)

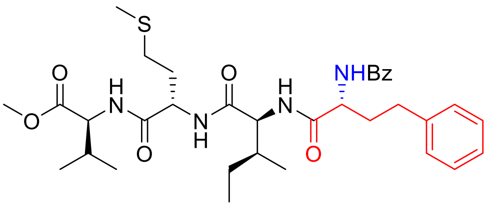

HPLC Conditions

Column: Chiralcel AD-H, Daicel Chemical Industries, Ltd.

Eluent: Hexanes/Isopropanol (92:8)

Flow rate: 0.8 mL/min

Detection: UV 230 nm

Transamination catalyzed by achiral catalyst 7

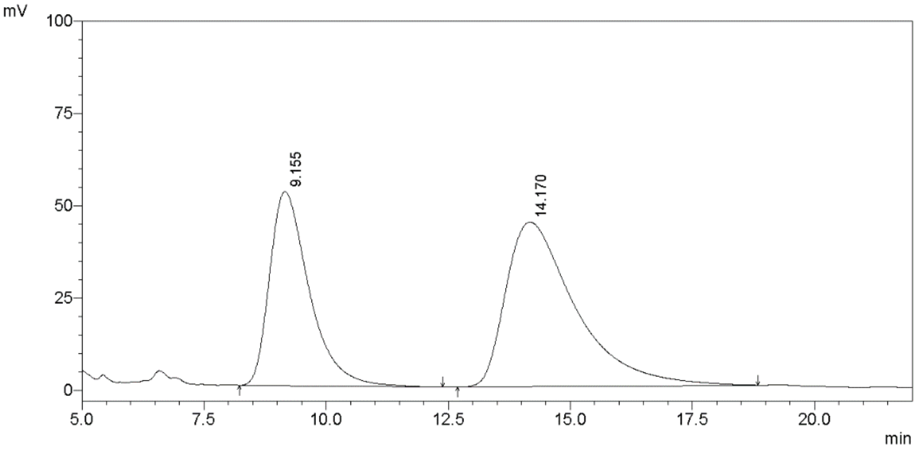

Detector A Ch1 230nm

Peak	Time	Area	Area %
1	9.155	2972442	40.136
2	14.170	4433474	59.864
		7405917	100.000

Chiral

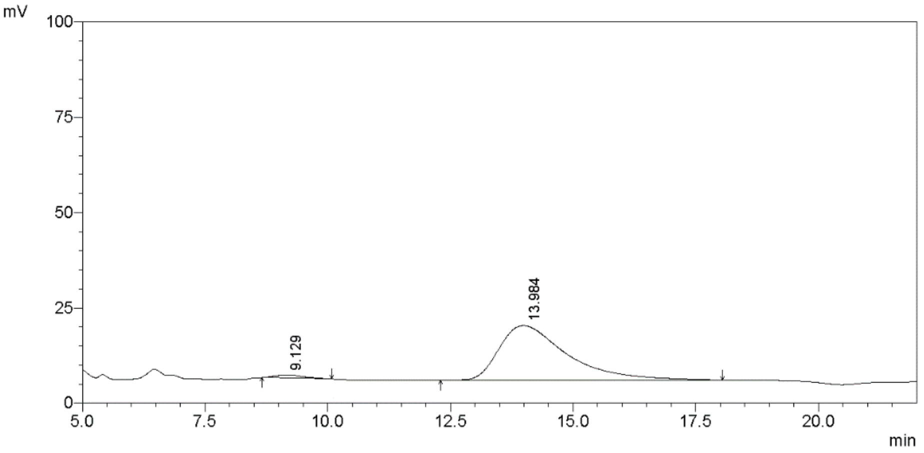

Detector A Ch1 230nm

Peak#	Ret. time	Area	Area %
1	9.129	34264	2.418
2	13.984	1382544	97.582
		1416808	100.000

Supplementary Figure 111. HPLC data of compound **3ac**

Compound 3ae (Fig. 5a)

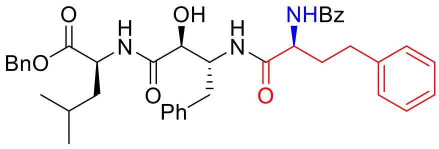

HPLC Conditions

Column: Chiralcel AD-H, Daicel Chemical Industries, Ltd.

Eluent: Hexanes/Isopropanol (88:12)

Flow rate: 1.2 mL/min

Detection: UV 230 nm

Transamination catalyzed by achiral catalyst 7

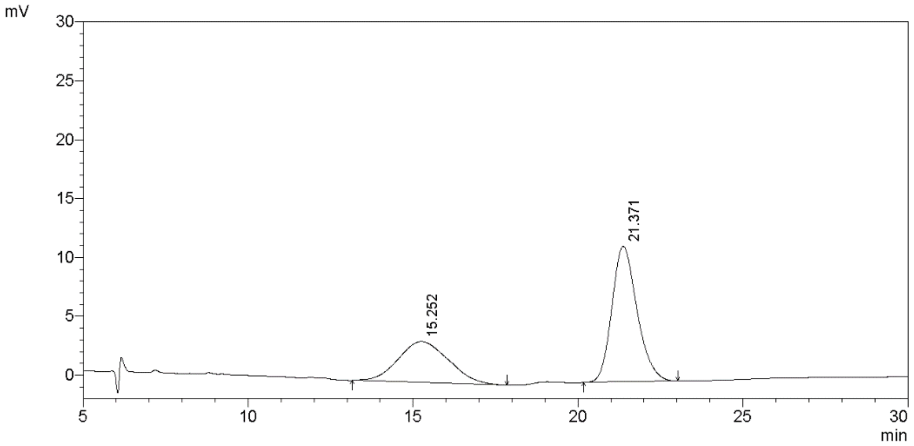

Detector A Ch1 230nm

Peak	Time	Area	Area %
1	15.252	371891	37.749
2	21.371	613276	62.251
		985167	100.000

Chiral

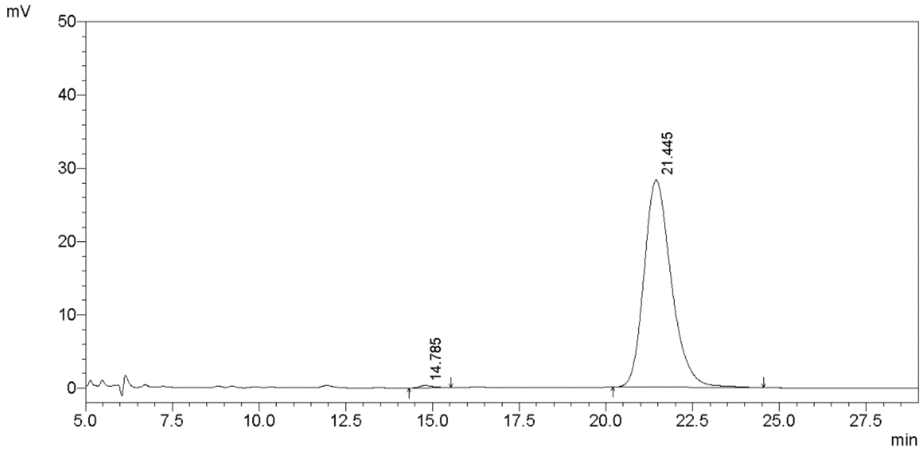

Detector A Ch1 230nm

Peak	Time	Area	Area %
1	14.785	9468	0.608
2	21.445	1548431	99.392
		1557900	100.000

Supplementary Figure 112. HPLC data of compound **3ae**

Compound 3af (Fig. 5a)

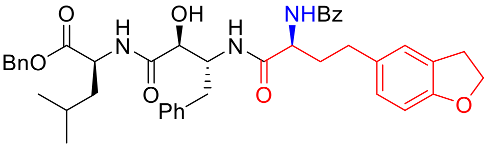

HPLC Conditions

Column: Chiralcel AD-H, Daicel Chemical Industries, Ltd.

Eluent: Hexanes/Isopropanol (85:15)

Flow rate: 1.2 mL/min

Detection: UV 254 nm

Transamination catalyzed by achiral catalyst 7

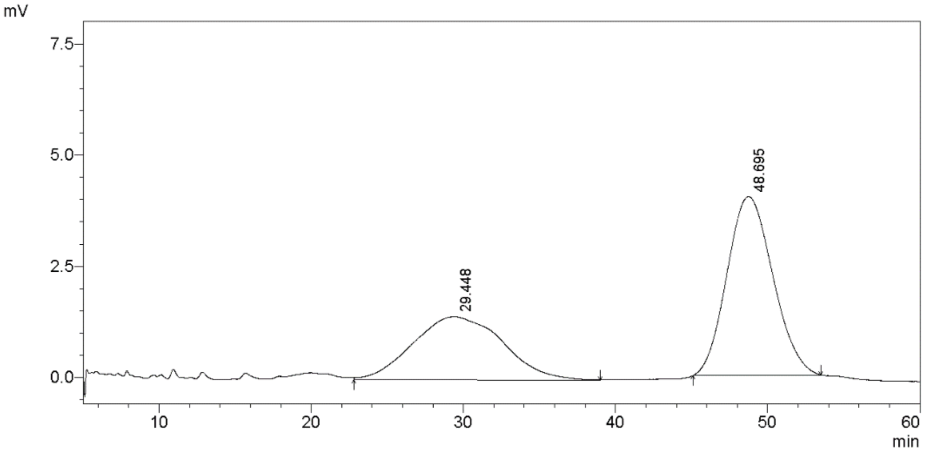

Detector A Ch1 254nm

Peak	Time	Area	Area %
1	29.448	582799	40.848
2	48.695	843953	59.152
		1426752	100.000

Chiral

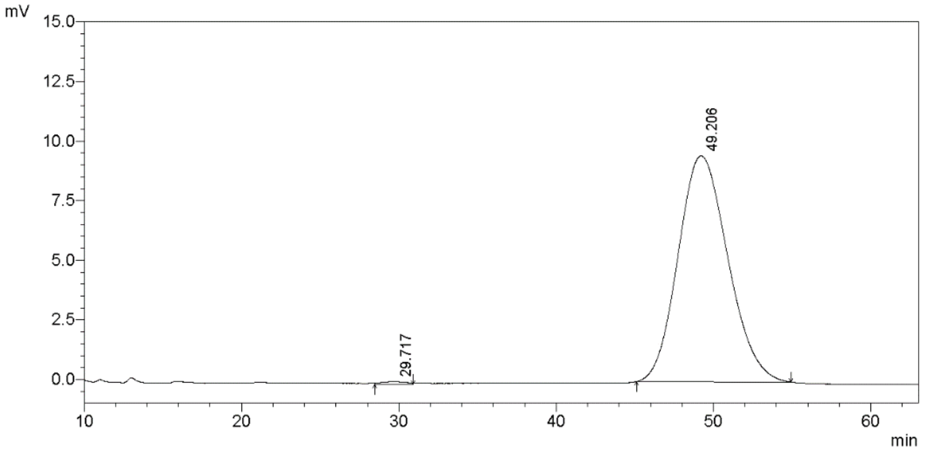

Detector A Ch1 254nm

Peak	Time	Area	Area %
1	29.717	11227	0.536
2	49.206	2083932	99.464
		2095158	100.000

Supplementary Figure 113. HPLC data of compound **3af**

Compound 3ag (Fig. 5a)

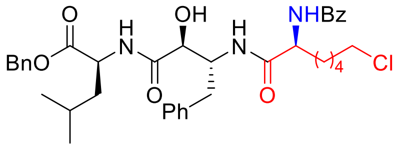

HPLC Conditions

Column: Chiralcel OD-H, Daicel Chemical Industries, Ltd.

Eluent: Hexanes/Isopropanol (96:4)

Flow rate: 0.7 mL/min

Detection: UV 230 nm

Transamination catalyzed by achiral catalyst 7

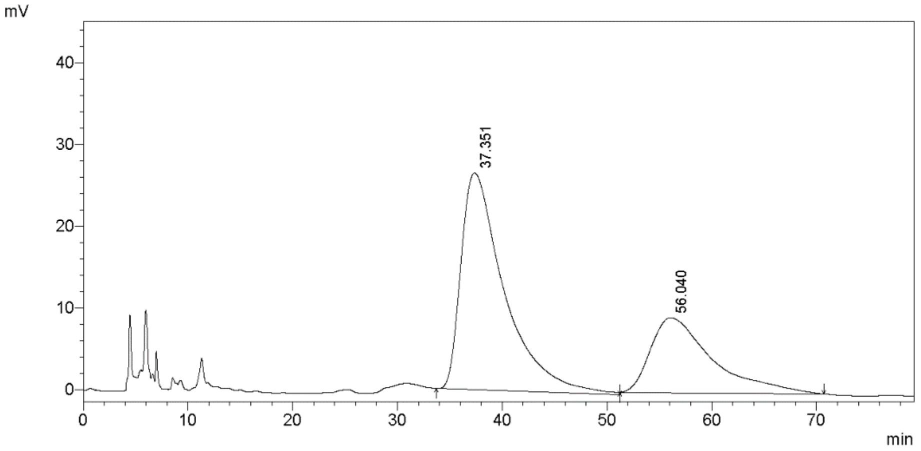

Detector A Ch1 230nm

Peak	Time	Area	Area %
1	37.351	7619314	67.044
2	56.040	3745283	32.956
		11364596	100.000

Chiral

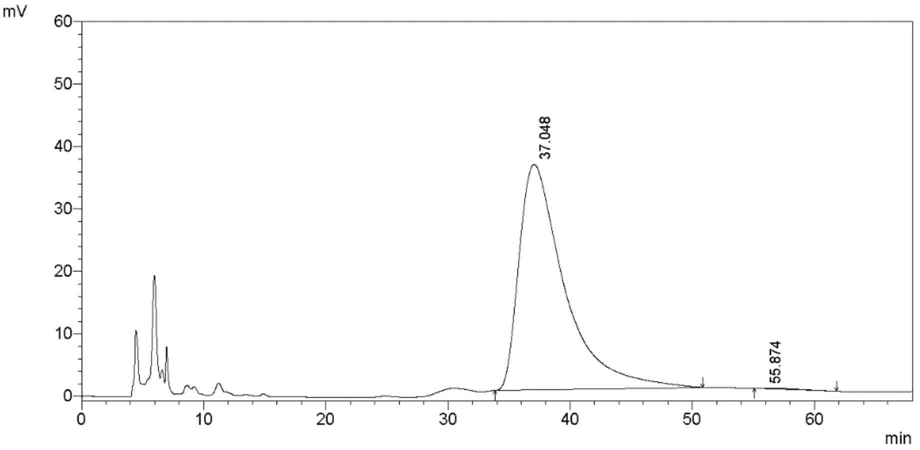

Detector A Ch1 230nm

Peak	Time	Area	Area %
1	37.048	9360129	99.679
2	55.874	30112	0.321
		9390241	100.000

Supplementary Figure 114. HPLC data of compound **3ag**

Compound 3ah (Fig. 5a)

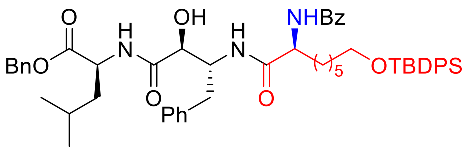

HPLC Conditions

Column: Chiralcel OD-H, Daicel Chemical Industries, Ltd.

Eluent: Hexanes/Isopropanol (95:5)

Flow rate: 1.0 mL/min

Detection: UV 254 nm

Transamination catalyzed by achiral catalyst 7

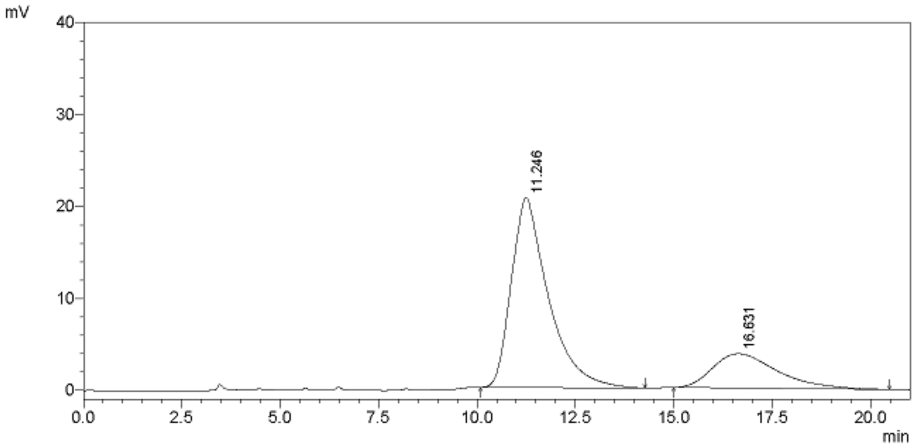

Detector A Ch1 254nm

Peak	Time	Area	Area %
1	11.246	1345716	75.986
2	16.631	425291	24.014
		1771008	100.000

Chiral

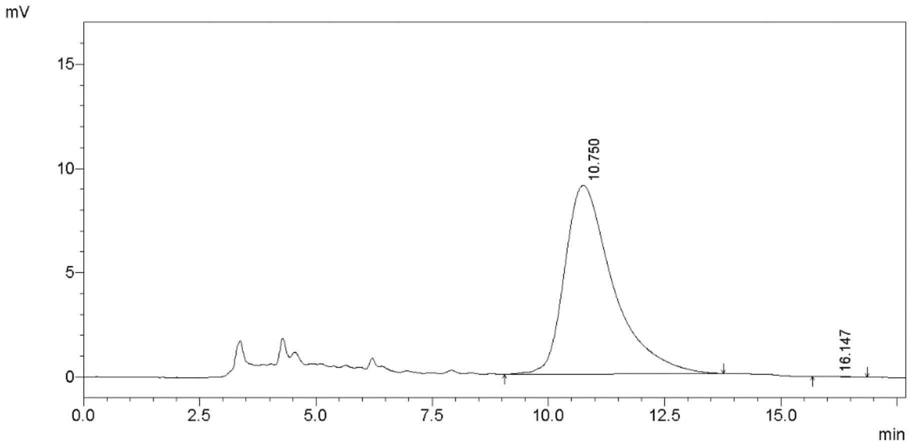

Detector A Ch1 254nm

Peak	Time	Area	Area %
1	10.750	657344	99.853
2	16.147	966	0.147
		658309	100.000

Supplementary Figure 115. HPLC data of compound **3ah**

Compound 3ai (Fig. 5a)

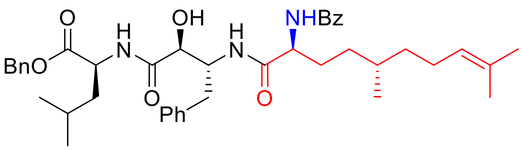

HPLC Conditions
Column: Chiralcel OD-H, Daicel Chemical Industries, Ltd.
Eluent: Hexanes/Isopropanol (95:5)
Flow rate: 0.5 mL/min
Detection: UV 230 nm

Transamination catalyzed by achiral catalyst 7

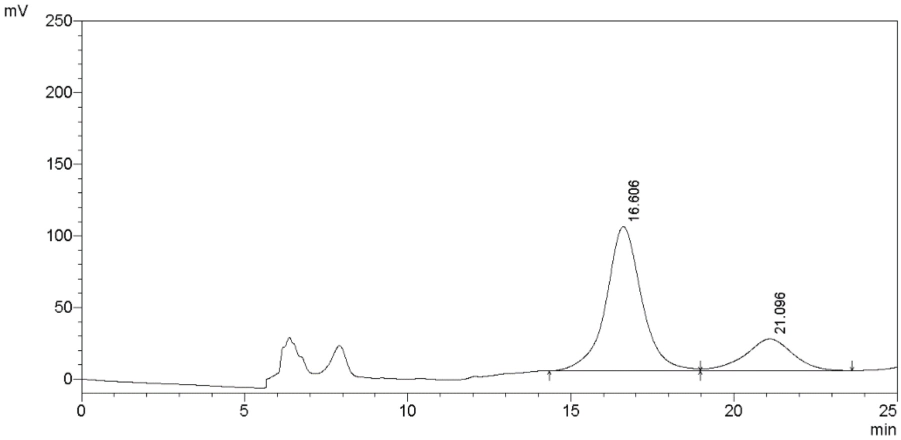

Detector A Ch1 230nm

Peak	Time	Area	Area %
1	16.606	7639011	77.200
2	21.096	2256071	22.800
		9895082	100.000

Chiral

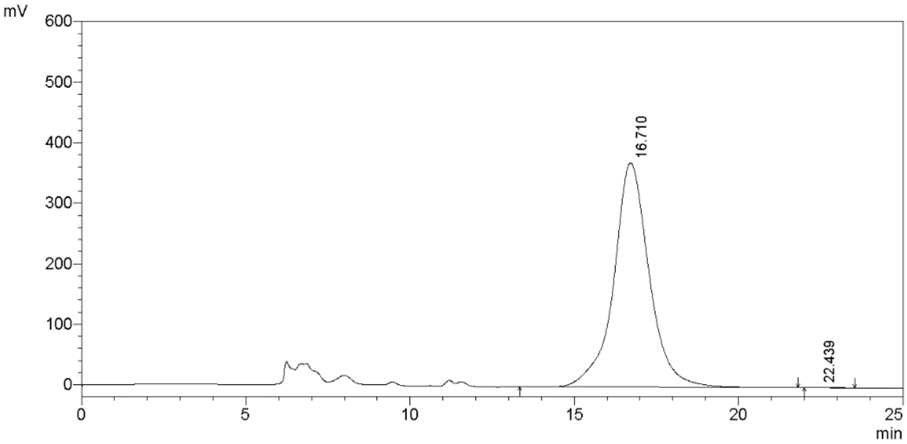

Detector A Ch1 230nm

Peak	Time	Area	Area %
1	16.710	28046708	99.956
2	22.439	12409	0.044
		28059117	100.000

Supplementary Figure 116. HPLC data of compound **3ai**

Compound 3aj (Fig. 5b)

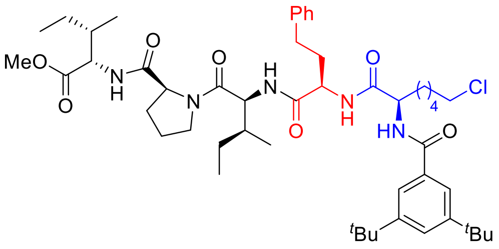

HPLC Conditions
Column: Chiralcel OD-H, Daicel Chemical Industries, Ltd.
Eluent: Hexanes/Isopropanol (93:7)
Flow rate: 1.0 mL/min
Detection: UV 230 nm

Transamination catalyzed by achiral catalyst 7

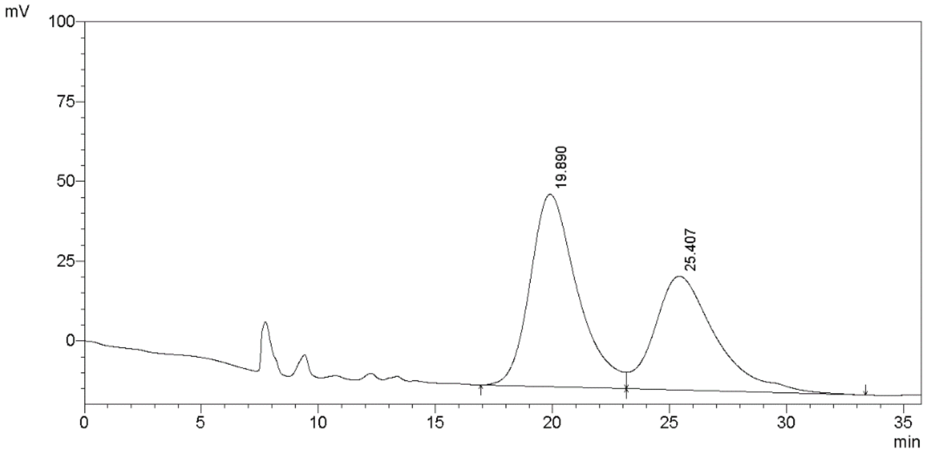

Detector A Ch1 230nm

Peak	Time	Area	Area %
1	19.890	8499495	56.670
2	25.407	6498667	43.330
		14998163	100.000

Chiral

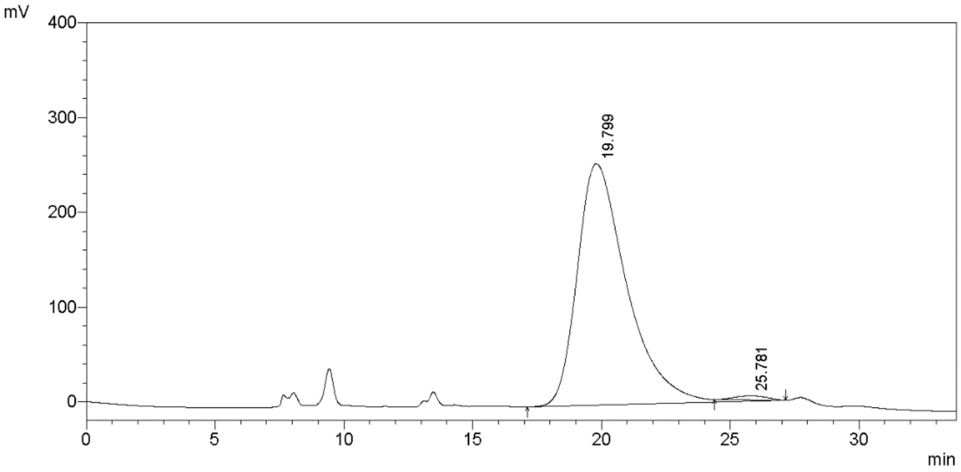

Detector A Ch1 230nm

Peak	Time	Area	Area %
1	19.799	34239271	98.910
2	25.781	377299	1.090
		34616570	100.000

Supplementary Figure 117. HPLC data of compound **3aj**

Compound 3ak (Fig. 5b)

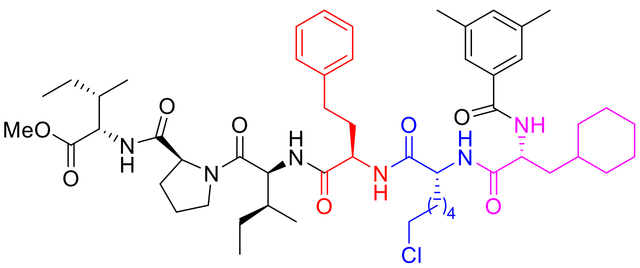

HPLC Conditions

Column: Chiralcel AD-H, DaicelChemical Industries, Ltd.

Eluent: Hexanes/Isopropanol (94:6)

Flow rate: 0.6 mL/min

Detection: UV 230 nm

Transamination catalyzed by achiral catalyst 7

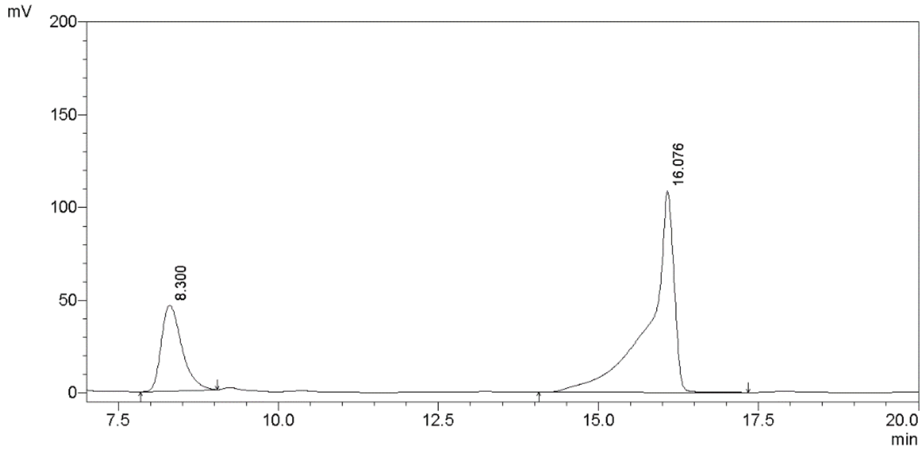

Detector A Ch1 230nm

Peak	Time	Area	Area %
1	8.300	1032103	26.056
2	16.076	2928999	73.944
		3961101	100.000

Chiral

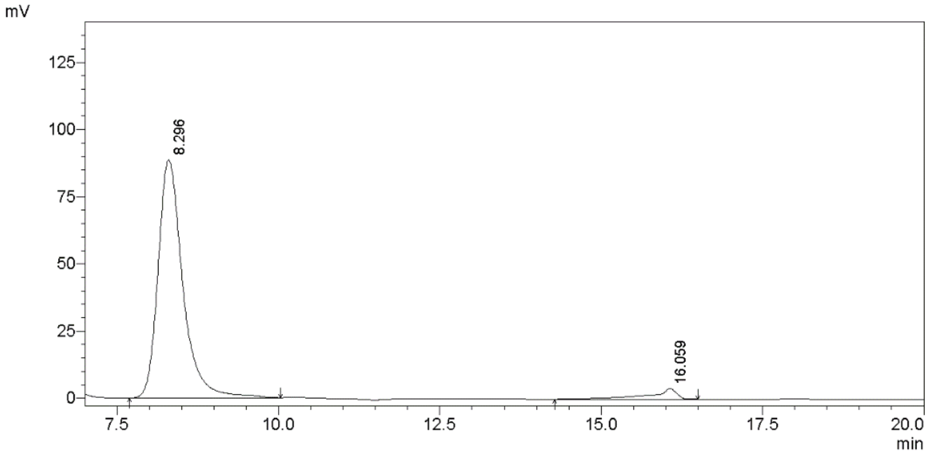

Detector A Ch1 230nm

Peak	Time	Area	Area %
1	8.296	2368120	95.358
2	16.059	115290	4.642
		2483410	100.000

Supplementary Figure 118. HPLC data of compound **3ak**

II. Supplementary References

(1) Liu, Y. E. *et al.* Enzyme-Inspired Axially Chiral Pyridoxamines Armed with a Cooperative Lateral Amine Chain for Enantioselective Biomimetic Transamination. *J. Am. Chem. Soc.* **138**, 10730-10733 (2016).

(2) Chen, J. et al. Carbonyl catalysis enables a biomimetic asymmetric Mannich reaction. *Science* **360**, 1438−1442 (2018).

(3) Grassot, J.-M., Masson, G. & Zhu, J. Synthesis of α-Ketoamides by a Molecular-Sieves-Promoted Formal Oxidative Coupling of Aliphatic Aldehydes with Isocyanides. *Angew. Chem. Int. Ed.* **47**, 947-950 (2008).

(4) Simmons, B. J., Hoffmann, M., Hwang, J., Jackl, M. K. & Garg, N. K. Nickel-Catalyzed Reduction of Secondary and Tertiary Amides. *Org. Lett.* **19**, 1910-1913 (2017).

(5) Sayyadi, N., Skropeta, D. & Jolliffe, K. A. N,O-Isopropylidenated Threonines as Tools for Peptide Cyclization: Application to the Synthesis of Mahafacyclin B. *Org. Lett.* **7**, 5497-5499 (2005).
